# Glycan site loss in two egg-adapted live attenuated influenza vaccine strains does not cause antigenic mismatches

**DOI:** 10.1099/jgv.0.002122

**Published:** 2025-07-03

**Authors:** Jack C. Hirst, Amy Napier, David Burbidge, Katherine Scott, Vivian Lindo, Yasunori Watanabe, Oliver Dibben

**Affiliations:** 1 Flu-BPD, Biopharmaceutical Development, R&D, AstraZeneca, Liverpool, UK; 2 Analytical Sciences, Biopharmaceutical Development, Biopharmaceuticals R&D, AstraZeneca, Cambridge, UK

**Keywords:** egg adaptation, glycosylation, influenza, live attenuated influenza vaccine (LAIV)

## Abstract

The haemagglutinin (HA) proteins of contemporary human H3N2 influenza viruses are heavily glycosylated. Glycans influence the protein’s receptor-binding properties and antigenic profile and can be lost when candidate influenza vaccine strains are propagated in embryonated chicken eggs. Glycan changes in egg-derived vaccine strains have been linked to reduced vaccine effectiveness in inactivated influenza vaccines due to changes in antigenicity, but this has not been investigated in live attenuated influenza vaccines (LAIVs). Here, we determined the impact of egg-adaptive glycosylation changes on the antigenicity of two H3N2 LAIV strains which lost an N-linked glycan site due to egg adaptation: A/New Caledonia/71/2014 (LAIV strain for the 2016–18 influenza seasons) and A/Kansas/14/2017 (LAIV strain for the 2019–20 influenza season). Glycosylation of these egg-adapted HA proteins, along with cell-adapted HA proteins from the same strains, was characterized using nano-liquid chromatography-MS, and their antigenic profiles were assessed with microneutralization assays. We found that the absent glycan sites in the egg-adapted strains were present and occupied by a glycan in the respective cell-adapted strains. Despite this, ferret sera raised against the egg-adapted A/New Caledonia/71/2014 strain were still able to effectively neutralize its glycosylated, cell-adapted counterpart and could also neutralize representatives of most circulating clades from 2016 to 2018. Sera raised against egg-adapted A/Kansas/71/2014 showed reduced cross-reactivity to its cell-adapted counterpart, but this effect was primarily driven by a separate egg adaptation, D190N, rather than the glycosylation change. These data show that glycan loss in LAIV HA proteins due to egg adaptation does not necessarily result in antigenic changes relative to cell-derived viruses.

## Introduction

Influenza is a common respiratory disease caused by the influenza virus which results in up to 650,000 deaths globally each year [1]. The most effective method to combat influenza mortality and morbidity is vaccination [2,4].

Making influenza vaccines is challenging because the epitopes of the primary influenza virus antigen, the receptor-binding protein haemagglutinin (HA), evolve rapidly in a process known as antigenic drift. This drift can result in antigenic mismatches between circulating strains and vaccine strains which, in turn, has been linked to reduced vaccine effectiveness (VE) [5,6]. Influenza vaccines are therefore reformulated annually based on recommendations from the World Health Organization (WHO). Vaccine strains are selected based on the antigenic similarity of their HA proteins to those of strains that are predicted to circulate in the upcoming influenza season. Strains are classified as antigenically similar if the antisera neutralization titres for the test strains are within fourfold of the corresponding neutralization titres of the reference strain [7].

Antigenic drift can be mediated by direct changes to epitopes or by preventing access to those epitopes by allosteric changes. One mechanism underlying such allosteric changes is N-linked glycosylation: a post-translational modification where glycans are added to an N-X-S/T sequon (where X is any amino acid except proline) [8,9]. These glycans take many isoforms, which can be grouped into three categories: (i) linear, high-mannose glycans; (ii) complex glycans with multiple branches; (iii) intermediate, hybrid glycans [10]. The presence of an N-X-S/T sequon does not guarantee that a glycan will be present, nor does the amino acid sequence indicate which isoform the glycan will take. When sites are occupied, the glycans can shield underlying protein epitopes from antibodies and thus influence the antigenic profile of the protein [11,12]. Contemporary H3N2 influenza virus strains typically have 12–13 glycan sites per HA monomer, and the occupied sites together form the HA ‘glycan shield’ [13]. This glycosylation is likely an evolutionary response to escape immune pressure [11,13, 14]. However, HA glycosylation modulates various other processes, such as the receptor-binding activity of the protein and the targeting of infected cells by natural killer cells and mannose-binding lectin [15,18]. The balance between these pressures results in the glycan shield being maintained under strong positive selection [13,19].

Most influenza vaccines are manufactured in embryonated chicken eggs via inoculation of the allantoic cavity [3,20]. Human-adapted influenza A viruses enter cells by binding *α*-2,6-linked sialic acids, but the chorioallantoic membrane of chicken eggs predominantly expresses *α*-2,3-linked sialic acids [21]. Propagating a candidate vaccine strain in eggs therefore often results in the strain acquiring egg-adaptive mutations in its HA protein [22]. The impact of glycosylation on receptor binding means that these egg adaptations often cause the loss of a glycan site, which may, in turn, alter their antigenic profile by exposing immunodominant epitopes that are not accessible on circulating strains [23,24]. However, the impact of these glycan shield changes may vary between vaccine platforms due to their different mechanisms of action. Inactivated influenza vaccines (IIVs) administer antigen intramuscularly, whereas live attenuated influenza vaccines (LAIVs) are administered intranasally and mimic a natural infection. Both platforms elicit serum antibodies, but LAIVs also elicit mucosal IgA and cell-mediated responses [25,26]. Glycosylation changes have been linked to antigenic mismatches and reduced VE for inactivated and recombinant vaccines [24], but the impact of glycan shield changes on LAIV antigenicity and VE has not been characterized.

In this study, we aimed to determine the impact of egg-adaptive glycosylation changes on the antigenicity of recent H3N2 LAIV strains. We selected two strains (from the 2016–18 and 2019–20 influenza seasons) in which an N-linked glycan site had been lost from the HA gene during egg propagation. Glycosylation of these egg-adapted HA proteins, along with cell-adapted HAs from the same strains, was characterized in detail using nano-liquid chromatography-MS (nLC-MS), and the resultant impact of any changes on antigenicity was assessed. Here, we show that H3N2 LAIV strains retained their antigenic character despite the loss of glycosylation sites due to egg adaptation.

## Methods

### Cells and eggs

Madin–Darby canine kidney (MDCK) cells, MDCK-SIAT1 cells and HEK-293T cells were cultured and maintained in Eagle’s Minimum Essential Medium (EMEM) (BioWhittaker; Lonza; Cat. No. BE12-662F) containing non-essential amino acids and sodium pyruvate and supplemented with 10% heat-inactivated FBS (v/v) (Gibco; Thermo Fisher Scientific; Cat. No. 10500056), 1% penicillin-streptomycin (v/v) (Gibco; Thermo Fisher Scientific; Cat. No. 15140122) and 1% 200 mM l-glutamine (v/v) (Gibco; Thermo Fisher Scientific; Cat. No. 25030018).

Specific pathogen-free embryonated hen’s eggs were obtained from Charles River Laboratories, Wilmington, USA. Eggs were incubated at 37 °C with rotation and 70% humidity for 10–11 days prior to being inoculated.

### Virus strains

The amino acid differences between the HA proteins of the strains in Table 1 are described in Table S2, available in the online Supplementary Material. Amino acid numbering throughout refers to the mature H3 protein sequence [27].

**Table 1.** List of all virus strains and abbreviations used in this study

Virus	Abbreviation	Accession
A/Kansas/14/2017 (egg)	eKAN17	EPI_ISL_316455
A/Kansas/14/2017 (cell)	cKAN17	EPI_ISL_292575
A/New Caledonia/71/2014 (egg)	eNC14	Derived from EPI_ISL_168901, with added egg-adaptive changes listed in Table 2
A/New Caledonia/71/2014 (cell)	cNC14	EPI_ISL_168901
A/Hong Kong/4801/2014	A/HK4801	EPI_ISL_198222
A/Hong Kong/5737/2014	A/HK5737	EPI_ISL_166844
A/Victoria/5060/2014	A/VIC14	EPI_ISL_168962
A/Singapore/INFIMH-16-0019/2016	A/INF16	EPI_ISL_285897
A/South Australia/135/2016	A/SA16	EPI_ISL_239828
A/Greece/4/2017	A/GRE17	EPI_ISL_257379
A/Singapore/GP2646/2016	A/GP2646	EPI_ISL_240797
A/Brisbane/190/2017	A/BRIS190	EPI_ISL_293803
A/Washington/16/2017	A/WASH17	EPI_ISL_275706
A/Switzerland/8060/2017	A/SWITZ17	EPI_ISL_331924
A/England/70180215/2016	A/ENG215	EPI_ISL_248548

Egg-grown virus strains were provided by the WHO. HA and neuraminidase (NA) genes were amplified by RT-PCR and cloned into a pAD3000 vector as previously described [28]. Cell-adapted HA genes, and NA genes where the cell-adapted sequence differed from the egg-adapted sequences, were ordered as GeneArt DNA Fragments (Thermo Fisher Scientific) and cloned into a pAD3000 vector in the same manner as the RT-PCR products.

Viruses were rescued as LAIV strains using an eight-plasmid reverse genetics system, comprising strain-specific HA and NA segments and six internal segments (PB2, PB1, PA, NP, M and NS) from the cold-adapted, temperature-sensitive, attenuated A/Ann Arbor/6/1960 master donor virus as previously described [28]. Egg-adapted strains were rescued in HEK-293T/MDCK co-cultures in accordance with standard procedures for candidate vaccine characterization of LAIVs. They were then passaged twice in the allantoic cavity of 10- to 11-day-old embryonated hen’s eggs at 33 °C and 70% humidity. As cell-adapted H3N2 strains from 2016 to 2020 typically showed poor replication in MDCK cells [29], these were instead rescued in HEK-293T/MDCK-SIAT1 co-cultures and passaged once in MDCK-SIAT1 cells at 33 °C and 5% CO_2_.

To confirm that no mutations had been acquired during propagation, viral RNA was extracted using an RNeasy Mini Kit (QIAGEN), and RT-PCR was performed on the HA and NA genes using a SuperScript III One-Step RT-PCR System with Platinum Taq DNA Polymerase (Thermo Fisher Scientific). Sanger sequencing of the resulting amplicons was performed by Azenta Life Sciences.

### Recombinant proteins

Recombinant HA proteins were obtained from the Native Antigen Company. The transmembrane and cytoplasmic domains were removed and replaced with a T4 foldon and C-terminal 6x-His-tag on a glycine series linker. Proteins were produced in HEK293 cells and purified by immobilized metal affinity chromatography and buffer exchange.

### Antisera generation

All ferret antisera were originally generated for candidate vaccine characterization during previous FluMist/Fluenz manufacturing seasons, and leftover antisera were repurposed for this study.

Four male ferrets (for serum raised against A/New Caledonia/71/2014) or two female ferrets (for serum raised against A/Kansas/14/2017) aged 4–6 months were sourced from a UK Home Office-accredited supplier (Marshall’s Biosciences, UK) and group-housed at Advisory Committee on Dangerous Pathogens containment level 2. Cages met with the UK Home Office Code of Practice for the Housing and Care of Animals Bred, Supplied or Used for Scientific Procedures (December 2014). Access to food and water was *ad libitum*, and environmental enrichment was provided. Before inoculation with A/New Caledonia/71/2014, ferrets were confirmed seronegative for A/New Caledonia/71/2014 by haemagglutination inhibition (HAI) assay. Before inoculation with A/Kansas/14/2017, ferrets were confirmed seronegative for A/Singapore/Infimh-16-0019/2016 by HAI assay.

Vaccine material was intranasally administered at 1 ml (~0.5 ml/naris) per ferret, following light sedation with isoflurane. The vaccine materials used for antisera generation were high-dose (≥7.0 log10 FFU/animal) LAIV. Fourteen days post-vaccination, ferrets were anaesthetized with intramuscular injection of ketamine/xylazine (17.9 and 3.6 mg kg^−1^ bodyweight), and exsanguination was effected via cardiac puncture, followed by injection of an anaesthetic overdose (sodium pentobarbitone, Dolethal, Vetoquinol UK Ltd, 140 mg kg^−1^). Sera were prepared from whole blood collected prior to overdose.

### Fluorescent focus assay

Viruses were titrated by performing a threefold dilution series of the stock in 1× EMEM supplemented with 1% penicillin-streptomycin (v/v) and 1% 200 mM l-glutamine (v/v) and applying to SIAT cells in a 96-well plate format. Cells were incubated for 16–18 h in the absence of trypsin. Cells were washed twice in 1× PBS before being incubated in 100% ice-cold methanol for 20 min to fix and permeabilize. Fixed plates were washed twice in PBS and blocked in 1% BSA (Sigma, A-2153) in PBS for 1 h. Mouse anti influenza A nucleoprotein primary antibody (BioRad, MCA400) was conjugated to DyLight 650 NHS Ester with a Microscale Protein Labelling Kit (Thermo Fisher) per the manufacturer’s instructions, then diluted 1 : 1,000 in 1% BSA and applied to fixed cells for 1 h. Cells were washed twice in 1× PBS before adding Hoechst 33342 at a final concentration of 1 in 20,000 and incubating at room temperature (RT) for at least 5 min.

Plates were imaged using a Cytation 7 Cell Imaging Multi-Mode Reader at 4× magnification. A stitched image of 6.7 mm^2^ was captured, and fluorescent foci were counted. Infectivity was calculated by dividing the DyLight 650 foci count by the Hoechst count.

### Microneutralization assay

The microneutralization assay was adapted from [30]. One hundred microlitres of ferret serum were added to 250 µl of 2× receptor-destroying enzyme (Deben Diagnostics, Cat. No. 370013) and incubated at 37 °C for 18–20 h. 225 µl EMEM (supplemented with 1% penicillin-streptomycin (v/v) and 1% 200 mM l-glutamine (v/v) was added and then the mixture incubated at 56 °C for 45 min. Sera were serially diluted twofold (NC14) or threefold (KAN17), mixed with an equivalent volume of virus (at a final titre of 10% infectivity) and incubated for 1 h. Virus/antisera mixtures were added to SIAT cells and incubated and stained per the fluorescent focus assay protocol above.

Neutralization titres were calculated by first normalizing foci counts to a no-antisera control. Standard curves were interpolated by using the sigmoidal, four-parameter logistic model in GraphPad Prism 9, and the antiserum dilution required to reduce the no-antisera control foci count by 50% (the MN_50_) was interpolated (example shown in Fig. S3). Fold differences were calculated by expressing the neutralization titre of the test strain as a proportion of the neutralization titre of the homologous strain for each ferret.

### Statistical analysis

To compare MN_50_ titres of NC14 and its associated test strains, for each strain, statistical significance was calculated by first assessing normality using a Shapiro–Wilk test. As the MN_50_ values for eNC14 were not normally distributed, comparisons between the homologous and test strains were performed using either a two-tailed Wilcoxon matched-pairs signed rank test (when comparing to a single test strain) or a Friedman test followed by Dunn’s multiple comparison test. Statistical comparisons of the MN_50_ titres for KAN17 strains were not performed as there were insufficient independent samples to assess how the values were distributed.

### Glycan analysis by nLC-MS

All chemicals and reagents were purchased from Thermo Fisher Scientific unless otherwise specified. HA proteins were denatured and reduced at 37 °C for 30 min in 90 mM Tris, pH 7.6 buffer containing 7.2 M GdnHCl, 0.1 mM EDTA and 45.5 mM dithiothreitol. Alkylation was followed by the addition of 500 mM iodacetamide for a further 30 min at RT in the dark. The alkylated HA was buffer-exchanged into 150 mM Tris, pH 7.6 for 2 h at RT using a 10K molecular weight cut-off microdialysis device. The recovered proteins were digested separately with either trypsin (Trypsin Gold MS Grade, Promega), chymotrypsin (Mass Spectrometry Grade, Promega) and endoproteinase GluC (Sequencing Grade, Promega) or elastase (Promega) at a ratio of 1 : 30 (w/w) at 37 °C for 16 h. Reaction mixtures were dried, and glycopeptides were resuspended in 0.1% formic acid prior to MS analysis by nanoLC-ESI MS with a nano Liquid Chromatography (LC) system coupled to an Orbitrap Fusion mass spectrometer.

Glycopeptides were separated using an EasySpray PepMap RSLC C18 column (75 µm×75 cm) with a 240 min linear solvent gradient of 0–32% acetonitrile in 0.1% formic acid, followed by 35 min of 80% acetonitrile in 0.1% formic acid. Other settings include an LC flow rate of 200 nl min^−1^, spray voltage of 2.8 kV, capillary temperature of 275 °C and a Higher-energy collisional dissociation collision energy of 50%. Precursor and fragmentation detection were performed using an Orbitrap at the following resolution: MS1=100,000 and MS2=30,000. The automatic gain control targets were MS1=4e5 and MS2=5e4, and injection times were MS1=50 and MS2=54. The following cleavage sites were used for the respective proteases: trypsin=R/K, chymotrypsin=F/Y/W, alpha lytic protease=T/A/S/V and Glu C=E/D. The number of missed cleavages was set at 3. The following modifications were also included: carbamidomethyl (+57.021464, target=C, fine control=fixed), oxidation (+15.994915, target=M, fine control=variable rare 1), Glu to pyro-Glu (−18.010565, target=peptide N-term E, fine control=variable rare 1) and Gln to pyro-Glu (−17.026549, target=peptide N-term Q, fine control=variable rare 1). Glycopeptide fragmentation data were extracted from raw files using Byonic^™^ (Version 3.5.0) and Byologic^™^ (Version 3.5-15; Protein Metrics Inc.). Glycopeptide fragmentation data were manually evaluated with true-positive assignments given when correct b- and y-fragments and oxonium ions corresponding to the peptide and glycan, respectively, were observed. The precursor mass tolerance was set at 4 ppm for precursor ions and 10 ppm for fragment ions. MS data were searched using a glycan library with the identical peptide sequence. A 1% false discovery rate was applied. The extracted ion chromatographic areas for each true-positive glycopeptide, with the same amino acid sequence, were compared to determine the relative quantitation of glycoforms at each specific N-linked glycan site.

### Protein modelling

The Swiss protein models were generated in Coot [31] using a crystal structure of HA A/Victoria/361/2011 (PDB ID: 4WE8) [18]. Complex-, hybrid- and oligomannose-type N-linked glycans (PDB ID 4BYH, 4B7I and 2WAH) were modelled onto the N-linked attachment site, to represent the most dominant isoform. The glycosylated HA protein structure was then imported into the Schrödinger software where substitutions identified in our sequences were mutated using the mutagenesis wizard. The visualization of the predicted model was done in PyMOL [32] showing N-linked glycosylation sites and their identified glycan structures and known antigenic sites A–E [33].

## Results

### Egg adaptation caused the loss of glycans near the receptor-binding site and antigenic site B in two H3N2 LAIV HA proteins

To assess the impact of glycosylation changes on the antigenicity of recent H3N2 LAIV strains, we examined two vaccine strains which lost an HA glycosylation site upon egg adaptation. The 2016–17 and 2017–18 H3N2 LAIV strains were egg-adapted A/New Caledonia/71/2014 (eNC14) from clade 3C.2a. The HA protein of eNC14 had three amino acid changes relative to its cell-derived counterpart (cNC14) (Table 2), of which T160K removed the N158 glycan site. The 2019–20 H3N2 LAIV strain was egg-adapted A/Kansas/14/2017 (eKAN17). It had two HA amino acid changes relative to its cell-derived counterpart (cKAN17) (Table 2), of which N246T removed the N246 glycan site. Missing glycan sites in the egg-adapted HA proteins represented the most likely change to the glycan shield but would only represent a change if the corresponding glycan sites in the respective cell-adapted protein were occupied. Similarly, sites may be present in both proteins but only occupied in one, which would also represent a change to the shield. To detect such changes, we assessed the composition and occupancy of the HA protein glycan sites using nLC-MS. Recombinant HA proteins for the egg and cell variants of each strain were expressed in HEK-293T cells and proteolytically digested for nLC-MS, allowing site-specific characterization of glycan composition.

**Table 2.** HA amino acid differences between egg-derived H3N2 LAIV strains and the corresponding cell-derived strains. Amino acid numbering is of the mature H3 protein [27]. When a difference occurs within a glycan site, the name of the site is indicated in brackets

	2016–18 seasonA/New Caledonia/71/2014 (NC14)			2019–20 seasonA/Kansas/14/2017 (KAN17)	
HA residue	160 (N158)	183	225	190	246 (N246)
Cell-adapted (c)	T	H	D	D	N
Egg-adapted (e)	K	L	G	N	T

**Fig. 1.**
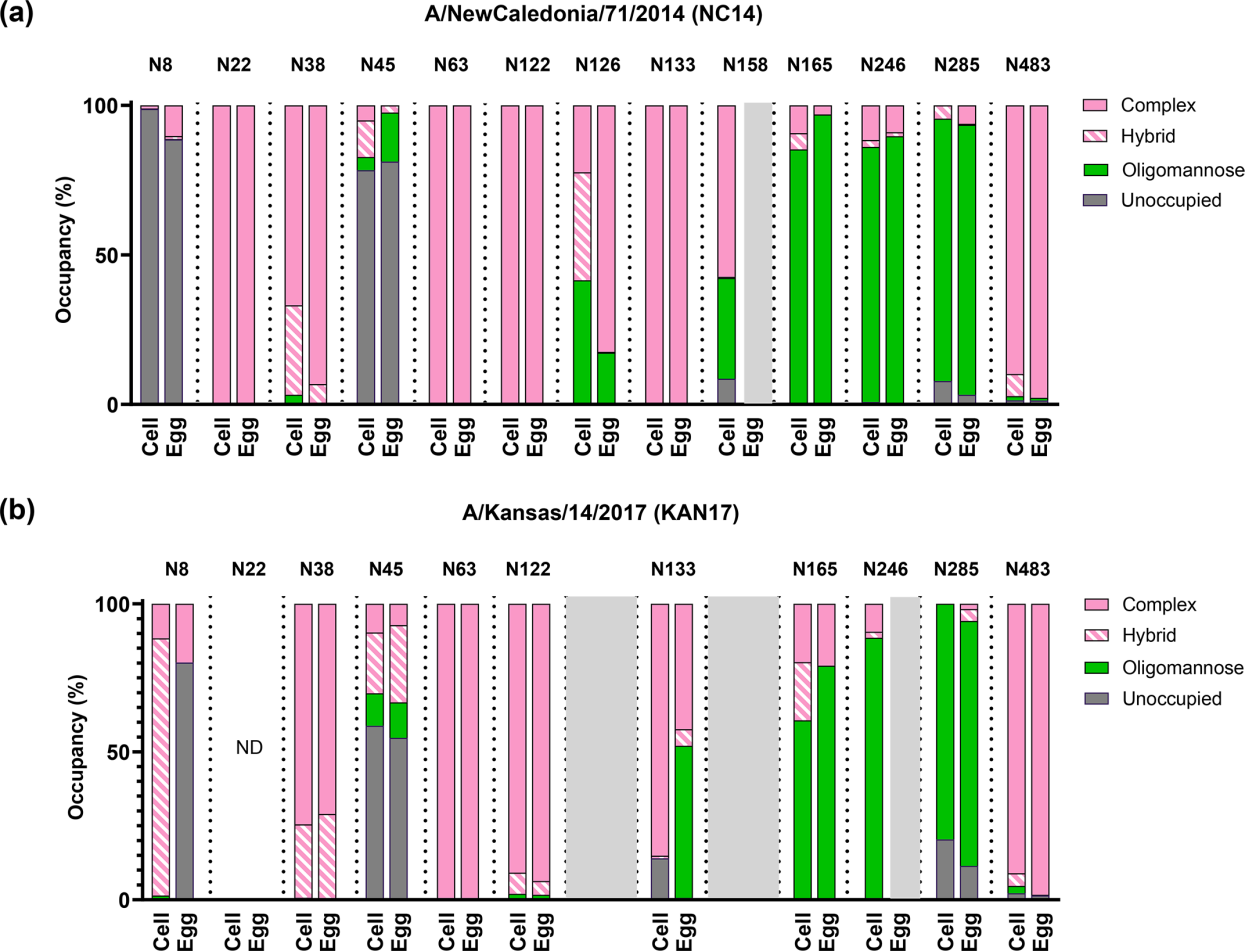
Egg adaptation changed the glycan shield of NC14 and KAN17. Recombinant HA proteins of cell- and egg-adapted variants of (a) NC14 and (b) KAN17 were proteolytically digested and analysed by nLC-MS. Graphs indicate the distribution of glycan categories identified at individual N-linked glycan sites, which are coloured per the key. Grey shading indicates no glycan site is present. Glycan sites where composition could not be determined are labelled as not determined (ND). Isoform-specific analysis of each site is shown in Figs S1 and S2.

Across all four HA proteins, most glycan sites were occupied (Fig. 1). When present, the sites at N22, N38, N63, N122, N126, N133, N158 and N483 were predominantly occupied by complex or hybrid glycans, whereas the sites at N165, N246 and N285 were predominantly occupied by oligomannose glycans. Predominantly unoccupied sites were seen in eNC14, cNC14 and eKAN17 at N8 and in all proteins at N45.

When glycan sites were present in cNC14 and eNC14 HAs (Figs 1a and S1), only N126 showed differences in occupancy, with cNC14 N126 occupied by a mix of oligomannose, hybrid and complex glycans, whereas eNC14 N126 was predominantly occupied by complex glycans. The N158 site, absent in eNC14, was occupied by either oligomannose or hybrid glycans in cNC14.

Glycan occupancy was generally similar between the cKAN17 and eKAN17 HA proteins for sites which were present in both (Figs 1b and S2). Differences were present at N133, which was predominantly occupied by complex glycans in cKAN17 HA but was a mix of complex and oligomannose glycans in eKAN17, and N8 which was occupied by hybrid glycans in cKAN17 and unoccupied in eKAN17 HA. The N246 site, absent in eKAN17, was occupied by oligomannose glycans in cKAN17.

Taken together, the analysis of egg- and cell-derived HA proteins of NC14 and KAN17 identified three sites which were occupied by glycans in a cell-adapted HA protein but were either absent or unoccupied in an egg-adapted HA protein: N158 in NC14, N8 in KAN17 and N246 in KAN17.

**Fig. 2.**
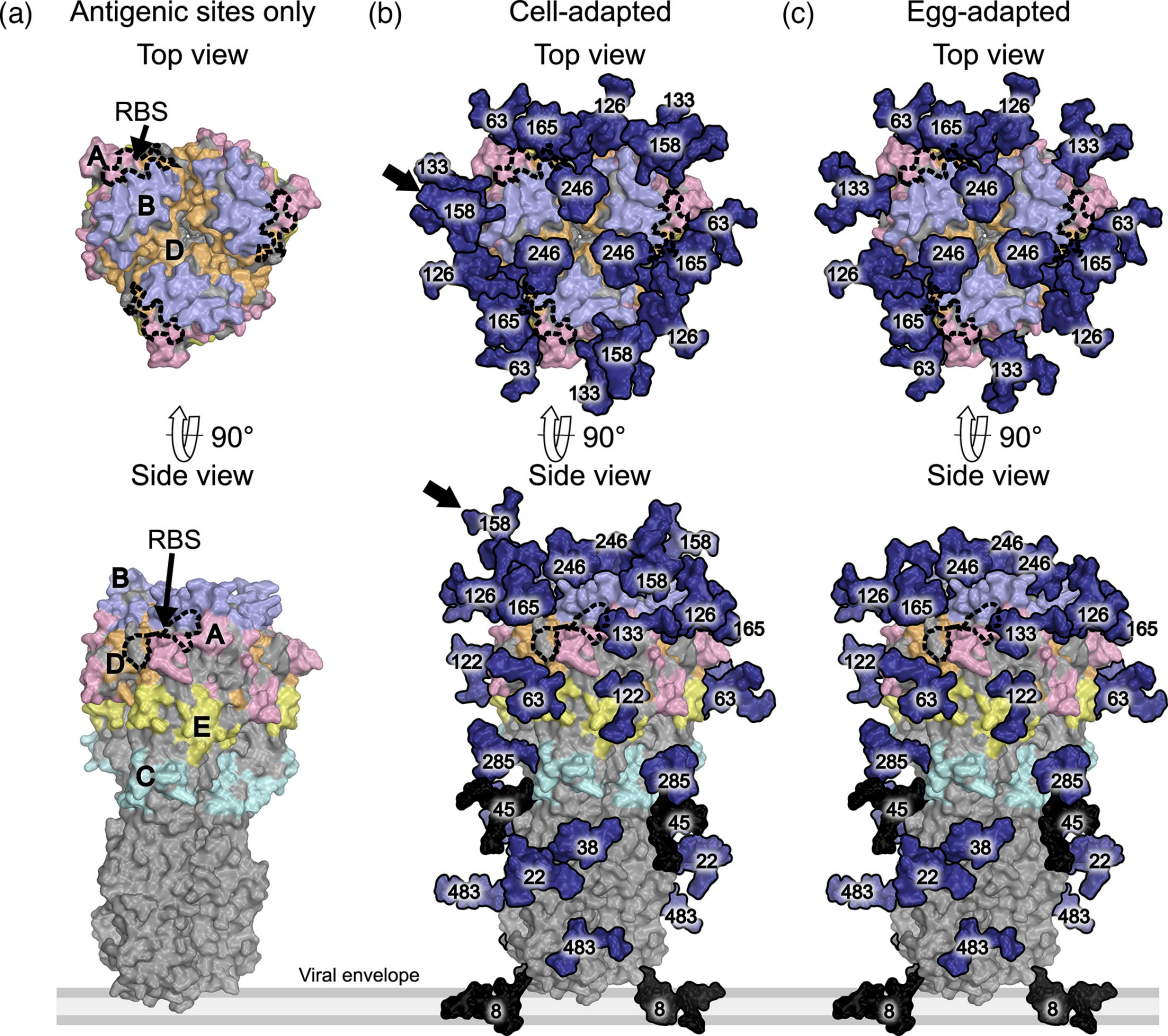
Models of glycosylated egg- and cell-adapted NC14 HA proteins. Experimentally observed NC14 glycans from Figure 1a are modelled on the prefusion structure of trimeric A/Victoria/361/2011 (PDB ID: 4WE8, (Yang et al., 2015)). The receptor binding site (RBS) is indicated by the dashed black line, antigenic sites A – E (Ndifon et al., 2009) are coloured per panel (a), glycans are coloured in navy, unoccupied glycan sites coloured in black, and the protein surface is coloured in grey. The black arrows in panel (b) indicate glycans that differ between the cell- and egg-adapted strains.

To assess the potential antigenic impact of changes to N8, N158 and N246, we generated HA protein models in PyMOL [32] (Figs2  3) containing the five major antigenic sites of H3 proteins [33] and populated them with the experimentally defined glycans from the MS analysis.

**Fig. 3.**
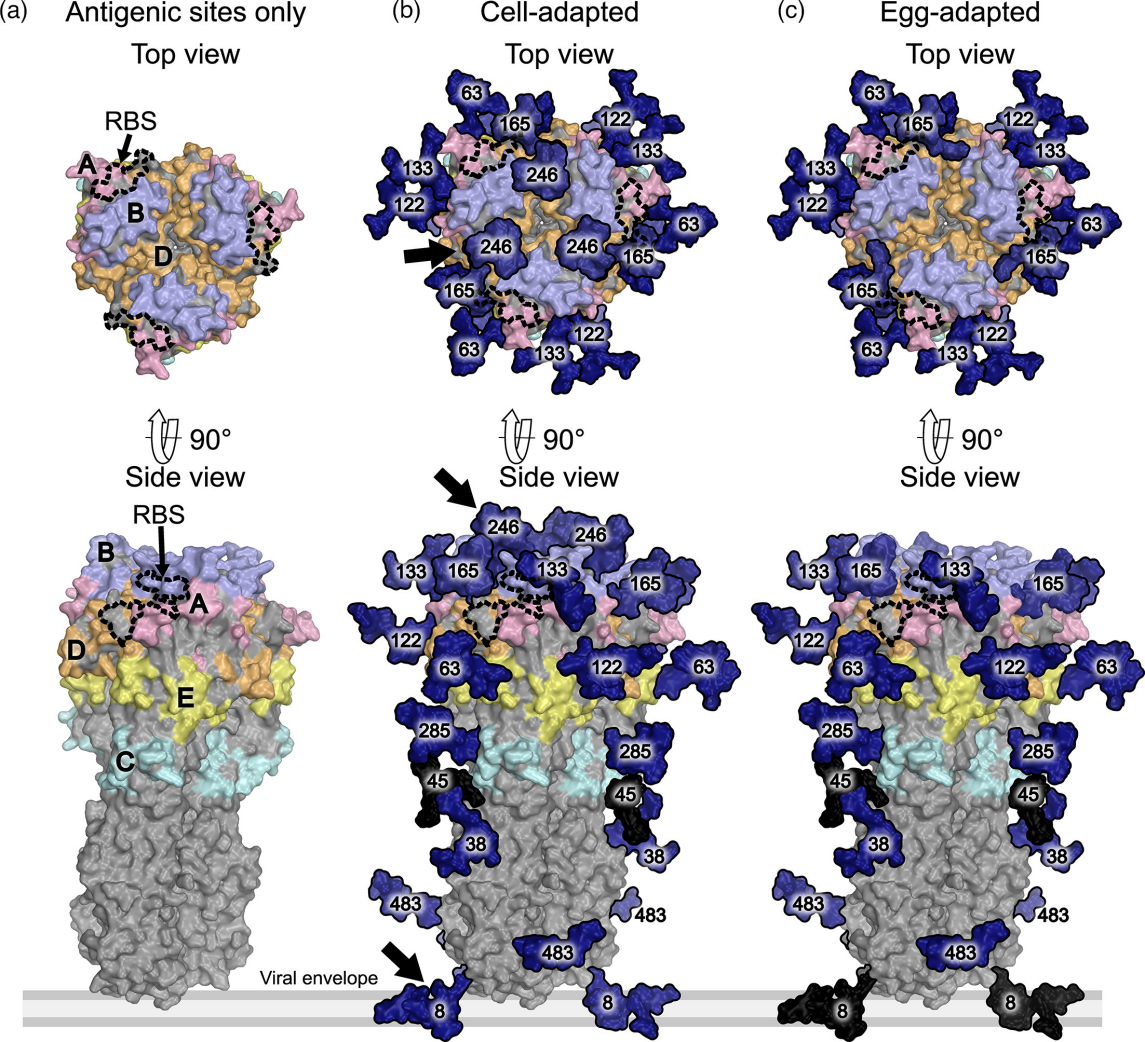
Models of glycosylated egg- and cell-adapted KAN17 HA proteins. Experimentally observed KAN17 glycans from Figure 1b are modelled on the prefusion structure of trimeric A/Victoria/361/2011 (PDB ID: 4WE8, (Yang et al., 2015)). The receptor binding site (RBS) is indicated by the dashed black line, antigenic sites A – E (Ndifon et al., 2009) are coloured per panel (a), glycans are coloured in navy, unoccupied glycan sites coloured in black, and the protein surface is coloured in grey. The black arrows in panel (b) indicate glycans that differ between the cell- and egg-adapted strains.

The N158 glycan in cNC14 was located in antigenic site B and in close proximity to the receptor-binding site (Fig. 2). Antigenic site B is the immunodominant epitope of recent H3N2 strains [34,35] and so the absence of the N158 glycan in eNC14 exposed residues of antigenic site B that were shielded on cNC14.

The N8 glycan that was unoccupied in eKAN17 HA was located near the base of the protein and therefore distant from the major antigenic sites and the receptor-binding site (Fig. 3). Conversely, the N246 glycan was in close proximity to the receptor-binding site and adjacent to antigenic site B. The loss of the N246 glycan in eKAN17 HA could therefore have altered the protein’s antigenic profile relative to that of cKAN17 HA.

### Glycan shield changes did not affect the antigenicity of eNC14 and eKAN17 LAIV viruses

We then considered whether alterations to the glycan shields would lead to egg-adapted LAIV strains eliciting antisera with reduced cross-reactivity to cell-adapted strains. Antisera against eNC14 and eKAN17 LAIV strains had previously been generated during candidate vaccine virus development, and we tested these antisera’s capacity to neutralize both egg- and cell-derived NC14 and KAN17 LAIV strains. Most cell-derived H3N2 strains from 2016 to 2020 were incapable of haemagglutination [36,37], so we instead adapted the microneutralization assay described by Jorqeura *et al*. [30]. According to WHO guidelines, the serum antibody response to a test strain must be within fourfold of the serum antibody response to a reference virus to be considered antigenically matched [36,37].

**Fig. 4.**
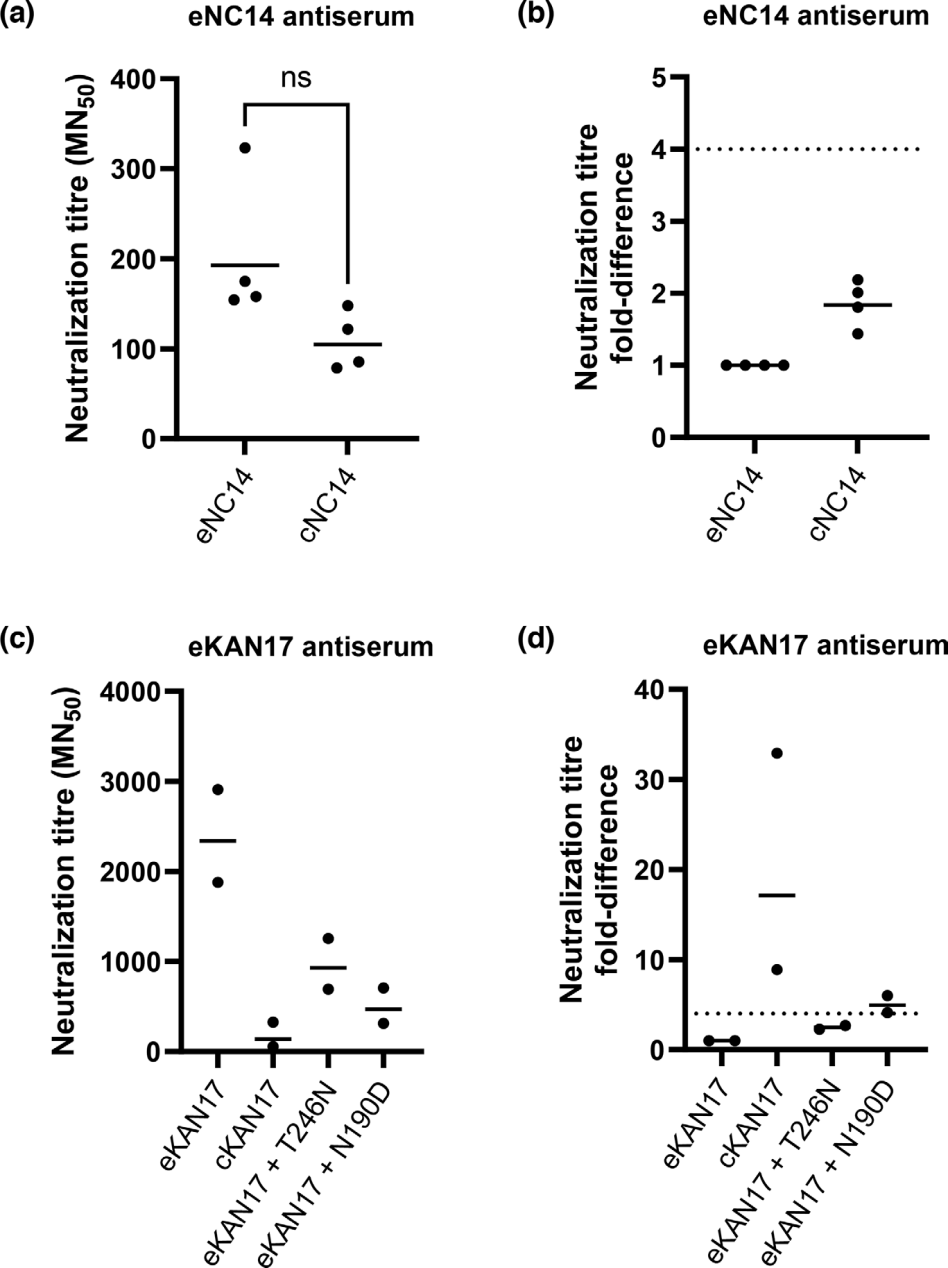
Neutralization titres of egg- and cell-adapted LAIV strains using antisera raised against the egg strain. Ferret antisera were raised against egg-adapted LAIV strains and used in microneutralisation assays against a panel of egg- and cell-adapted LAIV variants of NC14 and KAN17. (a) and (c) The dilution of neat antiserum required to reduce the numbers of fluorescent foci by 50% (MN50) was calculated for each ferret. Points indicate values for individual ferrets (mean of three assay repeats for NC14, two assay repeats for KAN17); bars indicate the geometric means. Statistical analysis was not performed for KAN17 as there were insufficient samples to assess the distribution. For NC14, p values were calculated by a two-tailed Wilcoxon matched-pairs signed rank test: ns p > 0.05. (b) and (d) The MN50 values from (a) and (c) expressed as a ratio to the MN50 titre for the homologous, egg-adapted strain. Dashed line indicates the four-fold threshold, below which strains are classed as antigenically similar.

Sera raised against eNC14 neutralized cNC14 1.8× less effectively than eNC14 (Fig. 4a, b, *P*=0.125) which was both statistically insignificant and within the fourfold range that indicates antigenic similarity. The loss of the N158 glycan due to egg adaptation therefore did not cause an antigenic mismatch.

Sera raised against eKAN17 neutralized cKAN17 17.1× less effectively than eKAN17 (Fig. 4c, d). However, it was not clear whether this mismatch was caused by the loss of glycosylation at N246, as eKAN17 contained a second egg adaptation mutation in antigenic site B, D190N (Table 2). To isolate the impact of the N246T egg adaptation on antigenicity, we generated intermediate eKAN17 variants which each reverted one of the egg adaptations to the cell-derived sequence: eKAN17+T246N and eKAN17+N190D. Sera raised against eKAN17 neutralized eKAN17+T246N 2.5× less effectively than eKAN17, indicating that the strains were antigenically similar. Conversely, eKAN17+N190D was neutralized 5.0× less effectively than eKAN17, indicating that the strains were antigenically distinct. The loss of the N246 glycan alone therefore did not cause an antigenic mismatch between eKAN17 and cKAN17.

### NC14 was distinct from circulating clades in 2016–18, but KAN17 was representative of clades in 2019–20

To this point, we had only compared egg- and cell-adapted variants of the same strain. However, cell-derived counterparts of vaccine strains may not represent circulating WT strains from the seasons when these vaccines were used clinically and so may offer only limited information on the impact of glycan shield changes in a real-world context.

To assess the relevance of the glycomic and antigenic data from cNC14 and cKAN17 to circulating strains, we assessed which H3N2 clades were prevalent during the appropriate influenza seasons and whether each LAIV represented these clades. We downloaded 2,406 HA protein sequences from the GISAID EpiFlu^™^ Database, covering the 2016–18 influenza seasons (H3N2 LAIV strain: eNC14) and the 2019–20 influenza season (H3N2 LAIV strain: eKAN17). We focused on sequences from the UK, as this represented the largest population using the Fluenz^™^ Tetra LAIV. To sort circulating strains into clades, we compared the HA sequence with the list of clade-defining amino acid residues defined by the WHO [36,37].

**Fig. 5.**
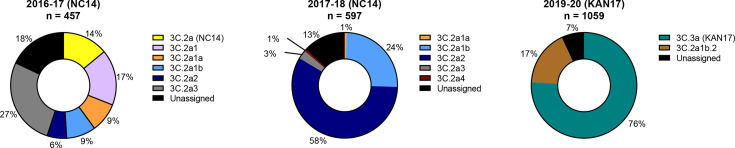
H3N2 clades circulating in the UK when NC14 and KAN17 were components of the LAIV. Complete HA amino acid sequences of strains isolated in the UK in the indicated influenza seasons were downloaded from the GISAID EpiFluTM database and sorted into clades by comparing the sequences with the list of clade-defining mutations described by the WHO for strains from 2016–2018 (McAuley et al., 2018) and for strains from 2019–2020 (McAuley et al., 2020). Vaccine strains are indicated in brackets, pie charts indicate the proportion of strains that were sorted into each clade, and “n” indicates the number of sequences analysed for each chart.

The 2016–17 H3N2 season was highly varied, with six clades each comprising at least 5% of the total sequences (Fig. 5). The largest clade was 3C.2a3, comprising 28% of the sequences, whereas NC14’s own clade, 3C.2a, comprised 14% of the sequences. In 2017–18, 3C.2a strains were no longer detected, and instead, the 3C.2a2 clade dominated, comprising 60% of the sequences. Therefore, at the clade level, NC14 did not represent the majority of circulating strains during its two seasons as a vaccine strain, and so antigenic comparisons between eNC14 and cNC14 did not fully represent the relationship between eNC14 and circulating strains.

In 2019–20, the 3C.3a clade was predominant amongst H3N2 strains, comprising 76% of the sequences and KAN17 itself. KAN17 therefore represented circulating strains at the clade level, and the antigenic comparison of eKAN17 and cKAN17 was likely to represent the antigenic similarity between eKAN17 and circulating strains in 2019–20.

### Despite the loss of glycosylation at N158, eNC14 was antigenically similar to most circulating clades from 2016 to 2018

As NC14 did not represent most circulating clades in 2016–18, assessing the impact of the loss of the N158 glycan in the eNC14 vaccine strain required a comparison of its antigenicity with a panel of 11 cell-adapted viruses encompassing all six major clades (see Table 1 and the ‘Methods’ section for full strain details). Most of this panel possessed the N158 site, which was only absent in eNC14, HK5738 and BRIS190. All other glycan sites present in eNC14 were present in all strains, except for GRE17 which lacked the sites at N122 and N133. To isolate the impact of changes to the HA and NA proteins from variation elsewhere in the genome, each virus was rescued with HA and NA genes from the cell-adapted strain and the remaining genes from the LAIV cold-adapted, temperature-sensitive, attenuated A/Ann Arbor/6/1960 master donor virus [28]. Antigenicity was assessed by microneutralization assay using sera raised against eNC14 as described above.

**Fig. 6.**
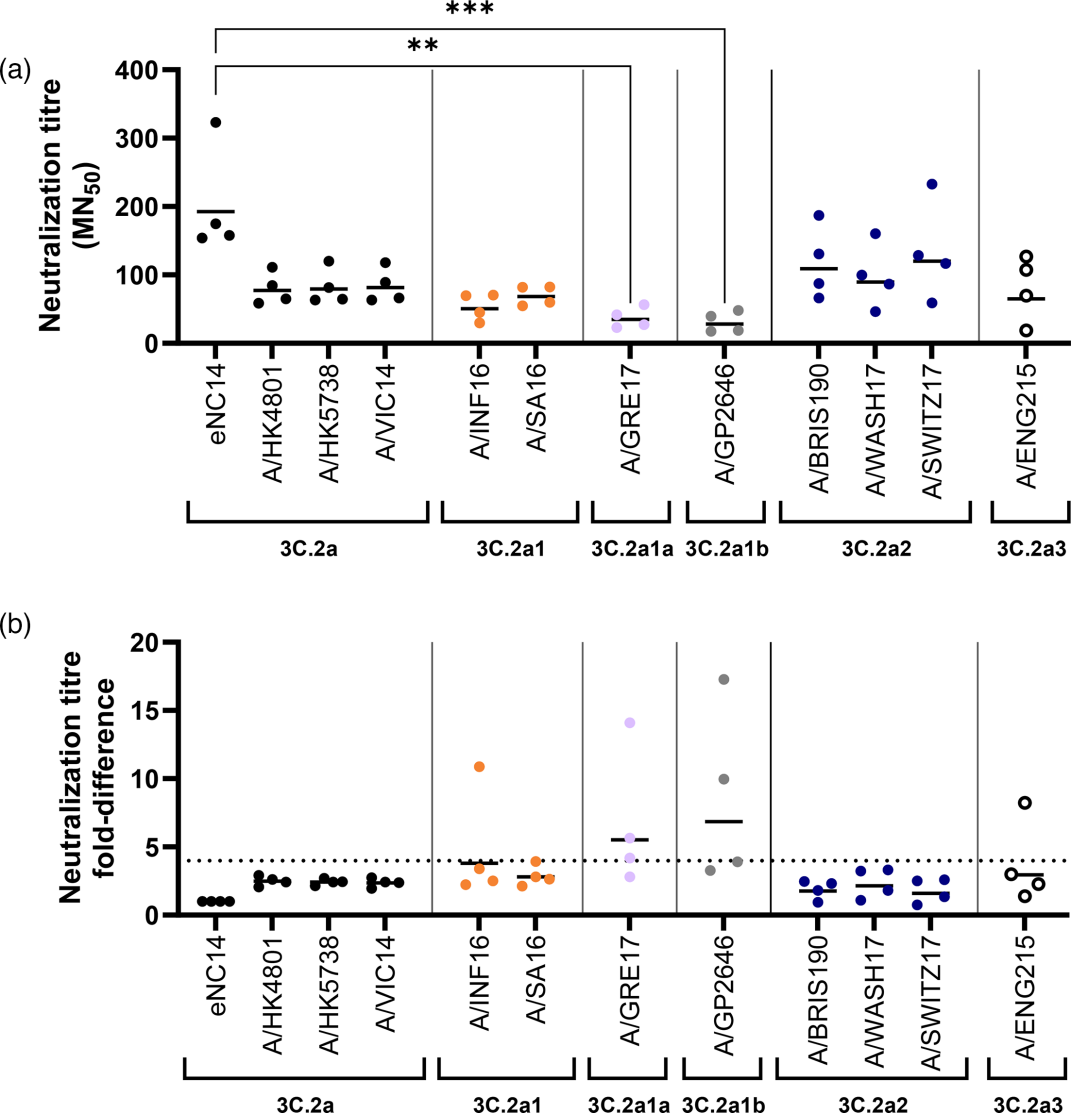
eNC14 was antigenically similar to most circulating clades from 2016 to 2018. Ferret antisera raised against eNC14 was used to perform microneutralisation assays against a panel of viruses representing the major clades circulating in the UK from 2016–18. (a) The antisera dilution required to reduce infectivity of MDCK-SIAT cells by 50% (MN50) was calculated. Points represent individual ferrets (mean of two assay repeats for 3C.2a2 strains and three assay repeats for all other strains), bars indicate the geometric mean. p values for all samples were calculated using a Friedman test followed by a Dunn’s multiple comparisons test: ** p < 0.01, *** p < 0.001, not shown p > 0.05. (b) MN50 values from (a) expressed as a fold-difference relative to the homologous titre. Dashed line indicates a four fold difference.

Strains from the 3C.2a, 3C.2a1, 3C.2a2 and 3C.3a3 clades all had geometric mean neutralization titres that were less than fourfold removed from the titre of the homologous eNC14 strain (Fig. 6). This indicated that most circulating clades were antigenically similar to eNC14 (comprising 78% of the sequences that could be assigned clades in 2016–17 and 70% in 2018–19). Sera raised against eNC14 neutralized the 3C.2a1a strain, A/GRE17, 5.5× less effectively than eNC14 (*P*=0.0021) and the 3C.2a1b strain, A/GP2646, 6.9× less effectively than eNC14 (*P*=0.0010). This indicated that a minority of circulating clades were antigenically distinct from eNC14 (22% of the sequences that could be assigned clades in 2016–17 and 30% in 2018–19). The loss of glycosylation at N158 therefore did not prevent antisera raised against eNC14 from neutralizing most circulating clades.

## Discussion

Here, we assessed the impact of egg adaptation-induced changes to the glycan shield on the antibody responses induced by the H3N2 LAIVs, NC14 and KAN17. We used nLC-MS to confirm that amino acid changes at glycan sites were reflected by occupancy changes in the glycan shields. However, despite their proximity to antigenic site B, the loss of glycan N158 in eNC14 and glycan N246 in eKAN17 did not cause an antigenic mismatch with circulating strains.

Our nLC-MS analysis expands the literature of H3N2 glycan profile analyses to include more recent strains. Our observation that glycans N22, N38, N63 and N483 were predominantly complex isoforms whereas N165, N285 and N246 were predominantly oligomannose isoforms (Fig. 1) was also seen in several previous studies and so suggests that the glycan isoform at these sites is highly conserved [10,38,41]. The varied isoforms seen at N126 between cNC17 and eNC17 agree with previous analyses, suggesting that this glycan is variable, with complex [10], oligomannose [10] and mixed [38,39] isoforms all being reported. Conversely, we observed that glycans N122 and N133 were predominantly complex isoforms in NC14 and KAN17 (Fig. 1), whereas older strains were predominantly oligomannose [10,39]. Together with our observation that N158 was predominantly complex in cNC14, these data indicate that glycan composition on the H3N2 HA protein is context-dependent and so does not support a simple model where increasing the number of glycan sites directly results in a higher proportion of oligomannose isoforms.

A previous study by Zost *et al*. suggested that egg-adapted H3N2 vaccines in 2016–17 had reduced VE due to an antigenic mismatch caused by the loss of the N158 glycan [24]. However, our data and subsequent studies suggest that several other factors must also be considered to explain VE. First, data from this study (Fig. 6), as well as characterization by the WHO, showed that strains such as eNC14 that were antigenically similar to the named vaccine strain, egg-derived A/Hong Kong/4801/2014, were also antigenically similar to their cell-derived counterparts and most other 3C.2a strains [37,42]. The discrepancy with Zost *et al*. may result from differences in vaccine dose and platform: Zost *et al*. [24] inoculated ferrets with 5.3 log10 PFU ml^−1^ of vaccine viruses on an A/Puerto Rico/8/1934 backbone, whereas we used a dose of ~7 log10 FFU ml^−1^ on a cold-adapted, temperature-sensitive, attenuated A/Ann Arbor/6/1960 backbone [28]. Second, A/Hong Kong/4801/2014-like strains were used in the vaccine from 2016 to 2018, but VE was lower for all vaccine platforms in 2017–18 [LAIV: −75.5%, 95% CI (−289.6 to 21%); IIV: −14.7%, 95% CI (−72.7 to 23.8%)] than in 2016–17 [LAIV: 57.0%, 95% CI (7.7–80.0%); IIV: 36.6%, 95% CI (10.4–55.1%) [43,44]]. This difference occurred despite egg-derived vaccine strains being antigenically similar to most circulating clades in both seasons (Fig. 6), and despite the presence of the N158 glycan site in circulating strains throughout [37,42]. Third, an analysis of the US 2017–18 influenza season identified only a modest difference between the VE of cell-derived and egg-derived vaccines, and no difference between cell-derived and high-dose egg-derived vaccines [45]. This agrees with Gouma *et al*. who found no significant difference in antibody response between cell-derived and egg-derived vaccines at standard doses [46]. Taken together, the available data suggest that the loss of glycosylation at N158 on vaccine strains did not impact their antigenic similarity to circulating strains from 2016 to 2018, nor did antigenic changes significantly contribute to the varied VE seen from 2016 to 2018.

Unlike NC14, only the LAIV of eKAN17 lost a glycan as a result of egg adaptation: eKAN17 LAIV contained the egg-adaptive mutations D190N and N246T, whereas eKAN17 IIV contained G186V, D190N and S219Y [47]. Despite these differences, our data and a study by Gouma *et al*. indicate that both vaccine platforms were antigenically dissimilar to circulating strains (Fig. 4 [47]). These findings correlate with both platforms exhibiting low to moderate VE in the UK in 2019–20 [LAIV: 39%, 95% CI (−21 to 69%); IIV: 31%, 95% CI (−8 to 56%) [48]]. In both cases, test viruses containing the egg-adaptive D190N were antigenically matched to the vaccine strain, but test viruses containing the circulating 190D were not (Fig. 4 [47]). D190N was therefore the primary driver of antigenic mismatch between eKAN17 vaccine strains and circulating strains, though the mismatch was greater in the presence of the N246T egg adaptation in LAIV (Fig. 4) and the G186V egg adaptation in IIV [47]. This finding is supported by the fact that residue 190 is part of the receptor-binding site and antigenic site B [33], and changes to this residue have previously been linked with reduced cross-reactivity in H3N2 vaccines [22]. For LAIV, as the N246T mutation alone did not cause an antigenic mismatch, we conclude that there was not a significant antibody population targeting an epitope shielded by the N246 glycan. Therefore, the loss of the N246 glycan did not substantially contribute to the antigenic profile of eKAN17, nor is it likely to have caused a reduction in VE.

There are some caveats to consider when contextualizing our data with clinical VE estimates. First, we analysed recombinant proteins that were generated in HEK-293T cells, but glycosylation patterns vary with cell type [49]. As LAIV viruses are manufactured in eggs and then replicate further in patients’ respiratory tracts, antibody responses are likely raised against HA proteins from a diverse array of cell types. This diversity may not be fully captured by an HEK-293T system. Second, immune responses in clinical influenza cases are influenced by previous exposure to influenza virus antigens, and any impact of this antigenic history will not be represented in the immunologically naïve ferrets used in our study [50]. However, as discussed above, our assessment of eNC14 antigenicity gave similar conclusions to those seen in a study using human sera samples [46] and an analysis of human patient data [45]. Third, the number of ferret samples in this study was low (four for eNC14 and two for eKAN17), which reduced the statistical power available for assessing antigenicity. This limitation was due to ethical considerations, as these antisera were originally raised during candidate vaccine virus development, and the minimum number of samples was selected that would be suitable for WHO antigenicity testing. Fourth, the HA antibody response is only one component of immune protection, and clinical VE for LAIV will likely also be influenced by a vaccine’s capacity to induce a neuraminidase antibody response [51], as well as cell-mediated responses [52]. The microneutralization assays used in this study only assess HA antigenicity, and so, further research is necessary to determine the contributions of the different components of the immune response to clinical LAIV VE.

Our finding that some egg-adaptive mutations occur without necessarily impacting antigenicity suggests two applications that could generate higher-yield vaccine strains. First, whilst the N158 glycan site has since been lost in circulating strains, the N246 site is still present [53]. Therefore, selecting vaccine strains which acquired N246T during egg isolation, or introducing N246T via site-directed mutagenesis, could produce strains with higher yields in eggs whilst remaining antigenically similar to circulating strains. In our study, the antigenic impact of N246T on eKAN17 could have been masked if the D190N egg adaptation was immunodominant, but a previous study found that a 2011 candidate H3N2 vaccine strain containing the similar N246K egg adaptation was antigenically matched to the parent strain in the absence of D190N [54]. Second, the antigenic similarity between eNC14 and cNC14 indicates that the egg-adaptive mutations H183L and D225G could also be rationally incorporated into new vaccine strains, although D225G has been previously identified as impacting the antigenicity of A/Singapore/INFIMH-16-0019/2016 which suggests that its impact is context-dependent [55]. Taken together, introducing egg adaptations at residues 183, 225 or 246 is a promising strategy to improve H3N2 vaccine yields, though the rapid evolution of H3N2 HA proteins necessitates testing this in contemporary strains to ensure that the effect persists in new sequence contexts.

In summary, we have used MS and microneutralization assays to show that egg adaptation did cause the loss of a glycan in NC14 and KAN17 LAIV strains. However, in neither case was the glycosylation change alone sufficient to cause an antigenic mismatch between the vaccine viruses and circulating strains.

## Supplementary material

10.1099/jgv.0.002122 Uncited Supplementary Material 1.

## Abbreviations

EMEM: Eagle’s Minimum Essential Medium
HA: haemagglutinin
HAI: haemagglutination inhibition
IIV: inactivated influenza vaccine
LAIV: live attenuated influenza vaccine
MDCK: Madin–Darby canine kidney
nLC-MS: nano-liquid chromatography-MS
RT: room temperature
VE: vaccine effectiveness
WHO: World Health Organization
