# Supplementary material for: Glycan site loss in two egg-adapted live attenuated influenza vaccine strains does not cause antigenic mismatches
Source: J Gen Virol. 2025 Jul 3;106(7):002122. doi: 10.1099/jgv.0.002122 (PMC12231097; doi:10.1099/jgv.0.002122)
Supplement: Uncited Supplementary Material 1. [file jgv-106-02122-s001.pdf]

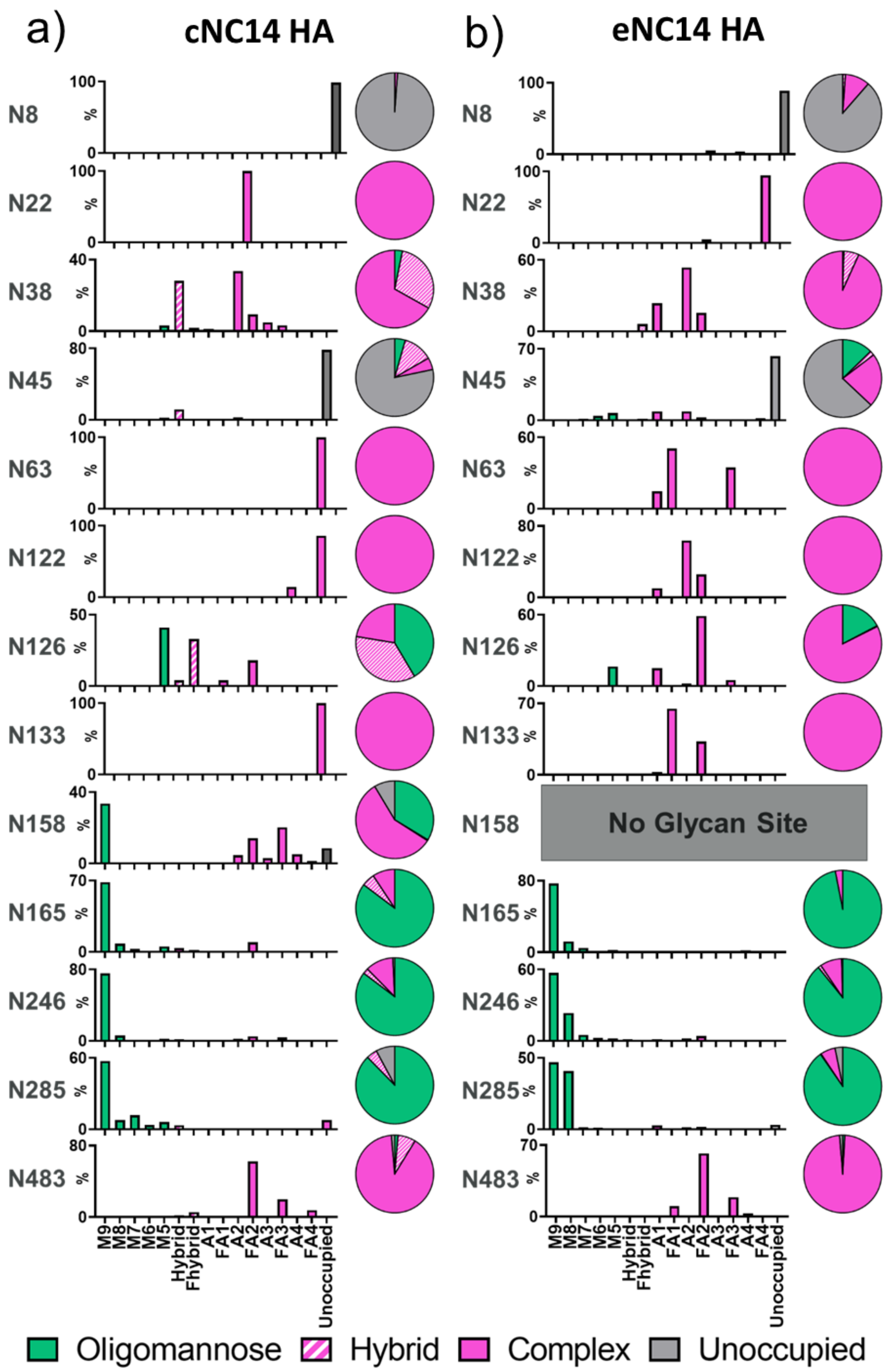

Supplementary Figure 1. Comparative site-specific N-linked glycosylation analysis of (a) eNC14 and (b) cNC14 HAs. The graphs summarize the quantitative mass spectrometric analysis of the glycan population present at individual N-linked glycan sites with oligomannose-type glycan series (M9 to M5; Man9GlcNAc2 to Man5GlcNAc2) (green), afucosylated and fucosylated hybrid glycans (Hybrid & F Hybrid) (dashed pink), and complex glycans grouped according to the number of antennae and presence of core fucosylation (A1 to FA4) (pink). Left to right; least processed to most processed. The pie charts summarise the quantification of these glycans. Unoccupancy of an N-linked glycan site is shown grey. N.D denotes no data. The pie charts summarize the quantification of these glycans.

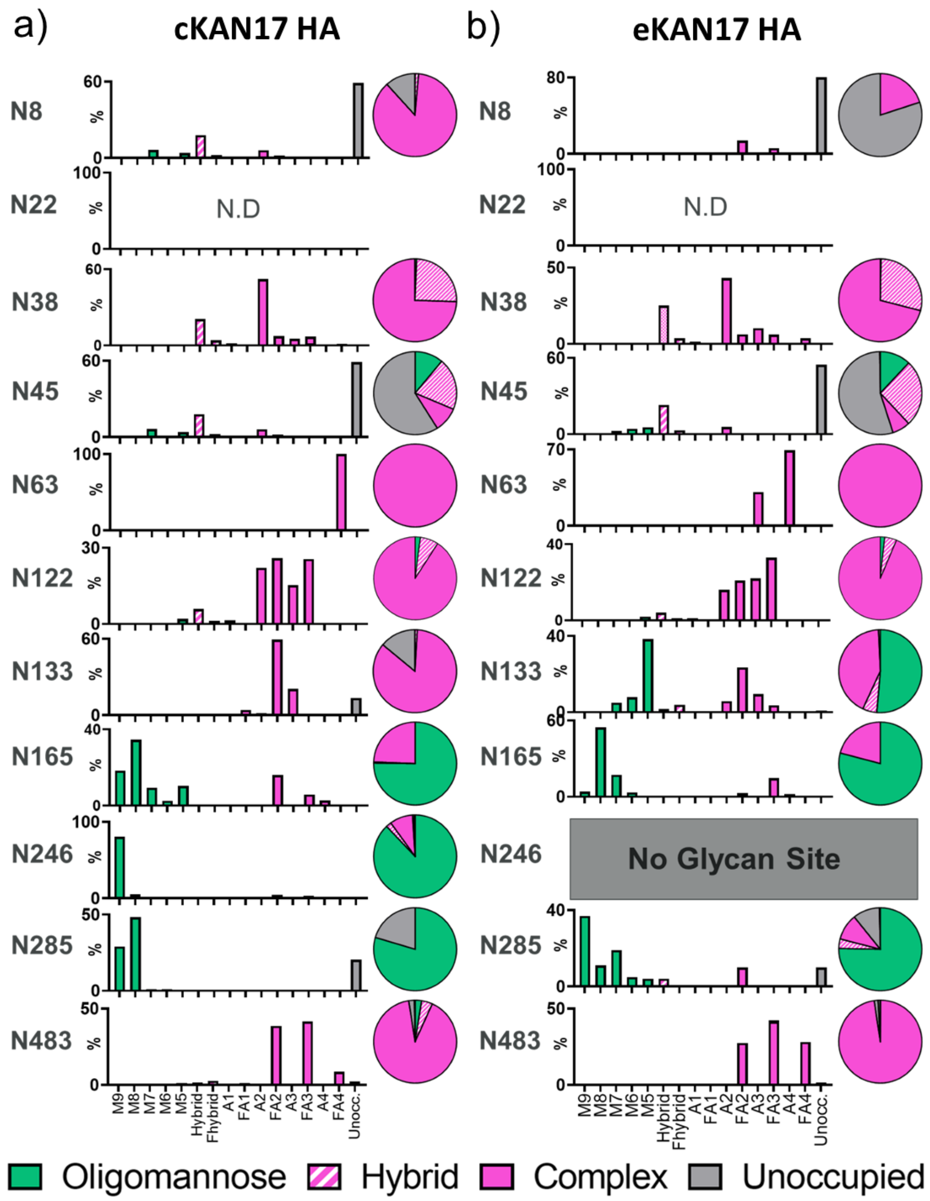

Supplementary Figure 2. Comparative site-specific N-linked glycosylation analysis of (A) eKAN17 and (B) eKAN17 HAs. The graphs summarize the quantitative mass spectrometric analysis of the glycan population present at individual N-linked glycan sites with oligomannose-type glycan series (M9 to M5; Man9GlcNAc2 to Man5GlcNAc2) (green), afucosylated and fucosylated hybrid glycans (Hybrid & F Hybrid) (dashed pink), and complex glycans grouped according to the number of antennae and presence of core fucosylation (A1 to FA4) (pink). Left to right; least processed to most processed. The pie charts summarise the quantification of these glycans. Unoccupancy of an N-linked glycan site is shown grey. N.D denotes no data. The pie charts summarize the quantification of these glycans.

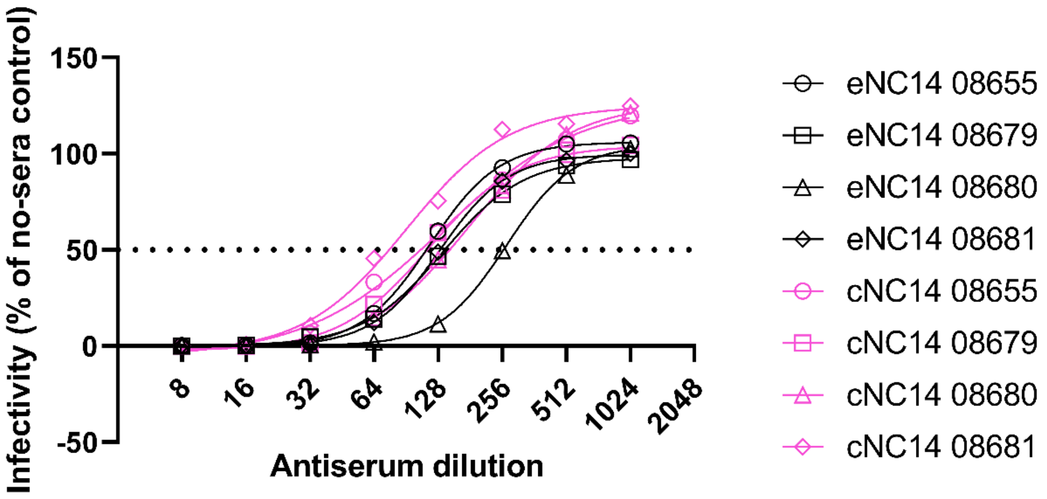

Supplementary Figure 3: Example MN_{50} curves of NC14 variants. Antisera from four ferrets (08655, 08679, 08681, 08681) were raised against eNC14 and used to perform microneutralisation assays against eNC14 and cNC14. Points indicate number of infected cells relative to a no-serum control. Lines indicate four-parameter, least squares curve fit for each group of samples. MN_{50} values were calculated by interpolating the 50% value for each curve which is indicated by the dashed line.

Supplementary Table 1: GISAID acknowledgements for virus strains used in this study.

We gratefully acknowledge the authors, originating and submitting laboratories of the sequences from GISAID's EpiFlu™ Database on which this research is based. The list is detailed below. All submitters of data may be contacted directly via www.gisaid.org.

Segment ID	Segment	Country	Collection date	Isolate-ID	Isolate name	Originating Lab	Submitting Lab	Authors
EPI1323469	NA	Switzerland	2018-Jan-01	EPI_ISL_331924	A/Switzerland/8060/2017	National Institute for Medical Research	Centers for Disease Control and Prevention	
EPI1323470	HA	Switzerland	2018-Jan-01	EPI_ISL_331924	A/Switzerland/8060/2017	National Institute for Medical Research	Centers for Disease Control and Prevention	
EPI1261066	NA	United States	2017-Dec-14	EPI_ISL_316455	A/Kansas/14/2017	Kansas Department of Health and Environment	Centers for Disease Control and Prevention	
EPI1261067	HA	United States	2017-Dec-14	EPI_ISL_316455	A/Kansas/14/2017	Kansas Department of Health and Environment	Centers for Disease Control and Prevention	
EPI1153061	NA	Australia	2017-Oct-18	EPI_ISL_293803	A/Brisbane/190/2017	Queensland Health Forensic and Scientific Services	WHO Collaborating Centre for Reference and Research on Influenza	Deng,Y-M.; Iannello,P.; Lau,H.; Kaye,M.; Todd,A.; Komadina,N.
EPI1153062	HA	Australia	2017-Oct-18	EPI_ISL_293803	A/Brisbane/190/2017	Queensland Health Forensic and Scientific Services	WHO Collaborating Centre for Reference and Research on Influenza	Deng,Y-M.; Iannello,P.; Lau,H.; Kaye,M.; Todd,A.; Komadina,N.
EPI1146344	NA	United States	2017-Dec-14	EPI_ISL_292575	A/Kansas/14/2017	Kansas Department of Health and Environment	Centers for Disease Control and Prevention	
EPI1146345	HA	United States	2017-Dec-14	EPI_ISL_292575	A/Kansas/14/2017	Kansas Department of Health and Environment	Centers for Disease Control and Prevention	
EPI1106226	NA	Singapore	2016-Jun-14	EPI_ISL_285897	A/Singapore/INFIMH-16-0019/2016	WHO Collaborating Centre for Reference and	Centers for Disease Control and Prevention	

Segment ID	Segment	Country	Collection date	Isolate-ID	Isolate name	Originating Lab	Submitting Lab	Authors
						Research on Influenza		
EPI1106227	HA	Singapore	2016-Jun-14	EPI_ISL_285897	A/Singapore/INFIMH-16-0019/2016	WHO Collaborating Centre for Reference and Research on Influenza	Centers for Disease Control and Prevention	
EPI1047573	NA	United States	2017-Jan-25	EPI_ISL_275706	A/Washington/16/2017	Washington State Public Health Laboratory	Centers for Disease Control and Prevention	
EPI1047574	HA	United States	2017-Jan-25	EPI_ISL_275706	A/Washington/16/2017	Washington State Public Health Laboratory	Centers for Disease Control and Prevention	
EPI967219	HA	Greece	2017-Jan-02	EPI_ISL_257379	A/Greece/4/2017	Aristotelian University of Thessaloniki	Crick Worldwide Influenza Centre	
EPI967220	NA	Greece	2017-Jan-02	EPI_ISL_257379	A/Greece/4/2017	Aristotelian University of Thessaloniki	Crick Worldwide Influenza Centre	
EPI914425	NA	United Kingdom	2016-Dec-19	EPI_ISL_248548	A/England/70180215/2016	UK Health Security Agency - Colindale	UK Health Security Agency - Colindale	Galiano,M.
EPI914426	HA	United Kingdom	2016-Dec-19	EPI_ISL_248548	A/England/70180215/2016	UK Health Security Agency - Colindale	UK Health Security Agency - Colindale	Galiano,M.
EPI874087	NA	Singapore	2016-Dec-08	EPI_ISL_240797	A/Singapore/GP2646/2016		Ministry of Health, Singapore	
EPI874088	HA	Singapore	2016-Dec-08	EPI_ISL_240797	A/Singapore/GP2646/2016		Ministry of Health, Singapore	
EPI868819	HA	Norway	2016-Nov-07	EPI_ISL_239788	A/Norway/4465/2016	WHO National Influenza Centre	Crick Worldwide Influenza Centre	
EPI868820	NA	Norway	2016-Nov-07	EPI_ISL_239788	A/Norway/4465/2016	WHO National Influenza Centre	Crick Worldwide Influenza Centre	
EPI653200	NA	Hong Kong (SAR)	2014-Feb-26	EPI_ISL_198222	A/Hong Kong/4801/2014	Crick Worldwide Influenza Centre	Centers for Disease Control and Prevention	
EPI653201	HA	Hong Kong (SAR)	2014-Feb-26	EPI_ISL_198222	A/Hong Kong/4801/2014	Crick Worldwide Influenza Centre	Centers for Disease Control and Prevention	

Segment ID	Segment	Country	Collection date	Isolate-ID	Isolate name	Originating Lab	Submitting Lab	Authors
EPI551848	NA	Australia	2014-Sep-19	EPI_ISL_168962	A/VICTORIA/5060/2014	Victorian Infectious Diseases Reference Laboratory	WHO Collaborating Centre for Reference and Research on Influenza	Deng,Y-M.; Iannello,P.; Spirason,N.; Jelley,L.; Lau,H.; Komadina,N.
EPI551849	HA	Australia	2014-Sep-19	EPI_ISL_168962	A/VICTORIA/5060/2014	Victorian Infectious Diseases Reference Laboratory	WHO Collaborating Centre for Reference and Research on Influenza	Deng,Y-M.; Iannello,P.; Spirason,N.; Jelley,L.; Lau,H.; Komadina,N.
EPI551568	NA	New Caledonia	2014-Aug-13	EPI_ISL_168901	A/NEW CALEDONIA/71/2014	Institut Pasteur New Caledonia	WHO Collaborating Centre for Reference and Research on Influenza	Deng,Y-M.; Iannello,P.; Spirason,N.; Jelley,L.; Lau,H.; Komadina,N.
EPI551570	HA	New Caledonia	2014-Aug-13	EPI_ISL_168901	A/NEW CALEDONIA/71/2014	Institut Pasteur New Caledonia	WHO Collaborating Centre for Reference and Research on Influenza	Deng,Y-M.; Iannello,P.; Spirason,N.; Jelley,L.; Lau,H.; Komadina,N.
EPI1322581	HA	United Kingdom	2017-Jan-01	EPI_ISL_331787	NIB-112 (A/Switzerland/8060/2017)	National Institute for Biological Standards and Control (NIBSC)	WHO Collaborating Centre for Reference and Research on Influenza	Deng,Y-M; Iannello,P; Lau,H; Kaye,M; Todd,A; Spirason,N; Komadina,N
EPI1126236	HA	United Kingdom	2017-Jan-01	EPI_ISL_288986	NIB-104 (A/Singapore/INFIMH-16-0019/2016)	National Institute for Biological Standards and Control (NIBSC)	WHO Collaborating Centre for Reference and Research on Influenza	Deng,Y-M.; Iannello,P.; Lau,H.; Kaye,M.; Todd,A.; Komadina,N.
EPI1126234	HA	United Kingdom	2017-Jan-01	EPI_ISL_288985	NIB-103 (A/Norway/3806/2016)	National Institute for Biological	WHO Collaborating Centre for	Deng,Y-M.; Iannello,P.; Lau,H.; Kaye,M.; Todd,A.; Komadina,N.

Segment ID	Segment	Country	Collection date	Isolate-ID	Isolate name	Originating Lab	Submitting Lab	Authors
						Standards and Control (NIBSC)	Reference and Research on Influenza	
EPI1086288	HA	United Kingdom	2017-Oct-24	EPI_ISL_282899	A/Singapore/INFIMH-16-0019/2016 (17/196)		National Institute for Biological Standards and Control (NIBSC)	Nicolson, Carolyn
EPI1531736	HA	United Kingdom	2018-Feb-27	EPI_ISL_373415	A/England/7400/2018			Gruner,W.E.; Fries,A.C.; Garrett,C.M.; Powell,M.L.; Minnich,D.E.; Couch,M.R.; Hanson,J.F.; DeMarcus,L.S.; Crum,M.E.; Bogue,A.L.; Federinko,S.P.; Macias,E.A.; West,C.A.; Poel,J.R.
EPI1531728	HA	United Kingdom	2018-Feb-21	EPI_ISL_373414	A/England/7399/2018			Gruner,W.E.; Fries,A.C.; Garrett,C.M.; Powell,M.L.; Minnich,D.E.; Couch,M.R.; Hanson,J.F.; DeMarcus,L.S.; Crum,M.E.; Bogue,A.L.; Federinko,S.P.; Macias,E.A.; West,C.A.; Poel,J.R.
EPI1531704	HA	United Kingdom	2018-Mar-12	EPI_ISL_373411	A/England/7335/2018			Gruner,W.E.; Fries,A.C.; Garrett,C.M.; Powell,M.L.; Minnich,D.E.; Couch,M.R.; Hanson,J.F.; DeMarcus,L.S.; Crum,M.E.; Bogue,A.L.; Federinko,S.P.; Macias,E.A.; West,C.A.; Poel,J.R.
EPI1310133	HA	United Kingdom	2018-Apr-16	EPI_ISL_329783	A/Northern Ireland/14060/2018	Regional Virus Laboratory, Microbiology Department, Royal Victoria Hospital	Crick Worldwide Influenza Centre	
EPI1310130	HA	United Kingdom	2018-Mar-20	EPI_ISL_329781	A/Northern Ireland/12797/2018	Regional Virus Laboratory, Microbiology Department, Royal Victoria Hospital	Crick Worldwide Influenza Centre	

Segment ID	Segment	Country	Collection date	Isolate-ID	Isolate name	Originating Lab	Submitting Lab	Authors
EPI1302963	HA	United Kingdom	2017-Nov-07	EPI_ISL_327918	A/Mold/7024/2017	Public Health Wales Microbiology Cardiff	Public Health Wales Microbiology Cardiff	
EPI1302967	HA	United Kingdom	2017-Nov-07	EPI_ISL_327918	A/Mold/7024/2017	Public Health Wales Microbiology Cardiff	Public Health Wales Microbiology Cardiff	
EPI1302867	HA	United Kingdom	2017-Oct-16	EPI_ISL_327845	A/Buckley/4801/2017	Public Health Wales Microbiology Cardiff	Public Health Wales Microbiology Cardiff	
EPI1302851	HA	United Kingdom	2018-Mar-18	EPI_ISL_327842	A/St Clears/2727/2018	Public Health Wales Microbiology Cardiff	Public Health Wales Microbiology Cardiff	
EPI1302852	HA	United Kingdom	2018-Mar-18	EPI_ISL_327842	A/St Clears/2727/2018	Public Health Wales Microbiology Cardiff	Public Health Wales Microbiology Cardiff	
EPI1302348	HA	United Kingdom	2018-Feb-13	EPI_ISL_327776	A/Pontyclun/4501/2018	Public Health Wales Microbiology Cardiff	Public Health Wales Microbiology Cardiff	
EPI1302247	HA	United Kingdom	2018-Feb-04	EPI_ISL_327753	A/Llanelli/5367/2018	Public Health Wales Microbiology Cardiff	Public Health Wales Microbiology Cardiff	
EPI1302134	HA	United Kingdom	2018-Feb-02	EPI_ISL_327732	A/Cardiff/1973/2018	Public Health Wales Microbiology Cardiff	Public Health Wales Microbiology Cardiff	
EPI1302055	HA	United Kingdom	2018-Feb-02	EPI_ISL_327722	A/Abertillery/1971/2018	Public Health Wales Microbiology Cardiff	Public Health Wales Microbiology Cardiff	
EPI1301944	HA	United Kingdom	2018-Feb-02	EPI_ISL_327709	A/Barry/1912/2018	Public Health Wales Microbiology Cardiff	Public Health Wales	

Segment ID	Segment	Country	Collection date	Isolate-ID	Isolate name	Originating Lab	Submitting Lab	Authors
							Microbiology Cardiff	
EPI1301834	HA	United Kingdom	2018-Mar-03	EPI_ISL_327694	A/Cardiff/9945/2018	Public Health Wales Microbiology Cardiff	Public Health Wales Microbiology Cardiff	
EPI1301800	HA	United Kingdom	2018-Feb-27	EPI_ISL_327690	A/Cardiff/9849/2018	Public Health Wales Microbiology Cardiff	Public Health Wales Microbiology Cardiff	
EPI1301796	HA	United Kingdom	2018-Feb-27	EPI_ISL_327689	A/Cardiff/9779/2018	Public Health Wales Microbiology Cardiff	Public Health Wales Microbiology Cardiff	
EPI1279369	HA	United Kingdom	2018-Feb-18	EPI_ISL_322180	A/Caerphilly/1813/2018	Public Health Wales Microbiology Cardiff	Public Health Wales Microbiology Cardiff	
EPI1275673	HA	United Kingdom	2018-Feb-27	EPI_ISL_321587	A/Aberdare/1604/2018	Public Health Wales Microbiology Cardiff	Public Health Wales Microbiology Cardiff	
EPI1275650	HA	United Kingdom	2018-Mar-12	EPI_ISL_321583	A/Swansea/8616/2018	Public Health Wales Microbiology Cardiff	Public Health Wales Microbiology Cardiff	
EPI1275634	HA	United Kingdom	2018-May-07	EPI_ISL_321581	A/Swansea/0699/2018	Public Health Wales Microbiology Cardiff	Public Health Wales Microbiology Cardiff	
EPI1275625	HA	United Kingdom	2018-Apr-05	EPI_ISL_321580	A/Aberystwyth/6333/2018	Public Health Wales Microbiology Cardiff	Public Health Wales Microbiology Cardiff	
EPI1275609	HA	United Kingdom	2018-Mar-16	EPI_ISL_321577	A/Lampeter/6039/2018	Public Health Wales Microbiology Cardiff	Public Health Wales Microbiology Cardiff	

Segment ID	Segment	Country	Collection date	Isolate-ID	Isolate name	Originating Lab	Submitting Lab	Authors
EPI1275599	HA	United Kingdom	2018-Mar-05	EPI_ISL_321575	A/Newquay/5831/2018	Public Health Wales Microbiology Cardiff	Public Health Wales Microbiology Cardiff	
EPI1275591	HA	United Kingdom	2018-Mar-05	EPI_ISL_321574	A/Abertillery/5073/2018	Public Health Wales Microbiology Cardiff	Public Health Wales Microbiology Cardiff	
EPI1275583	HA	United Kingdom	2018-Mar-05	EPI_ISL_321573	A/Aberystwyth/2999/2018	Public Health Wales Microbiology Cardiff	Public Health Wales Microbiology Cardiff	
EPI1275576	HA	United Kingdom	2018-Feb-16	EPI_ISL_321572	A/Rhyl/9918/2018	Public Health Wales Microbiology Cardiff	Public Health Wales Microbiology Cardiff	
EPI1275568	HA	United Kingdom	2018-Feb-10	EPI_ISL_321571	A/Wrexham/9243/2018	Public Health Wales Microbiology Cardiff	Public Health Wales Microbiology Cardiff	
EPI1275559	HA	United Kingdom	2018-Apr-08	EPI_ISL_321570	A/Wrexham/9057/2018	Public Health Wales Microbiology Cardiff	Public Health Wales Microbiology Cardiff	
EPI1275551	HA	United Kingdom	2018-Feb-07	EPI_ISL_321569	A/Anglesey/8900/2018	Public Health Wales Microbiology Cardiff	Public Health Wales Microbiology Cardiff	
EPI1275543	HA	United Kingdom	2018-Apr-15	EPI_ISL_321568	A/Caernarfon/8500/2018	Public Health Wales Microbiology Cardiff	Public Health Wales Microbiology Cardiff	
EPI1275535	HA	United Kingdom	2018-Apr-05	EPI_ISL_321567	A/Wrexham/7453/2018	Public Health Wales Microbiology Cardiff	Public Health Wales Microbiology Cardiff	
EPI1275528	HA	United Kingdom	2018-Mar-30	EPI_ISL_321566	A/Preston/7107/2018	Public Health Wales Microbiology Cardiff	Public Health Wales	

Segment ID	Segment	Country	Collection date	Isolate-ID	Isolate name	Originating Lab	Submitting Lab	Authors
							Microbiology Cardiff	
EPI1275520	HA	United Kingdom	2018-Mar-12	EPI_ISL_321565	A/Anglesey/5245/2018	Public Health Wales Microbiology Cardiff	Public Health Wales Microbiology Cardiff	
EPI1251822	HA	United Kingdom	2018-Mar-12	EPI_ISL_314000	A/Monmouth/0323/2018	Public Health Wales Microbiology Cardiff	Public Health Wales Microbiology Cardiff	
EPI1251818	HA	United Kingdom	2018-Mar-08	EPI_ISL_313999	A/Symonds-Yat/0271/2018	Public Health Wales Microbiology Cardiff	Public Health Wales Microbiology Cardiff	
EPI1251814	HA	United Kingdom	2018-Feb-26	EPI_ISL_313997	A/Powys/0208/2018	Public Health Wales Microbiology Cardiff	Public Health Wales Microbiology Cardiff	
EPI1251761	HA	United Kingdom	2018-Jan-30	EPI_ISL_313983	A/Caldicot/0050/2018	Public Health Wales Microbiology Cardiff	Public Health Wales Microbiology Cardiff	
EPI1251754	HA	United Kingdom	2018-Jan-25	EPI_ISL_313981	A/Port Talbot/0020/2018	Public Health Wales Microbiology Cardiff	Public Health Wales Microbiology Cardiff	
EPI1251725	HA	United Kingdom	2018-Jan-16	EPI_ISL_313974	A/Llanelli/7963/2018		Public Health Wales Microbiology Cardiff	
EPI1251718	HA	United Kingdom	2018-Jan-05	EPI_ISL_313971	A/Pontyclun/7902/2018	Public Health Wales Microbiology Cardiff	Public Health Wales Microbiology Cardiff	
EPI1251707	HA	United Kingdom	2018-Jan-05	EPI_ISL_313966	A/Neath/7890/2018	Public Health Wales Microbiology Cardiff	Public Health Wales Microbiology Cardiff	

Segment ID	Segment	Country	Collection date	Isolate-ID	Isolate name	Originating Lab	Submitting Lab	Authors
EPI1251704	HA	United Kingdom	2018-Jan-04	EPI_ISL_313965	A/Powys/7889/2018	Public Health Wales Microbiology Cardiff	Public Health Wales Microbiology Cardiff	
EPI1251691	HA	United Kingdom	2018-Jan-02	EPI_ISL_313958	A/Ross-on-Wye/7865/2018	Public Health Wales Microbiology Cardiff	Public Health Wales Microbiology Cardiff	
EPI1251689	HA	United Kingdom	2017-Dec-08	EPI_ISL_313957	A/Powys/7862/2017	Public Health Wales Microbiology Cardiff	Public Health Wales Microbiology Cardiff	
EPI1251687	HA	United Kingdom	2018-Jan-04	EPI_ISL_313956	A/Pontyclun/7859/2018	Public Health Wales Microbiology Cardiff	Public Health Wales Microbiology Cardiff	
EPI1251683	HA	United Kingdom	2017-Dec-27	EPI_ISL_313954	A/Neath/7855/2017	Public Health Wales Microbiology Cardiff	Public Health Wales Microbiology Cardiff	
EPI1251300	HA	United Kingdom	2017-Dec-04	EPI_ISL_313830	A/Powys/7718/2017	Public Health Wales Microbiology Cardiff	Public Health Wales Microbiology Cardiff	
EPI1251670	HA	United Kingdom	2017-Dec-04	EPI_ISL_313830	A/Powys/7718/2017	Public Health Wales Microbiology Cardiff	Public Health Wales Microbiology Cardiff	
EPI1251291	HA	United Kingdom	2017-Dec-29	EPI_ISL_313829	A/Port Talbot/7852/2017	Public Health Wales Microbiology Cardiff	Public Health Wales Microbiology Cardiff	
EPI1251681	HA	United Kingdom	2017-Dec-29	EPI_ISL_313829	A/Port Talbot/7852/2017	Public Health Wales Microbiology Cardiff	Public Health Wales Microbiology Cardiff	
EPI1281999	HA	United Kingdom	2017-Dec-29	EPI_ISL_313829	A/Port Talbot/7852/2017	Public Health Wales Microbiology Cardiff	Public Health Wales	

Segment ID	Segment	Country	Collection date	Isolate-ID	Isolate name	Originating Lab	Submitting Lab	Authors
							Microbiology Cardiff	
EPI1242325	HA	United Kingdom	2018-Feb-02	EPI_ISL_312063	A/England/81100461/2018	UK Health Security Agency - Colindale	UK Health Security Agency - Colindale	Galiano,M.
EPI1242317	HA	United Kingdom	2018-Feb-03	EPI_ISL_312062	A/England/81100460/2018	UK Health Security Agency - Colindale	UK Health Security Agency - Colindale	Galiano,M.
EPI1242309	HA	United Kingdom	2018-Feb-20	EPI_ISL_312061	A/England/81080591/2018	UK Health Security Agency - Colindale	UK Health Security Agency - Colindale	Galiano,M.
EPI1242301	HA	United Kingdom	2018-Feb-18	EPI_ISL_312060	A/England/81080590/2018	UK Health Security Agency - Colindale	UK Health Security Agency - Colindale	Galiano,M.
EPI1242285	HA	United Kingdom	2018-Feb-13	EPI_ISL_312058	A/England/81080588/2018	UK Health Security Agency - Colindale	UK Health Security Agency - Colindale	Galiano,M.
EPI1242277	HA	United Kingdom	2018-Feb-11	EPI_ISL_312057	A/England/81080584/2018	UK Health Security Agency - Colindale	UK Health Security Agency - Colindale	Galiano,M.
EPI1242269	HA	United Kingdom	2018-Feb-09	EPI_ISL_312056	A/England/81080582/2018	UK Health Security Agency - Colindale	UK Health Security Agency - Colindale	Galiano,M.
EPI1242261	HA	United Kingdom	2018-Mar-05	EPI_ISL_312055	A/England/81080577/2018	UK Health Security Agency - Colindale	UK Health Security Agency - Colindale	Galiano,M.
EPI1242253	HA	United Kingdom	2018-Mar-01	EPI_ISL_312054	A/England/81060632/2018	UK Health Security Agency - Colindale	UK Health Security Agency - Colindale	Galiano,M.
EPI1242245	HA	United Kingdom	2018-Mar-04	EPI_ISL_312053	A/England/81060625/2018	UK Health Security Agency - Colindale	UK Health Security Agency - Colindale	Galiano,M.
EPI1242237	HA	United Kingdom	2018-Mar-07	EPI_ISL_312052	A/England/540/2018	UK Health Security Agency - Colindale	UK Health Security Agency - Colindale	Galiano,M.
EPI1242229	HA	United Kingdom	2018-Mar-06	EPI_ISL_312051	A/England/81040771/2018	UK Health Security Agency - Colindale	UK Health Security Agency - Colindale	Galiano,M.

Segment ID	Segment	Country	Collection date	Isolate-ID	Isolate name	Originating Lab	Submitting Lab	Authors
EPI1242221	HA	United Kingdom	2018-Jan-24	EPI_ISL_312050	A/England/81040592/2018	UK Health Security Agency - Colindale	UK Health Security Agency - Colindale	Galiano,M.
EPI1242205	HA	United Kingdom	2018-Mar-04	EPI_ISL_312048	A/England/539/2018	UK Health Security Agency - Colindale	UK Health Security Agency - Colindale	Galiano,M.
EPI1242197	HA	United Kingdom	2018-Mar-02	EPI_ISL_312047	A/England/81020825/2018	UK Health Security Agency - Colindale	UK Health Security Agency - Colindale	Galiano,M.
EPI1242157	HA	United Kingdom	2018-Feb-26	EPI_ISL_312042	A/England/80960828/2018	UK Health Security Agency - Colindale	UK Health Security Agency - Colindale	Galiano,M.
EPI1242149	HA	United Kingdom	2018-Feb-26	EPI_ISL_312041	A/England/538/2018	UK Health Security Agency - Colindale	UK Health Security Agency - Colindale	Galiano,M.
EPI1242125	HA	United Kingdom	2018-Feb-22	EPI_ISL_312038	A/England/80920521/2018	UK Health Security Agency - Colindale	UK Health Security Agency - Colindale	Galiano,M.
EPI1242117	HA	United Kingdom	2018-Feb-21	EPI_ISL_312037	A/England/80920331/2018	UK Health Security Agency - Colindale	UK Health Security Agency - Colindale	Galiano,M.
EPI1242085	HA	United Kingdom	2018-Feb-21	EPI_ISL_312033	A/England/80880516/2018	UK Health Security Agency - Colindale	UK Health Security Agency - Colindale	Galiano,M.
EPI1242021	HA	United Kingdom	2018-Feb-21	EPI_ISL_312025	A/England/80880493/2018	UK Health Security Agency - Colindale	UK Health Security Agency - Colindale	Galiano,M.
EPI1241973	HA	United Kingdom	2018-Jan-21	EPI_ISL_312019	A/England/80840498/2018	UK Health Security Agency - Colindale	UK Health Security Agency - Colindale	Galiano,M.
EPI1241957	HA	United Kingdom	2018-Feb-19	EPI_ISL_312017	A/England/80840337/2018	UK Health Security Agency - Colindale	UK Health Security Agency - Colindale	Galiano,M.
EPI1241917	HA	United Kingdom	2018-Feb-15	EPI_ISL_312012	A/England/80820639/2018	UK Health Security Agency - Colindale	UK Health Security Agency - Colindale	Galiano,M.
EPI1241885	HA	United Kingdom	2018-Feb-02	EPI_ISL_312008	A/England/80580534/2018	UK Health Security Agency - Colindale	UK Health Security Agency - Colindale	Galiano,M.

Segment ID	Segment	Country	Collection date	Isolate-ID	Isolate name	Originating Lab	Submitting Lab	Authors
EPI1227597	HA	United Kingdom	2018-Mar-15	EPI_ISL_309473	A/Carmarthen/9096/2018	Public Health Wales Microbiology Cardiff	Public Health Wales Microbiology Cardiff	
EPI1227589	HA	United Kingdom	2018-Mar-16	EPI_ISL_309471	A/Cardiff/8383/2018	Public Health Wales Microbiology Cardiff	Public Health Wales Microbiology Cardiff	
EPI1227583	HA	United Kingdom	2018-Mar-17	EPI_ISL_309470	A/Barry/8372/2018	Public Health Wales Microbiology Cardiff	Public Health Wales Microbiology Cardiff	
EPI1227576	HA	United Kingdom	2018-Mar-16	EPI_ISL_309469	A/Cardiff/8360/2018	Public Health Wales Microbiology Cardiff	Public Health Wales Microbiology Cardiff	
EPI1227568	HA	United Kingdom	2018-Mar-15	EPI_ISL_309468	A/Cardiff/8328/2018	Public Health Wales Microbiology Cardiff	Public Health Wales Microbiology Cardiff	
EPI1227560	HA	United Kingdom	2018-Mar-13	EPI_ISL_309467	A/Penarth/8264/2018	Public Health Wales Microbiology Cardiff	Public Health Wales Microbiology Cardiff	
EPI1227552	HA	United Kingdom	2018-Mar-12	EPI_ISL_309466	A/Cardiff/8237/2018	Public Health Wales Microbiology Cardiff	Public Health Wales Microbiology Cardiff	
EPI1227541	HA	United Kingdom	2018-Mar-15	EPI_ISL_309465	A/Newport/8717/2018	Public Health Wales Microbiology Cardiff	Public Health Wales Microbiology Cardiff	
EPI1280809	HA	United Kingdom	2018-Mar-15	EPI_ISL_309465	A/Newport/8717/2018	Public Health Wales Microbiology Cardiff	Public Health Wales Microbiology Cardiff	
EPI1227523	HA	United Kingdom	2018-Mar-16	EPI_ISL_309462	A/Swansea/9497/2018	Public Health Wales Microbiology Cardiff	Public Health Wales	

Segment ID	Segment	Country	Collection date	Isolate-ID	Isolate name	Originating Lab	Submitting Lab	Authors
							Microbiology Cardiff	
EPI1227511	HA	United Kingdom	2018-Mar-16	EPI_ISL_309461	A/Swansea/9496/2018		Public Health Wales Microbiology Cardiff	
EPI1227487	HA	United Kingdom	2018-Mar-15	EPI_ISL_309460	A/Bridgend/9399/2018	Public Health Wales Microbiology Cardiff	Public Health Wales Microbiology Cardiff	
EPI1227480	HA	United Kingdom	2018-Mar-15	EPI_ISL_309459	A/Swansea/9391/2018	Public Health Wales Microbiology Cardiff	Public Health Wales Microbiology Cardiff	
EPI1227472	HA	United Kingdom	2018-Mar-15	EPI_ISL_309458	A/Swansea/9267/2018	Public Health Wales Microbiology Cardiff	Public Health Wales Microbiology Cardiff	
EPI1227464	HA	United Kingdom	2018-Mar-13	EPI_ISL_309457	A/Neath/9065/2018	Public Health Wales Microbiology Cardiff	Public Health Wales Microbiology Cardiff	
EPI1227455	HA	United Kingdom	2018-Mar-12	EPI_ISL_309456	A/Swansea/8779/2018	Public Health Wales Microbiology Cardiff	Public Health Wales Microbiology Cardiff	
EPI1227448	HA	United Kingdom	2018-Mar-06	EPI_ISL_309455	A/Swansea/7754/2018	Public Health Wales Microbiology Cardiff	Public Health Wales Microbiology Cardiff	
EPI1227441	HA	United Kingdom	2018-Mar-05	EPI_ISL_309454	A/Swansea/7582/2018	Public Health Wales Microbiology Cardiff	Public Health Wales Microbiology Cardiff	
EPI1227433	HA	United Kingdom	2018-Mar-01	EPI_ISL_309453	A/Bridgend/7331/2018	Public Health Wales Microbiology Cardiff	Public Health Wales Microbiology Cardiff	

Segment ID	Segment	Country	Collection date	Isolate-ID	Isolate name	Originating Lab	Submitting Lab	Authors
EPI1227427	HA	United Kingdom	2018-Mar-09	EPI_ISL_309452	A/Aberystwyth/5947/2018	Public Health Wales Microbiology Cardiff	Public Health Wales Microbiology Cardiff	
EPI1227421	HA	United Kingdom	2018-Mar-08	EPI_ISL_309451	A/Aberystwyth/5917/2018	Public Health Wales Microbiology Cardiff	Public Health Wales Microbiology Cardiff	
EPI1227413	HA	United Kingdom	2018-Mar-06	EPI_ISL_309450	A/Aberystwyth/5868/2018	Public Health Wales Microbiology Cardiff	Public Health Wales Microbiology Cardiff	
EPI1227405	HA	United Kingdom	2018-Mar-16	EPI_ISL_309449	A/Rhyl/5721/2018	Public Health Wales Microbiology Cardiff	Public Health Wales Microbiology Cardiff	
EPI1227397	HA	United Kingdom	2018-Mar-01	EPI_ISL_309448	A/Ammanford/7101/2018	Public Health Wales Microbiology Cardiff	Public Health Wales Microbiology Cardiff	
EPI1227389	HA	United Kingdom	2018-Mar-12	EPI_ISL_309447	A/Pwllheli/5246/2018	Public Health Wales Microbiology Cardiff	Public Health Wales Microbiology Cardiff	
EPI1227374	HA	United Kingdom	2018-Feb-21	EPI_ISL_309445	A/Swansea/5913/2018	Public Health Wales Microbiology Cardiff	Public Health Wales Microbiology Cardiff	
EPI1227366	HA	United Kingdom	2018-Feb-21	EPI_ISL_309444	A/Swansea/5724/2018	Public Health Wales Microbiology Cardiff	Public Health Wales Microbiology Cardiff	
EPI1227360	HA	United Kingdom	2018-Feb-20	EPI_ISL_309443	A/Port Talbot/5533/2018	Public Health Wales Microbiology Cardiff	Public Health Wales Microbiology Cardiff	
EPI1227353	HA	United Kingdom	2018-Feb-20	EPI_ISL_309442	A/Swansea/5501/2018	Public Health Wales Microbiology Cardiff	Public Health Wales	

Segment ID	Segment	Country	Collection date	Isolate-ID	Isolate name	Originating Lab	Submitting Lab	Authors
							Microbiology Cardiff	
EPI1227346	HA	United Kingdom	2018-Feb-20	EPI_ISL_309441	A/Swansea/5494/2018	Public Health Wales Microbiology Cardiff	Public Health Wales Microbiology Cardiff	
EPI1227260	HA	United Kingdom	2018-Mar-21	EPI_ISL_309430	A/Bridgend/4379/2018	Public Health Wales Microbiology Cardiff	Public Health Wales Microbiology Cardiff	
EPI1227252	HA	United Kingdom	2018-Mar-16	EPI_ISL_309429	A/Aberystwyth/6039/2018	Public Health Wales Microbiology Cardiff	Public Health Wales Microbiology Cardiff	
EPI1227236	HA	United Kingdom	2018-Mar-12	EPI_ISL_309428	A/Aberaeron/5961/2018	Public Health Wales Microbiology Cardiff	Public Health Wales Microbiology Cardiff	
EPI1227029	HA	United Kingdom	2018-Mar-10	EPI_ISL_309338	A/Aberystwyth/5949/2018	Public Health Wales Microbiology Cardiff	Public Health Wales Microbiology Cardiff	
EPI1220156	HA	United Kingdom	2018-Mar-19	EPI_ISL_307702	A/Cardiff/8379/2018	Public Health Wales Microbiology Cardiff	Public Health Wales Microbiology Cardiff	
EPI1220142	HA	United Kingdom	2018-Mar-19	EPI_ISL_307700	A/Cardiff/8371/2018	Public Health Wales Microbiology Cardiff	Public Health Wales Microbiology Cardiff	
EPI1220080	HA	United Kingdom	2018-Mar-14	EPI_ISL_307694	A/Cardiff/8307/2018	Public Health Wales Microbiology Cardiff	Public Health Wales Microbiology Cardiff	
EPI1220053	HA	United Kingdom	2018-Mar-14	EPI_ISL_307683	A/Cardiff/8285/2018	Public Health Wales Microbiology Cardiff	Public Health Wales Microbiology Cardiff	

Segment ID	Segment	Country	Collection date	Isolate-ID	Isolate name	Originating Lab	Submitting Lab	Authors
EPI1220045	HA	United Kingdom	2018-Mar-13	EPI_ISL_307682	A/Cardiff/8272/2018	Public Health Wales Microbiology Cardiff	Public Health Wales Microbiology Cardiff	
EPI1220030	HA	United Kingdom	2018-Apr-08	EPI_ISL_307679	A/Cardiff/8124/2018	Public Health Wales Microbiology Cardiff	Public Health Wales Microbiology Cardiff	
EPI1220022	HA	United Kingdom	2018-Mar-07	EPI_ISL_307677	A/Cardiff/8120/2018	Public Health Wales Microbiology Cardiff	Public Health Wales Microbiology Cardiff	
EPI1220009	HA	United Kingdom	2018-Mar-07	EPI_ISL_307675	A/Cardiff/8106/2018	Public Health Wales Microbiology Cardiff	Public Health Wales Microbiology Cardiff	
EPI1219939	HA	United Kingdom	2018-Mar-13	EPI_ISL_307665	A/Newport/8612/2018	Public Health Wales Microbiology Cardiff	Public Health Wales Microbiology Cardiff	
EPI1219940	HA	United Kingdom	2018-Mar-13	EPI_ISL_307665	A/Newport/8612/2018	Public Health Wales Microbiology Cardiff	Public Health Wales Microbiology Cardiff	
EPI1219850	HA	United Kingdom	2018-Mar-13	EPI_ISL_307651	A/Swansea/8785/2018	Public Health Wales Microbiology Cardiff	Public Health Wales Microbiology Cardiff	
EPI1219824	HA	United Kingdom	2018-Mar-01	EPI_ISL_307644	A/Swansea/7081/2018	Public Health Wales Microbiology Cardiff	Public Health Wales Microbiology Cardiff	
EPI1219786	HA	United Kingdom	2018-Feb-23	EPI_ISL_307639	A/Swansea/6191/2018	Public Health Wales Microbiology Cardiff	Public Health Wales Microbiology Cardiff	
EPI1219779	HA	United Kingdom	2018-Feb-21	EPI_ISL_307638	A/Swansea/5727/2018	Public Health Wales Microbiology Cardiff	Public Health Wales	

Segment ID	Segment	Country	Collection date	Isolate-ID	Isolate name	Originating Lab	Submitting Lab	Authors
							Microbiology Cardiff	
EPI1219770	HA	United Kingdom	2018-Mar-12	EPI_ISL_307636	A/Wrexham/1034/2018	Public Health Wales Microbiology Cardiff	Public Health Wales Microbiology Cardiff	
EPI1219762	HA	United Kingdom	2018-Mar-13	EPI_ISL_307635	A/Aberystwyth/5985/2018	Public Health Wales Microbiology Cardiff	Public Health Wales Microbiology Cardiff	
EPI1200334	HA	United Kingdom	2018-Jan-16	EPI_ISL_303519	A/England/80840506/2018	UK Health Security Agency - Colindale	UK Health Security Agency - Colindale	Galiano,M.
EPI1200326	HA	United Kingdom	2018-Feb-17	EPI_ISL_303515	A/England/80820629/2018	UK Health Security Agency - Colindale	UK Health Security Agency - Colindale	Galiano,M.
EPI1200312	HA	United Kingdom	2018-Jan-18	EPI_ISL_303508	A/England/80780609/2018	UK Health Security Agency - Colindale	UK Health Security Agency - Colindale	Galiano,M.
EPI1200271	HA	United Kingdom	2018-Jan-25	EPI_ISL_303486	A/England/80480677/2018	UK Health Security Agency - Colindale	UK Health Security Agency - Colindale	Galiano,M.
EPI1200182	HA	United Kingdom	2018-Jan-23	EPI_ISL_303441	A/England/80840505/2018	UK Health Security Agency - Colindale	UK Health Security Agency - Colindale	Galiano,M.
EPI1200158	HA	United Kingdom	2018-Feb-14	EPI_ISL_303438	A/England/80820627/2018	UK Health Security Agency - Colindale	UK Health Security Agency - Colindale	Galiano,M.
EPI1200142	HA	United Kingdom	2018-Feb-01	EPI_ISL_303436	A/England/80820621/2018	UK Health Security Agency - Colindale	UK Health Security Agency - Colindale	Galiano,M.
EPI1200134	HA	United Kingdom	2018-Feb-09	EPI_ISL_303435	A/England/80820616/2018	UK Health Security Agency - Colindale	UK Health Security Agency - Colindale	Galiano,M.
EPI1200126	HA	United Kingdom	2018-Feb-09	EPI_ISL_303434	A/England/80820615/2018	UK Health Security Agency - Colindale	UK Health Security Agency - Colindale	Galiano,M.

Segment ID	Segment	Country	Collection date	Isolate-ID	Isolate name	Originating Lab	Submitting Lab	Authors
EPI1200118	HA	United Kingdom	2018-Feb-13	EPI_ISL_303433	A/England/80820613/2018	UK Health Security Agency - Colindale	UK Health Security Agency - Colindale	Galiano,M.
EPI1200110	HA	United Kingdom	2018-Feb-13	EPI_ISL_303432	A/England/80820612/2018	UK Health Security Agency - Colindale	UK Health Security Agency - Colindale	Galiano,M.
EPI1200102	HA	United Kingdom	2018-Feb-10	EPI_ISL_303431	A/England/80820611/2018	UK Health Security Agency - Colindale	UK Health Security Agency - Colindale	Galiano,M.
EPI1200094	HA	United Kingdom	2018-Feb-12	EPI_ISL_303430	A/England/80820610/2018	UK Health Security Agency - Colindale	UK Health Security Agency - Colindale	Galiano,M.
EPI1200086	HA	United Kingdom	2018-Feb-15	EPI_ISL_303429	A/England/80800431/2018	UK Health Security Agency - Colindale	UK Health Security Agency - Colindale	Galiano,M.
EPI1200078	HA	United Kingdom	2018-Feb-13	EPI_ISL_303428	A/England/80800424/2018	UK Health Security Agency - Colindale	UK Health Security Agency - Colindale	Galiano,M.
EPI1200062	HA	United Kingdom	2018-Jan-18	EPI_ISL_303426	A/England/80780612/2018	UK Health Security Agency - Colindale	UK Health Security Agency - Colindale	Galiano,M.
EPI1200046	HA	United Kingdom	2018-Feb-09	EPI_ISL_303424	A/England/80760605/2018	UK Health Security Agency - Colindale	UK Health Security Agency - Colindale	Galiano,M.
EPI1200030	HA	United Kingdom	2018-Feb-06	EPI_ISL_303422	A/England/80760597/2018	UK Health Security Agency - Colindale	UK Health Security Agency - Colindale	Galiano,M.
EPI1200014	HA	United Kingdom	2018-Feb-08	EPI_ISL_303420	A/England/80720504/2018	UK Health Security Agency - Colindale	UK Health Security Agency - Colindale	Galiano,M.
EPI1200006	HA	United Kingdom	2018-Feb-10	EPI_ISL_303419	A/England/80720499/2018	UK Health Security Agency - Colindale	UK Health Security Agency - Colindale	Galiano,M.
EPI1199998	HA	United Kingdom	2018-Feb-07	EPI_ISL_303418	A/England/80720496/2018	UK Health Security Agency - Colindale	UK Health Security Agency - Colindale	Galiano,M.
EPI1199990	HA	United Kingdom	2018-Feb-09	EPI_ISL_303417	A/England/80720494/2018	UK Health Security Agency - Colindale	UK Health Security Agency - Colindale	Galiano,M.

Segment ID	Segment	Country	Collection date	Isolate-ID	Isolate name	Originating Lab	Submitting Lab	Authors
EPI1199974	HA	United Kingdom	2018-Feb-06	EPI_ISL_303415	A/England/80700661/2018	UK Health Security Agency - Colindale	UK Health Security Agency - Colindale	Galiano,M.
EPI1199966	HA	United Kingdom	2018-Feb-06	EPI_ISL_303414	A/England/80700659/2018	UK Health Security Agency - Colindale	UK Health Security Agency - Colindale	Galiano,M.
EPI1199950	HA	United Kingdom	2018-Jan-22	EPI_ISL_303412	A/England/80700594/2018	UK Health Security Agency - Colindale	UK Health Security Agency - Colindale	Galiano,M.
EPI1199934	HA	United Kingdom	2018-Jan-20	EPI_ISL_303410	A/England/80700591/2018	UK Health Security Agency - Colindale	UK Health Security Agency - Colindale	Galiano,M.
EPI1199926	HA	United Kingdom	2018-Jan-23	EPI_ISL_303409	A/England/80680610/2018	UK Health Security Agency - Colindale	UK Health Security Agency - Colindale	Galiano,M.
EPI1199918	HA	United Kingdom	2018-Feb-01	EPI_ISL_303408	A/England/80680594/2018	UK Health Security Agency - Colindale	UK Health Security Agency - Colindale	Galiano,M.
EPI1199910	HA	United Kingdom	2018-Feb-05	EPI_ISL_303407	A/England/80680593/2018	UK Health Security Agency - Colindale	UK Health Security Agency - Colindale	Galiano,M.
EPI1199902	HA	United Kingdom	2018-Jan-31	EPI_ISL_303406	A/England/80680588/2018	UK Health Security Agency - Colindale	UK Health Security Agency - Colindale	Galiano,M.
EPI1199894	HA	United Kingdom	2018-Feb-06	EPI_ISL_303405	A/England/80680583/2018	UK Health Security Agency - Colindale	UK Health Security Agency - Colindale	Galiano,M.
EPI1199854	HA	United Kingdom	2018-Feb-02	EPI_ISL_303400	A/England/80620359/2018	UK Health Security Agency - Colindale	UK Health Security Agency - Colindale	Galiano,M.
EPI1199822	HA	United Kingdom	2018-Feb-01	EPI_ISL_303396	A/England/80600316/2018	UK Health Security Agency - Colindale	UK Health Security Agency - Colindale	Galiano,M.
EPI1199814	HA	United Kingdom	2018-Feb-01	EPI_ISL_303395	A/England/80600305/2018	UK Health Security Agency - Colindale	UK Health Security Agency - Colindale	Galiano,M.
EPI1199806	HA	United Kingdom	2018-Feb-01	EPI_ISL_303394	A/England/80600014/2018	UK Health Security Agency - Colindale	UK Health Security Agency - Colindale	Galiano,M.

Segment ID	Segment	Country	Collection date	Isolate-ID	Isolate name	Originating Lab	Submitting Lab	Authors
EPI1199798	HA	United Kingdom	2018-Jan-29	EPI_ISL_303393	A/England/80600009/2018	UK Health Security Agency - Colindale	UK Health Security Agency - Colindale	Galiano,M.
EPI1199782	HA	United Kingdom	2018-Feb-02	EPI_ISL_303391	A/England/80600004/2018	UK Health Security Agency - Colindale	UK Health Security Agency - Colindale	Galiano,M.
EPI1199766	HA	United Kingdom	2018-Jan-31	EPI_ISL_303389	A/England/80580478/2018	UK Health Security Agency - Colindale	UK Health Security Agency - Colindale	Galiano,M.
EPI1199702	HA	United Kingdom	2018-Jan-31	EPI_ISL_303381	A/England/80580460/2018	UK Health Security Agency - Colindale	UK Health Security Agency - Colindale	Galiano,M.
EPI1199694	HA	United Kingdom	2018-Feb-01	EPI_ISL_303380	A/England/80580458/2018	UK Health Security Agency - Colindale	UK Health Security Agency - Colindale	Galiano,M.
EPI1199654	HA	United Kingdom	2018-Jan-16	EPI_ISL_303375	A/England/80540709/2018	UK Health Security Agency - Colindale	UK Health Security Agency - Colindale	Galiano,M.
EPI1199646	HA	United Kingdom	2018-Jan-13	EPI_ISL_303374	A/England/80540707/2018	UK Health Security Agency - Colindale	UK Health Security Agency - Colindale	Galiano,M.
EPI1199598	HA	United Kingdom	2018-Jan-24	EPI_ISL_303368	A/England/80520641/2018	UK Health Security Agency - Colindale	UK Health Security Agency - Colindale	Galiano,M.
EPI1199590	HA	United Kingdom	2018-Jan-29	EPI_ISL_303367	A/England/80520360/2018	UK Health Security Agency - Colindale	UK Health Security Agency - Colindale	Galiano,M.
EPI1199582	HA	United Kingdom	2018-Jan-29	EPI_ISL_303366	A/England/80520342/2018	UK Health Security Agency - Colindale	UK Health Security Agency - Colindale	Galiano,M.
EPI1199566	HA	United Kingdom	2018-Jan-26	EPI_ISL_303364	A/England/80520335/2018	UK Health Security Agency - Colindale	UK Health Security Agency - Colindale	Galiano,M.
EPI1199558	HA	United Kingdom	2018-Jan-25	EPI_ISL_303363	A/England/80500558/2018	UK Health Security Agency - Colindale	UK Health Security Agency - Colindale	Galiano,M.
EPI1199550	HA	United Kingdom	2018-Jan-14	EPI_ISL_303362	A/England/80500548/2018	UK Health Security Agency - Colindale	UK Health Security Agency - Colindale	Galiano,M.

Segment ID	Segment	Country	Collection date	Isolate-ID	Isolate name	Originating Lab	Submitting Lab	Authors
EPI1199542	HA	United Kingdom	2018-Jan-25	EPI_ISL_303361	A/England/80500371/2018	UK Health Security Agency - Colindale	UK Health Security Agency - Colindale	Galiano,M.
EPI1199534	HA	United Kingdom	2018-Jan-25	EPI_ISL_303360	A/England/80500319/2018	UK Health Security Agency - Colindale	UK Health Security Agency - Colindale	Galiano,M.
EPI1199526	HA	United Kingdom	2018-Jan-18	EPI_ISL_303359	A/England/80480665/2018	UK Health Security Agency - Colindale	UK Health Security Agency - Colindale	Galiano,M.
EPI1199494	HA	United Kingdom	2018-Jan-22	EPI_ISL_303355	A/England/80480630/2018	UK Health Security Agency - Colindale	UK Health Security Agency - Colindale	Galiano,M.
EPI1199478	HA	United Kingdom	2018-Jan-17	EPI_ISL_303353	A/England/80480620/2018	UK Health Security Agency - Colindale	UK Health Security Agency - Colindale	Galiano,M.
EPI1199470	HA	United Kingdom	2018-Jan-23	EPI_ISL_303352	A/England/80480609/2018	UK Health Security Agency - Colindale	UK Health Security Agency - Colindale	Galiano,M.
EPI1199462	HA	United Kingdom	2018-Jan-22	EPI_ISL_303351	A/England/80480581/2018	UK Health Security Agency - Colindale	UK Health Security Agency - Colindale	Galiano,M.
EPI1199438	HA	United Kingdom	2018-Jan-23	EPI_ISL_303348	A/England/80460453/2018	UK Health Security Agency - Colindale	UK Health Security Agency - Colindale	Galiano,M.
EPI1199430	HA	United Kingdom	2018-Jan-23	EPI_ISL_303347	A/England/80460428/2018	UK Health Security Agency - Colindale	UK Health Security Agency - Colindale	Galiano,M.
EPI1199406	HA	United Kingdom	2018-Jan-21	EPI_ISL_303344	A/England/80440609/2018	UK Health Security Agency - Colindale	UK Health Security Agency - Colindale	Galiano,M.
EPI1199390	HA	United Kingdom	2018-Jan-22	EPI_ISL_303342	A/England/80440421/2018	UK Health Security Agency - Colindale	UK Health Security Agency - Colindale	Galiano,M.
EPI1199382	HA	United Kingdom	2018-Jan-22	EPI_ISL_303341	A/England/80440407/2018	UK Health Security Agency - Colindale	UK Health Security Agency - Colindale	Galiano,M.
EPI1199374	HA	United Kingdom	2018-Jan-19	EPI_ISL_303340	A/England/80440405/2018	UK Health Security Agency - Colindale	UK Health Security Agency - Colindale	Galiano,M.

Segment ID	Segment	Country	Collection date	Isolate-ID	Isolate name	Originating Lab	Submitting Lab	Authors
EPI1199334	HA	United Kingdom	2018-Jan-22	EPI_ISL_303335	A/England/80420415/2018	UK Health Security Agency - Colindale	UK Health Security Agency - Colindale	Galiano,M.
EPI1199310	HA	United Kingdom	2018-Jan-22	EPI_ISL_303332	A/England/80420407/2018	UK Health Security Agency - Colindale	UK Health Security Agency - Colindale	Galiano,M.
EPI1199286	HA	United Kingdom	2018-Jan-19	EPI_ISL_303329	A/England/80400409/2018	UK Health Security Agency - Colindale	UK Health Security Agency - Colindale	Galiano,M.
EPI1199278	HA	United Kingdom	2018-Jan-18	EPI_ISL_303328	A/England/80400408/2018	UK Health Security Agency - Colindale	UK Health Security Agency - Colindale	Galiano,M.
EPI1199254	HA	United Kingdom	2018-Jan-22	EPI_ISL_303325	A/England/80400394/2018	UK Health Security Agency - Colindale	UK Health Security Agency - Colindale	Galiano,M.
EPI1199222	HA	United Kingdom	2018-Jan-17	EPI_ISL_303321	A/England/80380578/2018	UK Health Security Agency - Colindale	UK Health Security Agency - Colindale	Galiano,M.
EPI1199190	HA	United Kingdom	2018-Jan-16	EPI_ISL_303317	A/England/80360397/2018	UK Health Security Agency - Colindale	UK Health Security Agency - Colindale	Galiano,M.
EPI1199134	HA	United Kingdom	2018-Jan-09	EPI_ISL_303310	A/England/80260584/2018	UK Health Security Agency - Colindale	UK Health Security Agency - Colindale	Galiano,M.
EPI1189308	HA	United Kingdom	2018-Jan-23	EPI_ISL_301542	A/England/80520648/2018	UK Health Security Agency - Colindale	UK Health Security Agency - Colindale	Galiano,M.
EPI1189306	HA	United Kingdom	2018-Jan-15	EPI_ISL_301541	A/England/80520639/2018	UK Health Security Agency - Colindale	UK Health Security Agency - Colindale	Galiano,M.
EPI1189302	HA	United Kingdom	2018-Jan-20	EPI_ISL_301539	A/England/80480631/2018	UK Health Security Agency - Colindale	UK Health Security Agency - Colindale	Galiano,M.
EPI1189300	HA	United Kingdom	2018-Jan-16	EPI_ISL_301538	A/England/80480624/2018	UK Health Security Agency - Colindale	UK Health Security Agency - Colindale	Galiano,M.
EPI1189298	HA	United Kingdom	2018-Jan-12	EPI_ISL_301537	A/N.Ireland/80480595/2018	UK Health Security Agency - Colindale	UK Health Security Agency - Colindale	Galiano,M.

Segment ID	Segment	Country	Collection date	Isolate-ID	Isolate name	Originating Lab	Submitting Lab	Authors
EPI1189296	HA	United Kingdom	2018-Jan-14	EPI_ISL_301536	A/N.Ireland/80480591/2018	UK Health Security Agency - Colindale	UK Health Security Agency - Colindale	Galiano,M.
EPI1189294	HA	United Kingdom	2018-Jan-14	EPI_ISL_301535	A/England/80480577/2018	UK Health Security Agency - Colindale	UK Health Security Agency - Colindale	Galiano,M.
EPI1189286	HA	United Kingdom	2018-Jan-12	EPI_ISL_301530	A/England/80420857/2018	UK Health Security Agency - Colindale	UK Health Security Agency - Colindale	Galiano,M.
EPI1189284	HA	United Kingdom	2018-Jan-11	EPI_ISL_301529	A/England/80420853/2018	UK Health Security Agency - Colindale	UK Health Security Agency - Colindale	Galiano,M.
EPI1189282	HA	United Kingdom	2018-Jan-11	EPI_ISL_301528	A/England/80420844/2018	UK Health Security Agency - Colindale	UK Health Security Agency - Colindale	Galiano,M.
EPI1189280	HA	United Kingdom	2018-Jan-20	EPI_ISL_301527	A/England/80420672/2018	UK Health Security Agency - Colindale	UK Health Security Agency - Colindale	Galiano,M.
EPI1189278	HA	United Kingdom	2018-Jan-08	EPI_ISL_301526	A/England/80420664/2018	UK Health Security Agency - Colindale	UK Health Security Agency - Colindale	Galiano,M.
EPI1189276	HA	United Kingdom	2018-Jan-02	EPI_ISL_301525	A/N.Ireland/80400597/2018	UK Health Security Agency - Colindale	UK Health Security Agency - Colindale	Galiano,M.
EPI1189274	HA	United Kingdom	2018-Jan-02	EPI_ISL_301524	A/N.Ireland/80400596/2018	UK Health Security Agency - Colindale	UK Health Security Agency - Colindale	Galiano,M.
EPI1189257	HA	United Kingdom	2018-Jan-18	EPI_ISL_301512	A/England/80540708/2018	UK Health Security Agency - Colindale	UK Health Security Agency - Colindale	Galiano,M.
EPI1189249	HA	United Kingdom	2018-Jan-27	EPI_ISL_301511	A/England/80520665/2018	UK Health Security Agency - Colindale	UK Health Security Agency - Colindale	Galiano,M.
EPI1189241	HA	United Kingdom	2018-Jan-25	EPI_ISL_301510	A/England/80520663/2018	UK Health Security Agency - Colindale	UK Health Security Agency - Colindale	Galiano,M.
EPI1189233	HA	United Kingdom	2018-Jan-27	EPI_ISL_301509	A/England/80520643/2018	UK Health Security Agency - Colindale	UK Health Security Agency - Colindale	Galiano,M.

Segment ID	Segment	Country	Collection date	Isolate-ID	Isolate name	Originating Lab	Submitting Lab	Authors
EPI1189217	HA	United Kingdom	2018-Jan-22	EPI_ISL_301507	A/England/80480638/2018	UK Health Security Agency - Colindale	UK Health Security Agency - Colindale	Galiano,M.
EPI1189209	HA	United Kingdom	2018-Jan-18	EPI_ISL_301506	A/England/80480621/2018	UK Health Security Agency - Colindale	UK Health Security Agency - Colindale	Galiano,M.
EPI1189201	HA	United Kingdom	2018-Jan-17	EPI_ISL_301505	A/England/80480618/2018	UK Health Security Agency - Colindale	UK Health Security Agency - Colindale	Galiano,M.
EPI1189193	HA	United Kingdom	2018-Jan-22	EPI_ISL_301504	A/England/80480617/2018	UK Health Security Agency - Colindale	UK Health Security Agency - Colindale	Galiano,M.
EPI1189185	HA	United Kingdom	2018-Jan-18	EPI_ISL_301503	A/England/80480616/2018	UK Health Security Agency - Colindale	UK Health Security Agency - Colindale	Galiano,M.
EPI1189177	HA	United Kingdom	2018-Jan-22	EPI_ISL_301502	A/England/80480612/2018	UK Health Security Agency - Colindale	UK Health Security Agency - Colindale	Galiano,M.
EPI1189169	HA	United Kingdom	2018-Jan-16	EPI_ISL_301501	A/England/80480611/2018	UK Health Security Agency - Colindale	UK Health Security Agency - Colindale	Galiano,M.
EPI1189161	HA	United Kingdom	2018-Jan-23	EPI_ISL_301500	A/England/80480607/2018	UK Health Security Agency - Colindale	UK Health Security Agency - Colindale	Galiano,M.
EPI1189153	HA	United Kingdom	2018-Jan-24	EPI_ISL_301499	A/England/80480600/2018	UK Health Security Agency - Colindale	UK Health Security Agency - Colindale	Galiano,M.
EPI1189145	HA	United Kingdom	2018-Jan-15	EPI_ISL_301498	A/N.Ireland/80480597/2018	UK Health Security Agency - Colindale	UK Health Security Agency - Colindale	Galiano,M.
EPI1189137	HA	United Kingdom	2018-Jan-22	EPI_ISL_301497	A/England/80480582/2018	UK Health Security Agency - Colindale	UK Health Security Agency - Colindale	Galiano,M.
EPI1189129	HA	United Kingdom	2018-Jan-20	EPI_ISL_301496	A/England/80480571/2018	UK Health Security Agency - Colindale	UK Health Security Agency - Colindale	Galiano,M.
EPI1189121	HA	United Kingdom	2018-Jan-24	EPI_ISL_301495	A/England/80460723/2018	UK Health Security Agency - Colindale	UK Health Security Agency - Colindale	Galiano,M.

Segment ID	Segment	Country	Collection date	Isolate-ID	Isolate name	Originating Lab	Submitting Lab	Authors
EPI1189113	HA	United Kingdom	2018-Jan-22	EPI_ISL_301494	A/England/80460722/2018	UK Health Security Agency - Colindale	UK Health Security Agency - Colindale	Galiano,M.
EPI1189097	HA	United Kingdom	2018-Jan-18	EPI_ISL_301492	A/England/80440632/2018	UK Health Security Agency - Colindale	UK Health Security Agency - Colindale	Galiano,M.
EPI1189089	HA	United Kingdom	2018-Jan-18	EPI_ISL_301491	A/England/80440623/2018	UK Health Security Agency - Colindale	UK Health Security Agency - Colindale	Galiano,M.
EPI1189081	HA	United Kingdom	2018-Jan-19	EPI_ISL_301490	A/England/80440618/2018	UK Health Security Agency - Colindale	UK Health Security Agency - Colindale	Galiano,M.
EPI1189073	HA	United Kingdom	2018-Jan-20	EPI_ISL_301489	A/England/80440617/2018	UK Health Security Agency - Colindale	UK Health Security Agency - Colindale	Galiano,M.
EPI1189065	HA	United Kingdom	2018-Jan-22	EPI_ISL_301488	A/England/80440614/2018	UK Health Security Agency - Colindale	UK Health Security Agency - Colindale	Galiano,M.
EPI1189049	HA	United Kingdom	2018-Jan-22	EPI_ISL_301486	A/England/80440612/2018	UK Health Security Agency - Colindale	UK Health Security Agency - Colindale	Galiano,M.
EPI1189041	HA	United Kingdom	2018-Jan-22	EPI_ISL_301485	A/England/80440611/2018	UK Health Security Agency - Colindale	UK Health Security Agency - Colindale	Galiano,M.
EPI1189033	HA	United Kingdom	2018-Jan-21	EPI_ISL_301484	A/England/80440601/2018	UK Health Security Agency - Colindale	UK Health Security Agency - Colindale	Galiano,M.
EPI1189017	HA	United Kingdom	2018-Jan-13	EPI_ISL_301482	A/England/80420878/2018	UK Health Security Agency - Colindale	UK Health Security Agency - Colindale	Galiano,M.
EPI1189009	HA	United Kingdom	2018-Jan-14	EPI_ISL_301481	A/England/80420877/2018	UK Health Security Agency - Colindale	UK Health Security Agency - Colindale	Galiano,M.
EPI1189001	HA	United Kingdom	2018-Jan-17	EPI_ISL_301480	A/England/80420875/2018	UK Health Security Agency - Colindale	UK Health Security Agency - Colindale	Galiano,M.
EPI1188985	HA	United Kingdom	2018-Jan-10	EPI_ISL_301478	A/England/80420869/2018	UK Health Security Agency - Colindale	UK Health Security Agency - Colindale	Galiano,M.

Segment ID	Segment	Country	Collection date	Isolate-ID	Isolate name	Originating Lab	Submitting Lab	Authors
EPI1188977	HA	United Kingdom	2018-Jan-13	EPI_ISL_301477	A/England/80420865/2018	UK Health Security Agency - Colindale	UK Health Security Agency - Colindale	Galiano,M.
EPI1188969	HA	United Kingdom	2018-Jan-12	EPI_ISL_301476	A/England/80420860/2018	UK Health Security Agency - Colindale	UK Health Security Agency - Colindale	Galiano,M.
EPI1188961	HA	United Kingdom	2018-Jan-11	EPI_ISL_301475	A/England/80420848/2018	UK Health Security Agency - Colindale	UK Health Security Agency - Colindale	Galiano,M.
EPI1188953	HA	United Kingdom	2018-Jan-11	EPI_ISL_301474	A/England/80420847/2018	UK Health Security Agency - Colindale	UK Health Security Agency - Colindale	Galiano,M.
EPI1188945	HA	United Kingdom	2018-Jan-11	EPI_ISL_301473	A/England/80420846/2018	UK Health Security Agency - Colindale	UK Health Security Agency - Colindale	Galiano,M.
EPI1188937	HA	United Kingdom	2018-Jan-09	EPI_ISL_301472	A/England/80420843/2018	UK Health Security Agency - Colindale	UK Health Security Agency - Colindale	Galiano,M.
EPI1188929	HA	United Kingdom	2018-Jan-21	EPI_ISL_301471	A/England/80420673/2018	UK Health Security Agency - Colindale	UK Health Security Agency - Colindale	Galiano,M.
EPI1188921	HA	United Kingdom	2018-Jan-17	EPI_ISL_301470	A/England/80420661/2018	UK Health Security Agency - Colindale	UK Health Security Agency - Colindale	Galiano,M.
EPI1188913	HA	United Kingdom	2018-Jan-16	EPI_ISL_301469	A/England/80420659/2018	UK Health Security Agency - Colindale	UK Health Security Agency - Colindale	Galiano,M.
EPI1188905	HA	United Kingdom	2018-Jan-16	EPI_ISL_301468	A/England/80420658/2018	UK Health Security Agency - Colindale	UK Health Security Agency - Colindale	Galiano,M.
EPI1188897	HA	United Kingdom	2018-Jan-16	EPI_ISL_301467	A/England/80420657/2018	UK Health Security Agency - Colindale	UK Health Security Agency - Colindale	Galiano,M.
EPI1188889	HA	United Kingdom	2018-Jan-16	EPI_ISL_301466	A/England/80420655/2018	UK Health Security Agency - Colindale	UK Health Security Agency - Colindale	Galiano,M.
EPI1188881	HA	United Kingdom	2018-Jan-17	EPI_ISL_301465	A/England/80420642/2018	UK Health Security Agency - Colindale	UK Health Security Agency - Colindale	Galiano,M.

Segment ID	Segment	Country	Collection date	Isolate-ID	Isolate name	Originating Lab	Submitting Lab	Authors
EPI1188873	HA	United Kingdom	2018-Jan-22	EPI_ISL_301464	A/England/80420641/2018	UK Health Security Agency - Colindale	UK Health Security Agency - Colindale	Galiano,M.
EPI1188865	HA	United Kingdom	2018-Jan-17	EPI_ISL_301463	A/England/80400624/2018	UK Health Security Agency - Colindale	UK Health Security Agency - Colindale	Galiano,M.
EPI1188857	HA	United Kingdom	2018-Jan-17	EPI_ISL_301462	A/England/80400622/2018	UK Health Security Agency - Colindale	UK Health Security Agency - Colindale	Galiano,M.
EPI1188849	HA	United Kingdom	2018-Jan-22	EPI_ISL_301461	A/England/80400620/2018	UK Health Security Agency - Colindale	UK Health Security Agency - Colindale	Galiano,M.
EPI1188809	HA	United Kingdom	2018-Jan-16	EPI_ISL_301456	A/England/80380726/2018	UK Health Security Agency - Colindale	UK Health Security Agency - Colindale	Galiano,M.
EPI1188793	HA	United Kingdom	2018-Jan-15	EPI_ISL_301454	A/England/80380716/2018	UK Health Security Agency - Colindale	UK Health Security Agency - Colindale	Galiano,M.
EPI1188785	HA	United Kingdom	2018-Jan-15	EPI_ISL_301453	A/England/80380713/2018	UK Health Security Agency - Colindale	UK Health Security Agency - Colindale	Galiano,M.
EPI1188777	HA	United Kingdom	2018-Jan-08	EPI_ISL_301452	A/England/80380708/2018	UK Health Security Agency - Colindale	UK Health Security Agency - Colindale	Galiano,M.
EPI1188769	HA	United Kingdom	2018-Jan-08	EPI_ISL_301451	A/England/80380705/2018	UK Health Security Agency - Colindale	UK Health Security Agency - Colindale	Galiano,M.
EPI1188761	HA	United Kingdom	2018-Jan-06	EPI_ISL_301450	A/England/80380701/2018	UK Health Security Agency - Colindale	UK Health Security Agency - Colindale	Galiano,M.
EPI1188753	HA	United Kingdom	2018-Jan-09	EPI_ISL_301449	A/England/80380683/2018	UK Health Security Agency - Colindale	UK Health Security Agency - Colindale	Galiano,M.
EPI1188745	HA	United Kingdom	2018-Jan-13	EPI_ISL_301448	A/England/80380678/2018	UK Health Security Agency - Colindale	UK Health Security Agency - Colindale	Galiano,M.
EPI1188737	HA	United Kingdom	2018-Jan-09	EPI_ISL_301447	A/England/80380674/2018	UK Health Security Agency - Colindale	UK Health Security Agency - Colindale	Galiano,M.

Segment ID	Segment	Country	Collection date	Isolate-ID	Isolate name	Originating Lab	Submitting Lab	Authors
EPI1188729	HA	United Kingdom	2018-Jan-09	EPI_ISL_301446	A/England/80380665/2018	UK Health Security Agency - Colindale	UK Health Security Agency - Colindale	Galiano,M.
EPI1188721	HA	United Kingdom	2018-Jan-09	EPI_ISL_301445	A/England/80380664/2018	UK Health Security Agency - Colindale	UK Health Security Agency - Colindale	Galiano,M.
EPI1188705	HA	United Kingdom	2018-Jan-05	EPI_ISL_301443	A/England/80380656/2018	UK Health Security Agency - Colindale	UK Health Security Agency - Colindale	Galiano,M.
EPI1188689	HA	United Kingdom	2018-Jan-06	EPI_ISL_301441	A/England/80380650/2018	UK Health Security Agency - Colindale	UK Health Security Agency - Colindale	Galiano,M.
EPI1188681	HA	United Kingdom	2018-Jan-06	EPI_ISL_301440	A/England/80380649/2018	UK Health Security Agency - Colindale	UK Health Security Agency - Colindale	Galiano,M.
EPI1188673	HA	United Kingdom	2018-Jan-05	EPI_ISL_301439	A/England/80380646/2018	UK Health Security Agency - Colindale	UK Health Security Agency - Colindale	Galiano,M.
EPI1188665	HA	United Kingdom	2018-Jan-04	EPI_ISL_301438	A/England/80380642/2018	UK Health Security Agency - Colindale	UK Health Security Agency - Colindale	Galiano,M.
EPI1188657	HA	United Kingdom	2018-Jan-01	EPI_ISL_301437	A/England/80380636/2018	UK Health Security Agency - Colindale	UK Health Security Agency - Colindale	Galiano,M.
EPI1188649	HA	United Kingdom	2018-Jan-04	EPI_ISL_301436	A/England/80380635/2018	UK Health Security Agency - Colindale	UK Health Security Agency - Colindale	Galiano,M.
EPI1188641	HA	United Kingdom	2018-Jan-17	EPI_ISL_301435	A/England/80360710/2018	UK Health Security Agency - Colindale	UK Health Security Agency - Colindale	Galiano,M.
EPI1188633	HA	United Kingdom	2018-Jan-17	EPI_ISL_301434	A/England/80360697/2018	UK Health Security Agency - Colindale	UK Health Security Agency - Colindale	Galiano,M.
EPI1188625	HA	United Kingdom	2018-Jan-17	EPI_ISL_301433	A/England/80360696/2018	UK Health Security Agency - Colindale	UK Health Security Agency - Colindale	Galiano,M.
EPI1188617	HA	United Kingdom	2018-Jan-17	EPI_ISL_301432	A/England/80360690/2018	UK Health Security Agency - Colindale	UK Health Security Agency - Colindale	Galiano,M.

Segment ID	Segment	Country	Collection date	Isolate-ID	Isolate name	Originating Lab	Submitting Lab	Authors
EPI1188609	HA	United Kingdom	2018-Jan-17	EPI_ISL_301431	A/England/80360540/2018	UK Health Security Agency - Colindale	UK Health Security Agency - Colindale	Galiano,M.
EPI1188601	HA	United Kingdom	2018-Jan-17	EPI_ISL_301430	A/England/80360537/2018	UK Health Security Agency - Colindale	UK Health Security Agency - Colindale	Galiano,M.
EPI1188593	HA	United Kingdom	2018-Jan-17	EPI_ISL_301429	A/England/80360523/2018	UK Health Security Agency - Colindale	UK Health Security Agency - Colindale	Galiano,M.
EPI1188585	HA	United Kingdom	2018-Jan-18	EPI_ISL_301428	A/England/80360521/2018	UK Health Security Agency - Colindale	UK Health Security Agency - Colindale	Galiano,M.
EPI1188577	HA	United Kingdom	2018-Jan-07	EPI_ISL_301427	A/England/80360519/2018	UK Health Security Agency - Colindale	UK Health Security Agency - Colindale	Galiano,M.
EPI1188569	HA	United Kingdom	2018-Jan-13	EPI_ISL_301426	A/England/80360516/2018	UK Health Security Agency - Colindale	UK Health Security Agency - Colindale	Galiano,M.
EPI1188561	HA	United Kingdom	2018-Jan-13	EPI_ISL_301425	A/England/80360509/2018	UK Health Security Agency - Colindale	UK Health Security Agency - Colindale	Galiano,M.
EPI1188553	HA	United Kingdom	2018-Jan-18	EPI_ISL_301424	A/England/80360502/2018	UK Health Security Agency - Colindale	UK Health Security Agency - Colindale	Galiano,M.
EPI1188545	HA	United Kingdom	2018-Jan-15	EPI_ISL_301423	A/England/80360500/2018	UK Health Security Agency - Colindale	UK Health Security Agency - Colindale	Galiano,M.
EPI1188529	HA	United Kingdom	2018-Jan-11	EPI_ISL_301421	A/England/80320698/2018	UK Health Security Agency - Colindale	UK Health Security Agency - Colindale	Galiano,M.
EPI1188513	HA	United Kingdom	2018-Jan-12	EPI_ISL_301419	A/England/80320650/2018	UK Health Security Agency - Colindale	UK Health Security Agency - Colindale	Galiano,M.
EPI1188505	HA	United Kingdom	2018-Jan-11	EPI_ISL_301418	A/England/80320649/2018	UK Health Security Agency - Colindale	UK Health Security Agency - Colindale	Galiano,M.
EPI1188497	HA	United Kingdom	2018-Jan-09	EPI_ISL_301417	A/England/80320647/2018	UK Health Security Agency - Colindale	UK Health Security Agency - Colindale	Galiano,M.

Segment ID	Segment	Country	Collection date	Isolate-ID	Isolate name	Originating Lab	Submitting Lab	Authors
EPI1188481	HA	United Kingdom	2017-Dec-07	EPI_ISL_301415	B/England/186/2017	UK Health Security Agency - Colindale	UK Health Security Agency - Colindale	Galiano,M.
EPI1188448	HA	United Kingdom	2018-Jan-29	EPI_ISL_301411	A/Northern Ireland/5090/2018	Regional Virus Laboratory, Microbiology Department, Royal Victoria Hospital	Crick Worldwide Influenza Centre	
EPI1188446	HA	United Kingdom	2018-Jan-28	EPI_ISL_301410	A/Northern Ireland/5059/2018	Regional Virus Laboratory, Microbiology Department, Royal Victoria Hospital	Crick Worldwide Influenza Centre	
EPI1188444	HA	United Kingdom	2018-Jan-28	EPI_ISL_301409	A/Northern Ireland/5053/2018	Regional Virus Laboratory, Microbiology Department, Royal Victoria Hospital	Crick Worldwide Influenza Centre	
EPI1188442	HA	United Kingdom	2018-Jan-28	EPI_ISL_301408	A/Northern Ireland/4948/2018	Regional Virus Laboratory, Microbiology Department, Royal Victoria Hospital	Crick Worldwide Influenza Centre	
EPI1188440	HA	United Kingdom	2018-Jan-27	EPI_ISL_301407	A/Northern Ireland/4903/2018	Regional Virus Laboratory, Microbiology Department, Royal Victoria Hospital	Crick Worldwide Influenza Centre	
EPI1188436	HA	United Kingdom	2018-Jan-26	EPI_ISL_301404	A/Northern Ireland/4761/2018	Regional Virus Laboratory, Microbiology Department, Royal Victoria Hospital	Crick Worldwide Influenza Centre	
EPI1188434	HA	United Kingdom	2018-Jan-24	EPI_ISL_301403	A/Northern Ireland/4594/2018	Regional Virus Laboratory, Microbiology Department, Royal Victoria Hospital	Crick Worldwide Influenza Centre	

Segment ID	Segment	Country	Collection date	Isolate-ID	Isolate name	Originating Lab	Submitting Lab	Authors
EPI1188432	HA	United Kingdom	2018-Jan-23	EPI_ISL_301402	A/Northern Ireland/4453/2018	Regional Virus Laboratory, Microbiology Department, Royal Victoria Hospital	Crick Worldwide Influenza Centre	
EPI1188428	HA	United Kingdom	2018-Jan-19	EPI_ISL_301399	A/Northern Ireland/3645/2018	Regional Virus Laboratory, Microbiology Department, Royal Victoria Hospital	Crick Worldwide Influenza Centre	
EPI1188426	HA	United Kingdom	2018-Jan-18	EPI_ISL_301398	A/Northern Ireland/3483/2018	Regional Virus Laboratory, Microbiology Department, Royal Victoria Hospital	Crick Worldwide Influenza Centre	
EPI1188424	HA	United Kingdom	2018-Jan-17	EPI_ISL_301397	A/Northern Ireland/3411/2018	Regional Virus Laboratory, Microbiology Department, Royal Victoria Hospital	Crick Worldwide Influenza Centre	
EPI1179666	HA	United Kingdom	2017-Dec-05	EPI_ISL_299729	A/Port Talbot/3763/2017	Molecular Diagnostics Unit	Public Health Wales Microbiology Cardiff	
EPI1170171	HA	United Kingdom	2018-Jan-01	EPI_ISL_297462	A/Cardiff/7109/2018	Molecular Diagnostics Unit	Public Health Wales Microbiology Cardiff	
EPI1152059	HA	United Kingdom	2017-Dec-20	EPI_ISL_293630	A/England/120/2017	UK Health Security Agency - Colindale	UK Health Security Agency - Colindale	Galiano,M.
EPI1152019	HA	United Kingdom	2017-Dec-18	EPI_ISL_293625	A/England/75160359/2017	UK Health Security Agency - Colindale	UK Health Security Agency - Colindale	Galiano,M.
EPI1152003	HA	United Kingdom	2017-Dec-19	EPI_ISL_293623	A/England/75160354/2017	UK Health Security Agency - Colindale	UK Health Security Agency - Colindale	Galiano,M.

Segment ID	Segment	Country	Collection date	Isolate-ID	Isolate name	Originating Lab	Submitting Lab	Authors
EPI1151979	HA	United Kingdom	2017-Dec-13	EPI_ISL_293620	A/England/75100510/2017	UK Health Security Agency - Colindale	UK Health Security Agency - Colindale	Galiano,M.
EPI1151955	HA	United Kingdom	2017-Nov-17	EPI_ISL_293617	A/England/74680288/2017	UK Health Security Agency - Colindale	UK Health Security Agency - Colindale	Galiano,M.
EPI1148181	HA	United Kingdom	2017-Nov-15	EPI_ISL_292899	A/England/74700490/2017	UK Health Security Agency - Colindale	Crick Worldwide Influenza Centre	
EPI1148179	HA	United Kingdom	2017-Nov-08	EPI_ISL_292898	A/England/74600618/2017	UK Health Security Agency - Colindale	Crick Worldwide Influenza Centre	
EPI1148177	HA	United Kingdom	2017-Nov-06	EPI_ISL_292897	A/England/74560298/2017	UK Health Security Agency - Colindale	Crick Worldwide Influenza Centre	
EPI1148175	HA	United Kingdom	2017-Oct-21	EPI_ISL_292896	A/England/74400479/2017	UK Health Security Agency - Colindale	Crick Worldwide Influenza Centre	
EPI1148173	HA	United Kingdom	2017-Oct-25	EPI_ISL_292895	A/England/74380294/2017	UK Health Security Agency - Colindale	Crick Worldwide Influenza Centre	
EPI1148171	HA	United Kingdom	2017-Oct-17	EPI_ISL_292894	A/England/74320429/2017	UK Health Security Agency - Colindale	Crick Worldwide Influenza Centre	
EPI1148169	HA	United Kingdom	2017-Sep-24	EPI_ISL_292893	A/England/74000497/2017	UK Health Security Agency - Colindale	Crick Worldwide Influenza Centre	
EPI1139681	HA	United Kingdom	2017-Dec-10	EPI_ISL_291096	A/England/75020489/2017	UK Health Security Agency - Colindale	UK Health Security Agency - Colindale	Galiano,M.
EPI1139679	HA	United Kingdom	2017-Dec-06	EPI_ISL_291095	A/England/74980355/2017	UK Health Security Agency - Colindale	UK Health Security Agency - Colindale	Galiano,M.
EPI1139578	HA	United Kingdom	2017-Dec-11	EPI_ISL_291063	A/England/75120448/2017	UK Health Security Agency - Colindale	UK Health Security Agency - Colindale	Galiano,M.
EPI1139546	HA	United Kingdom	2017-Dec-12	EPI_ISL_291059	A/England/75100262/2017	UK Health Security Agency - Colindale	UK Health Security Agency - Colindale	Galiano,M.
EPI1139530	HA	United Kingdom	2017-Dec-12	EPI_ISL_291057	A/England/75080322/2017	UK Health Security Agency - Colindale	UK Health Security Agency - Colindale	Galiano,M.
EPI1139506	HA	United Kingdom	2017-Dec-11	EPI_ISL_291054	A/England/75040590/2017	UK Health Security Agency - Colindale	UK Health Security Agency - Colindale	Galiano,M.

Segment ID	Segment	Country	Collection date	Isolate-ID	Isolate name	Originating Lab	Submitting Lab	Authors
EPI1139490	HA	United Kingdom	2017-Dec-12	EPI_ISL_291052	A/England/75020484/2017	UK Health Security Agency - Colindale	UK Health Security Agency - Colindale	Galiano,M.
EPI1139466	HA	United Kingdom	2017-Dec-04	EPI_ISL_291049	A/England/75000578/2017	UK Health Security Agency - Colindale	UK Health Security Agency - Colindale	Galiano,M.
EPI1139379	HA	United Kingdom	2017-Nov-30	EPI_ISL_291038	A/England/74940548/2017	UK Health Security Agency - Colindale	UK Health Security Agency - Colindale	Galiano,M.
EPI1139347	HA	United Kingdom	2017-Nov-29	EPI_ISL_291034	A/England/74920384/2017	UK Health Security Agency - Colindale	UK Health Security Agency - Colindale	Galiano,M.
EPI1139339	HA	United Kingdom	2017-Nov-29	EPI_ISL_291033	A/England/74920383/2017	UK Health Security Agency - Colindale	UK Health Security Agency - Colindale	Galiano,M.
EPI1139291	HA	United Kingdom	2017-Nov-22	EPI_ISL_291027	A/England/74840579/2017	UK Health Security Agency - Colindale	UK Health Security Agency - Colindale	Galiano,M.
EPI1139275	HA	United Kingdom	2017-Nov-28	EPI_ISL_291025	A/England/74820686/2017	UK Health Security Agency - Colindale	UK Health Security Agency - Colindale	Galiano,M.
EPI1139219	HA	United Kingdom	2017-Nov-05	EPI_ISL_291018	A/England/74760563/2017	UK Health Security Agency - Colindale	UK Health Security Agency - Colindale	Galiano,M.
EPI1139195	HA	United Kingdom	2017-Nov-15	EPI_ISL_291015	A/England/74700490/2017	UK Health Security Agency - Colindale	UK Health Security Agency - Colindale	Galiano,M.
EPI1139155	HA	United Kingdom	2017-Nov-14	EPI_ISL_291010	A/England/74680316/2017	UK Health Security Agency - Colindale	UK Health Security Agency - Colindale	Galiano,M.
EPI1139147	HA	United Kingdom	2017-Nov-14	EPI_ISL_291009	A/England/74680310/2017	UK Health Security Agency - Colindale	UK Health Security Agency - Colindale	Galiano,M.
EPI1139139	HA	United Kingdom	2017-Nov-13	EPI_ISL_291008	A/England/74680307/2017	UK Health Security Agency - Colindale	UK Health Security Agency - Colindale	Galiano,M.
EPI1139131	HA	United Kingdom	2017-Nov-08	EPI_ISL_291007	A/England/74660585/2017	UK Health Security Agency - Colindale	UK Health Security Agency - Colindale	Galiano,M.

Segment ID	Segment	Country	Collection date	Isolate-ID	Isolate name	Originating Lab	Submitting Lab	Authors
EPI1139123	HA	United Kingdom	2017-Nov-13	EPI_ISL_291006	A/England/74660583/2017	UK Health Security Agency - Colindale	UK Health Security Agency - Colindale	Galiano,M.
EPI1139107	HA	United Kingdom	2017-Nov-10	EPI_ISL_291004	A/England/74640395/2017	UK Health Security Agency - Colindale	UK Health Security Agency - Colindale	Galiano,M.
EPI1139091	HA	United Kingdom	2017-Nov-03	EPI_ISL_291002	A/England/74620551/2017	UK Health Security Agency - Colindale	UK Health Security Agency - Colindale	Galiano,M.
EPI1139083	HA	United Kingdom	2017-Nov-06	EPI_ISL_291001	A/England/74620550/2017	UK Health Security Agency - Colindale	UK Health Security Agency - Colindale	Galiano,M.
EPI1139075	HA	United Kingdom	2017-Nov-03	EPI_ISL_291000	A/England/74620549/2017	UK Health Security Agency - Colindale	UK Health Security Agency - Colindale	Galiano,M.
EPI1139067	HA	United Kingdom	2017-Nov-08	EPI_ISL_290999	A/England/74600617/2017	UK Health Security Agency - Colindale	UK Health Security Agency - Colindale	Galiano,M.
EPI1139059	HA	United Kingdom	2017-Nov-01	EPI_ISL_290998	A/England/74500548/2017	UK Health Security Agency - Colindale	UK Health Security Agency - Colindale	Galiano,M.
EPI1139043	HA	United Kingdom	2017-Oct-21	EPI_ISL_290996	A/England/74400479/2017	UK Health Security Agency - Colindale	UK Health Security Agency - Colindale	Galiano,M.
EPI1139035	HA	United Kingdom	2017-Sep-24	EPI_ISL_290995	A/England/74000492/2017	UK Health Security Agency - Colindale	UK Health Security Agency - Colindale	Galiano,M.
EPI1139019	HA	United Kingdom	2017-Aug-15	EPI_ISL_290993	A/England/73380206/2017	UK Health Security Agency - Colindale	UK Health Security Agency - Colindale	Galiano,M.
EPI1112802	HA	United Kingdom	2017-Sep-30	EPI_ISL_286917	A/England/74100567/2017	UK Health Security Agency - Colindale	UK Health Security Agency - Colindale	Galiano,M.
EPI1112800	HA	United Kingdom	2017-Feb-23	EPI_ISL_286916	A/England/74000490/2017	UK Health Security Agency - Colindale	UK Health Security Agency - Colindale	Galiano,M.
EPI1112794	HA	United Kingdom	2017-Sep-17	EPI_ISL_286913	A/England/73880605/2017	UK Health Security Agency - Colindale	UK Health Security Agency - Colindale	Galiano,M.

Segment ID	Segment	Country	Collection date	Isolate-ID	Isolate name	Originating Lab	Submitting Lab	Authors
EPI1112788	HA	United Kingdom	2017-Aug-25	EPI_ISL_286910	A/England/73660229/2017	UK Health Security Agency - Colindale	UK Health Security Agency - Colindale	Galiano,M.
EPI1112784	HA	United Kingdom	2017-Aug-18	EPI_ISL_286908	A/England/73520179/2017	UK Health Security Agency - Colindale	UK Health Security Agency - Colindale	Galiano,M.
EPI1112769	HA	United Kingdom	2017-Jul-26	EPI_ISL_286900	A/England/73040630/2017	UK Health Security Agency - Colindale	UK Health Security Agency - Colindale	Galiano,M.
EPI1112767	HA	United Kingdom	2017-Jul-18	EPI_ISL_286899	A/England/72960597/2017	UK Health Security Agency - Colindale	UK Health Security Agency - Colindale	Galiano,M.
EPI1112757	HA	United Kingdom	2017-Apr-23	EPI_ISL_286894	A/England/72120694/2017	UK Health Security Agency - Colindale	UK Health Security Agency - Colindale	Galiano,M.
EPI1112730	HA	United Kingdom	2017-Mar-16	EPI_ISL_286880	A/England/71200236/2017	UK Health Security Agency - Colindale	UK Health Security Agency - Colindale	Galiano,M.
EPI1112728	HA	United Kingdom	2017-Feb-16	EPI_ISL_286879	A/England/70800154/2017	UK Health Security Agency - Colindale	UK Health Security Agency - Colindale	Galiano,M.
EPI1112726	HA	United Kingdom	2017-Jan-20	EPI_ISL_286878	A/England/70540345/2017	UK Health Security Agency - Colindale	UK Health Security Agency - Colindale	Galiano,M.
EPI1112692	HA	United Kingdom	2017-Nov-08	EPI_ISL_286873	A/England/74600618/2017	UK Health Security Agency - Colindale	UK Health Security Agency - Colindale	Galiano,M.
EPI1112676	HA	United Kingdom	2017-Nov-06	EPI_ISL_286871	A/England/74560298/2017	UK Health Security Agency - Colindale	UK Health Security Agency - Colindale	Galiano,M.
EPI1112652	HA	United Kingdom	2017-Oct-22	EPI_ISL_286868	A/England/74480500/2017	UK Health Security Agency - Colindale	UK Health Security Agency - Colindale	Galiano,M.
EPI1112644	HA	United Kingdom	2017-Oct-19	EPI_ISL_286867	A/England/74400480/2017	UK Health Security Agency - Colindale	UK Health Security Agency - Colindale	Galiano,M.
EPI1112636	HA	United Kingdom	2017-Oct-25	EPI_ISL_286866	A/England/74380294/2017	UK Health Security Agency - Colindale	UK Health Security Agency - Colindale	Galiano,M.

Segment ID	Segment	Country	Collection date	Isolate-ID	Isolate name	Originating Lab	Submitting Lab	Authors
EPI1112628	HA	United Kingdom	2017-Oct-19	EPI_ISL_286865	A/England/74340641/2017	UK Health Security Agency - Colindale	UK Health Security Agency - Colindale	Galiano,M.
EPI1112620	HA	United Kingdom	2017-Oct-18	EPI_ISL_286864	A/England/74340637/2017	UK Health Security Agency - Colindale	UK Health Security Agency - Colindale	Galiano,M.
EPI1112612	HA	United Kingdom	2017-Oct-17	EPI_ISL_286863	A/England/74320429/2017	UK Health Security Agency - Colindale	UK Health Security Agency - Colindale	Galiano,M.
EPI1112604	HA	United Kingdom	2017-Oct-17	EPI_ISL_286862	A/England/74320426/2017	UK Health Security Agency - Colindale	UK Health Security Agency - Colindale	Galiano,M.
EPI1112596	HA	United Kingdom	2017-Oct-17	EPI_ISL_286861	A/England/74300448/2017	UK Health Security Agency - Colindale	UK Health Security Agency - Colindale	Galiano,M.
EPI1112588	HA	United Kingdom	2017-Oct-16	EPI_ISL_286860	A/England/74300447/2017	UK Health Security Agency - Colindale	UK Health Security Agency - Colindale	Galiano,M.
EPI1112564	HA	United Kingdom	2017-Oct-16	EPI_ISL_286857	A/England/74240422/2017	UK Health Security Agency - Colindale	UK Health Security Agency - Colindale	Galiano,M.
EPI1112556	HA	United Kingdom	2017-Oct-05	EPI_ISL_286856	A/England/74160653/2017	UK Health Security Agency - Colindale	UK Health Security Agency - Colindale	Galiano,M.
EPI1112548	HA	United Kingdom	2017-Oct-03	EPI_ISL_286855	A/England/74100569/2017	UK Health Security Agency - Colindale	UK Health Security Agency - Colindale	Galiano,M.
EPI1112532	HA	United Kingdom	2017-Oct-06	EPI_ISL_286853	A/England/74080448/2017	UK Health Security Agency - Colindale	UK Health Security Agency - Colindale	Galiano,M.
EPI1112524	HA	United Kingdom	2017-Oct-06	EPI_ISL_286852	A/England/74080447/2017	UK Health Security Agency - Colindale	UK Health Security Agency - Colindale	Galiano,M.
EPI1112500	HA	United Kingdom	2017-Sep-24	EPI_ISL_286849	A/England/74000497/2017	UK Health Security Agency - Colindale	UK Health Security Agency - Colindale	Galiano,M.
EPI1112484	HA	United Kingdom	2017-Sep-07	EPI_ISL_286847	A/England/73880604/2017	UK Health Security Agency - Colindale	UK Health Security Agency - Colindale	Galiano,M.

Segment ID	Segment	Country	Collection date	Isolate-ID	Isolate name	Originating Lab	Submitting Lab	Authors
EPI1112476	HA	United Kingdom	2017-Sep-12	EPI_ISL_286846	A/England/73880601/2017	UK Health Security Agency - Colindale	UK Health Security Agency - Colindale	Galiano,M.
EPI1112460	HA	United Kingdom	2017-Sep-04	EPI_ISL_286844	A/England/73660232/2017	UK Health Security Agency - Colindale	UK Health Security Agency - Colindale	Galiano,M.
EPI1112452	HA	United Kingdom	2017-Aug-01	EPI_ISL_286843	A/England/73660230/2017	UK Health Security Agency - Colindale	UK Health Security Agency - Colindale	Galiano,M.
EPI1112444	HA	United Kingdom	2017-Aug-30	EPI_ISL_286842	A/England/73580521/2017	UK Health Security Agency - Colindale	UK Health Security Agency - Colindale	Galiano,M.
EPI1112436	HA	United Kingdom	2017-Aug-28	EPI_ISL_286841	A/England/73560808/2017	UK Health Security Agency - Colindale	UK Health Security Agency - Colindale	Galiano,M.
EPI1112428	HA	United Kingdom	2017-Aug-19	EPI_ISL_286840	A/England/73520490/2017	UK Health Security Agency - Colindale	UK Health Security Agency - Colindale	Galiano,M.
EPI1112420	HA	United Kingdom	2017-Aug-16	EPI_ISL_286839	A/England/73520177/2017	UK Health Security Agency - Colindale	UK Health Security Agency - Colindale	Galiano,M.
EPI1112412	HA	United Kingdom	2017-Aug-16	EPI_ISL_286838	A/England/73520176/2017	UK Health Security Agency - Colindale	UK Health Security Agency - Colindale	Galiano,M.
EPI1112404	HA	United Kingdom	2017-Aug-16	EPI_ISL_286837	A/England/73520174/2017	UK Health Security Agency - Colindale	UK Health Security Agency - Colindale	Galiano,M.
EPI1112396	HA	United Kingdom	2017-Aug-13	EPI_ISL_286836	A/England/73420564/2017	UK Health Security Agency - Colindale	UK Health Security Agency - Colindale	Galiano,M.
EPI1112348	HA	United Kingdom	2017-Aug-05	EPI_ISL_286830	A/England/73300573/2017	UK Health Security Agency - Colindale	UK Health Security Agency - Colindale	Galiano,M.
EPI1112340	HA	United Kingdom	2017-Jan-15	EPI_ISL_286829	A/England/73160663/2017	UK Health Security Agency - Colindale	UK Health Security Agency - Colindale	Galiano,M.
EPI1112332	HA	United Kingdom	2017-Jan-14	EPI_ISL_286828	A/England/73160662/2017	UK Health Security Agency - Colindale	UK Health Security Agency - Colindale	Galiano,M.

Segment ID	Segment	Country	Collection date	Isolate-ID	Isolate name	Originating Lab	Submitting Lab	Authors
EPI1112324	HA	United Kingdom	2017-Jan-15	EPI_ISL_286827	A/England/73160661/2017	UK Health Security Agency - Colindale	UK Health Security Agency - Colindale	Galiano,M.
EPI1112316	HA	United Kingdom	2017-Jan-12	EPI_ISL_286826	A/England/73160660/2017	UK Health Security Agency - Colindale	UK Health Security Agency - Colindale	Galiano,M.
EPI1112308	HA	United Kingdom	2017-Aug-02	EPI_ISL_286825	A/England/73160333/2017	UK Health Security Agency - Colindale	UK Health Security Agency - Colindale	Galiano,M.
EPI1112292	HA	United Kingdom	2017-Jul-19	EPI_ISL_286823	A/England/72980505/2017	UK Health Security Agency - Colindale	UK Health Security Agency - Colindale	Galiano,M.
EPI1112284	HA	United Kingdom	2017-Jul-04	EPI_ISL_286822	A/England/72840639/2017	UK Health Security Agency - Colindale	UK Health Security Agency - Colindale	Galiano,M.
EPI1112276	HA	United Kingdom	2017-Jul-06	EPI_ISL_286821	A/England/72820665/2017	UK Health Security Agency - Colindale	UK Health Security Agency - Colindale	Galiano,M.
EPI1112268	HA	United Kingdom	2017-Jun-21	EPI_ISL_286820	A/England/72720745/2017	UK Health Security Agency - Colindale	UK Health Security Agency - Colindale	Galiano,M.
EPI1112260	HA	United Kingdom	2017-Mar-01	EPI_ISL_286819	A/England/72520366/2017	UK Health Security Agency - Colindale	UK Health Security Agency - Colindale	Galiano,M.
EPI1112252	HA	United Kingdom	2017-Mar-01	EPI_ISL_286818	A/England/72520364/2017	UK Health Security Agency - Colindale	UK Health Security Agency - Colindale	Galiano,M.
EPI1112244	HA	United Kingdom	2017-Jun-01	EPI_ISL_286817	A/England/72480621/2017	UK Health Security Agency - Colindale	UK Health Security Agency - Colindale	Galiano,M.
EPI1112236	HA	United Kingdom	2017-Jun-10	EPI_ISL_286816	A/England/72420554/2017	UK Health Security Agency - Colindale	UK Health Security Agency - Colindale	Galiano,M.
EPI1112148	HA	United Kingdom	2017-Apr-28	EPI_ISL_286805	A/England/71900671/2017	UK Health Security Agency - Colindale	UK Health Security Agency - Colindale	Galiano,M.
EPI1112108	HA	United Kingdom	2017-Apr-04	EPI_ISL_286800	A/England/71480647/2017	UK Health Security Agency - Colindale	UK Health Security Agency - Colindale	Galiano,M.

Segment ID	Segment	Country	Collection date	Isolate-ID	Isolate name	Originating Lab	Submitting Lab	Authors
EPI1112100	HA	United Kingdom	2017-Jan-29	EPI_ISL_286799	A/England/71480645/2017	UK Health Security Agency - Colindale	UK Health Security Agency - Colindale	Galiano,M.
EPI1112068	HA	United Kingdom	2017-Feb-16	EPI_ISL_286795	A/England/70800156/2017	UK Health Security Agency - Colindale	UK Health Security Agency - Colindale	Galiano,M.
EPI1112060	HA	United Kingdom	2017-Feb-16	EPI_ISL_286794	A/England/70800155/2017	UK Health Security Agency - Colindale	UK Health Security Agency - Colindale	Galiano,M.
EPI1112052	HA	United Kingdom	2017-Jan-20	EPI_ISL_286793	A/England/70540344/2017	UK Health Security Agency - Colindale	UK Health Security Agency - Colindale	Galiano,M.
EPI1112044	HA	United Kingdom	2017-Jan-25	EPI_ISL_286792	A/England/70480157/2017	UK Health Security Agency - Colindale	UK Health Security Agency - Colindale	Galiano,M.
EPI1112036	HA	United Kingdom	2017-Jan-25	EPI_ISL_286791	A/England/70480156/2017	UK Health Security Agency - Colindale	UK Health Security Agency - Colindale	Galiano,M.
EPI1112028	HA	United Kingdom	2017-Jan-25	EPI_ISL_286790	A/England/70480155/2017	UK Health Security Agency - Colindale	UK Health Security Agency - Colindale	Galiano,M.
EPI1112020	HA	United Kingdom	2017-Jan-24	EPI_ISL_286789	A/England/70460001/2017	UK Health Security Agency - Colindale	UK Health Security Agency - Colindale	Galiano,M.
EPI1112012	HA	United Kingdom	2017-Jan-23	EPI_ISL_286788	A/England/70440238/2017	UK Health Security Agency - Colindale	UK Health Security Agency - Colindale	Galiano,M.
EPI1112004	HA	United Kingdom	2017-Jan-23	EPI_ISL_286787	A/England/70420060/2017	UK Health Security Agency - Colindale	UK Health Security Agency - Colindale	Galiano,M.
EPI1045294	HA	United Kingdom	2016-Dec-31	EPI_ISL_275187	A/London/66U016982-49_S1_L001/2016	Department of Clinical Virology, University College London Hospitals NHS Foundation Trust	University College London	
EPI1045302	HA	United Kingdom	2016-Dec-31	EPI_ISL_275186	A/London/66U016973-81_S33_L001/2016	Department of Clinical Virology, University College	University College London	

Segment ID	Segment	Country	Collection date	Isolate-ID	Isolate name	Originating Lab	Submitting Lab	Authors
						London Hospitals NHS Foundation Trust		
EPI1045747	HA	United Kingdom	2016-Dec-31	EPI_ISL_275183	A/London/66U016964-82_S34_L001/2016	Department of Clinical Virology, University College London Hospitals NHS Foundation Trust	University College London	
EPI1045286	HA	United Kingdom	2016-Dec-30	EPI_ISL_275182	A/London/66U016884-44_S44_L001/2016	Department of Clinical Virology, University College London Hospitals NHS Foundation Trust	University College London	
EPI1045278	HA	United Kingdom	2016-Dec-30	EPI_ISL_275181	A/London/66U016814-39_S39_L001/2016	Department of Clinical Virology, University College London Hospitals NHS Foundation Trust	University College London	
EPI1045739	HA	United Kingdom	2016-Dec-30	EPI_ISL_275179	A/London/66U016700-83_S35_L001/2016	Department of Clinical Virology, University College London Hospitals NHS Foundation Trust	University College London	
EPI1045758	HA	United Kingdom	2016-Dec-30	EPI_ISL_275177	A/London/66U011435-45_S45_L001/2016	Department of Clinical Virology, University College London Hospitals NHS Foundation Trust	University College London	
EPI1045769	HA	United Kingdom	2016-Dec-30	EPI_ISL_274890	A/London/66U011389-40_S40_L001/2106	Department of Clinical Virology, University College London Hospitals NHS Foundation Trust	University College London	

Segment ID	Segment	Country	Collection date	Isolate-ID	Isolate name	Originating Lab	Submitting Lab	Authors
EPI1045780	HA	United Kingdom	2016-Dec-29	EPI_ISL_274887	A/London/66U011102-32_S32_L001/2016	Department of Clinical Virology, University College London Hospitals NHS Foundation Trust	University College London	
EPI1046115	HA	United Kingdom	2016-Dec-28	EPI_ISL_274886	A/London/66U011084-35_S35_L001/2016	Department of Clinical Virology, University College London Hospitals NHS Foundation Trust	University College London	
EPI1045718	HA	United Kingdom	2016-Dec-28	EPI_ISL_274885	A/London/66U011082-36_S36_L001/2016	Department of Clinical Virology, University College London Hospitals NHS Foundation Trust	University College London	
EPI1045788	HA	United Kingdom	2017-Jan-02	EPI_ISL_274884	A/London/17U056107-57_S9_L001/2017	Department of Clinical Virology, University College London Hospitals NHS Foundation Trust	University College London	
EPI1046173	HA	United Kingdom	2017-Jan-02	EPI_ISL_274883	A/London/17U056100-55_S7_L001/2017	Department of Clinical Virology, University College London Hospitals NHS Foundation Trust	University College London	
EPI1046375	HA	United Kingdom	2017-Jan-02	EPI_ISL_274882	A/London/17U056098-54_S6_L001/2017	Department of Clinical Virology, University College London Hospitals NHS Foundation Trust	University College London	
EPI1046466	HA	United Kingdom	2017-Jan-02	EPI_ISL_274881	A/London/17U056092-53_S5_L001/2017	Department of Clinical Virology, University College	University College London	

Segment ID	Segment	Country	Collection date	Isolate-ID	Isolate name	Originating Lab	Submitting Lab	Authors
						London Hospitals NHS Foundation Trust		
EPI1046491	HA	United Kingdom	2017-Jan-02	EPI_ISL_274880	A/London/17U056088-52_S4_L001/2017	Department of Clinical Virology, University College London Hospitals NHS Foundation Trust	University College London	
EPI1046499	HA	United Kingdom	2017-Jan-02	EPI_ISL_274879	A/London/17U056084-51_S3_L001/	Department of Clinical Virology, University College London Hospitals NHS Foundation Trust	University College London	
EPI1046507	HA	United Kingdom	2017-Jan-02	EPI_ISL_274878	A/London/17U056080-25_S25_L001/2017	Department of Clinical Virology, University College London Hospitals NHS Foundation Trust	University College London	
EPI1046515	HA	United Kingdom	2017-Jan-03	EPI_ISL_274877	A/London/17U018045-58_S10_L001/2017	Department of Clinical Virology, University College London Hospitals NHS Foundation Trust	University College London	
EPI1046523	HA	United Kingdom	2016-Dec-16	EPI_ISL_274876	A/London/16U993945-75_S27_L001/2016	Department of Clinical Virology, University College London Hospitals NHS Foundation Trust	University College London	
EPI1046531	HA	United Kingdom	2016-Dec-15	EPI_ISL_274875	A/London/16U993610-74_S26_L001/2016	Department of Clinical Virology, University College London Hospitals NHS Foundation Trust	University College London	

Segment ID	Segment	Country	Collection date	Isolate-ID	Isolate name	Originating Lab	Submitting Lab	Authors
EPI1045726	HA	United Kingdom	2016-Dec-02	EPI_ISL_274874	A/London/16U952757-61_S13_L001/2016	Department of Clinical Virology, University College London Hospitals NHS Foundation Trust	University College London	
EPI1046539	HA	United Kingdom	2016-Dec-02	EPI_ISL_274873	A/London/16U952692-60_S12_L001/2016	Department of Clinical Virology, University College London Hospitals NHS Foundation Trust	University College London	
EPI1046547	HA	United Kingdom	2016-Dec-02	EPI_ISL_274872	A/London/16U952691-59_S11_L001/2016	Department of Clinical Virology, University College London Hospitals NHS Foundation Trust	University College London	
EPI1046555	HA	United Kingdom	2016-Dec-06	EPI_ISL_274871	A/London/16U951427-62_S14_L001/2016	Department of Clinical Virology, University College London Hospitals NHS Foundation Trust	University College London	
EPI1047422	HA	United Kingdom	2016-Dec-07	EPI_ISL_274843	A/London/16U949972-67_S19_L001/2016	Department of Clinical Virology, University College London Hospitals NHS Foundation Trust	University College London	
EPI1044433	HA	United Kingdom	2016-Dec-09	EPI_ISL_274842	A/London/16U948511-69_S21_L001/2016	Department of Clinical Virology, University College London Hospitals NHS Foundation Trust	University College London	
EPI1047406	HA	United Kingdom	2016-Dec-09	EPI_ISL_274841	A/London/16U948347-65_S17_L001/2016	Department of Clinical Virology, University College	University College London	

Segment ID	Segment	Country	Collection date	Isolate-ID	Isolate name	Originating Lab	Submitting Lab	Authors
						London Hospitals NHS Foundation Trust		
EPI1047414	HA	United Kingdom	2016-Dec-09	EPI_ISL_274840	A/London/16U948347-63_S15_L001/2016	Department of Clinical Virology, University College London Hospitals NHS Foundation Trust	University College London	
EPI1047444	HA	United Kingdom	2016-Nov-29	EPI_ISL_274837	A/London/16U938970-84_S36_L001/2016	Department of Clinical Virology, University College London Hospitals NHS Foundation Trust	University College London	
EPI1047452	HA	United Kingdom	2016-Nov-29	EPI_ISL_274836	A/London/16U938930-85_S37_L001/2016	Department of Clinical Virology, University College London Hospitals NHS Foundation Trust	University College London	
EPI1047464	HA	United Kingdom	2016-Dec-13	EPI_ISL_274835	A/London/16U932934-71_S23_L001/2016	Department of Clinical Virology, University College London Hospitals NHS Foundation Trust	University College London	
EPI1047522	HA	United Kingdom	2016-Dec-09	EPI_ISL_274834	A/London/16U932220-68_S20_L001/2016	Department of Clinical Virology, University College London Hospitals NHS Foundation Trust	University College London	
EPI1047535	HA	United Kingdom	2016-Dec-15	EPI_ISL_274833	A/London/16U928902-73_S25_L001/2016	Department of Clinical Virology, University College London Hospitals NHS Foundation Trust	University College London	

Segment ID	Segment	Country	Collection date	Isolate-ID	Isolate name	Originating Lab	Submitting Lab	Authors
EPI1047871	HA	United Kingdom	2016-Dec-14	EPI_ISL_274832	A/London/16U928822-72_S24_L001/2016	Department of Clinical Virology, University College London Hospitals NHS Foundation Trust	University College London	
EPI1047694	HA	United Kingdom	2016-Dec-13	EPI_ISL_274831	A/London/16U928199-70_S22_L001/2016	Department of Clinical Virology, University College London Hospitals NHS Foundation Trust	University College London	
EPI1047583	HA	United Kingdom	2016-Dec-27	EPI_ISL_274830	A/London/16U848977-80_S32_L001/2016	Department of Clinical Virology, University College London Hospitals NHS Foundation Trust	University College London	
EPI1047543	HA	United Kingdom	2016-Dec-19	EPI_ISL_274829	A/London/16U845471-77_S29_L001/2016	Department of Clinical Virology, University College London Hospitals NHS Foundation Trust	University College London	
EPI1044425	HA	United Kingdom	2016-Dec-19	EPI_ISL_274828	A/London/16U845469-76_S28_L001/2016	Department of Clinical Virology, University College London Hospitals NHS Foundation Trust	University College London	
EPI1047510	HA	United Kingdom	2016-Dec-23	EPI_ISL_274811	A/London/16U833659-18_S18_L001/2016	Department of Clinical Virology, University College London Hospitals NHS Foundation Trust	University College London	
EPI1047502	HA	United Kingdom	2016-Dec-23	EPI_ISL_274810	A/London/16U833585-7_S7_L001/2016	Department of Clinical Virology, University College	University College London	

Segment ID	Segment	Country	Collection date	Isolate-ID	Isolate name	Originating Lab	Submitting Lab	Authors
						London Hospitals NHS Foundation Trust		
EPI1047490	HA	United Kingdom	2016-Dec-22	EPI_ISL_274796	A/London/16U833255-2_S2_L001/2016	Department of Clinical Virology, University College London Hospitals NHS Foundation Trust	University College London	
EPI1047474	HA	United Kingdom	2016-Dec-25	EPI_ISL_274658	A/London/16U828964-20_S20_L001/2016	Department of Clinical Virology, University College London Hospitals NHS Foundation Trust	University College London	
EPI1047482	HA	United Kingdom	2016-Dec-25	EPI_ISL_274657	A/London/16U828956-21_S21_L001/2016	Department of Clinical Virology, University College London Hospitals NHS Foundation Trust	University College London	
EPI1045671	HA	United Kingdom	2016-Dec-25	EPI_ISL_274648	A/London/16U828954-22_S22_L001/2016	Department of Clinical Virology, University College London Hospitals NHS Foundation Trust	University College London	
EPI1045663	HA	United Kingdom	2016-Dec-25	EPI_ISL_274647	A/London/16U828953-24_S24_L001/2016	Department of Clinical Virology, University College London Hospitals NHS Foundation Trust	University College London	
EPI1045653	HA	United Kingdom	2016-Dec-24	EPI_ISL_274644	A/London/16U828821-17_S17_L001/2016	Department of Clinical Virology, University College London Hospitals NHS Foundation Trust	University College London	

Segment ID	Segment	Country	Collection date	Isolate-ID	Isolate name	Originating Lab	Submitting Lab	Authors
EPI1045645	HA	United Kingdom	2016-Dec-24	EPI_ISL_274635	A/London/16U828820-16_S16_L001/2016	Department of Clinical Virology, University College London Hospitals NHS Foundation Trust	University College London	
EPI1045638	HA	United Kingdom	2016-Dec-24	EPI_ISL_274633	A/London/16U828819-15_S15_L001/2016	Department of Clinical Virology, University College London Hospitals NHS Foundation Trust	University College London	
EPI1045630	HA	United Kingdom	2016-Dec-24	EPI_ISL_274625	A/London/16U828811-14_S14_L001/2016	Department of Clinical Virology, University College London Hospitals NHS Foundation Trust	University College London	
EPI1043410	HA	United Kingdom	2016-Dec-24	EPI_ISL_274435	A/London/16U828794-12_S12_L001/2016	Department of Clinical Virology, University College London Hospitals NHS Foundation Trust	University College London	
EPI1042518	HA	United Kingdom	2016-Dec-23	EPI_ISL_274434	A/London/16U828704-11_S11_L001/2016	Department of Clinical Virology, University College London Hospitals NHS Foundation Trust	University College London	
EPI1030593	HA	United Kingdom	2016-Oct-21	EPI_ISL_270919	A/England/64340119/2016	UK Health Security Agency - Colindale	UK Health Security Agency - Colindale	Galiano,M.
EPI1030474	HA	United Kingdom	2017-Mar-03	EPI_ISL_270814	A/England/71040520/2017	UK Health Security Agency - Colindale	UK Health Security Agency - Colindale	Galiano,M.
EPI1030472	HA	United Kingdom	2017-Mar-02	EPI_ISL_270813	A/England/71040516/2017	UK Health Security Agency - Colindale	UK Health Security Agency - Colindale	Galiano,M.

Segment ID	Segment	Country	Collection date	Isolate-ID	Isolate name	Originating Lab	Submitting Lab	Authors
EPI1030469	HA	United Kingdom	2017-Feb-15	EPI_ISL_270811	A/England/70840521/2017	UK Health Security Agency - Colindale	UK Health Security Agency - Colindale	Galiano,M.
EPI1030467	HA	United Kingdom	2017-Feb-20	EPI_ISL_270810	A/England/70840272/2017	UK Health Security Agency - Colindale	UK Health Security Agency - Colindale	Galiano,M.
EPI1030465	HA	United Kingdom	2017-Feb-14	EPI_ISL_270809	A/England/70740140/2017	UK Health Security Agency - Colindale	UK Health Security Agency - Colindale	Galiano,M.
EPI1030463	HA	United Kingdom	2017-Feb-10	EPI_ISL_270808	A/England/70740079/2017	UK Health Security Agency - Colindale	UK Health Security Agency - Colindale	Galiano,M.
EPI1030460	HA	United Kingdom	2017-Feb-03	EPI_ISL_270806	A/England/70680476/2017	UK Health Security Agency - Colindale	UK Health Security Agency - Colindale	Galiano,M.
EPI1030458	HA	United Kingdom	2017-Feb-06	EPI_ISL_270805	A/England/70660208/2017	UK Health Security Agency - Colindale	UK Health Security Agency - Colindale	Galiano,M.
EPI1030456	HA	United Kingdom	2017-Feb-01	EPI_ISL_270804	A/England/70600071/2017	UK Health Security Agency - Colindale	UK Health Security Agency - Colindale	Galiano,M.
EPI1030454	HA	United Kingdom	2017-Feb-01	EPI_ISL_270803	A/England/70540222/2017	UK Health Security Agency - Colindale	UK Health Security Agency - Colindale	Galiano,M.
EPI1030452	HA	United Kingdom	2017-Jan-31	EPI_ISL_270802	A/England/70520315/2017	UK Health Security Agency - Colindale	UK Health Security Agency - Colindale	Galiano,M.
EPI1030450	HA	United Kingdom	2017-Jan-03	EPI_ISL_270801	A/England/70520313/2017	UK Health Security Agency - Colindale	UK Health Security Agency - Colindale	Galiano,M.
EPI1030448	HA	United Kingdom	2017-Jan-30	EPI_ISL_270800	A/England/70520081/2017	UK Health Security Agency - Colindale	UK Health Security Agency - Colindale	Galiano,M.
EPI1030446	HA	United Kingdom	2017-Jan-30	EPI_ISL_270799	A/England/70520077/2017	UK Health Security Agency - Colindale	UK Health Security Agency - Colindale	Galiano,M.
EPI1030444	HA	United Kingdom	2017-Jan-26	EPI_ISL_270798	A/England/70500066/2017	UK Health Security Agency - Colindale	UK Health Security Agency - Colindale	Galiano,M.

Segment ID	Segment	Country	Collection date	Isolate-ID	Isolate name	Originating Lab	Submitting Lab	Authors
EPI1030442	HA	United Kingdom	2017-Jan-25	EPI_ISL_270797	A/England/70480142/2017	UK Health Security Agency - Colindale	UK Health Security Agency - Colindale	Galiano,M.
EPI1030440	HA	United Kingdom	2017-Jan-19	EPI_ISL_270796	A/England/70460008/2017	UK Health Security Agency - Colindale	UK Health Security Agency - Colindale	Galiano,M.
EPI1030438	HA	United Kingdom	2017-Jan-24	EPI_ISL_270795	A/England/70420067/2017	UK Health Security Agency - Colindale	UK Health Security Agency - Colindale	Galiano,M.
EPI1030436	HA	United Kingdom	2017-Jan-14	EPI_ISL_270794	A/England/70400233/2017	UK Health Security Agency - Colindale	UK Health Security Agency - Colindale	Galiano,M.
EPI1030434	HA	United Kingdom	2017-Jan-19	EPI_ISL_270793	A/England/70400016/2017	UK Health Security Agency - Colindale	UK Health Security Agency - Colindale	Galiano,M.
EPI1030431	HA	United Kingdom	2017-Jan-16	EPI_ISL_270791	A/England/70380304/2017	UK Health Security Agency - Colindale	UK Health Security Agency - Colindale	Galiano,M.
EPI1030429	HA	United Kingdom	2017-Jan-19	EPI_ISL_270790	A/England/70380287/2017	UK Health Security Agency - Colindale	UK Health Security Agency - Colindale	Galiano,M.
EPI1030427	HA	United Kingdom	2017-Jan-19	EPI_ISL_270789	A/England/70380038/2017	UK Health Security Agency - Colindale	UK Health Security Agency - Colindale	Galiano,M.
EPI1030425	HA	United Kingdom	2017-Jan-16	EPI_ISL_270788	A/England/70380024/2017	UK Health Security Agency - Colindale	UK Health Security Agency - Colindale	Galiano,M.
EPI1030423	HA	United Kingdom	2017-Jan-18	EPI_ISL_270787	A/England/70360614/2017	UK Health Security Agency - Colindale	UK Health Security Agency - Colindale	Galiano,M.
EPI1030421	HA	United Kingdom	2017-Jan-17	EPI_ISL_270786	A/England/70360612/2017	UK Health Security Agency - Colindale	UK Health Security Agency - Colindale	Galiano,M.
EPI1030419	HA	United Kingdom	2017-Jan-16	EPI_ISL_270785	A/England/70360601/2017	UK Health Security Agency - Colindale	UK Health Security Agency - Colindale	Galiano,M.
EPI1030417	HA	United Kingdom	2017-Jan-17	EPI_ISL_270784	A/England/70340160/2017	UK Health Security Agency - Colindale	UK Health Security Agency - Colindale	Galiano,M.

Segment ID	Segment	Country	Collection date	Isolate-ID	Isolate name	Originating Lab	Submitting Lab	Authors
EPI1030413	HA	United Kingdom	2017-Jan-16	EPI_ISL_270782	A/England/70300383/2017	UK Health Security Agency - Colindale	UK Health Security Agency - Colindale	Galiano,M.
EPI1030411	HA	United Kingdom	2017-Jan-12	EPI_ISL_270781	A/England/70300023/2017	UK Health Security Agency - Colindale	UK Health Security Agency - Colindale	Galiano,M.
EPI1030409	HA	United Kingdom	2017-Jan-12	EPI_ISL_270780	A/England/70300017/2017	UK Health Security Agency - Colindale	UK Health Security Agency - Colindale	Galiano,M.
EPI1030407	HA	United Kingdom	2017-Jan-11	EPI_ISL_270779	A/England/70260699/2017	UK Health Security Agency - Colindale	UK Health Security Agency - Colindale	Galiano,M.
EPI1030405	HA	United Kingdom	2017-Jan-04	EPI_ISL_270778	A/England/70200659/2017	UK Health Security Agency - Colindale	UK Health Security Agency - Colindale	Galiano,M.
EPI1030403	HA	United Kingdom	2017-Jan-01	EPI_ISL_270777	A/England/70180244/2017	UK Health Security Agency - Colindale	UK Health Security Agency - Colindale	Galiano,M.
EPI1030401	HA	United Kingdom	2017-Jan-03	EPI_ISL_270776	A/England/70180195/2017	UK Health Security Agency - Colindale	UK Health Security Agency - Colindale	Galiano,M.
EPI1030399	HA	United Kingdom	2016-Dec-31	EPI_ISL_270775	A/England/70160954/2016	UK Health Security Agency - Colindale	UK Health Security Agency - Colindale	Galiano,M.
EPI1030397	HA	United Kingdom	2017-Jan-05	EPI_ISL_270774	A/England/70160675/2017	UK Health Security Agency - Colindale	UK Health Security Agency - Colindale	Galiano,M.
EPI1030395	HA	United Kingdom	2016-Dec-29	EPI_ISL_270773	A/England/70160673/2016	UK Health Security Agency - Colindale	UK Health Security Agency - Colindale	Galiano,M.
EPI1030392	HA	United Kingdom	2016-Dec-30	EPI_ISL_270771	A/England/70160047/2016	UK Health Security Agency - Colindale	UK Health Security Agency - Colindale	Galiano,M.
EPI1030390	HA	United Kingdom	2017-Jan-03	EPI_ISL_270770	A/England/70120027/2017	UK Health Security Agency - Colindale	UK Health Security Agency - Colindale	Galiano,M.
EPI1030388	HA	United Kingdom	2017-Jan-03	EPI_ISL_270769	A/England/70120025/2017	UK Health Security Agency - Colindale	UK Health Security Agency - Colindale	Galiano,M.

Segment ID	Segment	Country	Collection date	Isolate-ID	Isolate name	Originating Lab	Submitting Lab	Authors
EPI1030385	HA	United Kingdom	2016-Dec-27	EPI_ISL_270767	A/England/65280702/2016	UK Health Security Agency - Colindale	UK Health Security Agency - Colindale	Galiano,M.
EPI1030383	HA	United Kingdom	2016-Dec-22	EPI_ISL_270766	A/England/65280697/2016	UK Health Security Agency - Colindale	UK Health Security Agency - Colindale	Galiano,M.
EPI1030381	HA	United Kingdom	2016-Dec-22	EPI_ISL_270765	A/England/65280695/2016	UK Health Security Agency - Colindale	UK Health Security Agency - Colindale	Galiano,M.
EPI1030379	HA	United Kingdom	2016-Dec-21	EPI_ISL_270764	A/England/65240024/2016	UK Health Security Agency - Colindale	UK Health Security Agency - Colindale	Galiano,M.
EPI1030377	HA	United Kingdom	2016-Dec-19	EPI_ISL_270763	A/England/65100009/2016	UK Health Security Agency - Colindale	UK Health Security Agency - Colindale	Galiano,M.
EPI1030375	HA	United Kingdom	2016-Dec-13	EPI_ISL_270762	A/England/65060416/2016	UK Health Security Agency - Colindale	UK Health Security Agency - Colindale	Galiano,M.
EPI1030373	HA	United Kingdom	2016-Dec-10	EPI_ISL_270761	A/England/65040829/2016	UK Health Security Agency - Colindale	UK Health Security Agency - Colindale	Galiano,M.
EPI1030371	HA	United Kingdom	2016-Dec-08	EPI_ISL_270760	A/England/65040082/2016	UK Health Security Agency - Colindale	UK Health Security Agency - Colindale	Galiano,M.
EPI1030369	HA	United Kingdom	2016-Dec-12	EPI_ISL_270759	A/England/65040077/2016	UK Health Security Agency - Colindale	UK Health Security Agency - Colindale	Galiano,M.
EPI1030367	HA	United Kingdom	2016-Dec-11	EPI_ISL_270758	A/England/65020629/2016	UK Health Security Agency - Colindale	UK Health Security Agency - Colindale	Galiano,M.
EPI1030365	HA	United Kingdom	2016-Dec-06	EPI_ISL_270757	A/England/65020628/2016	UK Health Security Agency - Colindale	UK Health Security Agency - Colindale	Galiano,M.
EPI1030363	HA	United Kingdom	2016-Dec-12	EPI_ISL_270756	A/England/65020051/2016	UK Health Security Agency - Colindale	UK Health Security Agency - Colindale	Galiano,M.
EPI1030361	HA	United Kingdom	2016-Dec-05	EPI_ISL_270755	A/England/64940740/2016	UK Health Security Agency - Colindale	UK Health Security Agency - Colindale	Galiano,M.

Segment ID	Segment	Country	Collection date	Isolate-ID	Isolate name	Originating Lab	Submitting Lab	Authors
EPI1030359	HA	United Kingdom	2016-Nov-28	EPI_ISL_270754	A/England/64840141/2016	UK Health Security Agency - Colindale	UK Health Security Agency - Colindale	Galiano,M.
EPI1030357	HA	United Kingdom	2016-Nov-19	EPI_ISL_270753	A/England/64760618/2016	UK Health Security Agency - Colindale	UK Health Security Agency - Colindale	Galiano,M.
EPI1030355	HA	United Kingdom	2016-Nov-08	EPI_ISL_270752	A/England/64580216/2016	UK Health Security Agency - Colindale	UK Health Security Agency - Colindale	Galiano,M.
EPI1030353	HA	United Kingdom	2016-Nov-08	EPI_ISL_270751	A/England/64560781/2016	UK Health Security Agency - Colindale	UK Health Security Agency - Colindale	Galiano,M.
EPI1030350	HA	United Kingdom	2016-Oct-24	EPI_ISL_270749	A/England/64360269/2016	UK Health Security Agency - Colindale	UK Health Security Agency - Colindale	Galiano,M.
EPI1029669	HA	United Kingdom	2017-Mar-14	EPI_ISL_270573	A/N.Ireland/71380480/2017	UK Health Security Agency - Colindale	UK Health Security Agency - Colindale	Galiano,M.
EPI1029661	HA	United Kingdom	2017-Mar-06	EPI_ISL_270572	A/N.Ireland/71380470/2017	UK Health Security Agency - Colindale	UK Health Security Agency - Colindale	Galiano,M.
EPI1029653	HA	United Kingdom	2017-Mar-04	EPI_ISL_270571	A/England/71180498/2017	UK Health Security Agency - Colindale	UK Health Security Agency - Colindale	Galiano,M.
EPI1029645	HA	United Kingdom	2017-Mar-06	EPI_ISL_270570	A/England/71180497/2017	UK Health Security Agency - Colindale	UK Health Security Agency - Colindale	Galiano,M.
EPI1029637	HA	United Kingdom	2017-Mar-13	EPI_ISL_270569	A/England/71180492/2017	UK Health Security Agency - Colindale	UK Health Security Agency - Colindale	Galiano,M.
EPI1029629	HA	United Kingdom	2017-Mar-11	EPI_ISL_270568	A/England/71140581/2017	UK Health Security Agency - Colindale	UK Health Security Agency - Colindale	Galiano,M.
EPI1029621	HA	United Kingdom	2017-Mar-10	EPI_ISL_270567	A/England/71140418/2017	UK Health Security Agency - Colindale	UK Health Security Agency - Colindale	Galiano,M.
EPI1029613	HA	United Kingdom	2017-Mar-08	EPI_ISL_270566	A/England/71100707/2017	UK Health Security Agency - Colindale	UK Health Security Agency - Colindale	Galiano,M.

Segment ID	Segment	Country	Collection date	Isolate-ID	Isolate name	Originating Lab	Submitting Lab	Authors
EPI1029605	HA	United Kingdom	2017-Mar-08	EPI_ISL_270565	A/England/71100706/2017	UK Health Security Agency - Colindale	UK Health Security Agency - Colindale	Galiano,M.
EPI1029597	HA	United Kingdom	2017-Mar-08	EPI_ISL_270564	A/England/71100704/2017	UK Health Security Agency - Colindale	UK Health Security Agency - Colindale	Galiano,M.
EPI1029589	HA	United Kingdom	2017-Mar-08	EPI_ISL_270563	A/England/71100703/2017	UK Health Security Agency - Colindale	UK Health Security Agency - Colindale	Galiano,M.
EPI1029581	HA	United Kingdom	2017-Mar-08	EPI_ISL_270562	A/England/71040522/2017	UK Health Security Agency - Colindale	UK Health Security Agency - Colindale	Galiano,M.
EPI1029573	HA	United Kingdom	2017-Mar-05	EPI_ISL_270561	A/England/71040521/2017	UK Health Security Agency - Colindale	UK Health Security Agency - Colindale	Galiano,M.
EPI1029565	HA	United Kingdom	2017-Mar-02	EPI_ISL_270560	A/England/71040518/2017	UK Health Security Agency - Colindale	UK Health Security Agency - Colindale	Galiano,M.
EPI1029557	HA	United Kingdom	2017-Mar-03	EPI_ISL_270559	A/England/71040517/2017	UK Health Security Agency - Colindale	UK Health Security Agency - Colindale	Galiano,M.
EPI1029549	HA	United Kingdom	2017-Mar-06	EPI_ISL_270558	A/England/71040514/2017	UK Health Security Agency - Colindale	UK Health Security Agency - Colindale	Galiano,M.
EPI1029541	HA	United Kingdom	2017-Feb-24	EPI_ISL_270557	A/England/71000565/2017	UK Health Security Agency - Colindale	UK Health Security Agency - Colindale	Galiano,M.
EPI1029533	HA	United Kingdom	2017-Mar-01	EPI_ISL_270556	A/England/70940259/2017	UK Health Security Agency - Colindale	UK Health Security Agency - Colindale	Galiano,M.
EPI1029525	HA	United Kingdom	2017-Feb-24	EPI_ISL_270555	A/England/70880241/2017	UK Health Security Agency - Colindale	UK Health Security Agency - Colindale	Galiano,M.
EPI1029517	HA	United Kingdom	2017-Feb-14	EPI_ISL_270554	A/England/70840527/2017	UK Health Security Agency - Colindale	UK Health Security Agency - Colindale	Galiano,M.
EPI1029509	HA	United Kingdom	2017-Feb-22	EPI_ISL_270553	A/England/70840265/2017	UK Health Security Agency - Colindale	UK Health Security Agency - Colindale	Galiano,M.

Segment ID	Segment	Country	Collection date	Isolate-ID	Isolate name	Originating Lab	Submitting Lab	Authors
EPI1029501	HA	United Kingdom	2017-Feb-21	EPI_ISL_270552	A/England/70820814/2017	UK Health Security Agency - Colindale	UK Health Security Agency - Colindale	Galiano,M.
EPI1029493	HA	United Kingdom	2017-Feb-16	EPI_ISL_270551	A/England/70820366/2017	UK Health Security Agency - Colindale	UK Health Security Agency - Colindale	Galiano,M.
EPI1029485	HA	United Kingdom	2017-Feb-20	EPI_ISL_270550	A/England/70820259/2017	UK Health Security Agency - Colindale	UK Health Security Agency - Colindale	Galiano,M.
EPI1029477	HA	United Kingdom	2017-Feb-20	EPI_ISL_270549	A/England/70800087/2017	UK Health Security Agency - Colindale	UK Health Security Agency - Colindale	Galiano,M.
EPI1029469	HA	United Kingdom	2017-Feb-20	EPI_ISL_270548	A/England/70800086/2017	UK Health Security Agency - Colindale	UK Health Security Agency - Colindale	Galiano,M.
EPI1029461	HA	United Kingdom	2017-Feb-07	EPI_ISL_270547	A/England/70780310/2017	UK Health Security Agency - Colindale	UK Health Security Agency - Colindale	Galiano,M.
EPI1029453	HA	United Kingdom	2017-Feb-07	EPI_ISL_270546	A/England/70780309/2017	UK Health Security Agency - Colindale	UK Health Security Agency - Colindale	Galiano,M.
EPI1029445	HA	United Kingdom	2017-Feb-13	EPI_ISL_270545	A/England/30/2017	UK Health Security Agency - Colindale	UK Health Security Agency - Colindale	Galiano,M.
EPI1029437	HA	United Kingdom	2017-Feb-05	EPI_ISL_270544	A/England/70740088/2017	UK Health Security Agency - Colindale	UK Health Security Agency - Colindale	Galiano,M.
EPI1029429	HA	United Kingdom	2017-Feb-10	EPI_ISL_270543	A/England/70740080/2017	UK Health Security Agency - Colindale	UK Health Security Agency - Colindale	Galiano,M.
EPI1029421	HA	United Kingdom	2017-Jan-31	EPI_ISL_270542	A/England/70720036/2017	UK Health Security Agency - Colindale	UK Health Security Agency - Colindale	Galiano,M.
EPI1029413	HA	United Kingdom	2017-Feb-13	EPI_ISL_270541	A/England/70700076/2017	UK Health Security Agency - Colindale	UK Health Security Agency - Colindale	Galiano,M.
EPI1029405	HA	United Kingdom	2017-Feb-13	EPI_ISL_270540	A/England/70700071/2017	UK Health Security Agency - Colindale	UK Health Security Agency - Colindale	Galiano,M.

Segment ID	Segment	Country	Collection date	Isolate-ID	Isolate name	Originating Lab	Submitting Lab	Authors
EPI1029397	HA	United Kingdom	2017-Feb-03	EPI_ISL_270539	A/England/70680474/2017	UK Health Security Agency - Colindale	UK Health Security Agency - Colindale	Galiano,M.
EPI1029389	HA	United Kingdom	2017-Feb-06	EPI_ISL_270538	A/England/70680440/2017	UK Health Security Agency - Colindale	UK Health Security Agency - Colindale	Galiano,M.
EPI1029381	HA	United Kingdom	2017-Feb-01	EPI_ISL_270537	A/England/70660194/2017	UK Health Security Agency - Colindale	UK Health Security Agency - Colindale	Galiano,M.
EPI1029373	HA	United Kingdom	2017-Feb-01	EPI_ISL_270536	A/England/70660193/2017	UK Health Security Agency - Colindale	UK Health Security Agency - Colindale	Galiano,M.
EPI1029365	HA	United Kingdom	2017-Feb-04	EPI_ISL_270535	A/England/70660085/2017	UK Health Security Agency - Colindale	UK Health Security Agency - Colindale	Galiano,M.
EPI1029357	HA	United Kingdom	2017-Feb-08	EPI_ISL_270534	A/England/70640087/2017	UK Health Security Agency - Colindale	UK Health Security Agency - Colindale	Galiano,M.
EPI1029349	HA	United Kingdom	2017-Feb-08	EPI_ISL_270533	A/England/70640082/2017	UK Health Security Agency - Colindale	UK Health Security Agency - Colindale	Galiano,M.
EPI1029341	HA	United Kingdom	2017-Feb-06	EPI_ISL_270532	A/England/70640077/2017	UK Health Security Agency - Colindale	UK Health Security Agency - Colindale	Galiano,M.
EPI1029333	HA	United Kingdom	2017-Feb-01	EPI_ISL_270531	A/England/70600059/2017	UK Health Security Agency - Colindale	UK Health Security Agency - Colindale	Galiano,M.
EPI1029325	HA	United Kingdom	2017-Feb-01	EPI_ISL_270530	A/England/70580680/2017	UK Health Security Agency - Colindale	UK Health Security Agency - Colindale	Galiano,M.
EPI1029317	HA	United Kingdom	2017-Feb-01	EPI_ISL_270529	A/England/70580679/2017	UK Health Security Agency - Colindale	UK Health Security Agency - Colindale	Galiano,M.
EPI1029309	HA	United Kingdom	2017-Feb-01	EPI_ISL_270528	A/England/70580334/2017	UK Health Security Agency - Colindale	UK Health Security Agency - Colindale	Galiano,M.
EPI1029301	HA	United Kingdom	2017-Jan-31	EPI_ISL_270527	A/England/19/2017	UK Health Security Agency - Colindale	UK Health Security Agency - Colindale	Galiano,M.

Segment ID	Segment	Country	Collection date	Isolate-ID	Isolate name	Originating Lab	Submitting Lab	Authors
EPI1029293	HA	United Kingdom	2017-Jan-27	EPI_ISL_270526	A/England/70580313/2017	UK Health Security Agency - Colindale	UK Health Security Agency - Colindale	Galiano,M.
EPI1029285	HA	United Kingdom	2017-Jan-27	EPI_ISL_270525	A/England/70580304/2017	UK Health Security Agency - Colindale	UK Health Security Agency - Colindale	Galiano,M.
EPI1029277	HA	United Kingdom	2017-Feb-01	EPI_ISL_270524	A/England/70580079/2017	UK Health Security Agency - Colindale	UK Health Security Agency - Colindale	Galiano,M.
EPI1029269	HA	United Kingdom	2017-Jan-29	EPI_ISL_270523	A/England/70560484/2017	UK Health Security Agency - Colindale	UK Health Security Agency - Colindale	Galiano,M.
EPI1029261	HA	United Kingdom	2017-Jan-31	EPI_ISL_270522	A/England/70560120/2017	UK Health Security Agency - Colindale	UK Health Security Agency - Colindale	Galiano,M.
EPI1029253	HA	United Kingdom	2017-Jan-30	EPI_ISL_270521	A/England/70540231/2017	UK Health Security Agency - Colindale	UK Health Security Agency - Colindale	Galiano,M.
EPI1029245	HA	United Kingdom	2017-Jan-30	EPI_ISL_270520	A/England/70540223/2017	UK Health Security Agency - Colindale	UK Health Security Agency - Colindale	Galiano,M.
EPI1029237	HA	United Kingdom	2017-Jan-31	EPI_ISL_270519	A/England/70540221/2017	UK Health Security Agency - Colindale	UK Health Security Agency - Colindale	Galiano,M.
EPI1029229	HA	United Kingdom	2017-Jan-27	EPI_ISL_270518	A/England/70520784/2017	UK Health Security Agency - Colindale	UK Health Security Agency - Colindale	Galiano,M.
EPI1029221	HA	United Kingdom	2017-Jan-31	EPI_ISL_270517	A/England/70520302/2017	UK Health Security Agency - Colindale	UK Health Security Agency - Colindale	Galiano,M.
EPI1029213	HA	United Kingdom	2017-Jan-27	EPI_ISL_270516	A/England/70520076/2017	UK Health Security Agency - Colindale	UK Health Security Agency - Colindale	Galiano,M.
EPI1029205	HA	United Kingdom	2017-Jan-27	EPI_ISL_270515	A/England/70520074/2017	UK Health Security Agency - Colindale	UK Health Security Agency - Colindale	Galiano,M.
EPI1029197	HA	United Kingdom	2016-Nov-18	EPI_ISL_270514	A/England/70500482/2016	UK Health Security Agency - Colindale	UK Health Security Agency - Colindale	Galiano,M.

Segment ID	Segment	Country	Collection date	Isolate-ID	Isolate name	Originating Lab	Submitting Lab	Authors
EPI1029189	HA	United Kingdom	2016-Nov-25	EPI_ISL_270513	A/England/70500481/2016	UK Health Security Agency - Colindale	UK Health Security Agency - Colindale	Galiano,M.
EPI1029181	HA	United Kingdom	2017-Jan-25	EPI_ISL_270512	A/England/70500471/2017	UK Health Security Agency - Colindale	UK Health Security Agency - Colindale	Galiano,M.
EPI1029173	HA	United Kingdom	2017-Jan-20	EPI_ISL_270511	A/England/70480390/2017	UK Health Security Agency - Colindale	UK Health Security Agency - Colindale	Galiano,M.
EPI1029165	HA	United Kingdom	2017-Jan-25	EPI_ISL_270510	A/England/70480389/2017	UK Health Security Agency - Colindale	UK Health Security Agency - Colindale	Galiano,M.
EPI1029157	HA	United Kingdom	2017-Jan-24	EPI_ISL_270509	A/England/70480384/2017	UK Health Security Agency - Colindale	UK Health Security Agency - Colindale	Galiano,M.
EPI1029149	HA	United Kingdom	2017-Jan-25	EPI_ISL_270508	A/England/70480369/2017	UK Health Security Agency - Colindale	UK Health Security Agency - Colindale	Galiano,M.
EPI1029141	HA	United Kingdom	2017-Jan-23	EPI_ISL_270507	A/England/70480365/2017	UK Health Security Agency - Colindale	UK Health Security Agency - Colindale	Galiano,M.
EPI1029133	HA	United Kingdom	2017-Jan-18	EPI_ISL_270506	A/England/70480363/2017	UK Health Security Agency - Colindale	UK Health Security Agency - Colindale	Galiano,M.
EPI1029125	HA	United Kingdom	2017-Jan-27	EPI_ISL_270505	A/England/70480149/2017	UK Health Security Agency - Colindale	UK Health Security Agency - Colindale	Galiano,M.
EPI1029117	HA	United Kingdom	2017-Jan-27	EPI_ISL_270504	A/England/70480140/2017	UK Health Security Agency - Colindale	UK Health Security Agency - Colindale	Galiano,M.
EPI1029109	HA	United Kingdom	2017-Jan-24	EPI_ISL_270503	A/England/70460720/2017	UK Health Security Agency - Colindale	UK Health Security Agency - Colindale	Galiano,M.
EPI1029101	HA	United Kingdom	2017-Jan-24	EPI_ISL_270502	A/England/70460136/2017	UK Health Security Agency - Colindale	UK Health Security Agency - Colindale	Galiano,M.
EPI1029093	HA	United Kingdom	2017-Jan-24	EPI_ISL_270501	A/England/70420063/2017	UK Health Security Agency - Colindale	UK Health Security Agency - Colindale	Galiano,M.

Segment ID	Segment	Country	Collection date	Isolate-ID	Isolate name	Originating Lab	Submitting Lab	Authors
EPI1029085	HA	United Kingdom	2017-Jan-16	EPI_ISL_270500	A/England/70400231/2017	UK Health Security Agency - Colindale	UK Health Security Agency - Colindale	Galiano,M.
EPI1029077	HA	United Kingdom	2017-Jan-14	EPI_ISL_270499	A/England/70400230/2017	UK Health Security Agency - Colindale	UK Health Security Agency - Colindale	Galiano,M.
EPI1029069	HA	United Kingdom	2017-Jan-23	EPI_ISL_270498	A/England/70400012/2017	UK Health Security Agency - Colindale	UK Health Security Agency - Colindale	Galiano,M.
EPI1029061	HA	United Kingdom	2017-Jan-19	EPI_ISL_270497	A/England/31/2017	UK Health Security Agency - Colindale	UK Health Security Agency - Colindale	Galiano,M.
EPI1029053	HA	United Kingdom	2017-Jan-23	EPI_ISL_270496	A/England/70400004/2017	UK Health Security Agency - Colindale	UK Health Security Agency - Colindale	Galiano,M.
EPI1029045	HA	United Kingdom	2017-Jan-18	EPI_ISL_270495	A/England/70400002/2017	UK Health Security Agency - Colindale	UK Health Security Agency - Colindale	Galiano,M.
EPI1029037	HA	United Kingdom	2017-Jan-15	EPI_ISL_270494	A/England/70380309/2017	UK Health Security Agency - Colindale	UK Health Security Agency - Colindale	Galiano,M.
EPI1029029	HA	United Kingdom	2017-Jan-16	EPI_ISL_270493	A/England/70380307/2017	UK Health Security Agency - Colindale	UK Health Security Agency - Colindale	Galiano,M.
EPI1029021	HA	United Kingdom	2017-Jan-16	EPI_ISL_270492	A/England/70380306/2017	UK Health Security Agency - Colindale	UK Health Security Agency - Colindale	Galiano,M.
EPI1029013	HA	United Kingdom	2017-Jan-16	EPI_ISL_270491	A/England/70380300/2017	UK Health Security Agency - Colindale	UK Health Security Agency - Colindale	Galiano,M.
EPI1029005	HA	United Kingdom	2017-Jan-16	EPI_ISL_270490	A/England/70380298/2017	UK Health Security Agency - Colindale	UK Health Security Agency - Colindale	Galiano,M.
EPI1028997	HA	United Kingdom	2017-Jan-20	EPI_ISL_270489	A/England/70380035/2017	UK Health Security Agency - Colindale	UK Health Security Agency - Colindale	Galiano,M.
EPI1028989	HA	United Kingdom	2017-Jan-13	EPI_ISL_270488	A/England/70360631/2017	UK Health Security Agency - Colindale	UK Health Security Agency - Colindale	Galiano,M.

Segment ID	Segment	Country	Collection date	Isolate-ID	Isolate name	Originating Lab	Submitting Lab	Authors
EPI1028981	HA	United Kingdom	2017-Jan-17	EPI_ISL_270487	A/England/70360610/2017	UK Health Security Agency - Colindale	UK Health Security Agency - Colindale	Galiano,M.
EPI1028973	HA	United Kingdom	2017-Jan-17	EPI_ISL_270486	A/England/70360024/2017	UK Health Security Agency - Colindale	UK Health Security Agency - Colindale	Galiano,M.
EPI1028965	HA	United Kingdom	2017-Jan-17	EPI_ISL_270485	A/England/70360020/2017	UK Health Security Agency - Colindale	UK Health Security Agency - Colindale	Galiano,M.
EPI1028957	HA	United Kingdom	2017-Jan-12	EPI_ISL_270484	A/England/70340163/2017	UK Health Security Agency - Colindale	UK Health Security Agency - Colindale	Galiano,M.
EPI1028949	HA	United Kingdom	2017-Jan-16	EPI_ISL_270483	A/England/70340064/2017	UK Health Security Agency - Colindale	UK Health Security Agency - Colindale	Galiano,M.
EPI1028941	HA	United Kingdom	2017-Jan-17	EPI_ISL_270482	A/England/70340062/2017	UK Health Security Agency - Colindale	UK Health Security Agency - Colindale	Galiano,M.
EPI1028933	HA	United Kingdom	2017-Jan-16	EPI_ISL_270481	A/England/70340059/2017	UK Health Security Agency - Colindale	UK Health Security Agency - Colindale	Galiano,M.
EPI1028925	HA	United Kingdom	2017-Jan-17	EPI_ISL_270480	A/England/70320034/2017	UK Health Security Agency - Colindale	UK Health Security Agency - Colindale	Galiano,M.
EPI1028917	HA	United Kingdom	2017-Jan-13	EPI_ISL_270479	A/England/70300013/2017	UK Health Security Agency - Colindale	UK Health Security Agency - Colindale	Galiano,M.
EPI1028909	HA	United Kingdom	2017-Jan-16	EPI_ISL_270478	A/England/70300007/2017	UK Health Security Agency - Colindale	UK Health Security Agency - Colindale	Galiano,M.
EPI1028901	HA	United Kingdom	2017-Jan-13	EPI_ISL_270477	A/England/70280828/2017	UK Health Security Agency - Colindale	UK Health Security Agency - Colindale	Galiano,M.
EPI1028893	HA	United Kingdom	2017-Jan-07	EPI_ISL_270476	A/England/6/2017	UK Health Security Agency - Colindale	UK Health Security Agency - Colindale	Galiano,M.
EPI1028885	HA	United Kingdom	2017-Jan-11	EPI_ISL_270475	A/England/70280069/2017	UK Health Security Agency - Colindale	UK Health Security Agency - Colindale	Galiano,M.

Segment ID	Segment	Country	Collection date	Isolate-ID	Isolate name	Originating Lab	Submitting Lab	Authors
EPI1028877	HA	United Kingdom	2017-Jan-13	EPI_ISL_270474	A/England/70280055/2017	UK Health Security Agency - Colindale	UK Health Security Agency - Colindale	Galiano,M.
EPI1028869	HA	United Kingdom	2017-Jan-12	EPI_ISL_270473	A/England/70280036/2017	UK Health Security Agency - Colindale	UK Health Security Agency - Colindale	Galiano,M.
EPI1028861	HA	United Kingdom	2017-Jan-09	EPI_ISL_270472	A/England/70240100/2017	UK Health Security Agency - Colindale	UK Health Security Agency - Colindale	Galiano,M.
EPI1028853	HA	United Kingdom	2017-Jan-09	EPI_ISL_270471	A/England/70240095/2017	UK Health Security Agency - Colindale	UK Health Security Agency - Colindale	Galiano,M.
EPI1028845	HA	United Kingdom	2017-Jan-10	EPI_ISL_270470	A/England/70220040/2017	UK Health Security Agency - Colindale	UK Health Security Agency - Colindale	Galiano,M.
EPI1028836	HA	United Kingdom	2017-Jan-10	EPI_ISL_270468	A/England/70220039/2017	UK Health Security Agency - Colindale	UK Health Security Agency - Colindale	Galiano,M.
EPI1028828	HA	United Kingdom	2017-Jan-03	EPI_ISL_270467	A/England/70180252/2017	UK Health Security Agency - Colindale	UK Health Security Agency - Colindale	Galiano,M.
EPI1028820	HA	United Kingdom	2016-Dec-30	EPI_ISL_270466	A/England/70180239/2016	UK Health Security Agency - Colindale	UK Health Security Agency - Colindale	Galiano,M.
EPI1028812	HA	United Kingdom	2017-Jan-03	EPI_ISL_270465	A/England/70160053/2017	UK Health Security Agency - Colindale	UK Health Security Agency - Colindale	Galiano,M.
EPI1028804	HA	United Kingdom	2017-Jan-05	EPI_ISL_270464	A/England/70160050/2017	UK Health Security Agency - Colindale	UK Health Security Agency - Colindale	Galiano,M.
EPI1028796	HA	United Kingdom	2016-Dec-30	EPI_ISL_270463	A/England/70140004/2016	UK Health Security Agency - Colindale	UK Health Security Agency - Colindale	Galiano,M.
EPI1028788	HA	United Kingdom	2016-Dec-28	EPI_ISL_270462	A/England/70120297/2016	UK Health Security Agency - Colindale	UK Health Security Agency - Colindale	Galiano,M.
EPI1028780	HA	United Kingdom	2016-Dec-28	EPI_ISL_270461	A/England/70120292/2016	UK Health Security Agency - Colindale	UK Health Security Agency - Colindale	Galiano,M.

Segment ID	Segment	Country	Collection date	Isolate-ID	Isolate name	Originating Lab	Submitting Lab	Authors
EPI1028771	HA	United Kingdom	2016-Dec-28	EPI_ISL_270460	A/England/70120290/2016	UK Health Security Agency - Colindale	UK Health Security Agency - Colindale	Galiano,M.
EPI1028763	HA	United Kingdom	2016-Dec-28	EPI_ISL_270459	A/England/70120289/2016	UK Health Security Agency - Colindale	UK Health Security Agency - Colindale	Galiano,M.
EPI1028755	HA	United Kingdom	2016-Dec-29	EPI_ISL_270458	A/England/70120024/2016	UK Health Security Agency - Colindale	UK Health Security Agency - Colindale	Galiano,M.
EPI1028747	HA	United Kingdom	2016-Dec-28	EPI_ISL_270457	A/England/65280819/2016	UK Health Security Agency - Colindale	UK Health Security Agency - Colindale	Galiano,M.
EPI1028739	HA	United Kingdom	2016-Dec-22	EPI_ISL_270456	A/England/65280698/2016	UK Health Security Agency - Colindale	UK Health Security Agency - Colindale	Galiano,M.
EPI1028731	HA	United Kingdom	2016-Dec-28	EPI_ISL_270455	A/England/65280004/2016	UK Health Security Agency - Colindale	UK Health Security Agency - Colindale	Galiano,M.
EPI1028723	HA	United Kingdom	2016-Dec-16	EPI_ISL_270454	A/England/65260861/2016	UK Health Security Agency - Colindale	UK Health Security Agency - Colindale	Galiano,M.
EPI1028715	HA	United Kingdom	2016-Dec-16	EPI_ISL_270453	A/England/65260859/2016	UK Health Security Agency - Colindale	UK Health Security Agency - Colindale	Galiano,M.
EPI1028707	HA	United Kingdom	2016-Dec-06	EPI_ISL_270452	A/England/65260852/2016	UK Health Security Agency - Colindale	UK Health Security Agency - Colindale	Galiano,M.
EPI1028699	HA	United Kingdom	2016-Dec-28	EPI_ISL_270451	A/England/65240070/2016	UK Health Security Agency - Colindale	UK Health Security Agency - Colindale	Galiano,M.
EPI1028691	HA	United Kingdom	2016-Dec-22	EPI_ISL_270449	A/England/65240016/2016	UK Health Security Agency - Colindale	UK Health Security Agency - Colindale	Galiano,M.
EPI1028683	HA	United Kingdom	2016-Dec-13	EPI_ISL_270448	A/England/65180303/2016	UK Health Security Agency - Colindale	UK Health Security Agency - Colindale	Galiano,M.
EPI1028675	HA	United Kingdom	2016-Dec-21	EPI_ISL_270447	A/England/65180267/2016	UK Health Security Agency - Colindale	UK Health Security Agency - Colindale	Galiano,M.

Segment ID	Segment	Country	Collection date	Isolate-ID	Isolate name	Originating Lab	Submitting Lab	Authors
EPI1028667	HA	United Kingdom	2016-Dec-23	EPI_ISL_270446	A/England/65180259/2016	UK Health Security Agency - Colindale	UK Health Security Agency - Colindale	Galiano,M.
EPI1028659	HA	United Kingdom	2016-Dec-21	EPI_ISL_270445	A/England/65180257/2016	UK Health Security Agency - Colindale	UK Health Security Agency - Colindale	Galiano,M.
EPI1028651	HA	United Kingdom	2016-Dec-20	EPI_ISL_270444	A/England/971/2016	UK Health Security Agency - Colindale	UK Health Security Agency - Colindale	Galiano,M.
EPI1028643	HA	United Kingdom	2016-Dec-20	EPI_ISL_270443	A/England/65160163/2016	UK Health Security Agency - Colindale	UK Health Security Agency - Colindale	Galiano,M.
EPI1028635	HA	United Kingdom	2016-Dec-20	EPI_ISL_270442	A/England/970/2016	UK Health Security Agency - Colindale	UK Health Security Agency - Colindale	Galiano,M.
EPI1028627	HA	United Kingdom	2016-Dec-22	EPI_ISL_270441	A/England/65160116/2016	UK Health Security Agency - Colindale	UK Health Security Agency - Colindale	Galiano,M.
EPI1028619	HA	United Kingdom	2016-Dec-20	EPI_ISL_270440	A/England/65140893/2016	UK Health Security Agency - Colindale	UK Health Security Agency - Colindale	Galiano,M.
EPI1028611	HA	United Kingdom	2016-Dec-21	EPI_ISL_270439	A/England/65140281/2016	UK Health Security Agency - Colindale	UK Health Security Agency - Colindale	Galiano,M.
EPI1028603	HA	United Kingdom	2016-Dec-16	EPI_ISL_270438	A/England/975/2016	UK Health Security Agency - Colindale	UK Health Security Agency - Colindale	Galiano,M.
EPI1028595	HA	United Kingdom	2016-Dec-17	EPI_ISL_270437	A/England/969/2016	UK Health Security Agency - Colindale	UK Health Security Agency - Colindale	Galiano,M.
EPI1028587	HA	United Kingdom	2016-Dec-16	EPI_ISL_270436	A/England/65120006/2016	UK Health Security Agency - Colindale	UK Health Security Agency - Colindale	Galiano,M.
EPI1028579	HA	United Kingdom	2016-Dec-19	EPI_ISL_270435	A/England/65120004/2016	UK Health Security Agency - Colindale	UK Health Security Agency - Colindale	Galiano,M.
EPI1028571	HA	United Kingdom	2016-Dec-15	EPI_ISL_270434	A/England/968/2016	UK Health Security Agency - Colindale	UK Health Security Agency - Colindale	Galiano,M.

Segment ID	Segment	Country	Collection date	Isolate-ID	Isolate name	Originating Lab	Submitting Lab	Authors
EPI1028563	HA	United Kingdom	2016-Dec-11	EPI_ISL_270433	A/England/65080393/2016	UK Health Security Agency - Colindale	UK Health Security Agency - Colindale	Galiano,M.
EPI1028555	HA	United Kingdom	2016-Dec-14	EPI_ISL_270432	A/England/65060442/2016	UK Health Security Agency - Colindale	UK Health Security Agency - Colindale	Galiano,M.
EPI1028547	HA	United Kingdom	2016-Dec-11	EPI_ISL_270431	A/England/967/2016	UK Health Security Agency - Colindale	UK Health Security Agency - Colindale	Galiano,M.
EPI1028539	HA	United Kingdom	2016-Dec-08	EPI_ISL_270430	A/England/966/2016	UK Health Security Agency - Colindale	UK Health Security Agency - Colindale	Galiano,M.
EPI1028531	HA	United Kingdom	2016-Dec-09	EPI_ISL_270429	A/England/65000039/2016	UK Health Security Agency - Colindale	UK Health Security Agency - Colindale	Galiano,M.
EPI1028523	HA	United Kingdom	2016-Dec-12	EPI_ISL_270428	A/England/65000010/2016	UK Health Security Agency - Colindale	UK Health Security Agency - Colindale	Galiano,M.
EPI1028515	HA	United Kingdom	2016-Dec-12	EPI_ISL_270427	A/England/65000001/2016	UK Health Security Agency - Colindale	UK Health Security Agency - Colindale	Galiano,M.
EPI1028507	HA	United Kingdom	2016-Dec-01	EPI_ISL_270426	A/England/64960379/2016	UK Health Security Agency - Colindale	UK Health Security Agency - Colindale	Galiano,M.
EPI1028499	HA	United Kingdom	2016-Dec-06	EPI_ISL_270425	A/England/64920038/2016	UK Health Security Agency - Colindale	UK Health Security Agency - Colindale	Galiano,M.
EPI1028491	HA	United Kingdom	2016-Nov-28	EPI_ISL_270424	A/England/64880514/2016	UK Health Security Agency - Colindale	UK Health Security Agency - Colindale	Galiano,M.
EPI1028483	HA	United Kingdom	2016-Nov-19	EPI_ISL_270423	A/England/64760613/2016	UK Health Security Agency - Colindale	UK Health Security Agency - Colindale	Galiano,M.
EPI1028475	HA	United Kingdom	2016-Nov-24	EPI_ISL_270422	A/England/64760153/2016	UK Health Security Agency - Colindale	UK Health Security Agency - Colindale	Galiano,M.
EPI1028467	HA	United Kingdom	2016-Nov-15	EPI_ISL_270421	A/England/64620002/2016	UK Health Security Agency - Colindale	UK Health Security Agency - Colindale	Galiano,M.

Segment ID	Segment	Country	Collection date	Isolate-ID	Isolate name	Originating Lab	Submitting Lab	Authors
EPI1028459	HA	United Kingdom	2016-Oct-14	EPI_ISL_270420	A/N.Ireland/64360271/2016	UK Health Security Agency - Colindale	UK Health Security Agency - Colindale	Galiano,M.
EPI1028449	HA	United Kingdom	2017-Feb-18	EPI_ISL_270419	A/England/70860422/2017	UK Health Security Agency - Colindale	UK Health Security Agency - Colindale	Galiano,M.
EPI1028441	HA	United Kingdom	2017-Feb-19	EPI_ISL_270418	A/England/70860420/2017	UK Health Security Agency - Colindale	UK Health Security Agency - Colindale	Galiano,M.
EPI1028433	HA	United Kingdom	2017-Feb-18	EPI_ISL_270417	A/England/70860419/2017	UK Health Security Agency - Colindale	UK Health Security Agency - Colindale	Galiano,M.
EPI1028425	HA	United Kingdom	2017-Feb-21	EPI_ISL_270416	A/England/70820260/2017	UK Health Security Agency - Colindale	UK Health Security Agency - Colindale	Galiano,M.
EPI1028417	HA	United Kingdom	2017-Feb-08	EPI_ISL_270415	A/England/70780311/2017	UK Health Security Agency - Colindale	UK Health Security Agency - Colindale	Galiano,M.
EPI1028409	HA	United Kingdom	2017-Feb-07	EPI_ISL_270414	A/England/70740089/2017	UK Health Security Agency - Colindale	UK Health Security Agency - Colindale	Galiano,M.
EPI1028401	HA	United Kingdom	2017-Feb-10	EPI_ISL_270413	A/England/70700078/2017	UK Health Security Agency - Colindale	UK Health Security Agency - Colindale	Galiano,M.
EPI1028393	HA	United Kingdom	2017-Feb-10	EPI_ISL_270412	A/England/70700062/2017	UK Health Security Agency - Colindale	UK Health Security Agency - Colindale	Galiano,M.
EPI1028385	HA	United Kingdom	2017-Feb-02	EPI_ISL_270411	A/England/70680443/2017	UK Health Security Agency - Colindale	UK Health Security Agency - Colindale	Galiano,M.
EPI1028377	HA	United Kingdom	2017-Feb-06	EPI_ISL_270410	A/England/70600061/2017	UK Health Security Agency - Colindale	UK Health Security Agency - Colindale	Galiano,M.
EPI1028369	HA	United Kingdom	2017-Jan-29	EPI_ISL_270409	A/England/70580309/2017	UK Health Security Agency - Colindale	UK Health Security Agency - Colindale	Galiano,M.
EPI1028361	HA	United Kingdom	2017-Jan-31	EPI_ISL_270408	A/England/70580294/2017	UK Health Security Agency - Colindale	UK Health Security Agency - Colindale	Galiano,M.

Segment ID	Segment	Country	Collection date	Isolate-ID	Isolate name	Originating Lab	Submitting Lab	Authors
EPI1028353	HA	United Kingdom	2017-Jan-25	EPI_ISL_270407	A/England/70500472/2017	UK Health Security Agency - Colindale	UK Health Security Agency - Colindale	Galiano,M.
EPI1028345	HA	United Kingdom	2017-Jan-17	EPI_ISL_270406	A/England/70480393/2017	UK Health Security Agency - Colindale	UK Health Security Agency - Colindale	Galiano,M.
EPI1028337	HA	United Kingdom	2017-Jan-27	EPI_ISL_270405	A/England/70480141/2017	UK Health Security Agency - Colindale	UK Health Security Agency - Colindale	Galiano,M.
EPI1028329	HA	United Kingdom	2017-Jan-19	EPI_ISL_270404	A/England/70420721/2017	UK Health Security Agency - Colindale	UK Health Security Agency - Colindale	Galiano,M.
EPI1028321	HA	United Kingdom	2017-Jan-20	EPI_ISL_270403	A/England/70420055/2017	UK Health Security Agency - Colindale	UK Health Security Agency - Colindale	Galiano,M.
EPI1028313	HA	United Kingdom	2017-Jan-16	EPI_ISL_270402	A/England/70400234/2017	UK Health Security Agency - Colindale	UK Health Security Agency - Colindale	Galiano,M.
EPI1028305	HA	United Kingdom	2017-Jan-12	EPI_ISL_270401	A/England/70360629/2017	UK Health Security Agency - Colindale	UK Health Security Agency - Colindale	Galiano,M.
EPI1028297	HA	United Kingdom	2017-Jan-19	EPI_ISL_270400	A/England/70360019/2017	UK Health Security Agency - Colindale	UK Health Security Agency - Colindale	Galiano,M.
EPI1028289	HA	United Kingdom	2017-Jan-16	EPI_ISL_270399	A/England/70340079/2017	UK Health Security Agency - Colindale	UK Health Security Agency - Colindale	Galiano,M.
EPI1028281	HA	United Kingdom	2017-Jan-05	EPI_ISL_270398	A/England/70260050/2017	UK Health Security Agency - Colindale	UK Health Security Agency - Colindale	Galiano,M.
EPI1028273	HA	United Kingdom	2017-Jan-06	EPI_ISL_270397	A/England/70220036/2017	UK Health Security Agency - Colindale	UK Health Security Agency - Colindale	Galiano,M.
EPI1028265	HA	United Kingdom	2016-Dec-25	EPI_ISL_270396	A/England/70140151/2016	UK Health Security Agency - Colindale	UK Health Security Agency - Colindale	Galiano,M.
EPI1028257	HA	United Kingdom	2016-Dec-16	EPI_ISL_270395	A/England/65140265/2016	UK Health Security Agency - Colindale	UK Health Security Agency - Colindale	Galiano,M.

Segment ID	Segment	Country	Collection date	Isolate-ID	Isolate name	Originating Lab	Submitting Lab	Authors
EPI1028248	HA	United Kingdom	2016-Dec-12	EPI_ISL_270394	A/England/65060431/2016	UK Health Security Agency - Colindale	UK Health Security Agency - Colindale	Galiano,M.
EPI1028240	HA	United Kingdom	2016-Dec-05	EPI_ISL_270393	A/England/64900714/2016	UK Health Security Agency - Colindale	UK Health Security Agency - Colindale	Galiano,M.
EPI1028232	HA	United Kingdom	2016-Nov-15	EPI_ISL_270392	A/England/64760617/2016	UK Health Security Agency - Colindale	UK Health Security Agency - Colindale	Galiano,M.
EPI1028224	HA	United Kingdom	2016-Nov-15	EPI_ISL_270391	A/England/64760610/2016	UK Health Security Agency - Colindale	UK Health Security Agency - Colindale	Galiano,M.
EPI1028216	HA	United Kingdom	2016-Nov-02	EPI_ISL_270390	A/England/64520167/2016	UK Health Security Agency - Colindale	UK Health Security Agency - Colindale	Galiano,M.
EPI914795	HA	United Kingdom	2017-Jan-04	EPI_ISL_248575	A/England/70180024/2017	UK Health Security Agency - Colindale	UK Health Security Agency - Colindale	Galiano,M.
EPI914787	HA	United Kingdom	2016-Dec-31	EPI_ISL_248574	A/England/70160677/2016	UK Health Security Agency - Colindale	UK Health Security Agency - Colindale	Galiano,M.
EPI914779	HA	United Kingdom	2016-Dec-22	EPI_ISL_248573	A/England/65280699/2016	UK Health Security Agency - Colindale	UK Health Security Agency - Colindale	Galiano,M.
EPI914763	HA	United Kingdom	2016-Dec-08	EPI_ISL_248571	A/England/64960063/2016	UK Health Security Agency - Colindale	UK Health Security Agency - Colindale	Galiano,M.
EPI914506	HA	United Kingdom	2016-Nov-28	EPI_ISL_248558	A/England/70500480/2016	UK Health Security Agency - Colindale	UK Health Security Agency - Colindale	Galiano,M.
EPI914498	HA	United Kingdom	2017-Jan-15	EPI_ISL_248557	A/England/70380305/2017	UK Health Security Agency - Colindale	UK Health Security Agency - Colindale	Galiano,M.
EPI914490	HA	United Kingdom	2017-Jan-15	EPI_ISL_248556	A/England/70360626/2017	UK Health Security Agency - Colindale	UK Health Security Agency - Colindale	Galiano,M.
EPI914482	HA	United Kingdom	2017-Jan-12	EPI_ISL_248555	A/England/10/2017	UK Health Security Agency - Colindale	UK Health Security Agency - Colindale	Galiano,M.

Segment ID	Segment	Country	Collection date	Isolate-ID	Isolate name	Originating Lab	Submitting Lab	Authors
EPI914474	HA	United Kingdom	2017-Jan-10	EPI_ISL_248554	A/England/70300381/2017	UK Health Security Agency - Colindale	UK Health Security Agency - Colindale	Galiano,M.
EPI914466	HA	United Kingdom	2017-Jan-10	EPI_ISL_248553	A/England/70220588/2017	UK Health Security Agency - Colindale	UK Health Security Agency - Colindale	Galiano,M.
EPI914458	HA	United Kingdom	2017-Jan-06	EPI_ISL_248552	A/England/70220042/2017	UK Health Security Agency - Colindale	UK Health Security Agency - Colindale	Galiano,M.
EPI914450	HA	United Kingdom	2017-Jan-10	EPI_ISL_248551	A/England/70220038/2017	UK Health Security Agency - Colindale	UK Health Security Agency - Colindale	Galiano,M.
EPI914442	HA	United Kingdom	2017-Jan-09	EPI_ISL_248550	A/England/70200051/2017	UK Health Security Agency - Colindale	UK Health Security Agency - Colindale	Galiano,M.
EPI914434	HA	United Kingdom	2017-Jan-05	EPI_ISL_248549	A/England/70200021/2017	UK Health Security Agency - Colindale	UK Health Security Agency - Colindale	Galiano,M.
EPI914426	HA	United Kingdom	2016-Dec-19	EPI_ISL_248548	A/England/70180215/2016	UK Health Security Agency - Colindale	UK Health Security Agency - Colindale	Galiano,M.
EPI914410	HA	United Kingdom	2017-Jan-05	EPI_ISL_248546	A/England/70180012/2017	UK Health Security Agency - Colindale	UK Health Security Agency - Colindale	Galiano,M.
EPI914402	HA	United Kingdom	2017-Jan-05	EPI_ISL_248545	A/England/1/2017	UK Health Security Agency - Colindale	UK Health Security Agency - Colindale	Galiano,M.
EPI914386	HA	United Kingdom	2016-Dec-31	EPI_ISL_248543	A/England/70160670/2016	UK Health Security Agency - Colindale	UK Health Security Agency - Colindale	Galiano,M.
EPI914378	HA	United Kingdom	2016-Dec-28	EPI_ISL_248542	A/England/974/2016	UK Health Security Agency - Colindale	UK Health Security Agency - Colindale	Galiano,M.
EPI914370	HA	United Kingdom	2017-Jan-03	EPI_ISL_248541	A/England/70160070/2017	UK Health Security Agency - Colindale	UK Health Security Agency - Colindale	Galiano,M.
EPI914362	HA	United Kingdom	2017-Jan-03	EPI_ISL_248540	A/England/70160064/2017	UK Health Security Agency - Colindale	UK Health Security Agency - Colindale	Galiano,M.

Segment ID	Segment	Country	Collection date	Isolate-ID	Isolate name	Originating Lab	Submitting Lab	Authors
EPI914354	HA	United Kingdom	2016-Dec-30	EPI_ISL_248539	A/England/70140008/2016	UK Health Security Agency - Colindale	UK Health Security Agency - Colindale	Galiano,M.
EPI914346	HA	United Kingdom	2016-Dec-29	EPI_ISL_248538	A/England/70120322/2016	UK Health Security Agency - Colindale	UK Health Security Agency - Colindale	Galiano,M.
EPI914338	HA	United Kingdom	2016-Dec-28	EPI_ISL_248537	A/England/70120291/2016	UK Health Security Agency - Colindale	UK Health Security Agency - Colindale	Galiano,M.
EPI914330	HA	United Kingdom	2017-Jan-03	EPI_ISL_248536	A/England/70120033/2017	UK Health Security Agency - Colindale	UK Health Security Agency - Colindale	Galiano,M.
EPI914322	HA	United Kingdom	2016-Dec-30	EPI_ISL_248535	A/England/70120030/2016	UK Health Security Agency - Colindale	UK Health Security Agency - Colindale	Galiano,M.
EPI914314	HA	United Kingdom	2016-Dec-30	EPI_ISL_248534	A/England/70120008/2016	UK Health Security Agency - Colindale	UK Health Security Agency - Colindale	Galiano,M.
EPI914290	HA	United Kingdom	2016-Dec-30	EPI_ISL_248531	A/England/65280021/2016	UK Health Security Agency - Colindale	UK Health Security Agency - Colindale	Galiano,M.
EPI914282	HA	United Kingdom	2016-Dec-28	EPI_ISL_248530	A/England/65280015/2016	UK Health Security Agency - Colindale	UK Health Security Agency - Colindale	Galiano,M.
EPI914274	HA	United Kingdom	2016-Dec-30	EPI_ISL_248529	A/England/65280002/2016	UK Health Security Agency - Colindale	UK Health Security Agency - Colindale	Galiano,M.
EPI914266	HA	United Kingdom	2016-Dec-22	EPI_ISL_248528	A/England/65260883/2016	UK Health Security Agency - Colindale	UK Health Security Agency - Colindale	Galiano,M.
EPI914258	HA	United Kingdom	2016-Dec-16	EPI_ISL_248527	A/England/65260857/2016	UK Health Security Agency - Colindale	UK Health Security Agency - Colindale	Galiano,M.
EPI914250	HA	United Kingdom	2016-Dec-26	EPI_ISL_248526	A/England/65260844/2016	UK Health Security Agency - Colindale	UK Health Security Agency - Colindale	Galiano,M.
EPI914242	HA	United Kingdom	2016-Dec-28	EPI_ISL_248525	A/England/65240021/2016	UK Health Security Agency - Colindale	UK Health Security Agency - Colindale	Galiano,M.

Segment ID	Segment	Country	Collection date	Isolate-ID	Isolate name	Originating Lab	Submitting Lab	Authors
EPI914234	HA	United Kingdom	2016-Dec-13	EPI_ISL_248524	A/England/65180304/2016	UK Health Security Agency - Colindale	UK Health Security Agency - Colindale	Galiano,M.
EPI914226	HA	United Kingdom	2016-Dec-21	EPI_ISL_248523	A/England/65180266/2016	UK Health Security Agency - Colindale	UK Health Security Agency - Colindale	Galiano,M.
EPI914218	HA	United Kingdom	2016-Dec-21	EPI_ISL_248522	A/England/65180255/2016	UK Health Security Agency - Colindale	UK Health Security Agency - Colindale	Galiano,M.
EPI914210	HA	United Kingdom	2016-Dec-23	EPI_ISL_248521	A/England/65180254/2016	UK Health Security Agency - Colindale	UK Health Security Agency - Colindale	Galiano,M.
EPI914194	HA	United Kingdom	2016-Dec-22	EPI_ISL_248519	A/England/65160117/2016	UK Health Security Agency - Colindale	UK Health Security Agency - Colindale	Galiano,M.
EPI914186	HA	United Kingdom	2016-Dec-16	EPI_ISL_248518	A/England/65140269/2016	UK Health Security Agency - Colindale	UK Health Security Agency - Colindale	Galiano,M.
EPI914178	HA	United Kingdom	2016-Dec-21	EPI_ISL_248517	A/England/65140264/2016	UK Health Security Agency - Colindale	UK Health Security Agency - Colindale	Galiano,M.
EPI914170	HA	United Kingdom	2016-Dec-17	EPI_ISL_248516	A/England/969/2016	UK Health Security Agency - Colindale	UK Health Security Agency - Colindale	Galiano,M.
EPI914162	HA	United Kingdom	2016-Dec-20	EPI_ISL_248515	A/England/65120001/2016	UK Health Security Agency - Colindale	UK Health Security Agency - Colindale	Galiano,M.
EPI914154	HA	United Kingdom	2016-Dec-19	EPI_ISL_248514	A/England/65100038/2016	UK Health Security Agency - Colindale	UK Health Security Agency - Colindale	Galiano,M.
EPI914146	HA	United Kingdom	2016-Dec-19	EPI_ISL_248513	A/England/65100002/2016	UK Health Security Agency - Colindale	UK Health Security Agency - Colindale	Galiano,M.
EPI914138	HA	United Kingdom	2016-Dec-12	EPI_ISL_248512	A/England/65060461/2016	UK Health Security Agency - Colindale	UK Health Security Agency - Colindale	Galiano,M.
EPI914130	HA	United Kingdom	2016-Dec-15	EPI_ISL_248511	A/England/65060420/2016	UK Health Security Agency - Colindale	UK Health Security Agency - Colindale	Galiano,M.

Segment ID	Segment	Country	Collection date	Isolate-ID	Isolate name	Originating Lab	Submitting Lab	Authors
EPI914122	HA	United Kingdom	2016-Dec-13	EPI_ISL_248510	A/England/65060417/2016	UK Health Security Agency - Colindale	UK Health Security Agency - Colindale	Galiano,M.
EPI914114	HA	United Kingdom	2016-Dec-11	EPI_ISL_248509	A/England/967/2016	UK Health Security Agency - Colindale	UK Health Security Agency - Colindale	Galiano,M.
EPI914106	HA	United Kingdom	2016-Dec-11	EPI_ISL_248508	A/England/65040827/2016	UK Health Security Agency - Colindale	UK Health Security Agency - Colindale	Galiano,M.
EPI914098	HA	United Kingdom	2016-Dec-12	EPI_ISL_248507	A/England/65040088/2016	UK Health Security Agency - Colindale	UK Health Security Agency - Colindale	Galiano,M.
EPI914090	HA	United Kingdom	2016-Dec-12	EPI_ISL_248506	A/England/65040076/2016	UK Health Security Agency - Colindale	UK Health Security Agency - Colindale	Galiano,M.
EPI914082	HA	United Kingdom	2016-Dec-08	EPI_ISL_248505	A/England/65020622/2016	UK Health Security Agency - Colindale	UK Health Security Agency - Colindale	Galiano,M.
EPI914074	HA	United Kingdom	2016-Dec-08	EPI_ISL_248504	A/England/966/2016	UK Health Security Agency - Colindale	UK Health Security Agency - Colindale	Galiano,M.
EPI914066	HA	United Kingdom	2016-Dec-13	EPI_ISL_248503	A/England/65020045/2016	UK Health Security Agency - Colindale	UK Health Security Agency - Colindale	Galiano,M.
EPI914058	HA	United Kingdom	2016-Dec-05	EPI_ISL_248502	A/England/65000770/2016	UK Health Security Agency - Colindale	UK Health Security Agency - Colindale	Galiano,M.
EPI914050	HA	United Kingdom	2016-Dec-12	EPI_ISL_248501	A/England/65000009/2016	UK Health Security Agency - Colindale	UK Health Security Agency - Colindale	Galiano,M.
EPI914042	HA	United Kingdom	2016-Dec-12	EPI_ISL_248500	A/England/65000006/2016	UK Health Security Agency - Colindale	UK Health Security Agency - Colindale	Galiano,M.
EPI914034	HA	United Kingdom	2016-Dec-05	EPI_ISL_248499	A/England/64980539/2016	UK Health Security Agency - Colindale	UK Health Security Agency - Colindale	Galiano,M.
EPI914026	HA	United Kingdom	2016-Dec-06	EPI_ISL_248498	A/England/64980276/2016	UK Health Security Agency - Colindale	UK Health Security Agency - Colindale	Galiano,M.

Segment ID	Segment	Country	Collection date	Isolate-ID	Isolate name	Originating Lab	Submitting Lab	Authors
EPI914018	HA	United Kingdom	2016-Dec-01	EPI_ISL_248497	A/England/64960381/2016	UK Health Security Agency - Colindale	UK Health Security Agency - Colindale	Galiano,M.
EPI914010	HA	United Kingdom	2016-Dec-01	EPI_ISL_248496	A/England/64960377/2016	UK Health Security Agency - Colindale	UK Health Security Agency - Colindale	Galiano,M.
EPI913994	HA	United Kingdom	2016-Dec-07	EPI_ISL_248494	A/England/64940741/2016	UK Health Security Agency - Colindale	UK Health Security Agency - Colindale	Galiano,M.
EPI913986	HA	United Kingdom	2016-Dec-07	EPI_ISL_248493	A/England/64940015/2016	UK Health Security Agency - Colindale	UK Health Security Agency - Colindale	Galiano,M.
EPI913978	HA	United Kingdom	2016-Nov-30	EPI_ISL_248492	A/England/64900713/2016	UK Health Security Agency - Colindale	UK Health Security Agency - Colindale	Galiano,M.
EPI913970	HA	United Kingdom	2016-Dec-05	EPI_ISL_248491	A/England/64900712/2016	UK Health Security Agency - Colindale	UK Health Security Agency - Colindale	Galiano,M.
EPI913962	HA	United Kingdom	2016-Nov-29	EPI_ISL_248490	A/England/965/2016	UK Health Security Agency - Colindale	UK Health Security Agency - Colindale	Galiano,M.
EPI913954	HA	United Kingdom	2016-Nov-15	EPI_ISL_248489	A/England/64760616/2016	UK Health Security Agency - Colindale	UK Health Security Agency - Colindale	Galiano,M.
EPI913946	HA	United Kingdom	2016-Nov-19	EPI_ISL_248488	A/England/64760612/2016	UK Health Security Agency - Colindale	UK Health Security Agency - Colindale	Galiano,M.
EPI913938	HA	United Kingdom	2016-Nov-16	EPI_ISL_248487	A/England/64680283/2016	UK Health Security Agency - Colindale	UK Health Security Agency - Colindale	Galiano,M.
EPI913930	HA	United Kingdom	2016-Nov-14	EPI_ISL_248486	A/England/64600021/2016	UK Health Security Agency - Colindale	UK Health Security Agency - Colindale	Galiano,M.
EPI913922	HA	United Kingdom	2016-Oct-31	EPI_ISL_248485	A/England/64460096/2016	UK Health Security Agency - Colindale	UK Health Security Agency - Colindale	Galiano,M.
EPI913913	HA	United Kingdom	2016-Oct-27	EPI_ISL_248484	A/England/64380522/2016	UK Health Security Agency - Colindale	UK Health Security Agency - Colindale	Galiano,M.

Segment ID	Segment	Country	Collection date	Isolate-ID	Isolate name	Originating Lab	Submitting Lab	Authors
EPI913905	HA	United Kingdom	2016-Oct-22	EPI_ISL_248483	A/N.Ireland/64360270/2016	UK Health Security Agency - Colindale	UK Health Security Agency - Colindale	Galiano,M.
EPI913897	HA	United Kingdom	2016-Oct-02	EPI_ISL_248482	A/England/64060217/2016	UK Health Security Agency - Colindale	UK Health Security Agency - Colindale	Galiano,M.
EPI868789	HA	United Kingdom	2016-Aug-30	EPI_ISL_239773	A/England/962/2016	UK Health Security Agency - Colindale	Crick Worldwide Influenza Centre	
EPI868787	HA	United Kingdom	2016-Nov-15	EPI_ISL_239772	A/England/64680170/2016	UK Health Security Agency - Colindale	Crick Worldwide Influenza Centre	
EPI868785	HA	United Kingdom	2016-Nov-15	EPI_ISL_239771	A/England/64680169/2016	UK Health Security Agency - Colindale	Crick Worldwide Influenza Centre	
EPI868783	HA	United Kingdom	2016-Nov-14	EPI_ISL_239770	A/England/64680165/2016	UK Health Security Agency - Colindale	Crick Worldwide Influenza Centre	
EPI868781	HA	United Kingdom	2016-Nov-08	EPI_ISL_239769	A/England/64580216/2016	UK Health Security Agency - Colindale	Crick Worldwide Influenza Centre	
EPI868779	HA	United Kingdom	2016-Nov-08	EPI_ISL_239768	A/England/64580215/2016	UK Health Security Agency - Colindale	Crick Worldwide Influenza Centre	
EPI868777	HA	United Kingdom	2016-Nov-08	EPI_ISL_239767	A/England/64560781/2016	UK Health Security Agency - Colindale	Crick Worldwide Influenza Centre	
EPI868775	HA	United Kingdom	2016-Nov-07	EPI_ISL_239766	A/England/64540250/2016	UK Health Security Agency - Colindale	Crick Worldwide Influenza Centre	
EPI868773	HA	United Kingdom	2016-Nov-02	EPI_ISL_239765	A/England/64520167/2016	UK Health Security Agency - Colindale	Crick Worldwide Influenza Centre	
EPI868771	HA	United Kingdom	2016-Nov-01	EPI_ISL_239764	A/England/64520165/2016	UK Health Security Agency - Colindale	Crick Worldwide Influenza Centre	
EPI868769	HA	United Kingdom	2016-Oct-28	EPI_ISL_239763	A/England/64460094/2016	UK Health Security Agency - Colindale	Crick Worldwide Influenza Centre	
EPI859071	HA	United Kingdom	2016-Aug-25	EPI_ISL_237667	A/Scotland/63440583/2016	UK Health Security Agency - Colindale	Crick Worldwide Influenza Centre	
EPI859069	HA	United Kingdom	2016-Aug-25	EPI_ISL_237666	A/Scotland/63440582/2016	UK Health Security Agency - Colindale	Crick Worldwide Influenza Centre	
EPI859067	HA	United Kingdom	2016-Aug-25	EPI_ISL_237665	A/Scotland/63440579/2016	UK Health Security Agency - Colindale	Crick Worldwide Influenza Centre	
EPI859065	HA	United Kingdom	2016-Aug-25	EPI_ISL_237664	A/Scotland/63440578/2016	UK Health Security Agency - Colindale	Crick Worldwide Influenza Centre	
EPI859063	HA	United Kingdom	2016-Aug-25	EPI_ISL_237663	A/Scotland/63440575/2016	UK Health Security Agency - Colindale	Crick Worldwide Influenza Centre	

Segment ID	Segment	Country	Collection date	Isolate-ID	Isolate name	Originating Lab	Submitting Lab	Authors
EPI859061	HA	United Kingdom	2016-Aug-25	EPI_ISL_237662	A/Scotland/63440574/2016	UK Health Security Agency - Colindale	Crick Worldwide Influenza Centre	
EPI858939	HA	United Kingdom	2016-Jul-07	EPI_ISL_237601	A/England/62840631/2016	UK Health Security Agency - Colindale	Crick Worldwide Influenza Centre	
EPI858937	HA	United Kingdom	2016-Jun-12	EPI_ISL_237600	A/England/62440429/2016	UK Health Security Agency - Colindale	Crick Worldwide Influenza Centre	
EPI858935	HA	United Kingdom	2016-Jun-12	EPI_ISL_237599	A/England/62440428/2016	UK Health Security Agency - Colindale	Crick Worldwide Influenza Centre	
EPI858933	HA	United Kingdom	2016-Jun-12	EPI_ISL_237598	A/England/62440426/2016	UK Health Security Agency - Colindale	Crick Worldwide Influenza Centre	
EPI1082230	HA	United Kingdom	2017-Oct-16	EPI_ISL_282213	A/Singapore/INFIMH-16-0019/2016 NIB-104 (17/194)		National Institute for Biological Standards and Control (NIBSC)	Nicolson, Carolyn
EPI1081401	HA	United Kingdom	2017-Oct-05	EPI_ISL_282009	A/Hong Kong/50/2016 NYMC-X-297 (17/190)		National Institute for Biological Standards and Control (NIBSC)	Nicolson, Carolyn
EPI1077151	HA	United Kingdom	2017-Sep-29	EPI_ISL_281392	A/Norway/3806/2016 NIB-103 (17/184)		National Institute for Biological Standards and Control (NIBSC)	Nicolson, Carolyn
EPI1242696	HA	United Kingdom	2018-Mar-09	EPI_ISL_312134	A/England/81260404/2018	UK Health Security Agency - Colindale	UK Health Security Agency - Colindale	Galiano,M.
EPI1242692	HA	United Kingdom	2018-Mar-12	EPI_ISL_312132	A/England/81260389/2018	UK Health Security Agency - Colindale	UK Health Security Agency - Colindale	Galiano,M.
EPI1242690	HA	United Kingdom	2018-Mar-16	EPI_ISL_312131	A/England/81240430/2018	UK Health Security Agency - Colindale	UK Health Security Agency - Colindale	Galiano,M.
EPI1242682	HA	United Kingdom	2018-Mar-06	EPI_ISL_312127	A/England/81140626/2018	UK Health Security Agency - Colindale	UK Health Security Agency - Colindale	Galiano,M.
EPI1242668	HA	United Kingdom	2018-Mar-05	EPI_ISL_312118	A/England/81080575/2018	UK Health Security Agency - Colindale	UK Health Security Agency - Colindale	Galiano,M.

Segment ID	Segment	Country	Collection date	Isolate-ID	Isolate name	Originating Lab	Submitting Lab	Authors
EPI1242663	HA	United Kingdom	2018-Feb-22	EPI_ISL_312115	A/England/80980298/2018	UK Health Security Agency - Colindale	UK Health Security Agency - Colindale	Galiano,M.
EPI1242629	HA	United Kingdom	2018-Mar-08	EPI_ISL_312101	A/England/81260406/2018	UK Health Security Agency - Colindale	UK Health Security Agency - Colindale	Galiano,M.
EPI1242597	HA	United Kingdom	2018-Mar-11	EPI_ISL_312097	A/England/81260391/2018	UK Health Security Agency - Colindale	UK Health Security Agency - Colindale	Galiano,M.
EPI1242589	HA	United Kingdom	2018-Mar-14	EPI_ISL_312096	A/England/81260388/2018	UK Health Security Agency - Colindale	UK Health Security Agency - Colindale	Galiano,M.
EPI1242573	HA	United Kingdom	2018-Mar-11	EPI_ISL_312094	A/England/81200483/2018	UK Health Security Agency - Colindale	UK Health Security Agency - Colindale	Galiano,M.
EPI1242541	HA	United Kingdom	2018-Mar-16	EPI_ISL_312090	A/England/81180616/2018	UK Health Security Agency - Colindale	UK Health Security Agency - Colindale	Galiano,M.
EPI1242533	HA	United Kingdom	2018-Mar-02	EPI_ISL_312089	A/England/81180615/2018	UK Health Security Agency - Colindale	UK Health Security Agency - Colindale	Galiano,M.
EPI1242525	HA	United Kingdom	2018-Mar-08	EPI_ISL_312088	A/England/81180613/2018	UK Health Security Agency - Colindale	UK Health Security Agency - Colindale	Galiano,M.
EPI1242517	HA	United Kingdom	2018-Mar-09	EPI_ISL_312087	A/England/81180611/2018	UK Health Security Agency - Colindale	UK Health Security Agency - Colindale	Galiano,M.
EPI1242509	HA	United Kingdom	2018-Feb-28	EPI_ISL_312086	A/England/81180607/2018	UK Health Security Agency - Colindale	UK Health Security Agency - Colindale	Galiano,M.
EPI1242501	HA	United Kingdom	2018-Mar-05	EPI_ISL_312085	A/England/81140633/2018	UK Health Security Agency - Colindale	UK Health Security Agency - Colindale	Galiano,M.
EPI1242485	HA	United Kingdom	2018-Mar-07	EPI_ISL_312083	A/England/81140628/2018	UK Health Security Agency - Colindale	UK Health Security Agency - Colindale	Galiano,M.
EPI1242477	HA	United Kingdom	2018-Mar-08	EPI_ISL_312082	A/England/81140622/2018	UK Health Security Agency - Colindale	UK Health Security Agency - Colindale	Galiano,M.

Segment ID	Segment	Country	Collection date	Isolate-ID	Isolate name	Originating Lab	Submitting Lab	Authors
EPI1242469	HA	United Kingdom	2018-Mar-06	EPI_ISL_312081	A/England/81140621/2018	UK Health Security Agency - Colindale	UK Health Security Agency - Colindale	Galiano,M.
EPI1242461	HA	United Kingdom	2018-Mar-07	EPI_ISL_312080	A/England/81140617/2018	UK Health Security Agency - Colindale	UK Health Security Agency - Colindale	Galiano,M.
EPI1242437	HA	United Kingdom	2018-Mar-10	EPI_ISL_312077	A/England/81140611/2018	UK Health Security Agency - Colindale	UK Health Security Agency - Colindale	Galiano,M.
EPI1242429	HA	United Kingdom	2018-Mar-06	EPI_ISL_312076	A/England/81120524/2018	UK Health Security Agency - Colindale	UK Health Security Agency - Colindale	Galiano,M.
EPI1242421	HA	United Kingdom	2018-Mar-05	EPI_ISL_312075	A/England/81120523/2018	UK Health Security Agency - Colindale	UK Health Security Agency - Colindale	Galiano,M.
EPI1242413	HA	United Kingdom	2018-Mar-06	EPI_ISL_312074	A/England/81120520/2018	UK Health Security Agency - Colindale	UK Health Security Agency - Colindale	Galiano,M.
EPI1242405	HA	United Kingdom	2018-Mar-04	EPI_ISL_312073	A/England/81120517/2018	UK Health Security Agency - Colindale	UK Health Security Agency - Colindale	Galiano,M.
EPI1242389	HA	United Kingdom	2018-Mar-04	EPI_ISL_312071	A/England/81120515/2018	UK Health Security Agency - Colindale	UK Health Security Agency - Colindale	Galiano,M.
EPI1242381	HA	United Kingdom	2018-Mar-01	EPI_ISL_312070	A/England/81120506/2018	UK Health Security Agency - Colindale	UK Health Security Agency - Colindale	Galiano,M.
EPI1242373	HA	United Kingdom	2018-Mar-05	EPI_ISL_312069	A/England/81120505/2018	UK Health Security Agency - Colindale	UK Health Security Agency - Colindale	Galiano,M.
EPI1242365	HA	United Kingdom	2018-Mar-05	EPI_ISL_312068	A/England/81120503/2018	UK Health Security Agency - Colindale	UK Health Security Agency - Colindale	Galiano,M.
EPI1242357	HA	United Kingdom	2018-Mar-06	EPI_ISL_312067	A/England/81120500/2018	UK Health Security Agency - Colindale	UK Health Security Agency - Colindale	Galiano,M.
EPI1242349	HA	United Kingdom	2018-Mar-05	EPI_ISL_312066	A/England/81120497/2018	UK Health Security Agency - Colindale	UK Health Security Agency - Colindale	Galiano,M.

Segment ID	Segment	Country	Collection date	Isolate-ID	Isolate name	Originating Lab	Submitting Lab	Authors
EPI1242341	HA	United Kingdom	2018-Feb-05	EPI_ISL_312065	A/England/81100470/2018	UK Health Security Agency - Colindale	UK Health Security Agency - Colindale	Galiano,M.
EPI1242333	HA	United Kingdom	2018-Feb-04	EPI_ISL_312064	A/England/81100469/2018	UK Health Security Agency - Colindale	UK Health Security Agency - Colindale	Galiano,M.
EPI1242293	HA	United Kingdom	2018-Feb-16	EPI_ISL_312059	A/England/81080589/2018	UK Health Security Agency - Colindale	UK Health Security Agency - Colindale	Galiano,M.
EPI1205150	HA	United Kingdom	2018-Feb-22	EPI_ISL_304951	A/England/80980300/2018	UK Health Security Agency - Colindale	UK Health Security Agency - Colindale	Galiano,M.
EPI1205142	HA	United Kingdom	2018-Feb-20	EPI_ISL_304950	A/England/80980296/2018	UK Health Security Agency - Colindale	UK Health Security Agency - Colindale	Galiano,M.
EPI1205134	HA	United Kingdom	2018-Feb-22	EPI_ISL_304949	A/England/80980293/2018	UK Health Security Agency - Colindale	UK Health Security Agency - Colindale	Galiano,M.
EPI1205126	HA	United Kingdom	2018-Feb-26	EPI_ISL_304948	A/England/80960638/2018	UK Health Security Agency - Colindale	UK Health Security Agency - Colindale	Galiano,M.
EPI1205094	HA	United Kingdom	2018-Feb-25	EPI_ISL_304944	A/England/80940356/2018	UK Health Security Agency - Colindale	UK Health Security Agency - Colindale	Galiano,M.
EPI1205086	HA	United Kingdom	2018-Feb-20	EPI_ISL_304943	A/England/80920519/2018	UK Health Security Agency - Colindale	UK Health Security Agency - Colindale	Galiano,M.
EPI1205078	HA	United Kingdom	2018-Feb-24	EPI_ISL_304942	A/England/80920517/2018	UK Health Security Agency - Colindale	UK Health Security Agency - Colindale	Galiano,M.
EPI1205054	HA	United Kingdom	2018-Feb-23	EPI_ISL_304939	A/England/80920509/2018	UK Health Security Agency - Colindale	UK Health Security Agency - Colindale	Galiano,M.
EPI1205038	HA	United Kingdom	2018-Feb-20	EPI_ISL_304937	A/England/80920503/2018	UK Health Security Agency - Colindale	UK Health Security Agency - Colindale	Galiano,M.
EPI1205030	HA	United Kingdom	2018-Feb-20	EPI_ISL_304936	A/England/80920501/2018	UK Health Security Agency - Colindale	UK Health Security Agency - Colindale	Galiano,M.

Segment ID	Segment	Country	Collection date	Isolate-ID	Isolate name	Originating Lab	Submitting Lab	Authors
EPI1205014	HA	United Kingdom	2018-Feb-21	EPI_ISL_304934	A/England/80920499/2018	UK Health Security Agency - Colindale	UK Health Security Agency - Colindale	Galiano,M.
EPI1205006	HA	United Kingdom	2018-Feb-24	EPI_ISL_304933	A/England/80920498/2018	UK Health Security Agency - Colindale	UK Health Security Agency - Colindale	Galiano,M.
EPI1204990	HA	United Kingdom	2018-Jan-29	EPI_ISL_304931	A/England/80900491/2018	UK Health Security Agency - Colindale	UK Health Security Agency - Colindale	Galiano,M.
EPI1204982	HA	United Kingdom	2018-Jan-29	EPI_ISL_304930	A/England/80900490/2018	UK Health Security Agency - Colindale	UK Health Security Agency - Colindale	Galiano,M.
EPI1204958	HA	United Kingdom	2018-Feb-19	EPI_ISL_304927	A/England/80880577/2018	UK Health Security Agency - Colindale	UK Health Security Agency - Colindale	Galiano,M.
EPI1204950	HA	United Kingdom	2018-Feb-16	EPI_ISL_304926	A/England/80880576/2018	UK Health Security Agency - Colindale	UK Health Security Agency - Colindale	Galiano,M.
EPI1204942	HA	United Kingdom	2018-Feb-16	EPI_ISL_304925	A/England/80880575/2018	UK Health Security Agency - Colindale	UK Health Security Agency - Colindale	Galiano,M.
EPI1204934	HA	United Kingdom	2018-Feb-16	EPI_ISL_304924	A/England/80880574/2018	UK Health Security Agency - Colindale	UK Health Security Agency - Colindale	Galiano,M.
EPI1204926	HA	United Kingdom	2018-Feb-16	EPI_ISL_304923	A/England/80880570/2018	UK Health Security Agency - Colindale	UK Health Security Agency - Colindale	Galiano,M.
EPI1204918	HA	United Kingdom	2018-Jan-26	EPI_ISL_304922	A/England/80880551/2018	UK Health Security Agency - Colindale	UK Health Security Agency - Colindale	Galiano,M.
EPI1204910	HA	United Kingdom	2018-Jan-27	EPI_ISL_304921	A/England/80880549/2018	UK Health Security Agency - Colindale	UK Health Security Agency - Colindale	Galiano,M.
EPI1204894	HA	United Kingdom	2018-Feb-13	EPI_ISL_304919	A/England/80860551/2018	UK Health Security Agency - Colindale	UK Health Security Agency - Colindale	Galiano,M.
EPI1204886	HA	United Kingdom	2018-Feb-13	EPI_ISL_304918	A/England/80860550/2018	UK Health Security Agency - Colindale	UK Health Security Agency - Colindale	Galiano,M.

Segment ID	Segment	Country	Collection date	Isolate-ID	Isolate name	Originating Lab	Submitting Lab	Authors
EPI1204878	HA	United Kingdom	2018-Feb-17	EPI_ISL_304917	A/England/80860544/2018	UK Health Security Agency - Colindale	UK Health Security Agency - Colindale	Galiano,M.
EPI1204870	HA	United Kingdom	2018-Feb-17	EPI_ISL_304916	A/England/80860542/2018	UK Health Security Agency - Colindale	UK Health Security Agency - Colindale	Galiano,M.
EPI1204862	HA	United Kingdom	2018-Feb-18	EPI_ISL_304915	A/England/80860540/2018	UK Health Security Agency - Colindale	UK Health Security Agency - Colindale	Galiano,M.
EPI1204854	HA	United Kingdom	2018-Feb-15	EPI_ISL_304914	A/England/80820443/2018	UK Health Security Agency - Colindale	UK Health Security Agency - Colindale	Galiano,M.
EPI1204846	HA	United Kingdom	2018-Feb-19	EPI_ISL_304913	A/England/80820442/2018	UK Health Security Agency - Colindale	UK Health Security Agency - Colindale	Galiano,M.
EPI1204790	HA	United Kingdom	2018-Feb-15	EPI_ISL_304906	A/England/80780400/2018	UK Health Security Agency - Colindale	UK Health Security Agency - Colindale	Galiano,M.
EPI1204774	HA	United Kingdom	2018-Feb-12	EPI_ISL_304904	A/England/80780396/2018	UK Health Security Agency - Colindale	UK Health Security Agency - Colindale	Galiano,M.
EPI1204750	HA	United Kingdom	2018-Feb-13	EPI_ISL_304901	A/England/80760805/2018	UK Health Security Agency - Colindale	UK Health Security Agency - Colindale	Galiano,M.
EPI1204734	HA	United Kingdom	2018-Feb-12	EPI_ISL_304899	A/England/80760794/2018	UK Health Security Agency - Colindale	UK Health Security Agency - Colindale	Galiano,M.
EPI1204702	HA	United Kingdom	2018-Feb-13	EPI_ISL_304895	A/England/80760344/2018	UK Health Security Agency - Colindale	UK Health Security Agency - Colindale	Galiano,M.
EPI1204670	HA	United Kingdom	2018-Feb-12	EPI_ISL_304891	A/England/80740428/2018	UK Health Security Agency - Colindale	UK Health Security Agency - Colindale	Galiano,M.
EPI1204662	HA	United Kingdom	2018-Feb-12	EPI_ISL_304890	A/England/80740426/2018	UK Health Security Agency - Colindale	UK Health Security Agency - Colindale	Galiano,M.
EPI1204654	HA	United Kingdom	2018-Feb-13	EPI_ISL_304889	A/England/80740425/2018	UK Health Security Agency - Colindale	UK Health Security Agency - Colindale	Galiano,M.

Segment ID	Segment	Country	Collection date	Isolate-ID	Isolate name	Originating Lab	Submitting Lab	Authors
EPI1204646	HA	United Kingdom	2018-Feb-13	EPI_ISL_304888	A/England/80740423/2018	UK Health Security Agency - Colindale	UK Health Security Agency - Colindale	Galiano,M.
EPI1204630	HA	United Kingdom	2018-Feb-12	EPI_ISL_304886	A/England/80740421/2018	UK Health Security Agency - Colindale	UK Health Security Agency - Colindale	Galiano,M.
EPI1204614	HA	United Kingdom	2018-Feb-13	EPI_ISL_304884	A/England/80740412/2018	UK Health Security Agency - Colindale	UK Health Security Agency - Colindale	Galiano,M.
EPI1204606	HA	United Kingdom	2018-Feb-09	EPI_ISL_304883	A/England/80720311/2018	UK Health Security Agency - Colindale	UK Health Security Agency - Colindale	Galiano,M.
EPI1204574	HA	United Kingdom	2018-Feb-08	EPI_ISL_304879	A/England/80700360/2018	UK Health Security Agency - Colindale	UK Health Security Agency - Colindale	Galiano,M.
EPI1204558	HA	United Kingdom	2018-Feb-09	EPI_ISL_304877	A/England/80700302/2018	UK Health Security Agency - Colindale	UK Health Security Agency - Colindale	Galiano,M.
EPI1204550	HA	United Kingdom	2018-Feb-09	EPI_ISL_304876	A/England/80700299/2018	UK Health Security Agency - Colindale	UK Health Security Agency - Colindale	Galiano,M.
EPI1204510	HA	United Kingdom	2018-Feb-09	EPI_ISL_304871	A/England/80700288/2018	UK Health Security Agency - Colindale	UK Health Security Agency - Colindale	Galiano,M.
EPI1204478	HA	United Kingdom	2018-Feb-07	EPI_ISL_304867	A/England/80700280/2018	UK Health Security Agency - Colindale	UK Health Security Agency - Colindale	Galiano,M.
EPI1204406	HA	United Kingdom	2018-Feb-05	EPI_ISL_304858	A/England/80640842/2018	UK Health Security Agency - Colindale	UK Health Security Agency - Colindale	Galiano,M.
EPI1204398	HA	United Kingdom	2018-Jan-22	EPI_ISL_304857	A/England/80640624/2018	UK Health Security Agency - Colindale	UK Health Security Agency - Colindale	Galiano,M.
EPI1204382	HA	United Kingdom	2018-Jan-19	EPI_ISL_304855	A/England/80640613/2018	UK Health Security Agency - Colindale	UK Health Security Agency - Colindale	Galiano,M.
EPI1204374	HA	United Kingdom	2018-Jan-18	EPI_ISL_304854	A/England/80640609/2018	UK Health Security Agency - Colindale	UK Health Security Agency - Colindale	Galiano,M.

Segment ID	Segment	Country	Collection date	Isolate-ID	Isolate name	Originating Lab	Submitting Lab	Authors
EPI1204342	HA	United Kingdom	2018-Jan-24	EPI_ISL_304850	A/England/80620419/2018	UK Health Security Agency - Colindale	UK Health Security Agency - Colindale	Galiano,M.
EPI1204334	HA	United Kingdom	2018-Jan-30	EPI_ISL_304849	A/England/80620416/2018	UK Health Security Agency - Colindale	UK Health Security Agency - Colindale	Galiano,M.
EPI1204310	HA	United Kingdom	2018-Jan-18	EPI_ISL_304846	A/England/80600456/2018	UK Health Security Agency - Colindale	UK Health Security Agency - Colindale	Galiano,M.
EPI1204294	HA	United Kingdom	2018-Jan-17	EPI_ISL_304844	A/England/80600451/2018	UK Health Security Agency - Colindale	UK Health Security Agency - Colindale	Galiano,M.
EPI1204214	HA	United Kingdom	2018-Jan-30	EPI_ISL_304834	A/England/80580567/2018	UK Health Security Agency - Colindale	UK Health Security Agency - Colindale	Galiano,M.
EPI1204206	HA	United Kingdom	2018-Jan-30	EPI_ISL_304833	A/England/80580566/2018	UK Health Security Agency - Colindale	UK Health Security Agency - Colindale	Galiano,M.
EPI1204198	HA	United Kingdom	2018-Jan-31	EPI_ISL_304832	A/England/80580564/2018	UK Health Security Agency - Colindale	UK Health Security Agency - Colindale	Galiano,M.
EPI1204190	HA	United Kingdom	2018-Jan-31	EPI_ISL_304831	A/England/80580562/2018	UK Health Security Agency - Colindale	UK Health Security Agency - Colindale	Galiano,M.
EPI1204182	HA	United Kingdom	2018-Jan-24	EPI_ISL_304830	A/England/80580558/2018	UK Health Security Agency - Colindale	UK Health Security Agency - Colindale	Galiano,M.
EPI1204174	HA	United Kingdom	2018-Jan-22	EPI_ISL_304829	A/England/80580557/2018	UK Health Security Agency - Colindale	UK Health Security Agency - Colindale	Galiano,M.
EPI1204166	HA	United Kingdom	2018-Jan-24	EPI_ISL_304828	A/England/80580556/2018	UK Health Security Agency - Colindale	UK Health Security Agency - Colindale	Galiano,M.
EPI1204142	HA	United Kingdom	2018-Jan-24	EPI_ISL_304825	A/England/80580549/2018	UK Health Security Agency - Colindale	UK Health Security Agency - Colindale	Galiano,M.
EPI1204110	HA	United Kingdom	2018-Jan-15	EPI_ISL_304821	A/England/80580518/2018	UK Health Security Agency - Colindale	UK Health Security Agency - Colindale	Galiano,M.

Segment ID	Segment	Country	Collection date	Isolate-ID	Isolate name	Originating Lab	Submitting Lab	Authors
EPI1204062	HA	United Kingdom	2018-Jan-27	EPI_ISL_304815	A/England/80560673/2018	UK Health Security Agency - Colindale	UK Health Security Agency - Colindale	Galiano,M.
EPI1204054	HA	United Kingdom	2018-Jan-26	EPI_ISL_304814	A/England/80560672/2018	UK Health Security Agency - Colindale	UK Health Security Agency - Colindale	Galiano,M.
EPI1204046	HA	United Kingdom	2018-Feb-01	EPI_ISL_304813	A/England/80560665/2018	UK Health Security Agency - Colindale	UK Health Security Agency - Colindale	Galiano,M.
EPI1204038	HA	United Kingdom	2018-Feb-01	EPI_ISL_304812	A/England/80560663/2018	UK Health Security Agency - Colindale	UK Health Security Agency - Colindale	Galiano,M.
EPI1204022	HA	United Kingdom	2018-Jan-31	EPI_ISL_304810	A/England/80560659/2018	UK Health Security Agency - Colindale	UK Health Security Agency - Colindale	Galiano,M.
EPI1204014	HA	United Kingdom	2018-Jan-26	EPI_ISL_304809	A/England/80560656/2018	UK Health Security Agency - Colindale	UK Health Security Agency - Colindale	Galiano,M.
EPI1203990	HA	United Kingdom	2018-Jan-26	EPI_ISL_304806	A/England/80500604/2018	UK Health Security Agency - Colindale	UK Health Security Agency - Colindale	Galiano,M.
EPI1203982	HA	United Kingdom	2018-Jan-26	EPI_ISL_304805	A/England/80500600/2018	UK Health Security Agency - Colindale	UK Health Security Agency - Colindale	Galiano,M.
EPI1203833	HA	United Kingdom	2018-Mar-01	EPI_ISL_304786	A/England/80980303/2018	UK Health Security Agency - Colindale	UK Health Security Agency - Colindale	Galiano,M.
EPI1203830	HA	United Kingdom	2018-Feb-25	EPI_ISL_304784	A/England/80940358/2018	UK Health Security Agency - Colindale	UK Health Security Agency - Colindale	Galiano,M.
EPI1203826	HA	United Kingdom	2018-Jan-29	EPI_ISL_304782	A/England/80900492/2018	UK Health Security Agency - Colindale	UK Health Security Agency - Colindale	Galiano,M.
EPI1203821	HA	United Kingdom	2018-Jan-27	EPI_ISL_304779	A/England/80880566/2018	UK Health Security Agency - Colindale	UK Health Security Agency - Colindale	Galiano,M.
EPI1203787	HA	United Kingdom	2018-Feb-05	EPI_ISL_304761	A/England/80660399/2018	UK Health Security Agency - Colindale	UK Health Security Agency - Colindale	Galiano,M.

Segment ID	Segment	Country	Collection date	Isolate-ID	Isolate name	Originating Lab	Submitting Lab	Authors
EPI1194679	HA	United Kingdom	2018-Jan-16	EPI_ISL_302690	A/England/80380576/2018	UK Health Security Agency - Colindale	UK Health Security Agency - Colindale	Galiano,M.
EPI1194673	HA	United Kingdom	2018-Jan-18	EPI_ISL_302687	A/England/80380565/2018	UK Health Security Agency - Colindale	UK Health Security Agency - Colindale	Galiano,M.
EPI1194537	HA	United Kingdom	2018-Jan-17	EPI_ISL_302644	A/England/80380561/2018	UK Health Security Agency - Colindale	UK Health Security Agency - Colindale	Galiano,M.
EPI1194441	HA	United Kingdom	2018-Jan-16	EPI_ISL_302632	A/England/80340568/2018	UK Health Security Agency - Colindale	UK Health Security Agency - Colindale	Galiano,M.
EPI1194401	HA	United Kingdom	2018-Jan-15	EPI_ISL_302627	A/England/80340555/2018	UK Health Security Agency - Colindale	UK Health Security Agency - Colindale	Galiano,M.
EPI1194313	HA	United Kingdom	2018-Jan-12	EPI_ISL_302616	A/England/80320541/2018	UK Health Security Agency - Colindale	UK Health Security Agency - Colindale	Galiano,M.
EPI1194273	HA	United Kingdom	2018-Jan-10	EPI_ISL_302611	A/England/80260726/2018	UK Health Security Agency - Colindale	UK Health Security Agency - Colindale	Galiano,M.
EPI1194249	HA	United Kingdom	2018-Jan-09	EPI_ISL_302608	A/England/80260626/2018	UK Health Security Agency - Colindale	UK Health Security Agency - Colindale	Galiano,M.
EPI1194241	HA	United Kingdom	2018-Jan-08	EPI_ISL_302607	A/England/80260616/2018	UK Health Security Agency - Colindale	UK Health Security Agency - Colindale	Galiano,M.
EPI1194233	HA	United Kingdom	2018-Jan-09	EPI_ISL_302606	A/England/80260596/2018	UK Health Security Agency - Colindale	UK Health Security Agency - Colindale	Galiano,M.
EPI1194185	HA	United Kingdom	2018-Jan-09	EPI_ISL_302600	A/England/80240352/2018	UK Health Security Agency - Colindale	UK Health Security Agency - Colindale	Galiano,M.
EPI1174019	HA	United Kingdom	2018-Jan-15	EPI_ISL_298307	A/England/80340463/2018	UK Health Security Agency - Colindale	UK Health Security Agency - Colindale	Galiano,M.
EPI1174005	HA	United Kingdom	2018-Jan-10	EPI_ISL_298299	A/England/80280652/2018	UK Health Security Agency - Colindale	UK Health Security Agency - Colindale	Galiano,M.

Segment ID	Segment	Country	Collection date	Isolate-ID	Isolate name	Originating Lab	Submitting Lab	Authors
EPI1173999	HA	United Kingdom	2018-Jan-05	EPI_ISL_298296	A/England/80260473/2018	UK Health Security Agency - Colindale	UK Health Security Agency - Colindale	Galiano,M.
EPI1173993	HA	United Kingdom	2017-Dec-28	EPI_ISL_298293	A/England/80180647/2017	UK Health Security Agency - Colindale	UK Health Security Agency - Colindale	Galiano,M.
EPI1173979	HA	United Kingdom	2018-Jan-02	EPI_ISL_298286	A/England/80120218/2018	UK Health Security Agency - Colindale	UK Health Security Agency - Colindale	Galiano,M.
EPI1173798	HA	United Kingdom	2018-Jan-15	EPI_ISL_298109	A/England/80340462/2018	UK Health Security Agency - Colindale	UK Health Security Agency - Colindale	Galiano,M.
EPI1173789	HA	United Kingdom	2018-Jan-14	EPI_ISL_298106	A/England/80340461/2018	UK Health Security Agency - Colindale	UK Health Security Agency - Colindale	Galiano,M.
EPI1173780	HA	United Kingdom	2018-Jan-14	EPI_ISL_298105	A/England/80340457/2018	UK Health Security Agency - Colindale	UK Health Security Agency - Colindale	Galiano,M.
EPI1173756	HA	United Kingdom	2018-Jan-11	EPI_ISL_298102	A/England/80320668/2018	UK Health Security Agency - Colindale	UK Health Security Agency - Colindale	Galiano,M.
EPI1173740	HA	United Kingdom	2018-Jan-11	EPI_ISL_298100	A/England/80300540/2018	UK Health Security Agency - Colindale	UK Health Security Agency - Colindale	Galiano,M.
EPI1173732	HA	United Kingdom	2018-Jan-10	EPI_ISL_298099	A/England/80280654/2018	UK Health Security Agency - Colindale	UK Health Security Agency - Colindale	Galiano,M.
EPI1173724	HA	United Kingdom	2018-Jan-09	EPI_ISL_298098	A/England/80280649/2018	UK Health Security Agency - Colindale	UK Health Security Agency - Colindale	Galiano,M.
EPI1173708	HA	United Kingdom	2018-Jan-09	EPI_ISL_298096	A/England/80280647/2018	UK Health Security Agency - Colindale	UK Health Security Agency - Colindale	Galiano,M.
EPI1173700	HA	United Kingdom	2018-Jan-10	EPI_ISL_298095	A/England/80280646/2018	UK Health Security Agency - Colindale	UK Health Security Agency - Colindale	Galiano,M.
EPI1173684	HA	United Kingdom	2018-Jan-03	EPI_ISL_298093	A/England/80260509/2018	UK Health Security Agency - Colindale	UK Health Security Agency - Colindale	Galiano,M.

Segment ID	Segment	Country	Collection date	Isolate-ID	Isolate name	Originating Lab	Submitting Lab	Authors
EPI1173668	HA	United Kingdom	2017-Dec-31	EPI_ISL_298091	A/England/80260505/2017	UK Health Security Agency - Colindale	UK Health Security Agency - Colindale	Galiano,M.
EPI1173644	HA	United Kingdom	2018-Jan-01	EPI_ISL_298088	A/England/80260494/2018	UK Health Security Agency - Colindale	UK Health Security Agency - Colindale	Galiano,M.
EPI1173636	HA	United Kingdom	2018-Jan-11	EPI_ISL_298087	A/England/80260493/2018	UK Health Security Agency - Colindale	UK Health Security Agency - Colindale	Galiano,M.
EPI1173628	HA	United Kingdom	2017-Dec-30	EPI_ISL_298086	A/England/80260482/2017	UK Health Security Agency - Colindale	UK Health Security Agency - Colindale	Galiano,M.
EPI1173612	HA	United Kingdom	2017-Dec-31	EPI_ISL_298084	A/England/80260475/2017	UK Health Security Agency - Colindale	UK Health Security Agency - Colindale	Galiano,M.
EPI1173604	HA	United Kingdom	2018-Jan-04	EPI_ISL_298083	A/England/80240633/2017	UK Health Security Agency - Colindale	UK Health Security Agency - Colindale	Galiano,M.
EPI1173524	HA	United Kingdom	2018-Jan-08	EPI_ISL_298073	A/England/80220360/2018	UK Health Security Agency - Colindale	UK Health Security Agency - Colindale	Galiano,M.
EPI1173388	HA	United Kingdom	2017-Dec-29	EPI_ISL_298056	A/England/80180628/2017	UK Health Security Agency - Colindale	UK Health Security Agency - Colindale	Galiano,M.
EPI1173292	HA	United Kingdom	2017-Dec-29	EPI_ISL_298044	A/England/80140583/2017	UK Health Security Agency - Colindale	UK Health Security Agency - Colindale	Galiano,M.
EPI1173276	HA	United Kingdom	2018-Jan-02	EPI_ISL_298042	A/England/80140578/2018	UK Health Security Agency - Colindale	UK Health Security Agency - Colindale	Galiano,M.
EPI1173236	HA	United Kingdom	2017-Dec-29	EPI_ISL_298037	A/England/80120221/2017	UK Health Security Agency - Colindale	UK Health Security Agency - Colindale	Galiano,M.
EPI1173204	HA	United Kingdom	2017-Dec-29	EPI_ISL_298033	A/England/80120210/2017	UK Health Security Agency - Colindale	UK Health Security Agency - Colindale	Galiano,M.
EPI1173188	HA	United Kingdom	2017-Dec-29	EPI_ISL_298031	A/England/80120203/2017	UK Health Security Agency - Colindale	UK Health Security Agency - Colindale	Galiano,M.

Segment ID	Segment	Country	Collection date	Isolate-ID	Isolate name	Originating Lab	Submitting Lab	Authors
EPI1173076	HA	United Kingdom	2017-Dec-25	EPI_ISL_298017	A/England/75280623/2017	UK Health Security Agency - Colindale	UK Health Security Agency - Colindale	Galiano,M.
EPI1173052	HA	United Kingdom	2017-Dec-19	EPI_ISL_298014	A/England/75180420/2017	UK Health Security Agency - Colindale	UK Health Security Agency - Colindale	Galiano,M.
EPI1173044	HA	United Kingdom	2017-Dec-14	EPI_ISL_298013	A/England/75160383/2017	UK Health Security Agency - Colindale	UK Health Security Agency - Colindale	Galiano,M.
EPI1173036	HA	United Kingdom	2017-Nov-20	EPI_ISL_298012	A/England/74820681/2017	UK Health Security Agency - Colindale	UK Health Security Agency - Colindale	Galiano,M.
EPI1173028	HA	United Kingdom	2017-Nov-21	EPI_ISL_298011	A/England/74820679/2017	UK Health Security Agency - Colindale	UK Health Security Agency - Colindale	Galiano,M.
EPI1152758	HA	United Kingdom	2018-Jan-02	EPI_ISL_293738	A/England/80220585/2018	UK Health Security Agency - Colindale	UK Health Security Agency - Colindale	Galiano,M.
EPI1152756	HA	United Kingdom	2018-Jan-04	EPI_ISL_293737	A/England/80220576/2018	UK Health Security Agency - Colindale	UK Health Security Agency - Colindale	Galiano,M.
EPI1152754	HA	United Kingdom	2017-Dec-31	EPI_ISL_293736	A/England/80160689/2017	UK Health Security Agency - Colindale	UK Health Security Agency - Colindale	Galiano,M.
EPI1152736	HA	United Kingdom	2017-Dec-27	EPI_ISL_293726	A/England/75280376/2017	UK Health Security Agency - Colindale	UK Health Security Agency - Colindale	Galiano,M.
EPI1152711	HA	United Kingdom	2018-Jan-07	EPI_ISL_293713	A/England/80220597/2018	UK Health Security Agency - Colindale	UK Health Security Agency - Colindale	Galiano,M.
EPI1152695	HA	United Kingdom	2018-Jan-05	EPI_ISL_293711	A/England/80220578/2018	UK Health Security Agency - Colindale	UK Health Security Agency - Colindale	Galiano,M.
EPI1152687	HA	United Kingdom	2018-Jan-03	EPI_ISL_293710	A/England/80200502/2018	UK Health Security Agency - Colindale	UK Health Security Agency - Colindale	Galiano,M.
EPI1152671	HA	United Kingdom	2018-Jan-03	EPI_ISL_293708	A/England/80200500/2018	UK Health Security Agency - Colindale	UK Health Security Agency - Colindale	Galiano,M.

Segment ID	Segment	Country	Collection date	Isolate-ID	Isolate name	Originating Lab	Submitting Lab	Authors
EPI1152631	HA	United Kingdom	2018-Jan-03	EPI_ISL_293703	A/England/80200464/2018	UK Health Security Agency - Colindale	UK Health Security Agency - Colindale	Galiano,M.
EPI1152623	HA	United Kingdom	2017-Dec-31	EPI_ISL_293702	A/England/80200461/2017	UK Health Security Agency - Colindale	UK Health Security Agency - Colindale	Galiano,M.
EPI1152615	HA	United Kingdom	2017-Nov-27	EPI_ISL_293701	A/England/80200460/2017	UK Health Security Agency - Colindale	UK Health Security Agency - Colindale	Galiano,M.
EPI1152607	HA	United Kingdom	2017-Dec-30	EPI_ISL_293700	A/England/80200458/2017	UK Health Security Agency - Colindale	UK Health Security Agency - Colindale	Galiano,M.
EPI1152599	HA	United Kingdom	2017-Dec-24	EPI_ISL_293699	A/England/80200455/2017	UK Health Security Agency - Colindale	UK Health Security Agency - Colindale	Galiano,M.
EPI1152591	HA	United Kingdom	2017-Dec-23	EPI_ISL_293698	A/England/80200453/2017	UK Health Security Agency - Colindale	UK Health Security Agency - Colindale	Galiano,M.
EPI1152575	HA	United Kingdom	2017-Dec-20	EPI_ISL_293696	A/England/80200447/2017	UK Health Security Agency - Colindale	UK Health Security Agency - Colindale	Galiano,M.
EPI1152567	HA	United Kingdom	2017-Dec-22	EPI_ISL_293695	A/England/80200446/2017	UK Health Security Agency - Colindale	UK Health Security Agency - Colindale	Galiano,M.
EPI1152558	HA	United Kingdom	2017-Dec-30	EPI_ISL_293692	A/England/80180627/2017	UK Health Security Agency - Colindale	UK Health Security Agency - Colindale	Galiano,M.
EPI1152547	HA	United Kingdom	2017-Dec-31	EPI_ISL_293691	A/England/80180624/2017	UK Health Security Agency - Colindale	UK Health Security Agency - Colindale	Galiano,M.
EPI1152539	HA	United Kingdom	2017-Dec-25	EPI_ISL_293690	A/England/80160688/2017	UK Health Security Agency - Colindale	UK Health Security Agency - Colindale	Galiano,M.
EPI1152531	HA	United Kingdom	2017-Dec-28	EPI_ISL_293689	A/England/80160678/2017	UK Health Security Agency - Colindale	UK Health Security Agency - Colindale	Galiano,M.
EPI1152507	HA	United Kingdom	2017-Dec-28	EPI_ISL_293686	A/England/80160674/2017	UK Health Security Agency - Colindale	UK Health Security Agency - Colindale	Galiano,M.

Segment ID	Segment	Country	Collection date	Isolate-ID	Isolate name	Originating Lab	Submitting Lab	Authors
EPI1152499	HA	United Kingdom	2017-Dec-27	EPI_ISL_293685	A/England/80160673/2017	UK Health Security Agency - Colindale	UK Health Security Agency - Colindale	Galiano,M.
EPI1152475	HA	United Kingdom	2017-Dec-28	EPI_ISL_293682	A/England/80160654/2017	UK Health Security Agency - Colindale	UK Health Security Agency - Colindale	Galiano,M.
EPI1152467	HA	United Kingdom	2017-Dec-29	EPI_ISL_293681	A/England/80140833/2017	UK Health Security Agency - Colindale	UK Health Security Agency - Colindale	Galiano,M.
EPI1152459	HA	United Kingdom	2017-Dec-21	EPI_ISL_293680	A/England/75280704/2017	UK Health Security Agency - Colindale	UK Health Security Agency - Colindale	Galiano,M.
EPI1152451	HA	United Kingdom	2017-Dec-21	EPI_ISL_293679	A/England/75280703/2017	UK Health Security Agency - Colindale	UK Health Security Agency - Colindale	Galiano,M.
EPI1152435	HA	United Kingdom	2017-Dec-23	EPI_ISL_293677	A/England/75280699/2017	UK Health Security Agency - Colindale	UK Health Security Agency - Colindale	Galiano,M.
EPI1152387	HA	United Kingdom	2017-Dec-23	EPI_ISL_293671	A/England/75280649/2017	UK Health Security Agency - Colindale	UK Health Security Agency - Colindale	Galiano,M.
EPI1152379	HA	United Kingdom	2017-Dec-18	EPI_ISL_293670	A/England/75280639/2017	UK Health Security Agency - Colindale	UK Health Security Agency - Colindale	Galiano,M.
EPI1152339	HA	United Kingdom	2017-Dec-27	EPI_ISL_293665	A/England/75280380/2017	UK Health Security Agency - Colindale	UK Health Security Agency - Colindale	Galiano,M.
EPI1152323	HA	United Kingdom	2017-Dec-28	EPI_ISL_293663	A/England/75260844/2017	UK Health Security Agency - Colindale	UK Health Security Agency - Colindale	Galiano,M.
EPI1152315	HA	United Kingdom	2017-Dec-28	EPI_ISL_293662	A/England/75260841/2017	UK Health Security Agency - Colindale	UK Health Security Agency - Colindale	Galiano,M.
EPI1152299	HA	United Kingdom	2017-Dec-27	EPI_ISL_293660	A/England/75260395/2017	UK Health Security Agency - Colindale	UK Health Security Agency - Colindale	Galiano,M.
EPI1152291	HA	United Kingdom	2017-Dec-27	EPI_ISL_293659	A/England/75260318/2017	UK Health Security Agency - Colindale	UK Health Security Agency - Colindale	Galiano,M.

Segment ID	Segment	Country	Collection date	Isolate-ID	Isolate name	Originating Lab	Submitting Lab	Authors
EPI1152275	HA	United Kingdom	2017-Dec-27	EPI_ISL_293657	A/England/75260312/2017	UK Health Security Agency - Colindale	UK Health Security Agency - Colindale	Galiano,M.
EPI1152251	HA	United Kingdom	2017-Dec-20	EPI_ISL_293654	A/England/75240075/2017	UK Health Security Agency - Colindale	UK Health Security Agency - Colindale	Galiano,M.
EPI1152243	HA	United Kingdom	2017-Dec-22	EPI_ISL_293653	A/England/75240073/2017	UK Health Security Agency - Colindale	UK Health Security Agency - Colindale	Galiano,M.
EPI1152227	HA	United Kingdom	2017-Dec-22	EPI_ISL_293651	A/England/75240051/2017	UK Health Security Agency - Colindale	UK Health Security Agency - Colindale	Galiano,M.
EPI1152171	HA	United Kingdom	2017-Dec-20	EPI_ISL_293644	A/England/75180417/2017	UK Health Security Agency - Colindale	UK Health Security Agency - Colindale	Galiano,M.
EPI1152083	HA	United Kingdom	2017-Dec-21	EPI_ISL_293633	A/England/75180387/2017	UK Health Security Agency - Colindale	UK Health Security Agency - Colindale	Galiano,M.
EPI1152027	HA	United Kingdom	2017-Dec-19	EPI_ISL_293626	A/England/75160360/2017	UK Health Security Agency - Colindale	UK Health Security Agency - Colindale	Galiano,M.
EPI1145040	HA	United Kingdom	2017-Dec-22	EPI_ISL_292322	A/England/75260825/2017	UK Health Security Agency - Colindale	UK Health Security Agency - Colindale	Galiano,M.
EPI1145021	HA	United Kingdom	2017-Dec-20	EPI_ISL_292311	A/England/75260846/2017	UK Health Security Agency - Colindale	UK Health Security Agency - Colindale	Galiano,M.
EPI1144997	HA	United Kingdom	2017-Dec-28	EPI_ISL_292308	A/England/75260839/2017	UK Health Security Agency - Colindale	UK Health Security Agency - Colindale	Galiano,M.
EPI1144989	HA	United Kingdom	2017-Dec-19	EPI_ISL_292307	A/England/75260837/2017	UK Health Security Agency - Colindale	UK Health Security Agency - Colindale	Galiano,M.
EPI1144981	HA	United Kingdom	2017-Dec-20	EPI_ISL_292306	A/England/75260836/2017	UK Health Security Agency - Colindale	UK Health Security Agency - Colindale	Galiano,M.
EPI1144973	HA	United Kingdom	2017-Dec-20	EPI_ISL_292305	A/England/75260834/2017	UK Health Security Agency - Colindale	UK Health Security Agency - Colindale	Galiano,M.

Segment ID	Segment	Country	Collection date	Isolate-ID	Isolate name	Originating Lab	Submitting Lab	Authors
EPI1144965	HA	United Kingdom	2017-Dec-20	EPI_ISL_292304	A/England/75260833/2017	UK Health Security Agency - Colindale	UK Health Security Agency - Colindale	Galiano,M.
EPI1144957	HA	United Kingdom	2017-Dec-21	EPI_ISL_292303	A/England/75260832/2017	UK Health Security Agency - Colindale	UK Health Security Agency - Colindale	Galiano,M.
EPI1144941	HA	United Kingdom	2017-Dec-25	EPI_ISL_292301	A/England/75260828/2017	UK Health Security Agency - Colindale	UK Health Security Agency - Colindale	Galiano,M.
EPI1144933	HA	United Kingdom	2017-Dec-23	EPI_ISL_292300	A/England/75260827/2017	UK Health Security Agency - Colindale	UK Health Security Agency - Colindale	Galiano,M.
EPI1144925	HA	United Kingdom	2017-Dec-25	EPI_ISL_292299	A/England/75260826/2017	UK Health Security Agency - Colindale	UK Health Security Agency - Colindale	Galiano,M.
EPI1144885	HA	United Kingdom	2017-Dec-17	EPI_ISL_292294	A/England/75260820/2017	UK Health Security Agency - Colindale	UK Health Security Agency - Colindale	Galiano,M.
EPI1144877	HA	United Kingdom	2017-Dec-17	EPI_ISL_292293	A/England/75260818/2017	UK Health Security Agency - Colindale	UK Health Security Agency - Colindale	Galiano,M.
EPI1144869	HA	United Kingdom	2017-Dec-17	EPI_ISL_292292	A/England/75260817/2017	UK Health Security Agency - Colindale	UK Health Security Agency - Colindale	Galiano,M.
EPI1144861	HA	United Kingdom	2017-Dec-19	EPI_ISL_292291	A/England/75260816/2017	UK Health Security Agency - Colindale	UK Health Security Agency - Colindale	Galiano,M.
EPI1144853	HA	United Kingdom	2017-Dec-28	EPI_ISL_292290	A/England/75260815/2017	UK Health Security Agency - Colindale	UK Health Security Agency - Colindale	Galiano,M.
EPI1144845	HA	United Kingdom	2017-Dec-28	EPI_ISL_292289	A/England/75260814/2017	UK Health Security Agency - Colindale	UK Health Security Agency - Colindale	Galiano,M.
EPI1144829	HA	United Kingdom	2017-Dec-19	EPI_ISL_292287	A/England/75260811/2017	UK Health Security Agency - Colindale	UK Health Security Agency - Colindale	Galiano,M.
EPI1144821	HA	United Kingdom	2017-Dec-19	EPI_ISL_292286	A/England/75260810/2017	UK Health Security Agency - Colindale	UK Health Security Agency - Colindale	Galiano,M.

Segment ID	Segment	Country	Collection date	Isolate-ID	Isolate name	Originating Lab	Submitting Lab	Authors
EPI1144813	HA	United Kingdom	2017-Dec-19	EPI_ISL_292285	A/England/75260808/2017	UK Health Security Agency - Colindale	UK Health Security Agency - Colindale	Galiano,M.
EPI1144805	HA	United Kingdom	2017-Dec-19	EPI_ISL_292284	A/England/75260807/2017	UK Health Security Agency - Colindale	UK Health Security Agency - Colindale	Galiano,M.
EPI1144789	HA	United Kingdom	2017-Dec-18	EPI_ISL_292282	A/England/75260796/2017	UK Health Security Agency - Colindale	UK Health Security Agency - Colindale	Galiano,M.
EPI1144757	HA	United Kingdom	2017-Dec-20	EPI_ISL_292278	A/England/75260783/2017	UK Health Security Agency - Colindale	UK Health Security Agency - Colindale	Galiano,M.
EPI1144749	HA	United Kingdom	2017-Dec-28	EPI_ISL_292277	A/England/75260779/2017	UK Health Security Agency - Colindale	UK Health Security Agency - Colindale	Galiano,M.
EPI1144733	HA	United Kingdom	2017-Dec-19	EPI_ISL_292275	A/England/75160441/2017	UK Health Security Agency - Colindale	UK Health Security Agency - Colindale	Galiano,M.
EPI1144725	HA	United Kingdom	2017-Dec-18	EPI_ISL_292274	A/England/75160399/2017	UK Health Security Agency - Colindale	UK Health Security Agency - Colindale	Galiano,M.
EPI1144717	HA	United Kingdom	2017-Dec-16	EPI_ISL_292273	A/England/75160396/2017	UK Health Security Agency - Colindale	UK Health Security Agency - Colindale	Galiano,M.
EPI1144709	HA	United Kingdom	2017-Dec-14	EPI_ISL_292272	A/England/75160395/2017	UK Health Security Agency - Colindale	UK Health Security Agency - Colindale	Galiano,M.
EPI1144701	HA	United Kingdom	2017-Dec-16	EPI_ISL_292271	A/England/75160393/2017	UK Health Security Agency - Colindale	UK Health Security Agency - Colindale	Galiano,M.
EPI1144693	HA	United Kingdom	2017-Dec-16	EPI_ISL_292270	A/England/75160392/2017	UK Health Security Agency - Colindale	UK Health Security Agency - Colindale	Galiano,M.
EPI1144685	HA	United Kingdom	2017-Dec-16	EPI_ISL_292269	A/England/75160390/2017	UK Health Security Agency - Colindale	UK Health Security Agency - Colindale	Galiano,M.
EPI1144677	HA	United Kingdom	2017-Dec-18	EPI_ISL_292268	A/England/75160389/2017	UK Health Security Agency - Colindale	UK Health Security Agency - Colindale	Galiano,M.

Segment ID	Segment	Country	Collection date	Isolate-ID	Isolate name	Originating Lab	Submitting Lab	Authors
EPI1144669	HA	United Kingdom	2017-Dec-12	EPI_ISL_292267	A/England/75160388/2017	UK Health Security Agency - Colindale	UK Health Security Agency - Colindale	Galiano,M.
EPI1144661	HA	United Kingdom	2017-Dec-17	EPI_ISL_292266	A/England/75160387/2017	UK Health Security Agency - Colindale	UK Health Security Agency - Colindale	Galiano,M.
EPI1144645	HA	United Kingdom	2017-Dec-16	EPI_ISL_292264	A/England/75160384/2017	UK Health Security Agency - Colindale	UK Health Security Agency - Colindale	Galiano,M.
EPI1144613	HA	United Kingdom	2017-Nov-22	EPI_ISL_292260	A/England/75120454/2017	UK Health Security Agency - Colindale	UK Health Security Agency - Colindale	Galiano,M.
EPI1144589	HA	United Kingdom	2017-Dec-11	EPI_ISL_292257	A/England/75100513/2017	UK Health Security Agency - Colindale	UK Health Security Agency - Colindale	Galiano,M.
EPI1144581	HA	United Kingdom	2017-Dec-11	EPI_ISL_292256	A/England/75100509/2017	UK Health Security Agency - Colindale	UK Health Security Agency - Colindale	Galiano,M.
EPI1144573	HA	United Kingdom	2017-Dec-14	EPI_ISL_292255	A/England/75060561/2017	UK Health Security Agency - Colindale	UK Health Security Agency - Colindale	Galiano,M.
EPI1144557	HA	United Kingdom	2017-Dec-09	EPI_ISL_292253	A/England/75060552/2017	UK Health Security Agency - Colindale	UK Health Security Agency - Colindale	Galiano,M.
EPI1144549	HA	United Kingdom	2017-Dec-12	EPI_ISL_292252	A/England/75060551/2017	UK Health Security Agency - Colindale	UK Health Security Agency - Colindale	Galiano,M.
EPI1144541	HA	United Kingdom	2017-Dec-08	EPI_ISL_292251	A/England/75040600/2017	UK Health Security Agency - Colindale	UK Health Security Agency - Colindale	Galiano,M.
EPI1144533	HA	United Kingdom	2017-Dec-08	EPI_ISL_292250	A/England/75040599/2017	UK Health Security Agency - Colindale	UK Health Security Agency - Colindale	Galiano,M.
EPI1144525	HA	United Kingdom	2017-Dec-08	EPI_ISL_292249	A/England/75040598/2017	UK Health Security Agency - Colindale	UK Health Security Agency - Colindale	Galiano,M.
EPI1144517	HA	United Kingdom	2017-Dec-05	EPI_ISL_292248	A/England/75020501/2017	UK Health Security Agency - Colindale	UK Health Security Agency - Colindale	Galiano,M.

Segment ID	Segment	Country	Collection date	Isolate-ID	Isolate name	Originating Lab	Submitting Lab	Authors
EPI1144485	HA	United Kingdom	2017-Dec-10	EPI_ISL_292244	A/England/75020492/2017	UK Health Security Agency - Colindale	UK Health Security Agency - Colindale	Galiano,M.
EPI1144477	HA	United Kingdom	2017-Dec-07	EPI_ISL_292243	A/England/75020491/2017	UK Health Security Agency - Colindale	UK Health Security Agency - Colindale	Galiano,M.
EPI1024846	HA	United Kingdom	2017-Mar-14	EPI_ISL_269903	A/England/71360549/2017	UK Health Security Agency - Colindale	UK Health Security Agency - Colindale	Galiano,M.
EPI1024838	HA	United Kingdom	2017-Mar-10	EPI_ISL_269902	A/England/71180496/2017	UK Health Security Agency - Colindale	UK Health Security Agency - Colindale	Galiano,M.
EPI1024830	HA	United Kingdom	2017-Feb-28	EPI_ISL_269901	A/England/70960256/2017	UK Health Security Agency - Colindale	UK Health Security Agency - Colindale	Galiano,M.
EPI1024822	HA	United Kingdom	2017-Feb-18	EPI_ISL_269900	A/England/70860421/2017	UK Health Security Agency - Colindale	UK Health Security Agency - Colindale	Galiano,M.
EPI1024814	HA	United Kingdom	2017-Feb-09	EPI_ISL_269899	A/England/28/2017	UK Health Security Agency - Colindale	UK Health Security Agency - Colindale	Galiano,M.
EPI1024806	HA	United Kingdom	2017-Feb-20	EPI_ISL_269898	A/England/70800085/2017	UK Health Security Agency - Colindale	UK Health Security Agency - Colindale	Galiano,M.
EPI1024798	HA	United Kingdom	2017-Feb-07	EPI_ISL_269897	A/England/70780308/2017	UK Health Security Agency - Colindale	UK Health Security Agency - Colindale	Galiano,M.
EPI1024790	HA	United Kingdom	2017-Feb-07	EPI_ISL_269896	A/England/70780305/2017	UK Health Security Agency - Colindale	UK Health Security Agency - Colindale	Galiano,M.
EPI1024782	HA	United Kingdom	2017-Feb-03	EPI_ISL_269895	A/England/70780304/2017	UK Health Security Agency - Colindale	UK Health Security Agency - Colindale	Galiano,M.
EPI1024774	HA	United Kingdom	2017-Feb-04	EPI_ISL_269894	A/England/70780303/2017	UK Health Security Agency - Colindale	UK Health Security Agency - Colindale	Galiano,M.
EPI1024766	HA	United Kingdom	2017-Feb-08	EPI_ISL_269893	A/England/70780302/2017	UK Health Security Agency - Colindale	UK Health Security Agency - Colindale	Galiano,M.

Segment ID	Segment	Country	Collection date	Isolate-ID	Isolate name	Originating Lab	Submitting Lab	Authors
EPI1024758	HA	United Kingdom	2017-Feb-08	EPI_ISL_269892	A/England/70780301/2017	UK Health Security Agency - Colindale	UK Health Security Agency - Colindale	Galiano,M.
EPI1024750	HA	United Kingdom	2017-Feb-13	EPI_ISL_269891	A/England/27/2017	UK Health Security Agency - Colindale	UK Health Security Agency - Colindale	Galiano,M.
EPI1024742	HA	United Kingdom	2017-Feb-15	EPI_ISL_269890	A/England/70740083/2017	UK Health Security Agency - Colindale	UK Health Security Agency - Colindale	Galiano,M.
EPI1024734	HA	United Kingdom	2017-Feb-10	EPI_ISL_269889	A/England/70700088/2017	UK Health Security Agency - Colindale	UK Health Security Agency - Colindale	Galiano,M.
EPI1024726	HA	United Kingdom	2017-Feb-10	EPI_ISL_269888	A/England/70680145/2017	UK Health Security Agency - Colindale	UK Health Security Agency - Colindale	Galiano,M.
EPI1024718	HA	United Kingdom	2017-Feb-08	EPI_ISL_269887	A/England/70680139/2017	UK Health Security Agency - Colindale	UK Health Security Agency - Colindale	Galiano,M.
EPI1024710	HA	United Kingdom	2017-Feb-08	EPI_ISL_269886	A/England/70660207/2017	UK Health Security Agency - Colindale	UK Health Security Agency - Colindale	Galiano,M.
EPI1024702	HA	United Kingdom	2017-Feb-06	EPI_ISL_269885	A/England/70640091/2017	UK Health Security Agency - Colindale	UK Health Security Agency - Colindale	Galiano,M.
EPI1024694	HA	United Kingdom	2017-Feb-06	EPI_ISL_269884	A/England/70620213/2017	UK Health Security Agency - Colindale	UK Health Security Agency - Colindale	Galiano,M.
EPI1024686	HA	United Kingdom	2017-Feb-06	EPI_ISL_269883	A/England/70600113/2017	UK Health Security Agency - Colindale	UK Health Security Agency - Colindale	Galiano,M.
EPI1024678	HA	United Kingdom	2017-Feb-06	EPI_ISL_269882	A/England/70600070/2017	UK Health Security Agency - Colindale	UK Health Security Agency - Colindale	Galiano,M.
EPI1024670	HA	United Kingdom	2017-Feb-03	EPI_ISL_269881	A/England/70600063/2017	UK Health Security Agency - Colindale	UK Health Security Agency - Colindale	Galiano,M.
EPI1024662	HA	United Kingdom	2017-Feb-02	EPI_ISL_269880	A/England/70600058/2017	UK Health Security Agency - Colindale	UK Health Security Agency - Colindale	Galiano,M.

Segment ID	Segment	Country	Collection date	Isolate-ID	Isolate name	Originating Lab	Submitting Lab	Authors
EPI1024654	HA	United Kingdom	2017-Jan-31	EPI_ISL_269879	A/England/70580332/2017	UK Health Security Agency - Colindale	UK Health Security Agency - Colindale	Galiano,M.
EPI1024646	HA	United Kingdom	2017-Jan-27	EPI_ISL_269878	A/England/70580320/2017	UK Health Security Agency - Colindale	UK Health Security Agency - Colindale	Galiano,M.
EPI1024638	HA	United Kingdom	2017-Jan-28	EPI_ISL_269877	A/England/70580319/2017	UK Health Security Agency - Colindale	UK Health Security Agency - Colindale	Galiano,M.
EPI1024630	HA	United Kingdom	2017-Jan-28	EPI_ISL_269876	A/England/70580311/2017	UK Health Security Agency - Colindale	UK Health Security Agency - Colindale	Galiano,M.
EPI1024622	HA	United Kingdom	2017-Jan-29	EPI_ISL_269875	A/England/70580300/2017	UK Health Security Agency - Colindale	UK Health Security Agency - Colindale	Galiano,M.
EPI1024614	HA	United Kingdom	2017-Feb-01	EPI_ISL_269874	A/England/70580091/2017	UK Health Security Agency - Colindale	UK Health Security Agency - Colindale	Galiano,M.
EPI1024606	HA	United Kingdom	2017-Feb-02	EPI_ISL_269873	A/England/70580084/2017	UK Health Security Agency - Colindale	UK Health Security Agency - Colindale	Galiano,M.
EPI1024598	HA	United Kingdom	2017-Jan-26	EPI_ISL_269872	A/England/70560473/2017	UK Health Security Agency - Colindale	UK Health Security Agency - Colindale	Galiano,M.
EPI1024590	HA	United Kingdom	2017-Feb-02	EPI_ISL_269871	A/England/70560121/2017	UK Health Security Agency - Colindale	UK Health Security Agency - Colindale	Galiano,M.
EPI1024582	HA	United Kingdom	2017-Jan-31	EPI_ISL_269870	A/England/70560118/2017	UK Health Security Agency - Colindale	UK Health Security Agency - Colindale	Galiano,M.
EPI1024574	HA	United Kingdom	2017-Feb-02	EPI_ISL_269869	A/England/70560111/2017	UK Health Security Agency - Colindale	UK Health Security Agency - Colindale	Galiano,M.
EPI1024566	HA	United Kingdom	2017-Feb-01	EPI_ISL_269868	A/England/70560102/2017	UK Health Security Agency - Colindale	UK Health Security Agency - Colindale	Galiano,M.
EPI1024558	HA	United Kingdom	2017-Jan-30	EPI_ISL_269867	A/England/70540238/2017	UK Health Security Agency - Colindale	UK Health Security Agency - Colindale	Galiano,M.

Segment ID	Segment	Country	Collection date	Isolate-ID	Isolate name	Originating Lab	Submitting Lab	Authors
EPI1024550	HA	United Kingdom	2017-Jan-30	EPI_ISL_269866	A/England/70540225/2017	UK Health Security Agency - Colindale	UK Health Security Agency - Colindale	Galiano,M.
EPI1024542	HA	United Kingdom	2017-Jan-30	EPI_ISL_269865	A/England/70520080/2017	UK Health Security Agency - Colindale	UK Health Security Agency - Colindale	Galiano,M.
EPI1024534	HA	United Kingdom	2017-Jan-26	EPI_ISL_269864	A/England/70520072/2017	UK Health Security Agency - Colindale	UK Health Security Agency - Colindale	Galiano,M.
EPI1024526	HA	United Kingdom	2017-Jan-25	EPI_ISL_269863	A/England/70500473/2017	UK Health Security Agency - Colindale	UK Health Security Agency - Colindale	Galiano,M.
EPI1024518	HA	United Kingdom	2017-Jan-26	EPI_ISL_269862	A/England/70500067/2017	UK Health Security Agency - Colindale	UK Health Security Agency - Colindale	Galiano,M.
EPI1024510	HA	United Kingdom	2017-Jan-27	EPI_ISL_269861	A/England/70500053/2017	UK Health Security Agency - Colindale	UK Health Security Agency - Colindale	Galiano,M.
EPI1024502	HA	United Kingdom	2017-Jan-24	EPI_ISL_269860	A/England/70480381/2017	UK Health Security Agency - Colindale	UK Health Security Agency - Colindale	Galiano,M.
EPI1024494	HA	United Kingdom	2017-Jan-27	EPI_ISL_269859	A/England/70480376/2017	UK Health Security Agency - Colindale	UK Health Security Agency - Colindale	Galiano,M.
EPI1024486	HA	United Kingdom	2017-Jan-20	EPI_ISL_269858	A/England/70480370/2017	UK Health Security Agency - Colindale	UK Health Security Agency - Colindale	Galiano,M.
EPI1024478	HA	United Kingdom	2017-Jan-20	EPI_ISL_269857	A/England/70480368/2017	UK Health Security Agency - Colindale	UK Health Security Agency - Colindale	Galiano,M.
EPI1024470	HA	United Kingdom	2017-Jan-24	EPI_ISL_269856	A/England/70480367/2017	UK Health Security Agency - Colindale	UK Health Security Agency - Colindale	Galiano,M.
EPI1024462	HA	United Kingdom	2017-Jan-26	EPI_ISL_269855	A/England/70460730/2017	UK Health Security Agency - Colindale	UK Health Security Agency - Colindale	Galiano,M.
EPI1024454	HA	United Kingdom	2017-Jan-25	EPI_ISL_269854	A/England/70460721/2017	UK Health Security Agency - Colindale	UK Health Security Agency - Colindale	Galiano,M.

Segment ID	Segment	Country	Collection date	Isolate-ID	Isolate name	Originating Lab	Submitting Lab	Authors
EPI1024446	HA	United Kingdom	2017-Jan-23	EPI_ISL_269853	A/England/70440224/2017	UK Health Security Agency - Colindale	UK Health Security Agency - Colindale	Galiano,M.
EPI1024438	HA	United Kingdom	2017-Jan-22	EPI_ISL_269852	A/England/70440216/2017	UK Health Security Agency - Colindale	UK Health Security Agency - Colindale	Galiano,M.
EPI1024430	HA	United Kingdom	2017-Jan-17	EPI_ISL_269851	A/England/70380291/2017	UK Health Security Agency - Colindale	UK Health Security Agency - Colindale	Galiano,M.
EPI1024422	HA	United Kingdom	2017-Jan-18	EPI_ISL_269850	A/England/70380289/2017	UK Health Security Agency - Colindale	UK Health Security Agency - Colindale	Galiano,M.
EPI1024414	HA	United Kingdom	2017-Jan-20	EPI_ISL_269849	A/England/70380039/2017	UK Health Security Agency - Colindale	UK Health Security Agency - Colindale	Galiano,M.
EPI1024406	HA	United Kingdom	2017-Jan-17	EPI_ISL_269848	A/England/12/2017	UK Health Security Agency - Colindale	UK Health Security Agency - Colindale	Galiano,M.
EPI1024398	HA	United Kingdom	2017-Jan-19	EPI_ISL_269847	A/England/11/2017	UK Health Security Agency - Colindale	UK Health Security Agency - Colindale	Galiano,M.
EPI1024390	HA	United Kingdom	2017-Jan-17	EPI_ISL_269846	A/England/70380021/2017	UK Health Security Agency - Colindale	UK Health Security Agency - Colindale	Galiano,M.
EPI1024382	HA	United Kingdom	2017-Jan-19	EPI_ISL_269845	A/England/70360642/2017	UK Health Security Agency - Colindale	UK Health Security Agency - Colindale	Galiano,M.
EPI1024374	HA	United Kingdom	2017-Jan-16	EPI_ISL_269844	A/England/70360628/2017	UK Health Security Agency - Colindale	UK Health Security Agency - Colindale	Galiano,M.
EPI1024366	HA	United Kingdom	2017-Jan-17	EPI_ISL_269843	A/England/70360615/2017	UK Health Security Agency - Colindale	UK Health Security Agency - Colindale	Galiano,M.
EPI1024358	HA	United Kingdom	2017-Jan-18	EPI_ISL_269842	A/England/70360040/2017	UK Health Security Agency - Colindale	UK Health Security Agency - Colindale	Galiano,M.
EPI1024350	HA	United Kingdom	2017-Jan-18	EPI_ISL_269841	A/England/70340165/2017	UK Health Security Agency - Colindale	UK Health Security Agency - Colindale	Galiano,M.

Segment ID	Segment	Country	Collection date	Isolate-ID	Isolate name	Originating Lab	Submitting Lab	Authors
EPI1024342	HA	United Kingdom	2017-Jan-11	EPI_ISL_269840	A/England/70320386/2017	UK Health Security Agency - Colindale	UK Health Security Agency - Colindale	Galiano,M.
EPI1024334	HA	United Kingdom	2017-Jan-16	EPI_ISL_269839	A/England/70320045/2017	UK Health Security Agency - Colindale	UK Health Security Agency - Colindale	Galiano,M.
EPI1024326	HA	United Kingdom	2017-Jan-16	EPI_ISL_269838	A/England/70320041/2017	UK Health Security Agency - Colindale	UK Health Security Agency - Colindale	Galiano,M.
EPI1024318	HA	United Kingdom	2017-Jan-17	EPI_ISL_269837	A/England/70320037/2017	UK Health Security Agency - Colindale	UK Health Security Agency - Colindale	Galiano,M.
EPI1024310	HA	United Kingdom	2017-Jan-17	EPI_ISL_269836	A/England/70320035/2017	UK Health Security Agency - Colindale	UK Health Security Agency - Colindale	Galiano,M.
EPI1024302	HA	United Kingdom	2017-Jan-16	EPI_ISL_269835	A/England/70300018/2017	UK Health Security Agency - Colindale	UK Health Security Agency - Colindale	Galiano,M.
EPI1024294	HA	United Kingdom	2017-Jan-11	EPI_ISL_269834	A/England/70300012/2017	UK Health Security Agency - Colindale	UK Health Security Agency - Colindale	Galiano,M.
EPI1024286	HA	United Kingdom	2017-Jan-13	EPI_ISL_269833	A/England/70280046/2017	UK Health Security Agency - Colindale	UK Health Security Agency - Colindale	Galiano,M.
EPI1024278	HA	United Kingdom	2017-Jan-11	EPI_ISL_269832	A/England/70260700/2017	UK Health Security Agency - Colindale	UK Health Security Agency - Colindale	Galiano,M.
EPI1024270	HA	United Kingdom	2017-Jan-10	EPI_ISL_269831	A/England/70260036/2017	UK Health Security Agency - Colindale	UK Health Security Agency - Colindale	Galiano,M.
EPI1024262	HA	United Kingdom	2017-Jan-10	EPI_ISL_269830	A/England/70260035/2017	UK Health Security Agency - Colindale	UK Health Security Agency - Colindale	Galiano,M.
EPI1024254	HA	United Kingdom	2017-Jan-10	EPI_ISL_269829	A/England/70260031/2017	UK Health Security Agency - Colindale	UK Health Security Agency - Colindale	Galiano,M.
EPI1024246	HA	United Kingdom	2017-Jan-11	EPI_ISL_269828	A/England/70240803/2017	UK Health Security Agency - Colindale	UK Health Security Agency - Colindale	Galiano,M.

Segment ID	Segment	Country	Collection date	Isolate-ID	Isolate name	Originating Lab	Submitting Lab	Authors
EPI1024238	HA	United Kingdom	2017-Jan-09	EPI_ISL_269827	A/England/70240083/2017	UK Health Security Agency - Colindale	UK Health Security Agency - Colindale	Galiano,M.
EPI1024230	HA	United Kingdom	2016-Dec-27	EPI_ISL_269826	A/England/70180218/2016	UK Health Security Agency - Colindale	UK Health Security Agency - Colindale	Galiano,M.
EPI1024222	HA	United Kingdom	2016-Dec-21	EPI_ISL_269825	A/England/70180196/2016	UK Health Security Agency - Colindale	UK Health Security Agency - Colindale	Galiano,M.
EPI1024214	HA	United Kingdom	2017-Jan-04	EPI_ISL_269824	A/England/70180023/2017	UK Health Security Agency - Colindale	UK Health Security Agency - Colindale	Galiano,M.
EPI1024206	HA	United Kingdom	2017-Jan-06	EPI_ISL_269823	A/England/70180006/2017	UK Health Security Agency - Colindale	UK Health Security Agency - Colindale	Galiano,M.
EPI1024198	HA	United Kingdom	2016-Dec-30	EPI_ISL_269822	A/England/70160650/2016	UK Health Security Agency - Colindale	UK Health Security Agency - Colindale	Galiano,M.
EPI1024190	HA	United Kingdom	2017-Jan-05	EPI_ISL_269821	A/England/70160045/2017	UK Health Security Agency - Colindale	UK Health Security Agency - Colindale	Galiano,M.
EPI1024182	HA	United Kingdom	2016-Dec-29	EPI_ISL_269820	A/England/70140168/2016	UK Health Security Agency - Colindale	UK Health Security Agency - Colindale	Galiano,M.
EPI1024174	HA	United Kingdom	2016-Dec-30	EPI_ISL_269819	A/England/70120321/2016	UK Health Security Agency - Colindale	UK Health Security Agency - Colindale	Galiano,M.
EPI1024166	HA	United Kingdom	2016-Dec-28	EPI_ISL_269818	A/England/70120299/2016	UK Health Security Agency - Colindale	UK Health Security Agency - Colindale	Galiano,M.
EPI1024158	HA	United Kingdom	2016-Dec-23	EPI_ISL_269817	A/England/973/2016	UK Health Security Agency - Colindale	UK Health Security Agency - Colindale	Galiano,M.
EPI1024150	HA	United Kingdom	2016-Dec-28	EPI_ISL_269816	A/England/70120293/2016	UK Health Security Agency - Colindale	UK Health Security Agency - Colindale	Galiano,M.
EPI1024142	HA	United Kingdom	2017-Jan-03	EPI_ISL_269815	A/England/70120011/2017	UK Health Security Agency - Colindale	UK Health Security Agency - Colindale	Galiano,M.

Segment ID	Segment	Country	Collection date	Isolate-ID	Isolate name	Originating Lab	Submitting Lab	Authors
EPI1024134	HA	United Kingdom	2017-Jan-03	EPI_ISL_269814	A/England/70120007/2017	UK Health Security Agency - Colindale	UK Health Security Agency - Colindale	Galiano,M.
EPI1024126	HA	United Kingdom	2016-Dec-28	EPI_ISL_269813	A/England/65280022/2016	UK Health Security Agency - Colindale	UK Health Security Agency - Colindale	Galiano,M.
EPI1024118	HA	United Kingdom	2016-Dec-28	EPI_ISL_269812	A/England/65280013/2016	UK Health Security Agency - Colindale	UK Health Security Agency - Colindale	Galiano,M.
EPI1024110	HA	United Kingdom	2016-Dec-28	EPI_ISL_269811	A/England/65280003/2016	UK Health Security Agency - Colindale	UK Health Security Agency - Colindale	Galiano,M.
EPI1024102	HA	United Kingdom	2016-Dec-22	EPI_ISL_269810	A/England/65260886/2016	UK Health Security Agency - Colindale	UK Health Security Agency - Colindale	Galiano,M.
EPI1024094	HA	United Kingdom	2016-Dec-21	EPI_ISL_269809	A/England/65260877/2016	UK Health Security Agency - Colindale	UK Health Security Agency - Colindale	Galiano,M.
EPI1024086	HA	United Kingdom	2016-Dec-29	EPI_ISL_269808	A/England/65260870/2016	UK Health Security Agency - Colindale	UK Health Security Agency - Colindale	Galiano,M.
EPI1024078	HA	United Kingdom	2016-Dec-21	EPI_ISL_269807	A/England/65260006/2016	UK Health Security Agency - Colindale	UK Health Security Agency - Colindale	Galiano,M.
EPI1024070	HA	United Kingdom	2016-Dec-23	EPI_ISL_269806	A/England/65180251/2016	UK Health Security Agency - Colindale	UK Health Security Agency - Colindale	Galiano,M.
EPI1024062	HA	United Kingdom	2016-Dec-19	EPI_ISL_269805	A/England/65100016/2016	UK Health Security Agency - Colindale	UK Health Security Agency - Colindale	Galiano,M.
EPI1024054	HA	United Kingdom	2016-Dec-08	EPI_ISL_269804	A/England/65080394/2016	UK Health Security Agency - Colindale	UK Health Security Agency - Colindale	Galiano,M.
EPI1024046	HA	United Kingdom	2016-Dec-15	EPI_ISL_269803	A/England/65060436/2016	UK Health Security Agency - Colindale	UK Health Security Agency - Colindale	Galiano,M.
EPI1024038	HA	United Kingdom	2016-Dec-15	EPI_ISL_269802	A/England/65060424/2016	UK Health Security Agency - Colindale	UK Health Security Agency - Colindale	Galiano,M.

Segment ID	Segment	Country	Collection date	Isolate-ID	Isolate name	Originating Lab	Submitting Lab	Authors
EPI1024030	HA	United Kingdom	2016-Dec-15	EPI_ISL_269801	A/England/65060418/2016	UK Health Security Agency - Colindale	UK Health Security Agency - Colindale	Galiano,M.
EPI1024022	HA	United Kingdom	2016-Dec-12	EPI_ISL_269800	A/England/65040090/2016	UK Health Security Agency - Colindale	UK Health Security Agency - Colindale	Galiano,M.
EPI1024014	HA	United Kingdom	2016-Dec-12	EPI_ISL_269799	A/England/65040089/2016	UK Health Security Agency - Colindale	UK Health Security Agency - Colindale	Galiano,M.
EPI1024006	HA	United Kingdom	2016-Dec-13	EPI_ISL_269798	A/England/65040087/2016	UK Health Security Agency - Colindale	UK Health Security Agency - Colindale	Galiano,M.
EPI1023998	HA	United Kingdom	2016-Dec-13	EPI_ISL_269797	A/England/65020044/2016	UK Health Security Agency - Colindale	UK Health Security Agency - Colindale	Galiano,M.
EPI1023990	HA	United Kingdom	2016-Dec-07	EPI_ISL_269796	A/England/65000035/2016	UK Health Security Agency - Colindale	UK Health Security Agency - Colindale	Galiano,M.
EPI1023982	HA	United Kingdom	2016-Dec-12	EPI_ISL_269795	A/England/65000030/2016	UK Health Security Agency - Colindale	UK Health Security Agency - Colindale	Galiano,M.
EPI1023974	HA	United Kingdom	2016-Dec-05	EPI_ISL_269794	A/England/64980542/2016	UK Health Security Agency - Colindale	UK Health Security Agency - Colindale	Galiano,M.
EPI1023966	HA	United Kingdom	2016-Dec-05	EPI_ISL_269793	A/England/64980541/2016	UK Health Security Agency - Colindale	UK Health Security Agency - Colindale	Galiano,M.
EPI1023958	HA	United Kingdom	2016-Dec-05	EPI_ISL_269792	A/England/64980537/2016	UK Health Security Agency - Colindale	UK Health Security Agency - Colindale	Galiano,M.
EPI1023950	HA	United Kingdom	2016-Dec-06	EPI_ISL_269791	A/England/64960069/2016	UK Health Security Agency - Colindale	UK Health Security Agency - Colindale	Galiano,M.
EPI1023942	HA	United Kingdom	2016-Dec-06	EPI_ISL_269790	A/England/64960061/2016	UK Health Security Agency - Colindale	UK Health Security Agency - Colindale	Galiano,M.
EPI1023934	HA	United Kingdom	2016-Nov-24	EPI_ISL_269789	A/England/64760678/2016	UK Health Security Agency - Colindale	UK Health Security Agency - Colindale	Galiano,M.

Segment ID	Segment	Country	Collection date	Isolate-ID	Isolate name	Originating Lab	Submitting Lab	Authors
EPI1023926	HA	United Kingdom	2016-Nov-08	EPI_ISL_269788	A/England/64580215/2016	UK Health Security Agency - Colindale	UK Health Security Agency - Colindale	Galiano,M.
EPI914771	HA	United Kingdom	2016-Dec-22	EPI_ISL_248572	A/England/65280696/2016	UK Health Security Agency - Colindale	UK Health Security Agency - Colindale	Galiano,M.
EPI878300	HA	United Kingdom	2016-Jul-17	EPI_ISL_241344	A/England/62960643/2016	UK Health Security Agency - Colindale	UK Health Security Agency - Colindale	Galiano,M.
EPI878204	HA	United Kingdom	2016-Jun-12	EPI_ISL_241332	A/England/62440430/2016	UK Health Security Agency - Colindale	UK Health Security Agency - Colindale	Galiano,M.
EPI878196	HA	United Kingdom	2016-Jun-12	EPI_ISL_241331	A/England/62440427/2016	UK Health Security Agency - Colindale	UK Health Security Agency - Colindale	Galiano,M.
EPI878188	HA	United Kingdom	2016-Jun-12	EPI_ISL_241330	A/England/62440424/2016	UK Health Security Agency - Colindale	UK Health Security Agency - Colindale	Galiano,M.
EPI878180	HA	United Kingdom	2016-Jun-12	EPI_ISL_241329	A/England/62440423/2016	UK Health Security Agency - Colindale	UK Health Security Agency - Colindale	Galiano,M.
EPI878172	HA	United Kingdom	2016-Jun-12	EPI_ISL_241328	A/England/62440422/2016	UK Health Security Agency - Colindale	UK Health Security Agency - Colindale	Galiano,M.
EPI878164	HA	United Kingdom	2016-Jun-12	EPI_ISL_241327	A/England/62440421/2016	UK Health Security Agency - Colindale	UK Health Security Agency - Colindale	Galiano,M.
EPI831326	HA	United Kingdom	2016-Jul-14	EPI_ISL_233165	A/England/62920695/2016	UK Health Security Agency - Colindale	UK Health Security Agency - Colindale	Ellis, Joanna
EPI829956	HA	United Kingdom	2016-Jul-07	EPI_ISL_233008	A/England/62840631/2016	UK Health Security Agency - Colindale	UK Health Security Agency - Colindale	Ellis, Joanna
EPI829949	HA	United Kingdom	2016-Jun-12	EPI_ISL_233007	A/England/62440429/2016	UK Health Security Agency - Colindale	UK Health Security Agency - Colindale	Ellis,Joanna
EPI829947	HA	United Kingdom	2016-Jun-12	EPI_ISL_233006	A/England/62440428/2016	UK Health Security Agency - Colindale	UK Health Security Agency - Colindale	Ellis, Joanna

Segment ID	Segment	Country	Collection date	Isolate-ID	Isolate name	Originating Lab	Submitting Lab	Authors
EPI829945	HA	United Kingdom	2016-Jun-12	EPI_ISL_233005	A/England/62440426/2016	UK Health Security Agency - Colindale	UK Health Security Agency - Colindale	Ellis, Joanna
EPI1191885	HA	United Kingdom	2017-Nov-07	EPI_ISL_302054	A/Wrexham/7024/2017	Molecular Diagnostics Unit	Public Health Wales Microbiology Cardiff	
EPI1299594	HA	United Kingdom	2018-Feb-23	EPI_ISL_327409	A/Cardiff/9666/2018	Public Health Wales Microbiology Cardiff	Public Health Wales Microbiology Cardiff	
EPI1299574	HA	United Kingdom	2018-Feb-21	EPI_ISL_327407	A/Abergavenny/9599/2018	Public Health Wales Microbiology Cardiff	Public Health Wales Microbiology Cardiff	
EPI1299562	HA	United Kingdom	2018-Feb-21	EPI_ISL_327405	A/Cardiff/9548/2018	Public Health Wales Microbiology Cardiff	Public Health Wales Microbiology Cardiff	
EPI1299554	HA	United Kingdom	2018-Feb-19	EPI_ISL_327404	A/Bargoed/9468/2018	Public Health Wales Microbiology Cardiff	Public Health Wales Microbiology Cardiff	
EPI1299543	HA	United Kingdom	2018-Feb-13	EPI_ISL_327402	A/Cardiff/9215/2018	Public Health Wales Microbiology Cardiff	Public Health Wales Microbiology Cardiff	
EPI1299535	HA	United Kingdom	2018-Feb-12	EPI_ISL_327401	A/Cardiff/9169/2018	Public Health Wales Microbiology Cardiff	Public Health Wales Microbiology Cardiff	
EPI1299527	HA	United Kingdom	2018-Apr-06	EPI_ISL_327400	A/Penarth/8862/2018	Public Health Wales Microbiology Cardiff	Public Health Wales Microbiology Cardiff	
EPI1299519	HA	United Kingdom	2018-Apr-06	EPI_ISL_327399	A/Cardiff/8857/2018	Public Health Wales Microbiology Cardiff	Public Health Wales Microbiology Cardiff	

Segment ID	Segment	Country	Collection date	Isolate-ID	Isolate name	Originating Lab	Submitting Lab	Authors
EPI1299504	HA	United Kingdom	2018-Mar-20	EPI_ISL_327397	A/Cardiff/8462/2018	Public Health Wales Microbiology Cardiff	Public Health Wales Microbiology Cardiff	
EPI1299494	HA	United Kingdom	2018-Mar-18	EPI_ISL_327396	A/Cowbridge/8371/2018	Public Health Wales Microbiology Cardiff	Public Health Wales Microbiology Cardiff	
EPI1299338	HA	United Kingdom	2018-Mar-13	EPI_ISL_327338	A/Penarth/8267/2018	Public Health Wales Microbiology Cardiff	Public Health Wales Microbiology Cardiff	
EPI1282051	HA	United Kingdom	2018-Jan-05	EPI_ISL_322856	A/Port Talbot/7890/2018	Public Health Wales Microbiology Cardiff	Public Health Wales Microbiology Cardiff	
EPI1224291	HA	United Kingdom	2018-Feb-28	EPI_ISL_308919	A/Port Talbot/7068/2018	Public Health Wales Microbiology Cardiff	Public Health Wales Microbiology Cardiff	
EPI1193293	HA	United Kingdom	2017-Sep-16	EPI_ISL_302486	A/Rhyl/1947/2017	Public Health Wales Microbiology Cardiff	Public Health Wales Microbiology Cardiff	
EPI1193285	HA	United Kingdom	2017-Nov-02	EPI_ISL_302485	A/Swansea/4343/2017	Public Health Wales Microbiology Cardiff	Public Health Wales Microbiology Cardiff	
EPI1193277	HA	United Kingdom	2017-Jul-10	EPI_ISL_302484	A/Cardiff/7495/2017	Public Health Wales Microbiology Cardiff	Public Health Wales Microbiology Cardiff	
EPI1193269	HA	United Kingdom	2017-Sep-12	EPI_ISL_302464	A/Aberystwyth/5413/2017	Public Health Wales Microbiology Cardiff	Public Health Wales Microbiology Cardiff	
EPI1193205	HA	United Kingdom	2017-Jul-31	EPI_ISL_302462	A/Swansea/5352/2017	Public Health Wales Microbiology Cardiff	Public Health Wales	

Segment ID	Segment	Country	Collection date	Isolate-ID	Isolate name	Originating Lab	Submitting Lab	Authors
							Microbiology Cardiff	
EPI1193197	HA	United Kingdom	2017-Oct-16	EPI_ISL_302461	A/Wrexham/4801/2017	Public Health Wales Microbiology Cardiff	Public Health Wales Microbiology Cardiff	
EPI1193179	HA	United Kingdom	2017-Oct-30	EPI_ISL_302456	A/Carmarthen/5313/2017	Public Health Wales Microbiology Cardiff	Public Health Wales Microbiology Cardiff	
EPI1192368	HA	United Kingdom	2018-Feb-09	EPI_ISL_302246	A/Swansea/3923/2018	Public Health Wales Microbiology Cardiff	Public Health Wales Microbiology Cardiff	
EPI1192362	HA	United Kingdom	2017-Oct-19	EPI_ISL_302244	A/Aberystwyth/8812/2017	Public Health Wales Microbiology Cardiff	Public Health Wales Microbiology Cardiff	
EPI1192290	HA	United Kingdom	2017-Sep-18	EPI_ISL_302187	A/Cardiff/7156/2017	Public Health Wales Microbiology Cardiff	Public Health Wales Microbiology Cardiff	
EPI1192280	HA	United Kingdom	2017-Aug-21	EPI_ISL_302186	A/Bridgend/4113/2017	Public Health Wales Microbiology Cardiff	Public Health Wales Microbiology Cardiff	
EPI1191892	HA	United Kingdom	2017-Aug-16	EPI_ISL_302057	A/Cardiff/4045/2017	Public Health Wales Microbiology Cardiff	Public Health Wales Microbiology Cardiff	
EPI1192007	HA	United Kingdom	2017-Nov-08	EPI_ISL_302087	A/Carmarthen/5184/2017		Public Health Wales Microbiology Cardiff	
EPI1270832	HA	United Kingdom	2018-Apr-12	EPI_ISL_320652	A/Northern Ireland/13591/2018	Regional Virus Laboratory, Microbiology Department, Royal Victoria Hospital	Crick Worldwide Influenza Centre	

Segment ID	Segment	Country	Collection date	Isolate-ID	Isolate name	Originating Lab	Submitting Lab	Authors
EPI1270830	HA	United Kingdom	2018-Apr-10	EPI_ISL_320651	A/Northern Ireland/13397/2018	Regional Virus Laboratory, Microbiology Department, Royal Victoria Hospital	Crick Worldwide Influenza Centre	
EPI1270828	HA	United Kingdom	2018-Apr-05	EPI_ISL_320650	A/Northern Ireland/12971/2018	Regional Virus Laboratory, Microbiology Department, Royal Victoria Hospital	Crick Worldwide Influenza Centre	
EPI1154858	HA	United Kingdom	2018-Jan-07	EPI_ISL_294242	A/Northern Ireland/18001076/2018	Regional Virus Laboratory, Microbiology Department, Royal Victoria Hospital	Crick Worldwide Influenza Centre	
EPI1154856	HA	United Kingdom	2018-Jan-04	EPI_ISL_294241	A/Northern Ireland/18000986/2018	Regional Virus Laboratory, Microbiology Department, Royal Victoria Hospital	Crick Worldwide Influenza Centre	
EPI1154854	HA	United Kingdom	2018-Jan-05	EPI_ISL_294240	A/Northern Ireland/18000962/2018	Regional Virus Laboratory, Microbiology Department, Royal Victoria Hospital	Crick Worldwide Influenza Centre	
EPI1154852	HA	United Kingdom	2018-Jan-05	EPI_ISL_294239	A/Northern Ireland/18000961/2018	Regional Virus Laboratory, Microbiology Department, Royal Victoria Hospital	Crick Worldwide Influenza Centre	
EPI1154850	HA	United Kingdom	2017-Dec-29	EPI_ISL_294238	A/Northern Ireland/17035348/2017	Regional Virus Laboratory, Microbiology Department, Royal Victoria Hospital	Crick Worldwide Influenza Centre	
EPI1154848	HA	United Kingdom	2017-Dec-28	EPI_ISL_294237	A/Northern Ireland/17035125/2017	Regional Virus Laboratory, Microbiology	Crick Worldwide Influenza Centre	

Segment ID	Segment	Country	Collection date	Isolate-ID	Isolate name	Originating Lab	Submitting Lab	Authors
						Department, Royal Victoria Hospital		
EPI1252533	HA	United Kingdom	2018-Feb-13	EPI_ISL_314164	A/England/555/2018	UK Health Security Agency - Colindale	Crick Worldwide Influenza Centre	
EPI1252531	HA	United Kingdom	2018-Feb-13	EPI_ISL_314163	A/England/554/2018	UK Health Security Agency - Colindale	Crick Worldwide Influenza Centre	
EPI1252529	HA	United Kingdom	2018-Feb-09	EPI_ISL_314162	A/England/553/2018	UK Health Security Agency - Colindale	Crick Worldwide Influenza Centre	
EPI1252526	HA	United Kingdom	2018-Mar-07	EPI_ISL_314160	A/England/540/2018	UK Health Security Agency - Colindale	Crick Worldwide Influenza Centre	
EPI1252524	HA	United Kingdom	2018-Feb-26	EPI_ISL_314159	A/England/538/2018	UK Health Security Agency - Colindale	Crick Worldwide Influenza Centre	
EPI1252522	HA	United Kingdom	2018-Feb-21	EPI_ISL_314158	A/England/535/2018	UK Health Security Agency - Colindale	Crick Worldwide Influenza Centre	
EPI1154804	HA	United Kingdom	2017-Oct-21	EPI_ISL_294215	A/England/74400479/2017	UK Health Security Agency - Colindale	Crick Worldwide Influenza Centre	
EPI1050825	HA	United Kingdom	2017-Feb-28	EPI_ISL_276267	A/England/32/2017	UK Health Security Agency - Colindale	Crick Worldwide Influenza Centre	
EPI1050823	HA	United Kingdom	2017-Feb-13	EPI_ISL_276266	A/England/30/2017	UK Health Security Agency - Colindale	Crick Worldwide Influenza Centre	
EPI1050821	HA	United Kingdom	2017-Feb-15	EPI_ISL_276265	A/England/27/2017	UK Health Security Agency - Colindale	Crick Worldwide Influenza Centre	
EPI1050819	HA	United Kingdom	2017-Jan-31	EPI_ISL_276264	A/England/19/2017	UK Health Security Agency - Colindale	Crick Worldwide Influenza Centre	
EPI1050938	HA	United Kingdom	2016-Dec-23	EPI_ISL_276318	A/London/16U833583-6_S6_L001/2016	Department of Clinical Virology, University College London Hospitals NHS Foundation Trust	University College London	
EPI1050930	HA	United Kingdom	2016-Dec-22	EPI_ISL_276317	A/London/16U833294-5_S5_L001/2016	Department of Clinical Virology, University College London Hospitals NHS Foundation Trust	University College London	
EPI1042192	HA	United Kingdom	2016-Dec-23	EPI_ISL_274392	A/London/16U828652-10_S10_L001/2016	Department of Clinical Virology, University College	University College London	

Segment ID	Segment	Country	Collection date	Isolate-ID	Isolate name	Originating Lab	Submitting Lab	Authors
						London Hospitals NHS Foundation Trust		
EPI1042184	HA	United Kingdom	2016-Sep-24	EPI_ISL_274391	A/London/16U729646-88_S40_L001/2016	Department of Clinical Virology, University College London Hospitals NHS Foundation Trust	University College London	
EPI1042176	HA	United Kingdom	2016-Sep-06	EPI_ISL_274388	A/London/16U716974-89_S41_L001/2016	Department of Clinical Virology, University College London Hospitals NHS Foundation Trust	University College London	
EPI1040812	HA	United Kingdom	2016-Dec-19	EPI_ISL_274146	A/London/16U088238-78_S30_L001/2016	Department of Clinical Virology, University College London Hospitals NHS Foundation Trust	University College London	
EPI1040804	HA	United Kingdom	2016-Dec-28	EPI_ISL_274145	A/London/16U035923-31_S31_L001/2016	Department of Clinical Virology, University College London Hospitals NHS Foundation Trust	University College London	
EPI1040515	HA	United Kingdom	2016-Dec-28	EPI_ISL_273956	A/London/16U035723-33_S33_L001/2016	Department of Clinical Virology, University College London Hospitals NHS Foundation Trust	University College London	
EPI1040507	HA	United Kingdom	2016-Dec-28	EPI_ISL_273955	A/London/16U035703-29_S29_L001/2016	Department of Clinical Virology, University College London Hospitals NHS Foundation Trust	University College London	

Segment ID	Segment	Country	Collection date	Isolate-ID	Isolate name	Originating Lab	Submitting Lab	Authors
EPI1040495	HA	United Kingdom	2016-Dec-28	EPI_ISL_273954	A/London/16U035676-27_S27_L001/2016	Department of Clinical Virology, University College London Hospitals NHS Foundation Trust	University College London	
EPI1040433	HA	United Kingdom	2016-Dec-28	EPI_ISL_273942	A/London/16U035668-28_S28_L001/2016	Department of Clinical Virology, University College London Hospitals NHS Foundation Trust	University College London	
EPI1040424	HA	United Kingdom	2016-Dec-28	EPI_ISL_273941	A/London/16U035667-26_S26_L001/2016	Department of Clinical Virology, University College London Hospitals NHS Foundation Trust	University College London	
EPI1541160	HA	United Kingdom	2018-Feb-09	EPI_ISL_376793	A/England/7211/2018			Gruner,W.E.; Fries,A.C.; Garrett,C.M.; Powell,M.L.; Minnich,D.E.; Couch,M.R.; Hanson,J.F.; DeMarcus,L.S.; Crum,M.E.; Bogue,A.L.; Federinko,S.P.; Macias,E.A.; West,C.A.; Poel,J.R.
EPI1541159	HA	United Kingdom	2018-Jan-31	EPI_ISL_376792	A/England/7210/2018			Gruner,W.E.; Fries,A.C.; Garrett,C.M.; Powell,M.L.; Minnich,D.E.; Couch,M.R.; Hanson,J.F.; DeMarcus,L.S.; Crum,M.E.; Bogue,A.L.; Federinko,S.P.; Macias,E.A.; West,C.A.; Poel,J.R.
EPI1541157	HA	United Kingdom	2018-Apr-04	EPI_ISL_376790	A/England/7208/2018			Gruner,W.E.; Fries,A.C.; Garrett,C.M.; Powell,M.L.; Minnich,D.E.; Couch,M.R.; Hanson,J.F.; DeMarcus,L.S.; Crum,M.E.; Bogue,A.L.; Federinko,S.P.; Macias,E.A.; West,C.A.; Poel,J.R.

Segment ID	Segment	Country	Collection date	Isolate-ID	Isolate name	Originating Lab	Submitting Lab	Authors
EPI1540506	HA	United Kingdom	2018-Feb-01	EPI_ISL_376709	A/England/7127/2018			Gruner,W.E.; Fries,A.C.; Garrett,C.M.; Powell,M.L.; Minnich,D.E.; Couch,M.R.; Hanson,J.F.; DeMarcus,L.S.; Crum,M.E.; Bogue,A.L.; Federinko,S.P.; Macias,E.A.; West,C.A.; Poel,J.R.
EPI1540505	HA	United Kingdom	2018-Jan-16	EPI_ISL_376708	A/England/7126/2018			Gruner,W.E.; Fries,A.C.; Garrett,C.M.; Powell,M.L.; Minnich,D.E.; Couch,M.R.; Hanson,J.F.; DeMarcus,L.S.; Crum,M.E.; Bogue,A.L.; Federinko,S.P.; Macias,E.A.; West,C.A.; Poel,J.R.
EPI1540065	HA	United Kingdom	2018-Jan-08	EPI_ISL_376628	A/England/7014/2018			Gruner,W.E.; Fries,A.C.; Garrett,C.M.; Powell,M.L.; Minnich,D.E.; Couch,M.R.; Hanson,J.F.; DeMarcus,L.S.; Crum,M.E.; Bogue,A.L.; Federinko,S.P.; Macias,E.A.; West,C.A.; Poel,J.R.
EPI1540064	HA	United Kingdom	2018-Jan-07	EPI_ISL_376627	A/England/7013/2018			Gruner,W.E.; Fries,A.C.; Garrett,C.M.; Powell,M.L.; Minnich,D.E.; Couch,M.R.; Hanson,J.F.; DeMarcus,L.S.; Crum,M.E.; Bogue,A.L.; Federinko,S.P.; Macias,E.A.; West,C.A.; Poel,J.R.
EPI1539537	HA	United Kingdom	2018-Feb-07	EPI_ISL_376556	A/England/6942/2018			Gruner,W.E.; Fries,A.C.; Garrett,C.M.; Powell,M.L.; Minnich,D.E.; Couch,M.R.; Hanson,J.F.; DeMarcus,L.S.; Crum,M.E.; Bogue,A.L.; Federinko,S.P.; Macias,E.A.; West,C.A.; Poel,J.R.
EPI1797562	HA	United Kingdom	2019-Jun-03	EPI_ISL_514060	A/England/192360671/2019	UK Health Security Agency - Colindale	UK Health Security Agency - Colindale	

Segment ID	Segment	Country	Collection date	Isolate-ID	Isolate name	Originating Lab	Submitting Lab	Authors
EPI1797554	HA	United Kingdom	2019-Jun-03	EPI_ISL_514059	A/England/192360670/2019	UK Health Security Agency - Colindale	UK Health Security Agency - Colindale	
EPI1797546	HA	United Kingdom	2019-Jun-03	EPI_ISL_514058	A/England/192360669/2019	UK Health Security Agency - Colindale	UK Health Security Agency - Colindale	
EPI1797538	HA	United Kingdom	2019-Jun-04	EPI_ISL_514057	A/England/192360668/2019	UK Health Security Agency - Colindale	UK Health Security Agency - Colindale	
EPI1754376	HA	United Kingdom	2019-Dec-24	EPI_ISL_486999	A/England/200181147/2019	UK Health Security Agency - Colindale	UK Health Security Agency - Colindale	
EPI1754374	HA	United Kingdom	2019-Dec-26	EPI_ISL_486998	A/England/200181145/2019	UK Health Security Agency - Colindale	UK Health Security Agency - Colindale	
EPI1754372	HA	United Kingdom	2019-Dec-24	EPI_ISL_486997	A/England/200181144/2019	UK Health Security Agency - Colindale	UK Health Security Agency - Colindale	
EPI1750372	HA	United Kingdom	2020-Jan-30	EPI_ISL_480550	A/England/200560848/2020	UK Health Security Agency - Colindale	UK Health Security Agency - Colindale	
EPI1750356	HA	United Kingdom	2020-Jan-22	EPI_ISL_480548	A/England/83/2020	UK Health Security Agency - Colindale	UK Health Security Agency - Colindale	
EPI1750340	HA	United Kingdom	2020-Jan-21	EPI_ISL_480546	A/England/200460681/2020	UK Health Security Agency - Colindale	UK Health Security Agency - Colindale	
EPI1750332	HA	United Kingdom	2020-Jan-21	EPI_ISL_480545	A/England/81/2020	UK Health Security Agency - Colindale	UK Health Security Agency - Colindale	
EPI1750324	HA	United Kingdom	2019-Dec-29	EPI_ISL_480544	A/England/200440332/2019	UK Health Security Agency - Colindale	UK Health Security Agency - Colindale	
EPI1750308	HA	United Kingdom	2019-Dec-17	EPI_ISL_480542	A/England/200440330/2019	UK Health Security Agency - Colindale	UK Health Security Agency - Colindale	
EPI1750300	HA	United Kingdom	2019-Dec-17	EPI_ISL_480541	A/England/934/2019	UK Health Security Agency - Colindale	UK Health Security Agency - Colindale	

Segment ID	Segment	Country	Collection date	Isolate-ID	Isolate name	Originating Lab	Submitting Lab	Authors
EPI1750292	HA	United Kingdom	2019-Dec-17	EPI_ISL_480540	A/England/200440328/2019	UK Health Security Agency - Colindale	UK Health Security Agency - Colindale	
EPI1750284	HA	United Kingdom	2019-Dec-17	EPI_ISL_480539	A/England/933/2019	UK Health Security Agency - Colindale	UK Health Security Agency - Colindale	
EPI1750276	HA	United Kingdom	2019-Dec-16	EPI_ISL_480538	A/England/932/2019	UK Health Security Agency - Colindale	UK Health Security Agency - Colindale	
EPI1750268	HA	United Kingdom	2019-Dec-14	EPI_ISL_480537	A/England/200440323/2019	UK Health Security Agency - Colindale	UK Health Security Agency - Colindale	
EPI1750252	HA	United Kingdom	2019-Dec-18	EPI_ISL_480535	A/England/931/2019	UK Health Security Agency - Colindale	UK Health Security Agency - Colindale	
EPI1750236	HA	United Kingdom	2019-Dec-24	EPI_ISL_480533	A/England/200420382/2019	UK Health Security Agency - Colindale	UK Health Security Agency - Colindale	
EPI1750226	HA	United Kingdom	2020-Jan-04	EPI_ISL_480531	A/England/200420372/2020	UK Health Security Agency - Colindale	UK Health Security Agency - Colindale	
EPI1750218	HA	United Kingdom	2020-Jan-03	EPI_ISL_480530	A/England/200420368/2020	UK Health Security Agency - Colindale	UK Health Security Agency - Colindale	
EPI1750186	HA	United Kingdom	2019-Nov-26	EPI_ISL_480526	A/England/200400493/2019	UK Health Security Agency - Colindale	UK Health Security Agency - Colindale	
EPI1750184	HA	United Kingdom	2019-Dec-06	EPI_ISL_480525	A/England/200400491/2019	UK Health Security Agency - Colindale	UK Health Security Agency - Colindale	
EPI1750168	HA	United Kingdom	2020-Jan-15	EPI_ISL_480523	A/England/200400487/2020	UK Health Security Agency - Colindale	UK Health Security Agency - Colindale	
EPI1750160	HA	United Kingdom	2020-Jan-14	EPI_ISL_480522	A/England/79/2020	UK Health Security Agency - Colindale	UK Health Security Agency - Colindale	
EPI1750152	HA	United Kingdom	2020-Jan-13	EPI_ISL_480521	A/England/96/2020	UK Health Security Agency - Colindale	UK Health Security Agency - Colindale	

Segment ID	Segment	Country	Collection date	Isolate-ID	Isolate name	Originating Lab	Submitting Lab	Authors
EPI1750144	HA	United Kingdom	2020-Jan-08	EPI_ISL_480520	A/England/200380303/2020	UK Health Security Agency - Colindale	UK Health Security Agency - Colindale	
EPI1750104	HA	United Kingdom	2020-Jan-09	EPI_ISL_480515	A/England/200360400/2020	UK Health Security Agency - Colindale	UK Health Security Agency - Colindale	
EPI1750096	HA	United Kingdom	2020-Jan-15	EPI_ISL_480514	A/England/200360354/2020	UK Health Security Agency - Colindale	UK Health Security Agency - Colindale	
EPI1750088	HA	United Kingdom	2020-Jan-13	EPI_ISL_480513	A/England/74/2020	UK Health Security Agency - Colindale	UK Health Security Agency - Colindale	
EPI1750072	HA	United Kingdom	2020-Jan-14	EPI_ISL_480511	A/England/200340469/2020	UK Health Security Agency - Colindale	UK Health Security Agency - Colindale	
EPI1750022	HA	United Kingdom	2020-Jan-09	EPI_ISL_480504	A/England/72/2020	UK Health Security Agency - Colindale	UK Health Security Agency - Colindale	
EPI1750014	HA	United Kingdom	2020-Jan-09	EPI_ISL_480503	A/England/200300134/2020	UK Health Security Agency - Colindale	UK Health Security Agency - Colindale	
EPI1750006	HA	United Kingdom	2020-Jan-08	EPI_ISL_480502	A/England/200280382/2020	UK Health Security Agency - Colindale	UK Health Security Agency - Colindale	
EPI1749998	HA	United Kingdom	2020-Jan-09	EPI_ISL_480501	A/England/200280379/2020	UK Health Security Agency - Colindale	UK Health Security Agency - Colindale	
EPI1749990	HA	United Kingdom	2020-Jan-09	EPI_ISL_480500	A/England/71/2020	UK Health Security Agency - Colindale	UK Health Security Agency - Colindale	
EPI1749982	HA	United Kingdom	2019-Dec-31	EPI_ISL_480499	A/England/928/2019	UK Health Security Agency - Colindale	UK Health Security Agency - Colindale	
EPI1749974	HA	United Kingdom	2020-Jan-07	EPI_ISL_480498	A/England/200260602/2020	UK Health Security Agency - Colindale	UK Health Security Agency - Colindale	
EPI1749966	HA	United Kingdom	2020-Jan-06	EPI_ISL_480497	A/England/200260435/2020	UK Health Security Agency - Colindale	UK Health Security Agency - Colindale	

Segment ID	Segment	Country	Collection date	Isolate-ID	Isolate name	Originating Lab	Submitting Lab	Authors
EPI1749958	HA	United Kingdom	2020-Jan-06	EPI_ISL_480496	A/England/67/2020	UK Health Security Agency - Colindale	UK Health Security Agency - Colindale	
EPI1749950	HA	United Kingdom	2020-Jan-06	EPI_ISL_480495	A/England/66/2020	UK Health Security Agency - Colindale	UK Health Security Agency - Colindale	
EPI1749942	HA	United Kingdom	2020-Jan-06	EPI_ISL_480494	A/England/200240300/2020	UK Health Security Agency - Colindale	UK Health Security Agency - Colindale	
EPI1749934	HA	United Kingdom	2020-Jan-06	EPI_ISL_480493	A/England/65/2020	UK Health Security Agency - Colindale	UK Health Security Agency - Colindale	
EPI1749926	HA	United Kingdom	2020-Jan-06	EPI_ISL_480492	A/England/200220918/2020	UK Health Security Agency - Colindale	UK Health Security Agency - Colindale	
EPI1749918	HA	United Kingdom	2019-Dec-31	EPI_ISL_480491	A/England/912/2019	UK Health Security Agency - Colindale	UK Health Security Agency - Colindale	
EPI1749910	HA	United Kingdom	2020-Jan-03	EPI_ISL_480490	A/England/56/2020	UK Health Security Agency - Colindale	UK Health Security Agency - Colindale	
EPI1749908	HA	United Kingdom	2020-Jan-07	EPI_ISL_480489	A/England/53/2020	UK Health Security Agency - Colindale	UK Health Security Agency - Colindale	
EPI1749892	HA	United Kingdom	2020-Jan-02	EPI_ISL_480487	A/England/200220428/2020	UK Health Security Agency - Colindale	UK Health Security Agency - Colindale	
EPI1749884	HA	United Kingdom	2020-Jan-06	EPI_ISL_480486	A/England/51/2020	UK Health Security Agency - Colindale	UK Health Security Agency - Colindale	
EPI1749868	HA	United Kingdom	2020-Jan-02	EPI_ISL_480484	A/England/200200175/2020	UK Health Security Agency - Colindale	UK Health Security Agency - Colindale	
EPI1749852	HA	United Kingdom	2020-Jan-02	EPI_ISL_480482	A/England/200200167/2020	UK Health Security Agency - Colindale	UK Health Security Agency - Colindale	
EPI1749844	HA	United Kingdom	2020-Jan-02	EPI_ISL_480481	A/England/200200159/2020	UK Health Security Agency - Colindale	UK Health Security Agency - Colindale	

Segment ID	Segment	Country	Collection date	Isolate-ID	Isolate name	Originating Lab	Submitting Lab	Authors
EPI1749836	HA	United Kingdom	2020-Jan-03	EPI_ISL_480480	A/England/200200156/2020	UK Health Security Agency - Colindale	UK Health Security Agency - Colindale	
EPI1749828	HA	United Kingdom	2020-Jan-02	EPI_ISL_480479	A/England/200200152/2020	UK Health Security Agency - Colindale	UK Health Security Agency - Colindale	
EPI1749812	HA	United Kingdom	2019-Dec-30	EPI_ISL_480477	A/England/900/2019	UK Health Security Agency - Colindale	UK Health Security Agency - Colindale	
EPI1749804	HA	United Kingdom	2019-Dec-31	EPI_ISL_480476	A/England/899/2019	UK Health Security Agency - Colindale	UK Health Security Agency - Colindale	
EPI1749796	HA	United Kingdom	2019-Dec-20	EPI_ISL_480475	A/England/898/2019	UK Health Security Agency - Colindale	UK Health Security Agency - Colindale	
EPI1749788	HA	United Kingdom	2019-Dec-31	EPI_ISL_480474	A/England/200181118/2019	UK Health Security Agency - Colindale	UK Health Security Agency - Colindale	
EPI1749780	HA	United Kingdom	2019-Dec-31	EPI_ISL_480473	A/England/200181105/2019	UK Health Security Agency - Colindale	UK Health Security Agency - Colindale	
EPI1749772	HA	United Kingdom	2020-Jan-02	EPI_ISL_480472	A/England/47/2020	UK Health Security Agency - Colindale	UK Health Security Agency - Colindale	
EPI1749764	HA	United Kingdom	2019-Dec-30	EPI_ISL_480471	A/England/200181097/2019	UK Health Security Agency - Colindale	UK Health Security Agency - Colindale	
EPI1749748	HA	United Kingdom	2019-Dec-31	EPI_ISL_480469	A/England/897/2019	UK Health Security Agency - Colindale	UK Health Security Agency - Colindale	
EPI1749740	HA	United Kingdom	2019-Dec-24	EPI_ISL_480468	A/England/200181078/2019	UK Health Security Agency - Colindale	UK Health Security Agency - Colindale	
EPI1749732	HA	United Kingdom	2019-Dec-31	EPI_ISL_480467	A/England/896/2019	UK Health Security Agency - Colindale	UK Health Security Agency - Colindale	
EPI1749724	HA	United Kingdom	2019-Dec-30	EPI_ISL_480466	A/England/895/2019	UK Health Security Agency - Colindale	UK Health Security Agency - Colindale	

Segment ID	Segment	Country	Collection date	Isolate-ID	Isolate name	Originating Lab	Submitting Lab	Authors
EPI1749716	HA	United Kingdom	2019-Dec-30	EPI_ISL_480465	A/England/200181044/2019	UK Health Security Agency - Colindale	UK Health Security Agency - Colindale	
EPI1749708	HA	United Kingdom	2020-Jan-03	EPI_ISL_480464	A/England/200181043/2020	UK Health Security Agency - Colindale	UK Health Security Agency - Colindale	
EPI1749700	HA	United Kingdom	2019-Dec-31	EPI_ISL_480463	A/England/894/2019	UK Health Security Agency - Colindale	UK Health Security Agency - Colindale	
EPI1749692	HA	United Kingdom	2020-Jan-02	EPI_ISL_480462	A/England/200181039/2020	UK Health Security Agency - Colindale	UK Health Security Agency - Colindale	
EPI1749684	HA	United Kingdom	2019-Dec-27	EPI_ISL_480461	A/England/882/2019	UK Health Security Agency - Colindale	UK Health Security Agency - Colindale	
EPI1749676	HA	United Kingdom	2019-Dec-27	EPI_ISL_480460	A/England/881/2019	UK Health Security Agency - Colindale	UK Health Security Agency - Colindale	
EPI1749668	HA	United Kingdom	2019-Dec-12	EPI_ISL_480459	A/England/195100502/2019	UK Health Security Agency - Colindale	UK Health Security Agency - Colindale	
EPI1749660	HA	United Kingdom	2019-Dec-05	EPI_ISL_480458	A/England/586/2019	UK Health Security Agency - Colindale	UK Health Security Agency - Colindale	
EPI1749652	HA	United Kingdom	2019-Nov-18	EPI_ISL_480457	A/England/392/2019	UK Health Security Agency - Colindale	UK Health Security Agency - Colindale	
EPI1749562	HA	United Kingdom	2020-Jan-21	EPI_ISL_479615	A/England/200480331/2020	UK Health Security Agency - Colindale	UK Health Security Agency - Colindale	
EPI1749560	HA	United Kingdom	2019-Dec-18	EPI_ISL_479614	A/England/200440339/2019	UK Health Security Agency - Colindale	UK Health Security Agency - Colindale	
EPI1749558	HA	United Kingdom	2019-Dec-17	EPI_ISL_479613	A/England/200440336/2019	UK Health Security Agency - Colindale	UK Health Security Agency - Colindale	
EPI1749555	HA	United Kingdom	2019-Nov-30	EPI_ISL_479611	A/England/200400489/2019	UK Health Security Agency - Colindale	UK Health Security Agency - Colindale	

Segment ID	Segment	Country	Collection date	Isolate-ID	Isolate name	Originating Lab	Submitting Lab	Authors
EPI1749548	HA	United Kingdom	2020-Jan-06	EPI_ISL_479606	A/England/200220912/2020	UK Health Security Agency - Colindale	UK Health Security Agency - Colindale	
EPI1749545	HA	United Kingdom	2019-Dec-30	EPI_ISL_479604	A/England/200181116/2019	UK Health Security Agency - Colindale	UK Health Security Agency - Colindale	
EPI1749296	HA	United Kingdom	2020-Feb-11	EPI_ISL_477527	A/England/107/2020	UK Health Security Agency - Colindale	Crick Worldwide Influenza Centre	
EPI1749294	HA	United Kingdom	2020-Feb-05	EPI_ISL_477526	A/England/103/2020	UK Health Security Agency - Colindale	Crick Worldwide Influenza Centre	
EPI1747119	HA	United Kingdom	2020-Jan-09	EPI_ISL_471078	A/England/200320321/2020	UK Health Security Agency - Colindale	UK Health Security Agency - Colindale	
EPI1747117	HA	United Kingdom	2020-Jan-09	EPI_ISL_471077	A/England/73/2020	UK Health Security Agency - Colindale	UK Health Security Agency - Colindale	
EPI1747101	HA	United Kingdom	2019-Dec-31	EPI_ISL_471075	A/England/200300676/2019	UK Health Security Agency - Colindale	UK Health Security Agency - Colindale	
EPI1747077	HA	United Kingdom	2020-Jan-01	EPI_ISL_471072	A/England/200280691/2020	UK Health Security Agency - Colindale	UK Health Security Agency - Colindale	
EPI1747045	HA	United Kingdom	2019-Dec-19	EPI_ISL_471063	A/England/200280684/2019	UK Health Security Agency - Colindale	UK Health Security Agency - Colindale	
EPI1747037	HA	United Kingdom	2019-Dec-31	EPI_ISL_471059	A/England/929/2019	UK Health Security Agency - Colindale	UK Health Security Agency - Colindale	
EPI1747021	HA	United Kingdom	2020-Jan-06	EPI_ISL_471051	A/England/200260852/2020	UK Health Security Agency - Colindale	UK Health Security Agency - Colindale	
EPI1747012	HA	United Kingdom	2020-Jan-04	EPI_ISL_471043	A/England/200260848/2020	UK Health Security Agency - Colindale	UK Health Security Agency - Colindale	
EPI1744620	HA	United Kingdom	2020-Feb-21	EPI_ISL_463769	A/England/11977/2020	U.S. Air Force School of Aerospace Medicine	U.S. Air Force School of Aerospace Medicine	Gruner, W.E.; Fries, A.C.; Garrett, C.M.; Powell, M.L.; Hanson, J.F.; DeMarcus, L.S.; Sjoberg, P.A.; Bogue, A.L.;

Segment ID	Segment	Country	Collection date	Isolate-ID	Isolate name	Originating Lab	Submitting Lab	Authors
								Robbins, A.S.; Macias, E.A.; Lambert, A.W.; Dane, D.J.
EPI1742622	HA	United Kingdom	2019-Dec-23	EPI_ISL_462118	A/England/874/2019	UK Health Security Agency - Colindale	Crick Worldwide Influenza Centre	
EPI1742620	HA	United Kingdom	2019-Dec-23	EPI_ISL_462117	A/England/873/2019	UK Health Security Agency - Colindale	Crick Worldwide Influenza Centre	
EPI1742610	HA	United Kingdom	2020-Jan-21	EPI_ISL_462112	A/England/200460681/2020	UK Health Security Agency - Colindale	Crick Worldwide Influenza Centre	
EPI1741905	HA	United Kingdom	2019-Dec-24	EPI_ISL_459855	A/England/862/2019	UK Health Security Agency - Colindale	UK Health Security Agency - Colindale	
EPI1741897	HA	United Kingdom	2019-Dec-13	EPI_ISL_459854	A/England/195220290/2019	UK Health Security Agency - Colindale	UK Health Security Agency - Colindale	
EPI1741889	HA	United Kingdom	2019-Dec-14	EPI_ISL_459853	A/England/861/2019	UK Health Security Agency - Colindale	UK Health Security Agency - Colindale	
EPI1741881	HA	United Kingdom	2019-Dec-17	EPI_ISL_459852	A/England/879/2019	UK Health Security Agency - Colindale	UK Health Security Agency - Colindale	
EPI1741873	HA	United Kingdom	2019-Dec-14	EPI_ISL_459851	A/England/195220285/2019	UK Health Security Agency - Colindale	UK Health Security Agency - Colindale	
EPI1741857	HA	United Kingdom	2019-Dec-20	EPI_ISL_459849	A/England/195220281/2019	UK Health Security Agency - Colindale	UK Health Security Agency - Colindale	
EPI1741849	HA	United Kingdom	2019-Dec-16	EPI_ISL_459848	A/England/924/2019	UK Health Security Agency - Colindale	UK Health Security Agency - Colindale	
EPI1741841	HA	United Kingdom	2019-Dec-16	EPI_ISL_459847	A/England/923/2019	UK Health Security Agency - Colindale	UK Health Security Agency - Colindale	
EPI1741833	HA	United Kingdom	2019-Dec-16	EPI_ISL_459846	A/England/922/2019	UK Health Security Agency - Colindale	UK Health Security Agency - Colindale	
EPI1741825	HA	United Kingdom	2019-Dec-20	EPI_ISL_459845	A/England/854/2019	UK Health Security Agency - Colindale	UK Health Security Agency - Colindale	

Segment ID	Segment	Country	Collection date	Isolate-ID	Isolate name	Originating Lab	Submitting Lab	Authors
EPI1741817	HA	United Kingdom	2019-Dec-19	EPI_ISL_459844	A/England/853/2019	UK Health Security Agency - Colindale	UK Health Security Agency - Colindale	
EPI1741809	HA	United Kingdom	2019-Dec-23	EPI_ISL_459843	A/England/195200780/2019	UK Health Security Agency - Colindale	UK Health Security Agency - Colindale	
EPI1741801	HA	United Kingdom	2019-Dec-20	EPI_ISL_459842	A/England/195200779/2019	UK Health Security Agency - Colindale	UK Health Security Agency - Colindale	
EPI1741793	HA	United Kingdom	2019-Dec-19	EPI_ISL_459841	A/England/852/2019	UK Health Security Agency - Colindale	UK Health Security Agency - Colindale	
EPI1741785	HA	United Kingdom	2019-Dec-20	EPI_ISL_459840	A/England/851/2019	UK Health Security Agency - Colindale	UK Health Security Agency - Colindale	
EPI1741777	HA	United Kingdom	2019-Dec-20	EPI_ISL_459839	A/England/195200770/2019	UK Health Security Agency - Colindale	UK Health Security Agency - Colindale	
EPI1741769	HA	United Kingdom	2019-Dec-19	EPI_ISL_459838	A/England/195200703/2019	UK Health Security Agency - Colindale	UK Health Security Agency - Colindale	
EPI1741761	HA	United Kingdom	2019-Dec-19	EPI_ISL_459837	A/England/850/2019	UK Health Security Agency - Colindale	UK Health Security Agency - Colindale	
EPI1741753	HA	United Kingdom	2019-Dec-18	EPI_ISL_459836	A/England/849/2019	UK Health Security Agency - Colindale	UK Health Security Agency - Colindale	
EPI1741745	HA	United Kingdom	2019-Dec-18	EPI_ISL_459835	A/England/838/2019	UK Health Security Agency - Colindale	UK Health Security Agency - Colindale	
EPI1741737	HA	United Kingdom	2019-Dec-18	EPI_ISL_459834	A/England/195200698/2019	UK Health Security Agency - Colindale	UK Health Security Agency - Colindale	
EPI1741729	HA	United Kingdom	2019-Dec-17	EPI_ISL_459833	A/England/195200697/2019	UK Health Security Agency - Colindale	UK Health Security Agency - Colindale	
EPI1741721	HA	United Kingdom	2019-Dec-18	EPI_ISL_459832	A/England/837/2019	UK Health Security Agency - Colindale	UK Health Security Agency - Colindale	

Segment ID	Segment	Country	Collection date	Isolate-ID	Isolate name	Originating Lab	Submitting Lab	Authors
EPI1741713	HA	United Kingdom	2019-Dec-18	EPI_ISL_459831	A/England/195200693/2019	UK Health Security Agency - Colindale	UK Health Security Agency - Colindale	
EPI1741705	HA	United Kingdom	2019-Dec-17	EPI_ISL_459830	A/England/195200691/2019	UK Health Security Agency - Colindale	UK Health Security Agency - Colindale	
EPI1741697	HA	United Kingdom	2019-Dec-19	EPI_ISL_459829	A/England/835/2019	UK Health Security Agency - Colindale	UK Health Security Agency - Colindale	
EPI1741689	HA	United Kingdom	2019-Dec-20	EPI_ISL_459828	A/England/848/2019	UK Health Security Agency - Colindale	UK Health Security Agency - Colindale	
EPI1741681	HA	United Kingdom	2019-Dec-19	EPI_ISL_459827	A/England/195200231/2019	UK Health Security Agency - Colindale	UK Health Security Agency - Colindale	
EPI1741673	HA	United Kingdom	2019-Dec-20	EPI_ISL_459826	A/England/834/2019	UK Health Security Agency - Colindale	UK Health Security Agency - Colindale	
EPI1741665	HA	United Kingdom	2019-Dec-18	EPI_ISL_459825	A/England/833/2019	UK Health Security Agency - Colindale	UK Health Security Agency - Colindale	
EPI1741657	HA	United Kingdom	2019-Dec-19	EPI_ISL_459824	A/England/832/2019	UK Health Security Agency - Colindale	UK Health Security Agency - Colindale	
EPI1741649	HA	United Kingdom	2019-Dec-18	EPI_ISL_459823	A/England/195200141/2019	UK Health Security Agency - Colindale	UK Health Security Agency - Colindale	
EPI1741641	HA	United Kingdom	2019-Dec-20	EPI_ISL_459822	A/England/831/2019	UK Health Security Agency - Colindale	UK Health Security Agency - Colindale	
EPI1741633	HA	United Kingdom	2019-Dec-23	EPI_ISL_459821	A/England/830/2019	UK Health Security Agency - Colindale	UK Health Security Agency - Colindale	
EPI1741625	HA	United Kingdom	2019-Dec-20	EPI_ISL_459820	A/England/829/2019	UK Health Security Agency - Colindale	UK Health Security Agency - Colindale	
EPI1741617	HA	United Kingdom	2019-Dec-20	EPI_ISL_459819	A/England/195200127/2019	UK Health Security Agency - Colindale	UK Health Security Agency - Colindale	

Segment ID	Segment	Country	Collection date	Isolate-ID	Isolate name	Originating Lab	Submitting Lab	Authors
EPI1741615	HA	United Kingdom	2019-Dec-18	EPI_ISL_459818	A/England/828/2019	UK Health Security Agency - Colindale	UK Health Security Agency - Colindale	
EPI1741607	HA	United Kingdom	2019-Dec-17	EPI_ISL_459817	A/England/195180601/2019	UK Health Security Agency - Colindale	UK Health Security Agency - Colindale	
EPI1741599	HA	United Kingdom	2019-Dec-18	EPI_ISL_459816	A/England/195180582/2019	UK Health Security Agency - Colindale	UK Health Security Agency - Colindale	
EPI1741591	HA	United Kingdom	2019-Dec-19	EPI_ISL_459815	A/England/195180527/2019	UK Health Security Agency - Colindale	UK Health Security Agency - Colindale	
EPI1741583	HA	United Kingdom	2019-Dec-18	EPI_ISL_459814	A/England/195180526/2019	UK Health Security Agency - Colindale	UK Health Security Agency - Colindale	
EPI1741575	HA	United Kingdom	2019-Dec-17	EPI_ISL_459813	A/England/817/2019	UK Health Security Agency - Colindale	UK Health Security Agency - Colindale	
EPI1741567	HA	United Kingdom	2019-Dec-18	EPI_ISL_459812	A/England/195180524/2019	UK Health Security Agency - Colindale	UK Health Security Agency - Colindale	
EPI1741559	HA	United Kingdom	2019-Dec-17	EPI_ISL_459811	A/England/195180519/2019	UK Health Security Agency - Colindale	UK Health Security Agency - Colindale	
EPI1741551	HA	United Kingdom	2019-Dec-18	EPI_ISL_459810	A/England/816/2019	UK Health Security Agency - Colindale	UK Health Security Agency - Colindale	
EPI1741543	HA	United Kingdom	2019-Dec-18	EPI_ISL_459809	A/England/195180363/2019	UK Health Security Agency - Colindale	UK Health Security Agency - Colindale	
EPI1741535	HA	United Kingdom	2019-Dec-18	EPI_ISL_459808	A/England/195180360/2019	UK Health Security Agency - Colindale	UK Health Security Agency - Colindale	
EPI1741527	HA	United Kingdom	2019-Dec-17	EPI_ISL_459807	A/England/195180353/2019	UK Health Security Agency - Colindale	UK Health Security Agency - Colindale	
EPI1741525	HA	United Kingdom	2019-Dec-18	EPI_ISL_459806	A/England/813/2019	UK Health Security Agency - Colindale	UK Health Security Agency - Colindale	

Segment ID	Segment	Country	Collection date	Isolate-ID	Isolate name	Originating Lab	Submitting Lab	Authors
EPI1741517	HA	United Kingdom	2019-Dec-16	EPI_ISL_459805	A/England/812/2019	UK Health Security Agency - Colindale	UK Health Security Agency - Colindale	
EPI1741509	HA	United Kingdom	2019-Dec-17	EPI_ISL_459804	A/England/811/2019	UK Health Security Agency - Colindale	UK Health Security Agency - Colindale	
EPI1741501	HA	United Kingdom	2019-Dec-18	EPI_ISL_459803	A/England/810/2019	UK Health Security Agency - Colindale	UK Health Security Agency - Colindale	
EPI1741493	HA	United Kingdom	2019-Dec-16	EPI_ISL_459802	A/England/800/2019	UK Health Security Agency - Colindale	UK Health Security Agency - Colindale	
EPI1741485	HA	United Kingdom	2019-Dec-16	EPI_ISL_459801	A/England/799/2019	UK Health Security Agency - Colindale	UK Health Security Agency - Colindale	
EPI1741477	HA	United Kingdom	2019-Dec-16	EPI_ISL_459800	A/England/798/2019	UK Health Security Agency - Colindale	UK Health Security Agency - Colindale	
EPI1741469	HA	United Kingdom	2019-Dec-17	EPI_ISL_459799	A/England/195160448/2019	UK Health Security Agency - Colindale	UK Health Security Agency - Colindale	
EPI1741453	HA	United Kingdom	2019-Dec-16	EPI_ISL_459797	A/England/788/2019	UK Health Security Agency - Colindale	UK Health Security Agency - Colindale	
EPI1741445	HA	United Kingdom	2019-Dec-17	EPI_ISL_459796	A/England/195160439/2019	UK Health Security Agency - Colindale	UK Health Security Agency - Colindale	
EPI1741429	HA	United Kingdom	2019-Dec-17	EPI_ISL_459794	A/England/787/2019	UK Health Security Agency - Colindale	UK Health Security Agency - Colindale	
EPI1741421	HA	United Kingdom	2019-Dec-16	EPI_ISL_459793	A/England/786/2019	UK Health Security Agency - Colindale	UK Health Security Agency - Colindale	
EPI1741413	HA	United Kingdom	2019-Dec-17	EPI_ISL_459792	A/England/195160231/2019	UK Health Security Agency - Colindale	UK Health Security Agency - Colindale	
EPI1741405	HA	United Kingdom	2019-Dec-17	EPI_ISL_459791	A/England/785/2019	UK Health Security Agency - Colindale	UK Health Security Agency - Colindale	

Segment ID	Segment	Country	Collection date	Isolate-ID	Isolate name	Originating Lab	Submitting Lab	Authors
EPI1741397	HA	United Kingdom	2019-Dec-17	EPI_ISL_459790	A/England/784/2019	UK Health Security Agency - Colindale	UK Health Security Agency - Colindale	
EPI1741389	HA	United Kingdom	2019-Dec-17	EPI_ISL_459789	A/England/195160223/2019	UK Health Security Agency - Colindale	UK Health Security Agency - Colindale	
EPI1741381	HA	United Kingdom	2019-Dec-16	EPI_ISL_459788	A/England/195160222/2019	UK Health Security Agency - Colindale	UK Health Security Agency - Colindale	
EPI1741373	HA	United Kingdom	2019-Dec-13	EPI_ISL_459787	A/England/782/2019	UK Health Security Agency - Colindale	UK Health Security Agency - Colindale	
EPI1741365	HA	United Kingdom	2019-Dec-16	EPI_ISL_459786	A/England/781/2019	UK Health Security Agency - Colindale	UK Health Security Agency - Colindale	
EPI1741357	HA	United Kingdom	2019-Dec-16	EPI_ISL_459785	A/England/780/2019	UK Health Security Agency - Colindale	UK Health Security Agency - Colindale	
EPI1741349	HA	United Kingdom	2019-Dec-11	EPI_ISL_459784	A/England/195160210/2019	UK Health Security Agency - Colindale	UK Health Security Agency - Colindale	
EPI1741341	HA	United Kingdom	2019-Dec-18	EPI_ISL_459783	A/England/779/2019	UK Health Security Agency - Colindale	UK Health Security Agency - Colindale	
EPI1741333	HA	United Kingdom	2019-Dec-12	EPI_ISL_459782	A/England/778/2019	UK Health Security Agency - Colindale	UK Health Security Agency - Colindale	
EPI1741325	HA	United Kingdom	2019-Dec-17	EPI_ISL_459781	A/England/777/2019	UK Health Security Agency - Colindale	UK Health Security Agency - Colindale	
EPI1741309	HA	United Kingdom	2019-Dec-13	EPI_ISL_459779	A/England/776/2019	UK Health Security Agency - Colindale	UK Health Security Agency - Colindale	
EPI1741301	HA	United Kingdom	2019-Dec-16	EPI_ISL_459778	A/England/775/2019	UK Health Security Agency - Colindale	UK Health Security Agency - Colindale	
EPI1741293	HA	United Kingdom	2019-Dec-16	EPI_ISL_459777	A/England/774/2019	UK Health Security Agency - Colindale	UK Health Security Agency - Colindale	

Segment ID	Segment	Country	Collection date	Isolate-ID	Isolate name	Originating Lab	Submitting Lab	Authors
EPI1741285	HA	United Kingdom	2019-Dec-17	EPI_ISL_459776	A/England/772/2019	UK Health Security Agency - Colindale	UK Health Security Agency - Colindale	
EPI1741277	HA	United Kingdom	2019-Dec-10	EPI_ISL_459775	A/England/771/2019	UK Health Security Agency - Colindale	UK Health Security Agency - Colindale	
EPI1741269	HA	United Kingdom	2019-Dec-16	EPI_ISL_459774	A/England/770/2019	UK Health Security Agency - Colindale	UK Health Security Agency - Colindale	
EPI1741261	HA	United Kingdom	2019-Dec-14	EPI_ISL_459773	A/England/195140735/2019	UK Health Security Agency - Colindale	UK Health Security Agency - Colindale	
EPI1741253	HA	United Kingdom	2019-Dec-16	EPI_ISL_459772	A/England/769/2019	UK Health Security Agency - Colindale	UK Health Security Agency - Colindale	
EPI1741245	HA	United Kingdom	2019-Dec-16	EPI_ISL_459771	A/England/767/2019	UK Health Security Agency - Colindale	UK Health Security Agency - Colindale	
EPI1741237	HA	United Kingdom	2019-Dec-16	EPI_ISL_459770	A/England/766/2019	UK Health Security Agency - Colindale	UK Health Security Agency - Colindale	
EPI1741229	HA	United Kingdom	2019-Dec-16	EPI_ISL_459769	A/England/765/2019	UK Health Security Agency - Colindale	UK Health Security Agency - Colindale	
EPI1741221	HA	United Kingdom	2019-Dec-13	EPI_ISL_459768	A/England/764/2019	UK Health Security Agency - Colindale	UK Health Security Agency - Colindale	
EPI1741213	HA	United Kingdom	2019-Dec-16	EPI_ISL_459767	A/England/763/2019	UK Health Security Agency - Colindale	UK Health Security Agency - Colindale	
EPI1741205	HA	United Kingdom	2019-Dec-16	EPI_ISL_459766	A/England/762/2019	UK Health Security Agency - Colindale	UK Health Security Agency - Colindale	
EPI1741189	HA	United Kingdom	2019-Dec-13	EPI_ISL_459764	A/England/761/2019	UK Health Security Agency - Colindale	UK Health Security Agency - Colindale	
EPI1741181	HA	United Kingdom	2019-Dec-13	EPI_ISL_459763	A/England/748/2019	UK Health Security Agency - Colindale	UK Health Security Agency - Colindale	

Segment ID	Segment	Country	Collection date	Isolate-ID	Isolate name	Originating Lab	Submitting Lab	Authors
EPI1741173	HA	United Kingdom	2019-Dec-11	EPI_ISL_459762	A/England/195140156/2019	UK Health Security Agency - Colindale	UK Health Security Agency - Colindale	
EPI1741165	HA	United Kingdom	2019-Dec-15	EPI_ISL_459761	A/England/732/2019	UK Health Security Agency - Colindale	UK Health Security Agency - Colindale	
EPI1741157	HA	United Kingdom	2019-Dec-11	EPI_ISL_459760	A/England/731/2019	UK Health Security Agency - Colindale	UK Health Security Agency - Colindale	
EPI1741149	HA	United Kingdom	2019-Dec-13	EPI_ISL_459759	A/England/724/2019	UK Health Security Agency - Colindale	UK Health Security Agency - Colindale	
EPI1741141	HA	United Kingdom	2019-Dec-13	EPI_ISL_459758	A/England/723/2019	UK Health Security Agency - Colindale	UK Health Security Agency - Colindale	
EPI1741133	HA	United Kingdom	2019-Dec-13	EPI_ISL_459757	A/England/722/2019	UK Health Security Agency - Colindale	UK Health Security Agency - Colindale	
EPI1741125	HA	United Kingdom	2019-Dec-13	EPI_ISL_459756	A/England/721/2019	UK Health Security Agency - Colindale	UK Health Security Agency - Colindale	
EPI1741117	HA	United Kingdom	2019-Dec-13	EPI_ISL_459755	A/England/719/2019	UK Health Security Agency - Colindale	UK Health Security Agency - Colindale	
EPI1741109	HA	United Kingdom	2019-Dec-12	EPI_ISL_459754	A/England/718/2019	UK Health Security Agency - Colindale	UK Health Security Agency - Colindale	
EPI1741101	HA	United Kingdom	2019-Dec-13	EPI_ISL_459753	A/England/717/2019	UK Health Security Agency - Colindale	UK Health Security Agency - Colindale	
EPI1741093	HA	United Kingdom	2019-Dec-12	EPI_ISL_459752	A/England/195100388/2019	UK Health Security Agency - Colindale	UK Health Security Agency - Colindale	
EPI1741091	HA	United Kingdom	2019-Dec-12	EPI_ISL_459751	A/England/716/2019	UK Health Security Agency - Colindale	UK Health Security Agency - Colindale	
EPI1741083	HA	United Kingdom	2019-Dec-11	EPI_ISL_459750	A/England/715/2019	UK Health Security Agency - Colindale	UK Health Security Agency - Colindale	

Segment ID	Segment	Country	Collection date	Isolate-ID	Isolate name	Originating Lab	Submitting Lab	Authors
EPI1741075	HA	United Kingdom	2019-Dec-11	EPI_ISL_459749	A/England/706/2019	UK Health Security Agency - Colindale	UK Health Security Agency - Colindale	
EPI1741067	HA	United Kingdom	2019-Nov-29	EPI_ISL_459748	A/England/705/2019	UK Health Security Agency - Colindale	UK Health Security Agency - Colindale	
EPI1741059	HA	United Kingdom	2019-Dec-09	EPI_ISL_459747	A/England/660/2019	UK Health Security Agency - Colindale	UK Health Security Agency - Colindale	
EPI1741051	HA	United Kingdom	2019-Dec-06	EPI_ISL_459746	A/England/659/2019	UK Health Security Agency - Colindale	UK Health Security Agency - Colindale	
EPI1741043	HA	United Kingdom	2019-Dec-06	EPI_ISL_459745	A/England/658/2019	UK Health Security Agency - Colindale	UK Health Security Agency - Colindale	
EPI1741035	HA	United Kingdom	2019-Dec-09	EPI_ISL_459744	A/England/656/2019	UK Health Security Agency - Colindale	UK Health Security Agency - Colindale	
EPI1741027	HA	United Kingdom	2019-Dec-09	EPI_ISL_459743	A/England/655/2019	UK Health Security Agency - Colindale	UK Health Security Agency - Colindale	
EPI1741019	HA	United Kingdom	2019-Dec-10	EPI_ISL_459742	A/England/654/2019	UK Health Security Agency - Colindale	UK Health Security Agency - Colindale	
EPI1741011	HA	United Kingdom	2019-Dec-10	EPI_ISL_459741	A/England/653/2019	UK Health Security Agency - Colindale	UK Health Security Agency - Colindale	
EPI1741003	HA	United Kingdom	2019-Dec-09	EPI_ISL_459740	A/England/195040642/2019	UK Health Security Agency - Colindale	UK Health Security Agency - Colindale	
EPI1740995	HA	United Kingdom	2019-Dec-10	EPI_ISL_459739	A/England/195040640/2019	UK Health Security Agency - Colindale	UK Health Security Agency - Colindale	
EPI1740987	HA	United Kingdom	2019-Dec-09	EPI_ISL_459738	A/England/652/2019	UK Health Security Agency - Colindale	UK Health Security Agency - Colindale	
EPI1740979	HA	United Kingdom	2019-Dec-05	EPI_ISL_459737	A/England/651/2019	UK Health Security Agency - Colindale	UK Health Security Agency - Colindale	

Segment ID	Segment	Country	Collection date	Isolate-ID	Isolate name	Originating Lab	Submitting Lab	Authors
EPI1740971	HA	United Kingdom	2019-Dec-09	EPI_ISL_459736	A/England/650/2019	UK Health Security Agency - Colindale	UK Health Security Agency - Colindale	
EPI1740963	HA	United Kingdom	2019-Dec-04	EPI_ISL_459735	A/England/649/2019	UK Health Security Agency - Colindale	UK Health Security Agency - Colindale	
EPI1740955	HA	United Kingdom	2019-Dec-06	EPI_ISL_459734	A/England/638/2019	UK Health Security Agency - Colindale	UK Health Security Agency - Colindale	
EPI1740947	HA	United Kingdom	2019-Dec-06	EPI_ISL_459733	A/England/637/2019	UK Health Security Agency - Colindale	UK Health Security Agency - Colindale	
EPI1740931	HA	United Kingdom	2019-Dec-05	EPI_ISL_459731	A/England/195000610/2019	UK Health Security Agency - Colindale	UK Health Security Agency - Colindale	
EPI1740915	HA	United Kingdom	2019-Dec-06	EPI_ISL_459729	A/England/621/2019	UK Health Security Agency - Colindale	UK Health Security Agency - Colindale	
EPI1740907	HA	United Kingdom	2019-Nov-29	EPI_ISL_459728	A/England/553/2019	UK Health Security Agency - Colindale	UK Health Security Agency - Colindale	
EPI1740897	HA	United Kingdom	2019-Dec-19	EPI_ISL_459302	A/England/195220279/2019	UK Health Security Agency - Colindale	UK Health Security Agency - Colindale	
EPI1740894	HA	United Kingdom	2019-Dec-17	EPI_ISL_459300	A/England/814/2019	UK Health Security Agency - Colindale	UK Health Security Agency - Colindale	
EPI1740892	HA	United Kingdom	2019-Dec-18	EPI_ISL_459299	A/England/195180342/2019	UK Health Security Agency - Colindale	UK Health Security Agency - Colindale	
EPI1740890	HA	United Kingdom	2019-Dec-11	EPI_ISL_459298	A/England/195100228/2019	UK Health Security Agency - Colindale	UK Health Security Agency - Colindale	
EPI1740886	HA	United Kingdom	2019-Nov-25	EPI_ISL_458289	A/Paris/2554/2019	Crick Worldwide Influenza Centre	WHO Collaborating Centre for Reference and Research on Influenza	Deng,Y-M; Iannello,P; Lau,H; Todd,A; Spirason,N; Moselen,J; Aziz,A; Komadina,N.

Segment ID	Segment	Country	Collection date	Isolate-ID	Isolate name	Originating Lab	Submitting Lab	Authors
EPI1733259	HA	United Kingdom	2019-Dec-08	EPI_ISL_428668	A/England/195180883/2019	UK Health Security Agency - Colindale	UK Health Security Agency - Colindale	
EPI1733251	HA	United Kingdom	2019-Dec-04	EPI_ISL_428667	A/England/827/2019	UK Health Security Agency - Colindale	UK Health Security Agency - Colindale	
EPI1733227	HA	United Kingdom	2019-Dec-17	EPI_ISL_428664	A/England/826/2019	UK Health Security Agency - Colindale	UK Health Security Agency - Colindale	
EPI1733219	HA	United Kingdom	2019-Dec-13	EPI_ISL_428663	A/England/825/2019	UK Health Security Agency - Colindale	UK Health Security Agency - Colindale	
EPI1733211	HA	United Kingdom	2019-Dec-17	EPI_ISL_428662	A/England/823/2019	UK Health Security Agency - Colindale	UK Health Security Agency - Colindale	
EPI1733203	HA	United Kingdom	2019-Dec-14	EPI_ISL_428661	A/England/822/2019	UK Health Security Agency - Colindale	UK Health Security Agency - Colindale	
EPI1733195	HA	United Kingdom	2019-Dec-17	EPI_ISL_428660	A/England/821/2019	UK Health Security Agency - Colindale	UK Health Security Agency - Colindale	
EPI1733187	HA	United Kingdom	2019-Dec-17	EPI_ISL_428659	A/England/195180869/2019	UK Health Security Agency - Colindale	UK Health Security Agency - Colindale	
EPI1733179	HA	United Kingdom	2019-Dec-13	EPI_ISL_428658	A/England/819/2019	UK Health Security Agency - Colindale	UK Health Security Agency - Colindale	
EPI1733171	HA	United Kingdom	2019-Dec-16	EPI_ISL_428657	A/England/195160830/2019	UK Health Security Agency - Colindale	UK Health Security Agency - Colindale	
EPI1733163	HA	United Kingdom	2019-Dec-17	EPI_ISL_428656	A/England/195160669/2019	UK Health Security Agency - Colindale	UK Health Security Agency - Colindale	
EPI1733155	HA	United Kingdom	2019-Dec-16	EPI_ISL_428655	A/England/195160666/2019	UK Health Security Agency - Colindale	UK Health Security Agency - Colindale	
EPI1733147	HA	United Kingdom	2019-Dec-17	EPI_ISL_428654	A/England/809/2019	UK Health Security Agency - Colindale	UK Health Security Agency - Colindale	

Segment ID	Segment	Country	Collection date	Isolate-ID	Isolate name	Originating Lab	Submitting Lab	Authors
EPI1733139	HA	United Kingdom	2019-Dec-17	EPI_ISL_428653	A/England/195160663/2019	UK Health Security Agency - Colindale	UK Health Security Agency - Colindale	
EPI1733131	HA	United Kingdom	2019-Dec-16	EPI_ISL_428652	A/England/808/2019	UK Health Security Agency - Colindale	UK Health Security Agency - Colindale	
EPI1733123	HA	United Kingdom	2019-Dec-12	EPI_ISL_428651	A/England/195160659/2019	UK Health Security Agency - Colindale	UK Health Security Agency - Colindale	
EPI1733115	HA	United Kingdom	2019-Dec-10	EPI_ISL_428650	A/England/195160658/2019	UK Health Security Agency - Colindale	UK Health Security Agency - Colindale	
EPI1733107	HA	United Kingdom	2019-Dec-11	EPI_ISL_428649	A/England/807/2019	UK Health Security Agency - Colindale	UK Health Security Agency - Colindale	
EPI1733099	HA	United Kingdom	2019-Dec-12	EPI_ISL_428648	A/England/806/2019	UK Health Security Agency - Colindale	UK Health Security Agency - Colindale	
EPI1733091	HA	United Kingdom	2019-Dec-15	EPI_ISL_428647	A/England/805/2019	UK Health Security Agency - Colindale	UK Health Security Agency - Colindale	
EPI1733083	HA	United Kingdom	2019-Dec-14	EPI_ISL_428646	A/England/195160653/2019	UK Health Security Agency - Colindale	UK Health Security Agency - Colindale	
EPI1733075	HA	United Kingdom	2019-Dec-17	EPI_ISL_428645	A/England/195160650/2019	UK Health Security Agency - Colindale	UK Health Security Agency - Colindale	
EPI1733067	HA	United Kingdom	2019-Dec-13	EPI_ISL_428644	A/England/802/2019	UK Health Security Agency - Colindale	UK Health Security Agency - Colindale	
EPI1733059	HA	United Kingdom	2019-Dec-12	EPI_ISL_428643	A/England/801/2019	UK Health Security Agency - Colindale	UK Health Security Agency - Colindale	
EPI1733051	HA	United Kingdom	2019-Dec-11	EPI_ISL_428642	A/England/759/2019	UK Health Security Agency - Colindale	UK Health Security Agency - Colindale	
EPI1733043	HA	United Kingdom	2019-Dec-10	EPI_ISL_428641	A/England/758/2019	UK Health Security Agency - Colindale	UK Health Security Agency - Colindale	

Segment ID	Segment	Country	Collection date	Isolate-ID	Isolate name	Originating Lab	Submitting Lab	Authors
EPI1733035	HA	United Kingdom	2019-Dec-11	EPI_ISL_428640	A/England/756/2019	UK Health Security Agency - Colindale	UK Health Security Agency - Colindale	
EPI1733027	HA	United Kingdom	2019-Dec-09	EPI_ISL_428639	A/England/195140660/2019	UK Health Security Agency - Colindale	UK Health Security Agency - Colindale	
EPI1733019	HA	United Kingdom	2019-Dec-11	EPI_ISL_428638	A/England/754/2019	UK Health Security Agency - Colindale	UK Health Security Agency - Colindale	
EPI1733011	HA	United Kingdom	2019-Dec-10	EPI_ISL_428637	A/England/753/2019	UK Health Security Agency - Colindale	UK Health Security Agency - Colindale	
EPI1733003	HA	United Kingdom	2019-Dec-10	EPI_ISL_428636	A/England/752/2019	UK Health Security Agency - Colindale	UK Health Security Agency - Colindale	
EPI1732995	HA	United Kingdom	2019-Dec-10	EPI_ISL_428635	A/England/751/2019	UK Health Security Agency - Colindale	UK Health Security Agency - Colindale	
EPI1732987	HA	United Kingdom	2019-Dec-10	EPI_ISL_428634	A/England/749/2019	UK Health Security Agency - Colindale	UK Health Security Agency - Colindale	
EPI1732979	HA	United Kingdom	2019-Dec-10	EPI_ISL_428633	A/England/747/2019	UK Health Security Agency - Colindale	UK Health Security Agency - Colindale	
EPI1732971	HA	United Kingdom	2019-Dec-13	EPI_ISL_428632	A/England/195140179/2019	UK Health Security Agency - Colindale	UK Health Security Agency - Colindale	
EPI1732963	HA	United Kingdom	2019-Dec-11	EPI_ISL_428631	A/England/195140178/2019	UK Health Security Agency - Colindale	UK Health Security Agency - Colindale	
EPI1732955	HA	United Kingdom	2019-Dec-14	EPI_ISL_428630	A/England/195140177/2019	UK Health Security Agency - Colindale	UK Health Security Agency - Colindale	
EPI1732947	HA	United Kingdom	2019-Dec-14	EPI_ISL_428629	A/England/195140176/2019	UK Health Security Agency - Colindale	UK Health Security Agency - Colindale	
EPI1732939	HA	United Kingdom	2019-Dec-13	EPI_ISL_428628	A/England/746/2019	UK Health Security Agency - Colindale	UK Health Security Agency - Colindale	

Segment ID	Segment	Country	Collection date	Isolate-ID	Isolate name	Originating Lab	Submitting Lab	Authors
EPI1732931	HA	United Kingdom	2019-Dec-09	EPI_ISL_428627	A/England/744/2019	UK Health Security Agency - Colindale	UK Health Security Agency - Colindale	
EPI1732923	HA	United Kingdom	2019-Dec-09	EPI_ISL_428626	A/England/195140169/2019	UK Health Security Agency - Colindale	UK Health Security Agency - Colindale	
EPI1732915	HA	United Kingdom	2019-Dec-09	EPI_ISL_428625	A/England/743/2019	UK Health Security Agency - Colindale	UK Health Security Agency - Colindale	
EPI1732907	HA	United Kingdom	2019-Dec-10	EPI_ISL_428624	A/England/195140167/2019	UK Health Security Agency - Colindale	UK Health Security Agency - Colindale	
EPI1732899	HA	United Kingdom	2019-Dec-13	EPI_ISL_428623	A/England/742/2019	UK Health Security Agency - Colindale	UK Health Security Agency - Colindale	
EPI1732891	HA	United Kingdom	2019-Dec-13	EPI_ISL_428622	A/England/195140161/2019	UK Health Security Agency - Colindale	UK Health Security Agency - Colindale	
EPI1732875	HA	United Kingdom	2019-Dec-12	EPI_ISL_428620	A/England/741/2019	UK Health Security Agency - Colindale	UK Health Security Agency - Colindale	
EPI1732867	HA	United Kingdom	2019-Dec-12	EPI_ISL_428619	A/England/739/2019	UK Health Security Agency - Colindale	UK Health Security Agency - Colindale	
EPI1732859	HA	United Kingdom	2019-Dec-12	EPI_ISL_428618	A/England/738/2019	UK Health Security Agency - Colindale	UK Health Security Agency - Colindale	
EPI1732851	HA	United Kingdom	2019-Dec-12	EPI_ISL_428617	A/England/737/2019	UK Health Security Agency - Colindale	UK Health Security Agency - Colindale	
EPI1732843	HA	United Kingdom	2019-Dec-13	EPI_ISL_428616	A/England/195140148/2019	UK Health Security Agency - Colindale	UK Health Security Agency - Colindale	
EPI1732835	HA	United Kingdom	2019-Dec-13	EPI_ISL_428615	A/England/735/2019	UK Health Security Agency - Colindale	UK Health Security Agency - Colindale	
EPI1732827	HA	United Kingdom	2019-Dec-13	EPI_ISL_428614	A/England/734/2019	UK Health Security Agency - Colindale	UK Health Security Agency - Colindale	

Segment ID	Segment	Country	Collection date	Isolate-ID	Isolate name	Originating Lab	Submitting Lab	Authors
EPI1732819	HA	United Kingdom	2019-Dec-13	EPI_ISL_428613	A/England/195140140/2019	UK Health Security Agency - Colindale	UK Health Security Agency - Colindale	
EPI1732811	HA	United Kingdom	2019-Dec-13	EPI_ISL_428612	A/England/195140139/2019	UK Health Security Agency - Colindale	UK Health Security Agency - Colindale	
EPI1732795	HA	United Kingdom	2019-Dec-15	EPI_ISL_428610	A/England/195120681/2019	UK Health Security Agency - Colindale	UK Health Security Agency - Colindale	
EPI1732771	HA	United Kingdom	2019-Dec-10	EPI_ISL_428607	A/England/730/2019	UK Health Security Agency - Colindale	UK Health Security Agency - Colindale	
EPI1732763	HA	United Kingdom	2019-Dec-08	EPI_ISL_428606	A/England/195120668/2019	UK Health Security Agency - Colindale	UK Health Security Agency - Colindale	
EPI1732755	HA	United Kingdom	2019-Dec-10	EPI_ISL_428605	A/England/195120667/2019	UK Health Security Agency - Colindale	UK Health Security Agency - Colindale	
EPI1732747	HA	United Kingdom	2019-Dec-08	EPI_ISL_428604	A/England/195120666/2019	UK Health Security Agency - Colindale	UK Health Security Agency - Colindale	
EPI1732739	HA	United Kingdom	2019-Dec-10	EPI_ISL_428603	A/England/728/2019	UK Health Security Agency - Colindale	UK Health Security Agency - Colindale	
EPI1732731	HA	United Kingdom	2019-Dec-10	EPI_ISL_428602	A/England/195120662/2019	UK Health Security Agency - Colindale	UK Health Security Agency - Colindale	
EPI1732723	HA	United Kingdom	2019-Dec-10	EPI_ISL_428601	A/England/727/2019	UK Health Security Agency - Colindale	UK Health Security Agency - Colindale	
EPI1732715	HA	United Kingdom	2019-Dec-09	EPI_ISL_428600	A/England/726/2019	UK Health Security Agency - Colindale	UK Health Security Agency - Colindale	
EPI1732707	HA	United Kingdom	2019-Dec-12	EPI_ISL_428599	A/England/725/2019	UK Health Security Agency - Colindale	UK Health Security Agency - Colindale	
EPI1732699	HA	United Kingdom	2019-Dec-10	EPI_ISL_428598	A/England/195100776/2019	UK Health Security Agency - Colindale	UK Health Security Agency - Colindale	

Segment ID	Segment	Country	Collection date	Isolate-ID	Isolate name	Originating Lab	Submitting Lab	Authors
EPI1732691	HA	United Kingdom	2019-Dec-09	EPI_ISL_428597	A/England/195100772/2019	UK Health Security Agency - Colindale	UK Health Security Agency - Colindale	
EPI1732683	HA	United Kingdom	2019-Dec-12	EPI_ISL_428596	A/England/195100332/2019	UK Health Security Agency - Colindale	UK Health Security Agency - Colindale	
EPI1732675	HA	United Kingdom	2019-Dec-04	EPI_ISL_428595	A/England/714/2019	UK Health Security Agency - Colindale	UK Health Security Agency - Colindale	
EPI1732667	HA	United Kingdom	2019-Dec-13	EPI_ISL_428594	A/England/712/2019	UK Health Security Agency - Colindale	UK Health Security Agency - Colindale	
EPI1732659	HA	United Kingdom	2019-Dec-13	EPI_ISL_428593	A/England/711/2019	UK Health Security Agency - Colindale	UK Health Security Agency - Colindale	
EPI1732651	HA	United Kingdom	2019-Dec-13	EPI_ISL_428592	A/England/195100191/2019	UK Health Security Agency - Colindale	UK Health Security Agency - Colindale	
EPI1732643	HA	United Kingdom	2019-Dec-13	EPI_ISL_428591	A/England/195100190/2019	UK Health Security Agency - Colindale	UK Health Security Agency - Colindale	
EPI1732635	HA	United Kingdom	2019-Dec-11	EPI_ISL_428590	A/England/195100186/2019	UK Health Security Agency - Colindale	UK Health Security Agency - Colindale	
EPI1732627	HA	United Kingdom	2019-Dec-10	EPI_ISL_428589	A/England/710/2019	UK Health Security Agency - Colindale	UK Health Security Agency - Colindale	
EPI1732619	HA	United Kingdom	2019-Dec-13	EPI_ISL_428588	A/England/709/2019	UK Health Security Agency - Colindale	UK Health Security Agency - Colindale	
EPI1732611	HA	United Kingdom	2019-Dec-11	EPI_ISL_428587	A/England/708/2019	UK Health Security Agency - Colindale	UK Health Security Agency - Colindale	
EPI1732603	HA	United Kingdom	2019-Dec-11	EPI_ISL_428586	A/England/195100176/2019	UK Health Security Agency - Colindale	UK Health Security Agency - Colindale	
EPI1732595	HA	United Kingdom	2019-Dec-10	EPI_ISL_428585	A/England/707/2019	UK Health Security Agency - Colindale	UK Health Security Agency - Colindale	

Segment ID	Segment	Country	Collection date	Isolate-ID	Isolate name	Originating Lab	Submitting Lab	Authors
EPI1732587	HA	United Kingdom	2019-Dec-12	EPI_ISL_428584	A/England/195100163/2019	UK Health Security Agency - Colindale	UK Health Security Agency - Colindale	
EPI1732579	HA	United Kingdom	2019-Dec-13	EPI_ISL_428583	A/England/195100160/2019	UK Health Security Agency - Colindale	UK Health Security Agency - Colindale	
EPI1732571	HA	United Kingdom	2019-Dec-07	EPI_ISL_428582	A/England/195080690/2019	UK Health Security Agency - Colindale	UK Health Security Agency - Colindale	
EPI1732563	HA	United Kingdom	2019-Dec-05	EPI_ISL_428581	A/England/195080688/2019	UK Health Security Agency - Colindale	UK Health Security Agency - Colindale	
EPI1732555	HA	United Kingdom	2019-Dec-11	EPI_ISL_428580	A/England/195080686/2019	UK Health Security Agency - Colindale	UK Health Security Agency - Colindale	
EPI1732547	HA	United Kingdom	2019-Dec-07	EPI_ISL_428579	A/England/195080685/2019	UK Health Security Agency - Colindale	UK Health Security Agency - Colindale	
EPI1732539	HA	United Kingdom	2019-Dec-06	EPI_ISL_428578	A/England/704/2019	UK Health Security Agency - Colindale	UK Health Security Agency - Colindale	
EPI1732531	HA	United Kingdom	2019-Dec-06	EPI_ISL_428577	A/England/703/2019	UK Health Security Agency - Colindale	UK Health Security Agency - Colindale	
EPI1732523	HA	United Kingdom	2019-Dec-11	EPI_ISL_428576	A/England/702/2019	UK Health Security Agency - Colindale	UK Health Security Agency - Colindale	
EPI1732515	HA	United Kingdom	2019-Dec-12	EPI_ISL_428575	A/England/682/2019	UK Health Security Agency - Colindale	UK Health Security Agency - Colindale	
EPI1732499	HA	United Kingdom	2019-Dec-11	EPI_ISL_428573	A/England/195080609/2019	UK Health Security Agency - Colindale	UK Health Security Agency - Colindale	
EPI1732491	HA	United Kingdom	2019-Dec-11	EPI_ISL_428572	A/England/681/2019	UK Health Security Agency - Colindale	UK Health Security Agency - Colindale	
EPI1732483	HA	United Kingdom	2019-Dec-11	EPI_ISL_428571	A/England/680/2019	UK Health Security Agency - Colindale	UK Health Security Agency - Colindale	

Segment ID	Segment	Country	Collection date	Isolate-ID	Isolate name	Originating Lab	Submitting Lab	Authors
EPI1732475	HA	United Kingdom	2019-Dec-10	EPI_ISL_428570	A/England/195080606/2019	UK Health Security Agency - Colindale	UK Health Security Agency - Colindale	
EPI1732467	HA	United Kingdom	2019-Dec-11	EPI_ISL_428569	A/England/679/2019	UK Health Security Agency - Colindale	UK Health Security Agency - Colindale	
EPI1732459	HA	United Kingdom	2019-Dec-09	EPI_ISL_428568	A/England/195080599/2019	UK Health Security Agency - Colindale	UK Health Security Agency - Colindale	
EPI1732451	HA	United Kingdom	2019-Dec-11	EPI_ISL_428567	A/England/678/2019	UK Health Security Agency - Colindale	UK Health Security Agency - Colindale	
EPI1732443	HA	United Kingdom	2019-Dec-11	EPI_ISL_428566	A/England/195080592/2019	UK Health Security Agency - Colindale	UK Health Security Agency - Colindale	
EPI1732435	HA	United Kingdom	2019-Dec-11	EPI_ISL_428565	A/England/677/2019	UK Health Security Agency - Colindale	UK Health Security Agency - Colindale	
EPI1732427	HA	United Kingdom	2019-Dec-08	EPI_ISL_428564	A/England/676/2019	UK Health Security Agency - Colindale	UK Health Security Agency - Colindale	
EPI1732411	HA	United Kingdom	2019-Dec-08	EPI_ISL_428562	A/England/675/2019	UK Health Security Agency - Colindale	UK Health Security Agency - Colindale	
EPI1732403	HA	United Kingdom	2019-Dec-07	EPI_ISL_428561	A/England/674/2019	UK Health Security Agency - Colindale	UK Health Security Agency - Colindale	
EPI1732395	HA	United Kingdom	2019-Dec-07	EPI_ISL_428560	A/England/673/2019	UK Health Security Agency - Colindale	UK Health Security Agency - Colindale	
EPI1732387	HA	United Kingdom	2019-Dec-09	EPI_ISL_428559	A/England/195060724/2019	UK Health Security Agency - Colindale	UK Health Security Agency - Colindale	
EPI1732379	HA	United Kingdom	2019-Dec-11	EPI_ISL_428558	A/England/672/2019	UK Health Security Agency - Colindale	UK Health Security Agency - Colindale	
EPI1732371	HA	United Kingdom	2019-Dec-09	EPI_ISL_428557	A/England/195060600/2019	UK Health Security Agency - Colindale	UK Health Security Agency - Colindale	

Segment ID	Segment	Country	Collection date	Isolate-ID	Isolate name	Originating Lab	Submitting Lab	Authors
EPI1732363	HA	United Kingdom	2019-Dec-10	EPI_ISL_428556	A/England/671/2019	UK Health Security Agency - Colindale	UK Health Security Agency - Colindale	
EPI1732355	HA	United Kingdom	2019-Dec-09	EPI_ISL_428555	A/England/670/2019	UK Health Security Agency - Colindale	UK Health Security Agency - Colindale	
EPI1732339	HA	United Kingdom	2019-Dec-11	EPI_ISL_428553	A/England/669/2019	UK Health Security Agency - Colindale	UK Health Security Agency - Colindale	
EPI1732331	HA	United Kingdom	2019-Dec-11	EPI_ISL_428552	A/England/668/2019	UK Health Security Agency - Colindale	UK Health Security Agency - Colindale	
EPI1732323	HA	United Kingdom	2019-Dec-10	EPI_ISL_428551	A/England/667/2019	UK Health Security Agency - Colindale	UK Health Security Agency - Colindale	
EPI1732315	HA	United Kingdom	2019-Dec-08	EPI_ISL_428550	A/England/666/2019	UK Health Security Agency - Colindale	UK Health Security Agency - Colindale	
EPI1732307	HA	United Kingdom	2019-Dec-06	EPI_ISL_428549	A/England/664/2019	UK Health Security Agency - Colindale	UK Health Security Agency - Colindale	
EPI1732299	HA	United Kingdom	2019-Dec-11	EPI_ISL_428548	A/England/195040731/2019	UK Health Security Agency - Colindale	UK Health Security Agency - Colindale	
EPI1732291	HA	United Kingdom	2019-Dec-11	EPI_ISL_428547	A/England/661/2019	UK Health Security Agency - Colindale	UK Health Security Agency - Colindale	
EPI1732283	HA	United Kingdom	2019-Dec-09	EPI_ISL_428546	A/England/195040663/2019	UK Health Security Agency - Colindale	UK Health Security Agency - Colindale	
EPI1732275	HA	United Kingdom	2019-Dec-09	EPI_ISL_428545	A/England/195040635/2019	UK Health Security Agency - Colindale	UK Health Security Agency - Colindale	
EPI1732259	HA	United Kingdom	2019-Dec-03	EPI_ISL_428543	A/England/639/2019	UK Health Security Agency - Colindale	UK Health Security Agency - Colindale	
EPI1732251	HA	United Kingdom	2019-Dec-06	EPI_ISL_428542	A/England/620/2019	UK Health Security Agency - Colindale	UK Health Security Agency - Colindale	

Segment ID	Segment	Country	Collection date	Isolate-ID	Isolate name	Originating Lab	Submitting Lab	Authors
EPI1732243	HA	United Kingdom	2019-Dec-05	EPI_ISL_428541	A/England/619/2019	UK Health Security Agency - Colindale	UK Health Security Agency - Colindale	
EPI1732235	HA	United Kingdom	2019-Dec-04	EPI_ISL_428540	A/England/618/2019	UK Health Security Agency - Colindale	UK Health Security Agency - Colindale	
EPI1732227	HA	United Kingdom	2019-Dec-06	EPI_ISL_428539	A/England/616/2019	UK Health Security Agency - Colindale	UK Health Security Agency - Colindale	
EPI1732219	HA	United Kingdom	2019-Dec-02	EPI_ISL_428538	A/England/588/2019	UK Health Security Agency - Colindale	UK Health Security Agency - Colindale	
EPI1732211	HA	United Kingdom	2019-Dec-04	EPI_ISL_428537	A/England/587/2019	UK Health Security Agency - Colindale	UK Health Security Agency - Colindale	
EPI1732203	HA	United Kingdom	2019-Dec-03	EPI_ISL_428536	A/England/583/2019	UK Health Security Agency - Colindale	UK Health Security Agency - Colindale	
EPI1732195	HA	United Kingdom	2019-Dec-03	EPI_ISL_428535	A/England/194980208/2019	UK Health Security Agency - Colindale	UK Health Security Agency - Colindale	
EPI1732187	HA	United Kingdom	2019-Dec-04	EPI_ISL_428534	A/England/582/2019	UK Health Security Agency - Colindale	UK Health Security Agency - Colindale	
EPI1732179	HA	United Kingdom	2019-Dec-04	EPI_ISL_428533	A/England/581/2019	UK Health Security Agency - Colindale	UK Health Security Agency - Colindale	
EPI1732171	HA	United Kingdom	2019-Dec-03	EPI_ISL_428532	A/England/580/2019	UK Health Security Agency - Colindale	UK Health Security Agency - Colindale	
EPI1732163	HA	United Kingdom	2019-Dec-03	EPI_ISL_428531	A/England/194960708/2019	UK Health Security Agency - Colindale	UK Health Security Agency - Colindale	
EPI1732155	HA	United Kingdom	2019-Dec-02	EPI_ISL_428530	A/England/579/2019	UK Health Security Agency - Colindale	UK Health Security Agency - Colindale	
EPI1732147	HA	United Kingdom	2019-Dec-05	EPI_ISL_428529	A/England/575/2019	UK Health Security Agency - Colindale	UK Health Security Agency - Colindale	

Segment ID	Segment	Country	Collection date	Isolate-ID	Isolate name	Originating Lab	Submitting Lab	Authors
EPI1732139	HA	United Kingdom	2019-Dec-02	EPI_ISL_428528	A/England/194960683/2019	UK Health Security Agency - Colindale	UK Health Security Agency - Colindale	
EPI1732131	HA	United Kingdom	2019-Dec-02	EPI_ISL_428527	A/England/574/2019	UK Health Security Agency - Colindale	UK Health Security Agency - Colindale	
EPI1732123	HA	United Kingdom	2019-Dec-02	EPI_ISL_428526	A/England/573/2019	UK Health Security Agency - Colindale	UK Health Security Agency - Colindale	
EPI1732115	HA	United Kingdom	2019-Dec-02	EPI_ISL_428525	A/England/569/2019	UK Health Security Agency - Colindale	UK Health Security Agency - Colindale	
EPI1732107	HA	United Kingdom	2019-Dec-02	EPI_ISL_428524	A/England/566/2019	UK Health Security Agency - Colindale	UK Health Security Agency - Colindale	
EPI1732099	HA	United Kingdom	2019-Nov-29	EPI_ISL_428523	A/England/565/2019	UK Health Security Agency - Colindale	UK Health Security Agency - Colindale	
EPI1732091	HA	United Kingdom	2019-Dec-02	EPI_ISL_428522	A/England/530/2019	UK Health Security Agency - Colindale	UK Health Security Agency - Colindale	
EPI1732083	HA	United Kingdom	2019-Nov-22	EPI_ISL_428521	A/England/194780194/2019	UK Health Security Agency - Colindale	UK Health Security Agency - Colindale	
EPI1732075	HA	United Kingdom	2019-Nov-12	EPI_ISL_428520	A/England/370/2019	UK Health Security Agency - Colindale	UK Health Security Agency - Colindale	
EPI1732067	HA	United Kingdom	2019-Dec-13	EPI_ISL_428519	A/England/195180880/2019	UK Health Security Agency - Colindale	UK Health Security Agency - Colindale	
EPI1732065	HA	United Kingdom	2019-Dec-16	EPI_ISL_428518	A/England/195160661/2019	UK Health Security Agency - Colindale	UK Health Security Agency - Colindale	
EPI1732063	HA	United Kingdom	2019-Dec-16	EPI_ISL_428517	A/England/195160654/2019	UK Health Security Agency - Colindale	UK Health Security Agency - Colindale	
EPI1732061	HA	United Kingdom	2019-Dec-11	EPI_ISL_428516	A/England/803/2019	UK Health Security Agency - Colindale	UK Health Security Agency - Colindale	

Segment ID	Segment	Country	Collection date	Isolate-ID	Isolate name	Originating Lab	Submitting Lab	Authors
EPI1732059	HA	United Kingdom	2019-Dec-13	EPI_ISL_428515	A/England/757/2019	UK Health Security Agency - Colindale	UK Health Security Agency - Colindale	
EPI1732057	HA	United Kingdom	2019-Dec-10	EPI_ISL_428514	A/England/195140661/2019	UK Health Security Agency - Colindale	UK Health Security Agency - Colindale	
EPI1732055	HA	United Kingdom	2019-Dec-16	EPI_ISL_428513	A/England/195140657/2019	UK Health Security Agency - Colindale	UK Health Security Agency - Colindale	
EPI1732050	HA	United Kingdom	2019-Dec-12	EPI_ISL_428510	A/England/740/2019	UK Health Security Agency - Colindale	UK Health Security Agency - Colindale	
EPI1732048	HA	United Kingdom	2019-Dec-12	EPI_ISL_428509	A/England/736/2019	UK Health Security Agency - Colindale	UK Health Security Agency - Colindale	
EPI1732046	HA	United Kingdom	2019-Dec-08	EPI_ISL_428508	A/England/729/2019	UK Health Security Agency - Colindale	UK Health Security Agency - Colindale	
EPI1732044	HA	United Kingdom	2019-Dec-12	EPI_ISL_428507	A/England/713/2019	UK Health Security Agency - Colindale	UK Health Security Agency - Colindale	
EPI1732038	HA	United Kingdom	2019-Nov-30	EPI_ISL_428504	A/England/195080680/2019	UK Health Security Agency - Colindale	UK Health Security Agency - Colindale	
EPI1732036	HA	United Kingdom	2019-Dec-04	EPI_ISL_428503	A/England/585/2019	UK Health Security Agency - Colindale	UK Health Security Agency - Colindale	
EPI1732034	HA	United Kingdom	2019-Dec-04	EPI_ISL_428502	A/England/584/2019	UK Health Security Agency - Colindale	UK Health Security Agency - Colindale	
EPI1732032	HA	United Kingdom	2019-Nov-26	EPI_ISL_428501	A/England/577/2019	UK Health Security Agency - Colindale	UK Health Security Agency - Colindale	
EPI1732030	HA	United Kingdom	2019-Dec-03	EPI_ISL_428500	A/England/576/2019	UK Health Security Agency - Colindale	UK Health Security Agency - Colindale	
EPI1732026	HA	United Kingdom	2019-Dec-02	EPI_ISL_428497	A/England/546/2019	UK Health Security Agency - Colindale	UK Health Security Agency - Colindale	

Segment ID	Segment	Country	Collection date	Isolate-ID	Isolate name	Originating Lab	Submitting Lab	Authors
EPI1732021	HA	United Kingdom	2019-Nov-25	EPI_ISL_428494	A/England/531/2019	UK Health Security Agency - Colindale	UK Health Security Agency - Colindale	
EPI1732018	HA	United Kingdom	2019-Nov-22	EPI_ISL_428492	A/England/194780198/2019	UK Health Security Agency - Colindale	UK Health Security Agency - Colindale	
EPI1719077	HA	United Kingdom	2019-Nov-13	EPI_ISL_418458	A/England/599/2019	UK Health Security Agency - Colindale	Crick Worldwide Influenza Centre	
EPI1719075	HA	United Kingdom	2019-Nov-20	EPI_ISL_418457	A/England/476/2019	UK Health Security Agency - Colindale	Crick Worldwide Influenza Centre	
EPI1719069	HA	United Kingdom	2019-Nov-13	EPI_ISL_418454	A/England/387/2019	UK Health Security Agency - Colindale	Crick Worldwide Influenza Centre	
EPI1719065	HA	United Kingdom	2019-Nov-17	EPI_ISL_418452	A/England/194820759/2019	UK Health Security Agency - Colindale	Crick Worldwide Influenza Centre	
EPI1719063	HA	United Kingdom	2019-Nov-12	EPI_ISL_418451	A/England/194660616/2019	UK Health Security Agency - Colindale	Crick Worldwide Influenza Centre	
EPI1719061	HA	United Kingdom	2019-Nov-04	EPI_ISL_418450	A/England/194560464/2019	UK Health Security Agency - Colindale	Crick Worldwide Influenza Centre	
EPI1716309	HA	United Kingdom	2019-Dec-08	EPI_ISL_416213	A/England/675/2019	UK Health Security Agency - Colindale	Crick Worldwide Influenza Centre	
EPI1716307	HA	United Kingdom	2019-Dec-05	EPI_ISL_416212	A/England/651/2019	UK Health Security Agency - Colindale	Crick Worldwide Influenza Centre	
EPI1716305	HA	United Kingdom	2019-Dec-07	EPI_ISL_416211	A/England/648/2019	UK Health Security Agency - Colindale	Crick Worldwide Influenza Centre	
EPI1716303	HA	United Kingdom	2019-Dec-05	EPI_ISL_416210	A/England/645/2019	UK Health Security Agency - Colindale	Crick Worldwide Influenza Centre	
EPI1716301	HA	United Kingdom	2019-Nov-29	EPI_ISL_416209	A/England/612/2019	UK Health Security Agency - Colindale	Crick Worldwide Influenza Centre	
EPI1716299	HA	United Kingdom	2019-Nov-29	EPI_ISL_416208	A/England/551/2019	UK Health Security Agency - Colindale	Crick Worldwide Influenza Centre	
EPI1716297	HA	United Kingdom	2019-Nov-25	EPI_ISL_416207	A/England/531/2019	UK Health Security Agency - Colindale	Crick Worldwide Influenza Centre	
EPI1716295	HA	United Kingdom	2019-Nov-29	EPI_ISL_416206	A/England/521/2019	UK Health Security Agency - Colindale	Crick Worldwide Influenza Centre	
EPI1716293	HA	United Kingdom	2019-Nov-26	EPI_ISL_416205	A/England/513/2019	UK Health Security Agency - Colindale	Crick Worldwide Influenza Centre	
EPI1716291	HA	United Kingdom	2019-Nov-24	EPI_ISL_416204	A/England/194840298/2019	UK Health Security Agency - Colindale	Crick Worldwide Influenza Centre	

Segment ID	Segment	Country	Collection date	Isolate-ID	Isolate name	Originating Lab	Submitting Lab	Authors
EPI1716289	HA	United Kingdom	2019-Nov-25	EPI_ISL_416203	A/England/194840192/2019	UK Health Security Agency - Colindale	Crick Worldwide Influenza Centre	
EPI1708666	HA	United Kingdom	2020-Jan-09	EPI_ISL_414711	A/England/11099/2020	U.S. Air Force School of Aerospace Medicine	U.S. Air Force School of Aerospace Medicine	Gruner, W.E.; Fries, A.C.; Garrett, C.M.; Powell, M.L.; Hanson, J.F.; DeMarcus, L.S.; Sjoberg, P.A.; Bogue, A.L.; Robbins, A.S.; Macias, E.A.; Lambert, A.W.; Dane, D.J.
EPI1706243	HA	United Kingdom	2020-Jan-06	EPI_ISL_414060	A/England/10775/2020	U.S. Air Force School of Aerospace Medicine	U.S. Air Force School of Aerospace Medicine	Gruner, W.E.; Fries, A.C.; Garrett, C.M.; Powell, M.L.; Hanson, J.F.; DeMarcus, L.S.; Sjoberg, P.A.; Bogue, A.L.; Robbins, A.S.; Macias, E.A.; Lambert, A.W.; Dane, D.J.
EPI1706239	HA	United Kingdom	2019-Dec-22	EPI_ISL_414059	A/England/10774/2019	U.S. Air Force School of Aerospace Medicine	U.S. Air Force School of Aerospace Medicine	Gruner, W.E.; Fries, A.C.; Garrett, C.M.; Powell, M.L.; Hanson, J.F.; DeMarcus, L.S.; Sjoberg, P.A.; Bogue, A.L.; Robbins, A.S.; Macias, E.A.; Lambert, A.W.; Dane, D.J.
EPI1706231	HA	United Kingdom	2019-Dec-25	EPI_ISL_414058	A/England/10773/2019	U.S. Air Force School of Aerospace Medicine	U.S. Air Force School of Aerospace Medicine	Gruner, W.E.; Fries, A.C.; Garrett, C.M.; Powell, M.L.; Hanson, J.F.; DeMarcus, L.S.; Sjoberg, P.A.; Bogue, A.L.; Robbins, A.S.; Macias, E.A.; Lambert, A.W.; Dane, D.J.
EPI1706223	HA	United Kingdom	2019-Dec-24	EPI_ISL_414057	A/England/10772/2019	U.S. Air Force School of Aerospace Medicine	U.S. Air Force School of Aerospace Medicine	Gruner, W.E.; Fries, A.C.; Garrett, C.M.; Powell, M.L.; Hanson, J.F.; DeMarcus, L.S.; Sjoberg, P.A.; Bogue, A.L.; Robbins, A.S.; Macias, E.A.; Lambert, A.W.; Dane, D.J.
EPI1698915	HA	United Kingdom	2019-Dec-13	EPI_ISL_412475	A/England/10455/2019	U.S. Air Force School of Aerospace Medicine	U.S. Air Force School of Aerospace Medicine	Gruner, W.E.; Fries, A.C.; Garrett, C.M.; Powell, M.L.; Hanson, J.F.; DeMarcus, L.S.; Sjoberg, P.A.; Bogue, A.L.; Robbins, A.S.; Macias, E.A.; Lambert, A.W.; Dane, D.J.

Segment ID	Segment	Country	Collection date	Isolate-ID	Isolate name	Originating Lab	Submitting Lab	Authors
EPI1689464	HA	United Kingdom	2019-Dec-09	EPI_ISL_409978	A/England/670/2019	UK Health Security Agency - Colindale	Crick Worldwide Influenza Centre	
EPI1689462	HA	United Kingdom	2019-Dec-09	EPI_ISL_409977	A/England/652/2019	UK Health Security Agency - Colindale	Crick Worldwide Influenza Centre	
EPI1689460	HA	United Kingdom	2019-Dec-23	EPI_ISL_409976	A/England/195200780/2019	UK Health Security Agency - Colindale	Crick Worldwide Influenza Centre	
EPI1689458	HA	United Kingdom	2019-Dec-10	EPI_ISL_409975	A/England/195160658/2019	UK Health Security Agency - Colindale	Crick Worldwide Influenza Centre	
EPI1679281	HA	United Kingdom	2019-Dec-06	EPI_ISL_408384	A/England/665/2019	UK Health Security Agency - Colindale	UK Health Security Agency - Colindale	Thompson,C.
EPI1679273	HA	United Kingdom	2019-Dec-06	EPI_ISL_408383	A/England/663/2019	UK Health Security Agency - Colindale	UK Health Security Agency - Colindale	Thompson,C.
EPI1679265	HA	United Kingdom	2019-Dec-06	EPI_ISL_408382	A/England/195040736/2019	UK Health Security Agency - Colindale	UK Health Security Agency - Colindale	Thompson,C.
EPI1679257	HA	United Kingdom	2019-Dec-02	EPI_ISL_408381	A/England/195040727/2019	UK Health Security Agency - Colindale	UK Health Security Agency - Colindale	Thompson,C.
EPI1679249	HA	United Kingdom	2019-Dec-05	EPI_ISL_408380	A/England/647/2019	UK Health Security Agency - Colindale	UK Health Security Agency - Colindale	Thompson,C.
EPI1679241	HA	United Kingdom	2019-Dec-05	EPI_ISL_408379	A/England/646/2019	UK Health Security Agency - Colindale	UK Health Security Agency - Colindale	Thompson,C.
EPI1679233	HA	United Kingdom	2019-Dec-05	EPI_ISL_408378	A/England/195020685/2019	UK Health Security Agency - Colindale	UK Health Security Agency - Colindale	Thompson,C.
EPI1679225	HA	United Kingdom	2019-Dec-05	EPI_ISL_408377	A/England/195020684/2019	UK Health Security Agency - Colindale	UK Health Security Agency - Colindale	Thompson,C.
EPI1679217	HA	United Kingdom	2019-Dec-05	EPI_ISL_408376	A/England/645/2019	UK Health Security Agency - Colindale	UK Health Security Agency - Colindale	Thompson,C.
EPI1679209	HA	United Kingdom	2019-Dec-05	EPI_ISL_408375	A/England/644/2019	UK Health Security Agency - Colindale	UK Health Security Agency - Colindale	Thompson,C.

Segment ID	Segment	Country	Collection date	Isolate-ID	Isolate name	Originating Lab	Submitting Lab	Authors
EPI1679201	HA	United Kingdom	2019-Dec-06	EPI_ISL_408374	A/England/195020680/2019	UK Health Security Agency - Colindale	UK Health Security Agency - Colindale	Thompson,C.
EPI1679193	HA	United Kingdom	2019-Dec-06	EPI_ISL_408373	A/England/643/2019	UK Health Security Agency - Colindale	UK Health Security Agency - Colindale	Thompson,C.
EPI1679185	HA	United Kingdom	2019-Dec-08	EPI_ISL_408372	A/England/636/2019	UK Health Security Agency - Colindale	UK Health Security Agency - Colindale	Thompson,C.
EPI1679177	HA	United Kingdom	2019-Dec-03	EPI_ISL_408371	A/England/635/2019	UK Health Security Agency - Colindale	UK Health Security Agency - Colindale	Thompson,C.
EPI1679169	HA	United Kingdom	2019-Dec-04	EPI_ISL_408370	A/England/195000649/2019	UK Health Security Agency - Colindale	UK Health Security Agency - Colindale	Thompson,C.
EPI1679153	HA	United Kingdom	2019-Dec-04	EPI_ISL_408368	A/England/634/2019	UK Health Security Agency - Colindale	UK Health Security Agency - Colindale	Thompson,C.
EPI1679145	HA	United Kingdom	2019-Dec-04	EPI_ISL_408367	A/England/633/2019	UK Health Security Agency - Colindale	UK Health Security Agency - Colindale	Thompson,C.
EPI1679137	HA	United Kingdom	2019-Dec-03	EPI_ISL_408366	A/England/632/2019	UK Health Security Agency - Colindale	UK Health Security Agency - Colindale	Thompson,C.
EPI1679129	HA	United Kingdom	2019-Dec-03	EPI_ISL_408365	A/England/631/2019	UK Health Security Agency - Colindale	UK Health Security Agency - Colindale	Thompson,C.
EPI1679121	HA	United Kingdom	2019-Dec-01	EPI_ISL_408364	A/England/630/2019	UK Health Security Agency - Colindale	UK Health Security Agency - Colindale	Thompson,C.
EPI1679113	HA	United Kingdom	2019-Dec-09	EPI_ISL_408363	A/England/629/2019	UK Health Security Agency - Colindale	UK Health Security Agency - Colindale	Thompson,C.
EPI1679105	HA	United Kingdom	2019-Dec-03	EPI_ISL_408362	A/England/628/2019	UK Health Security Agency - Colindale	UK Health Security Agency - Colindale	Thompson,C.
EPI1679097	HA	United Kingdom	2019-Dec-04	EPI_ISL_408361	A/England/627/2019	UK Health Security Agency - Colindale	UK Health Security Agency - Colindale	Thompson,C.

Segment ID	Segment	Country	Collection date	Isolate-ID	Isolate name	Originating Lab	Submitting Lab	Authors
EPI1679089	HA	United Kingdom	2019-Dec-02	EPI_ISL_408360	A/England/626/2019	UK Health Security Agency - Colindale	UK Health Security Agency - Colindale	Thompson,C.
EPI1679081	HA	United Kingdom	2019-Dec-01	EPI_ISL_408359	A/England/625/2019	UK Health Security Agency - Colindale	UK Health Security Agency - Colindale	Thompson,C.
EPI1679073	HA	United Kingdom	2019-Dec-04	EPI_ISL_408358	A/England/624/2019	UK Health Security Agency - Colindale	UK Health Security Agency - Colindale	Thompson,C.
EPI1679065	HA	United Kingdom	2019-Dec-04	EPI_ISL_408357	A/England/623/2019	UK Health Security Agency - Colindale	UK Health Security Agency - Colindale	Thompson,C.
EPI1679057	HA	United Kingdom	2019-Nov-30	EPI_ISL_408356	A/England/615/2019	UK Health Security Agency - Colindale	UK Health Security Agency - Colindale	Thompson,C.
EPI1679049	HA	United Kingdom	2019-Nov-28	EPI_ISL_408355	A/England/614/2019	UK Health Security Agency - Colindale	UK Health Security Agency - Colindale	Thompson,C.
EPI1679041	HA	United Kingdom	2019-Nov-29	EPI_ISL_408354	A/England/194980832/2019	UK Health Security Agency - Colindale	UK Health Security Agency - Colindale	Thompson,C.
EPI1679033	HA	United Kingdom	2019-Nov-27	EPI_ISL_408353	A/England/613/2019	UK Health Security Agency - Colindale	UK Health Security Agency - Colindale	Thompson,C.
EPI1679025	HA	United Kingdom	2019-Nov-29	EPI_ISL_408352	A/England/612/2019	UK Health Security Agency - Colindale	UK Health Security Agency - Colindale	Thompson,C.
EPI1679017	HA	United Kingdom	2019-Nov-29	EPI_ISL_408351	A/England/610/2019	UK Health Security Agency - Colindale	UK Health Security Agency - Colindale	Thompson,C.
EPI1679009	HA	United Kingdom	2019-Nov-29	EPI_ISL_408350	A/England/609/2019	UK Health Security Agency - Colindale	UK Health Security Agency - Colindale	Thompson,C.
EPI1679001	HA	United Kingdom	2019-Nov-29	EPI_ISL_408349	A/England/194980826/2019	UK Health Security Agency - Colindale	UK Health Security Agency - Colindale	Thompson,C.
EPI1678993	HA	United Kingdom	2019-Dec-01	EPI_ISL_408348	A/England/194980825/2019	UK Health Security Agency - Colindale	UK Health Security Agency - Colindale	Thompson,C.

Segment ID	Segment	Country	Collection date	Isolate-ID	Isolate name	Originating Lab	Submitting Lab	Authors
EPI1678985	HA	United Kingdom	2019-Nov-30	EPI_ISL_408347	A/England/608/2019	UK Health Security Agency - Colindale	UK Health Security Agency - Colindale	Thompson,C.
EPI1678977	HA	United Kingdom	2019-Nov-30	EPI_ISL_408346	A/England/607/2019	UK Health Security Agency - Colindale	UK Health Security Agency - Colindale	Thompson,C.
EPI1678969	HA	United Kingdom	2019-Nov-30	EPI_ISL_408345	A/England/606/2019	UK Health Security Agency - Colindale	UK Health Security Agency - Colindale	Thompson,C.
EPI1678961	HA	United Kingdom	2019-Dec-02	EPI_ISL_408344	A/England/194980819/2019	UK Health Security Agency - Colindale	UK Health Security Agency - Colindale	Thompson,C.
EPI1678953	HA	United Kingdom	2019-Nov-29	EPI_ISL_408343	A/England/598/2019	UK Health Security Agency - Colindale	UK Health Security Agency - Colindale	Thompson,C.
EPI1678945	HA	United Kingdom	2019-Nov-30	EPI_ISL_408342	A/England/194980817/2019	UK Health Security Agency - Colindale	UK Health Security Agency - Colindale	Thompson,C.
EPI1678937	HA	United Kingdom	2019-Dec-01	EPI_ISL_408341	A/England/597/2019	UK Health Security Agency - Colindale	UK Health Security Agency - Colindale	Thompson,C.
EPI1678929	HA	United Kingdom	2019-Nov-29	EPI_ISL_408340	A/England/194980815/2019	UK Health Security Agency - Colindale	UK Health Security Agency - Colindale	Thompson,C.
EPI1678921	HA	United Kingdom	2019-Dec-02	EPI_ISL_408339	A/England/194980814/2019	UK Health Security Agency - Colindale	UK Health Security Agency - Colindale	Thompson,C.
EPI1678913	HA	United Kingdom	2019-Dec-01	EPI_ISL_408338	A/England/596/2019	UK Health Security Agency - Colindale	UK Health Security Agency - Colindale	Thompson,C.
EPI1678905	HA	United Kingdom	2019-Dec-01	EPI_ISL_408337	A/England/194980812/2019	UK Health Security Agency - Colindale	UK Health Security Agency - Colindale	Thompson,C.
EPI1678897	HA	United Kingdom	2019-Dec-02	EPI_ISL_408336	A/England/194980811/2019	UK Health Security Agency - Colindale	UK Health Security Agency - Colindale	Thompson,C.
EPI1678889	HA	United Kingdom	2019-Dec-01	EPI_ISL_408335	A/England/595/2019	UK Health Security Agency - Colindale	UK Health Security Agency - Colindale	Thompson,C.

Segment ID	Segment	Country	Collection date	Isolate-ID	Isolate name	Originating Lab	Submitting Lab	Authors
EPI1678881	HA	United Kingdom	2019-Dec-01	EPI_ISL_408334	A/England/594/2019	UK Health Security Agency - Colindale	UK Health Security Agency - Colindale	Thompson,C.
EPI1678873	HA	United Kingdom	2019-Dec-01	EPI_ISL_408333	A/England/593/2019	UK Health Security Agency - Colindale	UK Health Security Agency - Colindale	Thompson,C.
EPI1678865	HA	United Kingdom	2019-Nov-30	EPI_ISL_408332	A/England/592/2019	UK Health Security Agency - Colindale	UK Health Security Agency - Colindale	Thompson,C.
EPI1678849	HA	United Kingdom	2019-Dec-02	EPI_ISL_408330	A/England/591/2019	UK Health Security Agency - Colindale	UK Health Security Agency - Colindale	Thompson,C.
EPI1678833	HA	United Kingdom	2019-Dec-03	EPI_ISL_408328	A/England/194980802/2019	UK Health Security Agency - Colindale	UK Health Security Agency - Colindale	Thompson,C.
EPI1678825	HA	United Kingdom	2019-Dec-03	EPI_ISL_408327	A/England/194980801/2019	UK Health Security Agency - Colindale	UK Health Security Agency - Colindale	Thompson,C.
EPI1678817	HA	United Kingdom	2019-Dec-03	EPI_ISL_408326	A/England/194980799/2019	UK Health Security Agency - Colindale	UK Health Security Agency - Colindale	Thompson,C.
EPI1678801	HA	United Kingdom	2019-Dec-02	EPI_ISL_408324	A/England/194980796/2019	UK Health Security Agency - Colindale	UK Health Security Agency - Colindale	Thompson,C.
EPI1678793	HA	United Kingdom	2019-Dec-02	EPI_ISL_408323	A/England/194980795/2019	UK Health Security Agency - Colindale	UK Health Security Agency - Colindale	Thompson,C.
EPI1678785	HA	United Kingdom	2019-Dec-02	EPI_ISL_408322	A/England/590/2019	UK Health Security Agency - Colindale	UK Health Security Agency - Colindale	Thompson,C.
EPI1678777	HA	United Kingdom	2019-Dec-04	EPI_ISL_408321	A/England/194980793/2019	UK Health Security Agency - Colindale	UK Health Security Agency - Colindale	Thompson,C.
EPI1678769	HA	United Kingdom	2019-Nov-29	EPI_ISL_408320	A/England/572/2019	UK Health Security Agency - Colindale	UK Health Security Agency - Colindale	Thompson,C.
EPI1678761	HA	United Kingdom	2019-Dec-02	EPI_ISL_408319	A/England/571/2019	UK Health Security Agency - Colindale	UK Health Security Agency - Colindale	Thompson,C.

Segment ID	Segment	Country	Collection date	Isolate-ID	Isolate name	Originating Lab	Submitting Lab	Authors
EPI1678745	HA	United Kingdom	2019-Dec-05	EPI_ISL_408317	A/England/570/2019	UK Health Security Agency - Colindale	UK Health Security Agency - Colindale	Thompson,C.
EPI1678737	HA	United Kingdom	2019-Dec-05	EPI_ISL_408316	A/England/568/2019	UK Health Security Agency - Colindale	UK Health Security Agency - Colindale	Thompson,C.
EPI1678729	HA	United Kingdom	2019-Nov-30	EPI_ISL_408315	A/England/567/2019	UK Health Security Agency - Colindale	UK Health Security Agency - Colindale	Thompson,C.
EPI1678721	HA	United Kingdom	2019-Nov-28	EPI_ISL_408314	A/England/194940533/2019	UK Health Security Agency - Colindale	UK Health Security Agency - Colindale	Thompson,C.
EPI1678713	HA	United Kingdom	2019-Nov-29	EPI_ISL_408313	A/England/194940532/2019	UK Health Security Agency - Colindale	UK Health Security Agency - Colindale	Thompson,C.
EPI1678705	HA	United Kingdom	2019-Nov-28	EPI_ISL_408312	A/England/194940531/2019	UK Health Security Agency - Colindale	UK Health Security Agency - Colindale	Thompson,C.
EPI1678697	HA	United Kingdom	2019-Nov-25	EPI_ISL_408311	A/England/564/2019	UK Health Security Agency - Colindale	UK Health Security Agency - Colindale	Thompson,C.
EPI1678689	HA	United Kingdom	2019-Nov-28	EPI_ISL_408310	A/England/194940529/2019	UK Health Security Agency - Colindale	UK Health Security Agency - Colindale	Thompson,C.
EPI1678681	HA	United Kingdom	2019-Nov-25	EPI_ISL_408309	A/England/563/2019	UK Health Security Agency - Colindale	UK Health Security Agency - Colindale	Thompson,C.
EPI1678673	HA	United Kingdom	2019-Nov-27	EPI_ISL_408308	A/England/562/2019	UK Health Security Agency - Colindale	UK Health Security Agency - Colindale	Thompson,C.
EPI1678665	HA	United Kingdom	2019-Nov-27	EPI_ISL_408307	A/England/561/2019	UK Health Security Agency - Colindale	UK Health Security Agency - Colindale	Thompson,C.
EPI1678657	HA	United Kingdom	2019-Nov-26	EPI_ISL_408306	A/England/194940525/2019	UK Health Security Agency - Colindale	UK Health Security Agency - Colindale	Thompson,C.
EPI1678649	HA	United Kingdom	2019-Nov-25	EPI_ISL_408305	A/England/194940524/2019	UK Health Security Agency - Colindale	UK Health Security Agency - Colindale	Thompson,C.

Segment ID	Segment	Country	Collection date	Isolate-ID	Isolate name	Originating Lab	Submitting Lab	Authors
EPI1678641	HA	United Kingdom	2019-Nov-28	EPI_ISL_408304	A/England/560/2019	UK Health Security Agency - Colindale	UK Health Security Agency - Colindale	Thompson,C.
EPI1678633	HA	United Kingdom	2019-Nov-29	EPI_ISL_408303	A/England/559/2019	UK Health Security Agency - Colindale	UK Health Security Agency - Colindale	Thompson,C.
EPI1678625	HA	United Kingdom	2019-Nov-28	EPI_ISL_408302	A/England/558/2019	UK Health Security Agency - Colindale	UK Health Security Agency - Colindale	Thompson,C.
EPI1678617	HA	United Kingdom	2019-Nov-26	EPI_ISL_408301	A/England/557/2019	UK Health Security Agency - Colindale	UK Health Security Agency - Colindale	Thompson,C.
EPI1678609	HA	United Kingdom	2019-Nov-26	EPI_ISL_408300	A/England/556/2019	UK Health Security Agency - Colindale	UK Health Security Agency - Colindale	Thompson,C.
EPI1678601	HA	United Kingdom	2019-Nov-26	EPI_ISL_408299	A/England/194940517/2019	UK Health Security Agency - Colindale	UK Health Security Agency - Colindale	Thompson,C.
EPI1678593	HA	United Kingdom	2019-Nov-26	EPI_ISL_408298	A/England/555/2019	UK Health Security Agency - Colindale	UK Health Security Agency - Colindale	Thompson,C.
EPI1678585	HA	United Kingdom	2019-Nov-26	EPI_ISL_408297	A/England/554/2019	UK Health Security Agency - Colindale	UK Health Security Agency - Colindale	Thompson,C.
EPI1678577	HA	United Kingdom	2019-Nov-26	EPI_ISL_408296	A/England/605/2019	UK Health Security Agency - Colindale	UK Health Security Agency - Colindale	Thompson,C.
EPI1678569	HA	United Kingdom	2019-Nov-29	EPI_ISL_408295	A/England/552/2019	UK Health Security Agency - Colindale	UK Health Security Agency - Colindale	Thompson,C.
EPI1678561	HA	United Kingdom	2019-Nov-29	EPI_ISL_408294	A/England/551/2019	UK Health Security Agency - Colindale	UK Health Security Agency - Colindale	Thompson,C.
EPI1678553	HA	United Kingdom	2019-Nov-29	EPI_ISL_408293	A/England/549/2019	UK Health Security Agency - Colindale	UK Health Security Agency - Colindale	Thompson,C.
EPI1678545	HA	United Kingdom	2019-Nov-30	EPI_ISL_408292	A/England/548/2019	UK Health Security Agency - Colindale	UK Health Security Agency - Colindale	Thompson,C.

Segment ID	Segment	Country	Collection date	Isolate-ID	Isolate name	Originating Lab	Submitting Lab	Authors
EPI1678537	HA	United Kingdom	2019-Dec-01	EPI_ISL_408291	A/England/194940503/2019	UK Health Security Agency - Colindale	UK Health Security Agency - Colindale	Thompson,C.
EPI1678529	HA	United Kingdom	2019-Nov-28	EPI_ISL_408290	A/England/194920611/2019	UK Health Security Agency - Colindale	UK Health Security Agency - Colindale	Thompson,C.
EPI1678521	HA	United Kingdom	2019-Dec-01	EPI_ISL_408289	A/England/194920610/2019	UK Health Security Agency - Colindale	UK Health Security Agency - Colindale	Thompson,C.
EPI1678513	HA	United Kingdom	2019-Nov-27	EPI_ISL_408288	A/England/194920609/2019	UK Health Security Agency - Colindale	UK Health Security Agency - Colindale	Thompson,C.
EPI1678505	HA	United Kingdom	2019-Nov-30	EPI_ISL_408287	A/England/539/2019	UK Health Security Agency - Colindale	UK Health Security Agency - Colindale	Thompson,C.
EPI1678497	HA	United Kingdom	2019-Nov-26	EPI_ISL_408286	A/England/537/2019	UK Health Security Agency - Colindale	UK Health Security Agency - Colindale	Thompson,C.
EPI1678489	HA	United Kingdom	2019-Nov-28	EPI_ISL_408285	A/England/604/2019	UK Health Security Agency - Colindale	UK Health Security Agency - Colindale	Thompson,C.
EPI1678481	HA	United Kingdom	2019-Nov-27	EPI_ISL_408284	A/England/194920599/2019	UK Health Security Agency - Colindale	UK Health Security Agency - Colindale	Thompson,C.
EPI1677261	HA	United Kingdom	2019-Nov-26	EPI_ISL_408120	A/England/535/2019	UK Health Security Agency - Colindale	UK Health Security Agency - Colindale	Thompson,C.
EPI1677253	HA	United Kingdom	2019-Nov-28	EPI_ISL_408119	A/England/194920595/2019	UK Health Security Agency - Colindale	UK Health Security Agency - Colindale	Thompson,C.
EPI1677245	HA	United Kingdom	2019-Nov-28	EPI_ISL_408118	A/England/533/2019	UK Health Security Agency - Colindale	UK Health Security Agency - Colindale	Thompson,C.
EPI1677237	HA	United Kingdom	2019-Dec-02	EPI_ISL_408117	A/England/529/2019	UK Health Security Agency - Colindale	UK Health Security Agency - Colindale	Thompson,C.
EPI1677229	HA	United Kingdom	2019-Dec-02	EPI_ISL_408116	A/England/528/2019	UK Health Security Agency - Colindale	UK Health Security Agency - Colindale	Thompson,C.

Segment ID	Segment	Country	Collection date	Isolate-ID	Isolate name	Originating Lab	Submitting Lab	Authors
EPI1677221	HA	United Kingdom	2019-Nov-28	EPI_ISL_408115	A/England/527/2019	UK Health Security Agency - Colindale	UK Health Security Agency - Colindale	Thompson,C.
EPI1677213	HA	United Kingdom	2019-Dec-02	EPI_ISL_408114	A/England/526/2019	UK Health Security Agency - Colindale	UK Health Security Agency - Colindale	Thompson,C.
EPI1677197	HA	United Kingdom	2019-Nov-29	EPI_ISL_408112	A/England/524/2019	UK Health Security Agency - Colindale	UK Health Security Agency - Colindale	Thompson,C.
EPI1677189	HA	United Kingdom	2019-Nov-27	EPI_ISL_408111	A/England/523/2019	UK Health Security Agency - Colindale	UK Health Security Agency - Colindale	Thompson,C.
EPI1677181	HA	United Kingdom	2019-Nov-27	EPI_ISL_408110	A/England/194900585/2019	UK Health Security Agency - Colindale	UK Health Security Agency - Colindale	Thompson,C.
EPI1677173	HA	United Kingdom	2019-Nov-29	EPI_ISL_408109	A/England/521/2019	UK Health Security Agency - Colindale	UK Health Security Agency - Colindale	Thompson,C.
EPI1677165	HA	United Kingdom	2019-Dec-02	EPI_ISL_408108	A/England/543/2019	UK Health Security Agency - Colindale	UK Health Security Agency - Colindale	Thompson,C.
EPI1677157	HA	United Kingdom	2019-Nov-29	EPI_ISL_408107	A/England/194900386/2019	UK Health Security Agency - Colindale	UK Health Security Agency - Colindale	Thompson,C.
EPI1677141	HA	United Kingdom	2019-Nov-25	EPI_ISL_408105	A/England/520/2019	UK Health Security Agency - Colindale	UK Health Security Agency - Colindale	Thompson,C.
EPI1677133	HA	United Kingdom	2019-Dec-02	EPI_ISL_408104	A/England/519/2019	UK Health Security Agency - Colindale	UK Health Security Agency - Colindale	Thompson,C.
EPI1677125	HA	United Kingdom	2019-Nov-28	EPI_ISL_408103	A/England/194900370/2019	UK Health Security Agency - Colindale	UK Health Security Agency - Colindale	Thompson,C.
EPI1677117	HA	United Kingdom	2019-Nov-29	EPI_ISL_408102	A/England/516/2019	UK Health Security Agency - Colindale	UK Health Security Agency - Colindale	Thompson,C.
EPI1677109	HA	United Kingdom	2019-Nov-27	EPI_ISL_408101	A/England/515/2019	UK Health Security Agency - Colindale	UK Health Security Agency - Colindale	Thompson,C.

Segment ID	Segment	Country	Collection date	Isolate-ID	Isolate name	Originating Lab	Submitting Lab	Authors
EPI1677101	HA	United Kingdom	2019-Nov-27	EPI_ISL_408100	A/England/514/2019	UK Health Security Agency - Colindale	UK Health Security Agency - Colindale	Thompson,C.
EPI1677093	HA	United Kingdom	2019-Nov-26	EPI_ISL_408099	A/England/513/2019	UK Health Security Agency - Colindale	UK Health Security Agency - Colindale	Thompson,C.
EPI1677085	HA	United Kingdom	2019-Nov-26	EPI_ISL_408098	A/England/194880448/2019	UK Health Security Agency - Colindale	UK Health Security Agency - Colindale	Thompson,C.
EPI1677077	HA	United Kingdom	2019-Nov-26	EPI_ISL_408097	A/England/194880445/2019	UK Health Security Agency - Colindale	UK Health Security Agency - Colindale	Thompson,C.
EPI1677069	HA	United Kingdom	2019-Nov-26	EPI_ISL_408096	A/England/194880444/2019	UK Health Security Agency - Colindale	UK Health Security Agency - Colindale	Thompson,C.
EPI1677061	HA	United Kingdom	2019-Nov-25	EPI_ISL_408095	A/England/194880443/2019	UK Health Security Agency - Colindale	UK Health Security Agency - Colindale	Thompson,C.
EPI1677053	HA	United Kingdom	2019-Nov-27	EPI_ISL_408094	A/England/512/2019	UK Health Security Agency - Colindale	UK Health Security Agency - Colindale	Thompson,C.
EPI1677045	HA	United Kingdom	2019-Nov-26	EPI_ISL_408093	A/England/511/2019	UK Health Security Agency - Colindale	UK Health Security Agency - Colindale	Thompson,C.
EPI1677037	HA	United Kingdom	2019-Nov-27	EPI_ISL_408092	A/England/494/2019	UK Health Security Agency - Colindale	UK Health Security Agency - Colindale	Thompson,C.
EPI1677029	HA	United Kingdom	2019-Nov-27	EPI_ISL_408091	A/England/493/2019	UK Health Security Agency - Colindale	UK Health Security Agency - Colindale	Thompson,C.
EPI1677021	HA	United Kingdom	2019-Nov-24	EPI_ISL_408090	A/England/601/2019	UK Health Security Agency - Colindale	UK Health Security Agency - Colindale	Thompson,C.
EPI1677013	HA	United Kingdom	2019-Nov-24	EPI_ISL_408089	A/England/194860507/2019	UK Health Security Agency - Colindale	UK Health Security Agency - Colindale	Thompson,C.
EPI1677005	HA	United Kingdom	2019-Nov-21	EPI_ISL_408088	A/England/194860502/2019	UK Health Security Agency - Colindale	UK Health Security Agency - Colindale	Thompson,C.

Segment ID	Segment	Country	Collection date	Isolate-ID	Isolate name	Originating Lab	Submitting Lab	Authors
EPI1676997	HA	United Kingdom	2019-Nov-21	EPI_ISL_408087	A/England/194860500/2019	UK Health Security Agency - Colindale	UK Health Security Agency - Colindale	Thompson,C.
EPI1676989	HA	United Kingdom	2019-Nov-21	EPI_ISL_408086	A/England/194860499/2019	UK Health Security Agency - Colindale	UK Health Security Agency - Colindale	Thompson,C.
EPI1676981	HA	United Kingdom	2019-Nov-23	EPI_ISL_408085	A/England/194860492/2019	UK Health Security Agency - Colindale	UK Health Security Agency - Colindale	Thompson,C.
EPI1676973	HA	United Kingdom	2019-Nov-24	EPI_ISL_408084	A/England/194860490/2019	UK Health Security Agency - Colindale	UK Health Security Agency - Colindale	Thompson,C.
EPI1676965	HA	United Kingdom	2019-Nov-25	EPI_ISL_408083	A/England/491/2019	UK Health Security Agency - Colindale	UK Health Security Agency - Colindale	Thompson,C.
EPI1676949	HA	United Kingdom	2019-Nov-25	EPI_ISL_408081	A/England/194860481/2019	UK Health Security Agency - Colindale	UK Health Security Agency - Colindale	Thompson,C.
EPI1676941	HA	United Kingdom	2019-Nov-20	EPI_ISL_408080	A/England/486/2019	UK Health Security Agency - Colindale	UK Health Security Agency - Colindale	Thompson,C.
EPI1676933	HA	United Kingdom	2019-Nov-18	EPI_ISL_408079	A/England/194860472/2019	UK Health Security Agency - Colindale	UK Health Security Agency - Colindale	Thompson,C.
EPI1676925	HA	United Kingdom	2019-Nov-23	EPI_ISL_408078	A/England/485/2019	UK Health Security Agency - Colindale	UK Health Security Agency - Colindale	Thompson,C.
EPI1676917	HA	United Kingdom	2019-Nov-26	EPI_ISL_408077	A/England/510/2019	UK Health Security Agency - Colindale	UK Health Security Agency - Colindale	Thompson,C.
EPI1676909	HA	United Kingdom	2019-Nov-25	EPI_ISL_408076	A/England/509/2019	UK Health Security Agency - Colindale	UK Health Security Agency - Colindale	Thompson,C.
EPI1676901	HA	United Kingdom	2019-Nov-26	EPI_ISL_408075	A/England/507/2019	UK Health Security Agency - Colindale	UK Health Security Agency - Colindale	Thompson,C.
EPI1676885	HA	United Kingdom	2019-Nov-26	EPI_ISL_408073	A/England/506/2019	UK Health Security Agency - Colindale	UK Health Security Agency - Colindale	Thompson,C.

Segment ID	Segment	Country	Collection date	Isolate-ID	Isolate name	Originating Lab	Submitting Lab	Authors
EPI1676877	HA	United Kingdom	2019-Nov-26	EPI_ISL_408072	A/England/505/2019	UK Health Security Agency - Colindale	UK Health Security Agency - Colindale	Thompson,C.
EPI1676869	HA	United Kingdom	2019-Nov-25	EPI_ISL_408071	A/England/504/2019	UK Health Security Agency - Colindale	UK Health Security Agency - Colindale	Thompson,C.
EPI1676861	HA	United Kingdom	2019-Nov-25	EPI_ISL_408070	A/England/503/2019	UK Health Security Agency - Colindale	UK Health Security Agency - Colindale	Thompson,C.
EPI1676853	HA	United Kingdom	2019-Nov-26	EPI_ISL_408069	A/England/484/2019	UK Health Security Agency - Colindale	UK Health Security Agency - Colindale	Thompson,C.
EPI1676845	HA	United Kingdom	2019-Nov-22	EPI_ISL_408067	A/England/483/2019	UK Health Security Agency - Colindale	UK Health Security Agency - Colindale	Thompson,C.
EPI1676837	HA	United Kingdom	2019-Nov-20	EPI_ISL_408066	A/England/480/2019	UK Health Security Agency - Colindale	UK Health Security Agency - Colindale	Thompson,C.
EPI1676829	HA	United Kingdom	2019-Nov-19	EPI_ISL_408065	A/England/479/2019	UK Health Security Agency - Colindale	UK Health Security Agency - Colindale	Thompson,C.
EPI1676821	HA	United Kingdom	2019-Nov-22	EPI_ISL_408064	A/England/600/2019	UK Health Security Agency - Colindale	UK Health Security Agency - Colindale	Thompson,C.
EPI1676813	HA	United Kingdom	2019-Nov-24	EPI_ISL_408063	A/England/194840311/2019	UK Health Security Agency - Colindale	UK Health Security Agency - Colindale	Thompson,C.
EPI1676805	HA	United Kingdom	2019-Nov-25	EPI_ISL_408062	A/England/470/2019	UK Health Security Agency - Colindale	UK Health Security Agency - Colindale	Thompson,C.
EPI1676797	HA	United Kingdom	2019-Nov-26	EPI_ISL_408061	A/England/469/2019	UK Health Security Agency - Colindale	UK Health Security Agency - Colindale	Thompson,C.
EPI1676789	HA	United Kingdom	2019-Nov-26	EPI_ISL_408060	A/England/468/2019	UK Health Security Agency - Colindale	UK Health Security Agency - Colindale	Thompson,C.
EPI1676781	HA	United Kingdom	2019-Nov-25	EPI_ISL_408059	A/England/467/2019	UK Health Security Agency - Colindale	UK Health Security Agency - Colindale	Thompson,C.

Segment ID	Segment	Country	Collection date	Isolate-ID	Isolate name	Originating Lab	Submitting Lab	Authors
EPI1676773	HA	United Kingdom	2019-Nov-26	EPI_ISL_408058	A/England/466/2019	UK Health Security Agency - Colindale	UK Health Security Agency - Colindale	Thompson,C.
EPI1676765	HA	United Kingdom	2019-Nov-25	EPI_ISL_408057	A/England/194840192/2019	UK Health Security Agency - Colindale	UK Health Security Agency - Colindale	Thompson,C.
EPI1676757	HA	United Kingdom	2019-Nov-25	EPI_ISL_408056	A/England/194820277/2019	UK Health Security Agency - Colindale	UK Health Security Agency - Colindale	Thompson,C.
EPI1676749	HA	United Kingdom	2019-Nov-25	EPI_ISL_408055	A/England/461/2019	UK Health Security Agency - Colindale	UK Health Security Agency - Colindale	Thompson,C.
EPI1676741	HA	United Kingdom	2019-Nov-20	EPI_ISL_408054	A/England/460/2019	UK Health Security Agency - Colindale	UK Health Security Agency - Colindale	Thompson,C.
EPI1676733	HA	United Kingdom	2019-Nov-19	EPI_ISL_408053	A/England/194800313/2019	UK Health Security Agency - Colindale	UK Health Security Agency - Colindale	Thompson,C.
EPI1676725	HA	United Kingdom	2019-Nov-18	EPI_ISL_408052	A/England/454/2019	UK Health Security Agency - Colindale	UK Health Security Agency - Colindale	Thompson,C.
EPI1676717	HA	United Kingdom	2019-Nov-20	EPI_ISL_408051	A/England/451/2019	UK Health Security Agency - Colindale	UK Health Security Agency - Colindale	Thompson,C.
EPI1676709	HA	United Kingdom	2019-Nov-15	EPI_ISL_408050	A/England/403/2019	UK Health Security Agency - Colindale	UK Health Security Agency - Colindale	Thompson,C.
EPI1676693	HA	United Kingdom	2019-Nov-18	EPI_ISL_408048	A/England/400/2019	UK Health Security Agency - Colindale	UK Health Security Agency - Colindale	Thompson,C.
EPI1676685	HA	United Kingdom	2019-Nov-18	EPI_ISL_408047	A/England/399/2019	UK Health Security Agency - Colindale	UK Health Security Agency - Colindale	Thompson,C.
EPI1676677	HA	United Kingdom	2019-Nov-20	EPI_ISL_408046	A/England/398/2019	UK Health Security Agency - Colindale	UK Health Security Agency - Colindale	Thompson,C.
EPI1676669	HA	United Kingdom	2019-Nov-19	EPI_ISL_408045	A/England/397/2019	UK Health Security Agency - Colindale	UK Health Security Agency - Colindale	Thompson,C.

Segment ID	Segment	Country	Collection date	Isolate-ID	Isolate name	Originating Lab	Submitting Lab	Authors
EPI1676661	HA	United Kingdom	2019-Nov-19	EPI_ISL_408044	A/England/396/2019	UK Health Security Agency - Colindale	UK Health Security Agency - Colindale	Thompson,C.
EPI1676653	HA	United Kingdom	2019-Nov-20	EPI_ISL_408043	A/England/395/2019	UK Health Security Agency - Colindale	UK Health Security Agency - Colindale	Thompson,C.
EPI1676645	HA	United Kingdom	2019-Nov-18	EPI_ISL_408042	A/England/194740483/2019	UK Health Security Agency - Colindale	UK Health Security Agency - Colindale	Thompson,C.
EPI1676637	HA	United Kingdom	2019-Nov-18	EPI_ISL_408041	A/England/394/2019	UK Health Security Agency - Colindale	UK Health Security Agency - Colindale	Thompson,C.
EPI1676629	HA	United Kingdom	2019-Nov-18	EPI_ISL_408040	A/England/393/2019	UK Health Security Agency - Colindale	UK Health Security Agency - Colindale	Thompson,C.
EPI1676621	HA	United Kingdom	2019-Nov-19	EPI_ISL_408039	A/England/391/2019	UK Health Security Agency - Colindale	UK Health Security Agency - Colindale	Thompson,C.
EPI1676613	HA	United Kingdom	2019-Nov-19	EPI_ISL_408038	A/England/390/2019	UK Health Security Agency - Colindale	UK Health Security Agency - Colindale	Thompson,C.
EPI1676605	HA	United Kingdom	2019-Nov-19	EPI_ISL_408037	A/England/194740379/2019	UK Health Security Agency - Colindale	UK Health Security Agency - Colindale	Thompson,C.
EPI1676597	HA	United Kingdom	2019-Nov-18	EPI_ISL_408036	A/England/386/2019	UK Health Security Agency - Colindale	UK Health Security Agency - Colindale	Thompson,C.
EPI1676581	HA	United Kingdom	2019-Nov-15	EPI_ISL_408034	A/England/385/2019	UK Health Security Agency - Colindale	UK Health Security Agency - Colindale	Thompson,C.
EPI1676573	HA	United Kingdom	2019-Nov-18	EPI_ISL_408033	A/England/384/2019	UK Health Security Agency - Colindale	UK Health Security Agency - Colindale	Thompson,C.
EPI1676565	HA	United Kingdom	2019-Nov-18	EPI_ISL_408032	A/England/383/2019	UK Health Security Agency - Colindale	UK Health Security Agency - Colindale	Thompson,C.
EPI1676557	HA	United Kingdom	2019-Nov-18	EPI_ISL_408031	A/England/382/2019	UK Health Security Agency - Colindale	UK Health Security Agency - Colindale	Thompson,C.

Segment ID	Segment	Country	Collection date	Isolate-ID	Isolate name	Originating Lab	Submitting Lab	Authors
EPI1676549	HA	United Kingdom	2019-Nov-18	EPI_ISL_408030	A/England/378/2019	UK Health Security Agency - Colindale	UK Health Security Agency - Colindale	Thompson,C.
EPI1676541	HA	United Kingdom	2019-Nov-15	EPI_ISL_408029	A/England/377/2019	UK Health Security Agency - Colindale	UK Health Security Agency - Colindale	Thompson,C.
EPI1676533	HA	United Kingdom	2019-Nov-14	EPI_ISL_408028	A/England/376/2019	UK Health Security Agency - Colindale	UK Health Security Agency - Colindale	Thompson,C.
EPI1676525	HA	United Kingdom	2019-Nov-11	EPI_ISL_408027	A/England/194680695/2019	UK Health Security Agency - Colindale	UK Health Security Agency - Colindale	Thompson,C.
EPI1676517	HA	United Kingdom	2019-Nov-12	EPI_ISL_408026	A/England/369/2019	UK Health Security Agency - Colindale	UK Health Security Agency - Colindale	Thompson,C.
EPI1676509	HA	United Kingdom	2019-Nov-12	EPI_ISL_408025	A/England/368/2019	UK Health Security Agency - Colindale	UK Health Security Agency - Colindale	Thompson,C.
EPI1676501	HA	United Kingdom	2019-Nov-12	EPI_ISL_408024	A/England/367/2019	UK Health Security Agency - Colindale	UK Health Security Agency - Colindale	Thompson,C.
EPI1676493	HA	United Kingdom	2019-Nov-12	EPI_ISL_408023	A/England/371/2019	UK Health Security Agency - Colindale	UK Health Security Agency - Colindale	Thompson,C.
EPI1676485	HA	United Kingdom	2019-Nov-08	EPI_ISL_408022	A/England/194600762/2019	UK Health Security Agency - Colindale	UK Health Security Agency - Colindale	Thompson,C.
EPI1676477	HA	United Kingdom	2019-Nov-07	EPI_ISL_408021	A/England/365/2019	UK Health Security Agency - Colindale	UK Health Security Agency - Colindale	Thompson,C.
EPI1676469	HA	United Kingdom	2019-Dec-07	EPI_ISL_408020	A/England/648/2019	UK Health Security Agency - Colindale	UK Health Security Agency - Colindale	Thompson,C.
EPI1676466	HA	United Kingdom	2019-Dec-02	EPI_ISL_408018	A/England/611/2019	UK Health Security Agency - Colindale	UK Health Security Agency - Colindale	Thompson,C.
EPI1676464	HA	United Kingdom	2019-Dec-03	EPI_ISL_408017	A/England/194980800/2019	UK Health Security Agency - Colindale	UK Health Security Agency - Colindale	Thompson,C.

Segment ID	Segment	Country	Collection date	Isolate-ID	Isolate name	Originating Lab	Submitting Lab	Authors
EPI1676462	HA	United Kingdom	2019-Nov-27	EPI_ISL_408016	A/England/194900587/2019	UK Health Security Agency - Colindale	UK Health Security Agency - Colindale	Thompson,C.
EPI1676458	HA	United Kingdom	2019-Nov-26	EPI_ISL_408014	A/England/194880447/2019	UK Health Security Agency - Colindale	UK Health Security Agency - Colindale	Thompson,C.
EPI1676456	HA	United Kingdom	2019-Nov-27	EPI_ISL_408013	A/England/508/2019	UK Health Security Agency - Colindale	UK Health Security Agency - Colindale	Thompson,C.
EPI1676454	HA	United Kingdom	2019-Nov-21	EPI_ISL_408012	A/England/194840312/2019	UK Health Security Agency - Colindale	UK Health Security Agency - Colindale	Thompson,C.
EPI1676452	HA	United Kingdom	2019-Nov-22	EPI_ISL_408011	A/England/540/2019	UK Health Security Agency - Colindale	UK Health Security Agency - Colindale	Thompson,C.
EPI1674783	HA	United Kingdom	2020-Jan-04	EPI_ISL_407601	A/Northern Ireland/438/2020	Regional Virus Laboratory, Microbiology Department, Royal Victoria Hospital	Crick Worldwide Influenza Centre	
EPI1674781	HA	United Kingdom	2020-Jan-11	EPI_ISL_407600	A/Northern Ireland/1423/2020	Regional Virus Laboratory, Microbiology Department, Royal Victoria Hospital	Crick Worldwide Influenza Centre	
EPI1670620	HA	United Kingdom	2019-Nov-07	EPI_ISL_406222	A/Scotland/546/2019	Gart Naval General Hospital	Crick Worldwide Influenza Centre	
EPI1670618	HA	United Kingdom	2019-Nov-17	EPI_ISL_406221	A/Scotland/478/2019	Gart Naval General Hospital	Crick Worldwide Influenza Centre	
EPI1670616	HA	United Kingdom	2019-Nov-18	EPI_ISL_406220	A/Scotland/422/2019	Gart Naval General Hospital	Crick Worldwide Influenza Centre	
EPI1670612	HA	United Kingdom	2019-Nov-05	EPI_ISL_406218	A/Scotland/223/2019	Gart Naval General Hospital	Crick Worldwide Influenza Centre	
EPI1670610	HA	United Kingdom	2019-Nov-22	EPI_ISL_406217	A/Scotland/078/2019	Gart Naval General Hospital	Crick Worldwide Influenza Centre	
EPI1670608	HA	United Kingdom	2019-Nov-22	EPI_ISL_406216	A/Scotland/074/2019	Gart Naval General Hospital	Crick Worldwide Influenza Centre	
EPI1670606	HA	United Kingdom	2019-Sep-24	EPI_ISL_406215	A/Scotland/033/2019	Gart Naval General Hospital	Crick Worldwide Influenza Centre	

Segment ID	Segment	Country	Collection date	Isolate-ID	Isolate name	Originating Lab	Submitting Lab	Authors
EPI1660010	HA	United Kingdom	2019-Dec-03	EPI_ISL_404293	A/England/10144/2019	U.S. Air Force School of Aerospace Medicine	U.S. Air Force School of Aerospace Medicine	Gruner, W.E.; Fries, A.C.; Garrett, C.M.; Powell, M.L.; Hanson, J.F.; DeMarcus, L.S.; Sjoberg, P.A.; Bogue, A.L.; Robbins, A.S.; Macias, E.A.; Lambert, A.W.; Dane, D.J.
EPI1647236	HA	United Kingdom	2019-Dec-18	EPI_ISL_401794	A/Ystrad Mynach/9971/2019	Public Health Wales Microbiology Cardiff	Public Health Wales Microbiology Cardiff	
EPI1647228	HA	United Kingdom	2019-Dec-17	EPI_ISL_401793	A/Beaumaris/9855/2019	Public Health Wales Microbiology Cardiff	Public Health Wales Microbiology Cardiff	
EPI1647212	HA	United Kingdom	2019-Dec-16	EPI_ISL_401791	A/Ystrad Mynach/9751/2019	Public Health Wales Microbiology Cardiff	Public Health Wales Microbiology Cardiff	
EPI1647204	HA	United Kingdom	2019-Dec-11	EPI_ISL_401790	A/Beaumaris/9668/2019	Public Health Wales Microbiology Cardiff	Public Health Wales Microbiology Cardiff	
EPI1647196	HA	United Kingdom	2019-Dec-12	EPI_ISL_401789	A/Beaumaris/9667/2019	Public Health Wales Microbiology Cardiff	Public Health Wales Microbiology Cardiff	
EPI1647188	HA	United Kingdom	2019-Dec-12	EPI_ISL_401788	A/Bridgend/3347/2019	Public Health Wales Microbiology Cardiff	Public Health Wales Microbiology Cardiff	
EPI1647042	HA	United Kingdom	2019-Nov-17	EPI_ISL_401708	A/Northern Ireland/31109/2019	Regional Virus Laboratory, Microbiology Department, Royal Victoria Hospital	Crick Worldwide Influenza Centre	
EPI1647040	HA	United Kingdom	2019-Nov-17	EPI_ISL_401707	A/Northern Ireland/30990/2019	Regional Virus Laboratory, Microbiology	Crick Worldwide Influenza Centre	

Segment ID	Segment	Country	Collection date	Isolate-ID	Isolate name	Originating Lab	Submitting Lab	Authors
						Department, Royal Victoria Hospital		
EPI1647038	HA	United Kingdom	2019-Nov-16	EPI_ISL_401706	A/Northern Ireland/30977/2019	Regional Virus Laboratory, Microbiology Department, Royal Victoria Hospital	Crick Worldwide Influenza Centre	
EPI1647036	HA	United Kingdom	2019-Nov-15	EPI_ISL_401705	A/Northern Ireland/30966/2019	Regional Virus Laboratory, Microbiology Department, Royal Victoria Hospital	Crick Worldwide Influenza Centre	
EPI1647034	HA	United Kingdom	2019-Nov-15	EPI_ISL_401704	A/Northern Ireland/30965/2019	Regional Virus Laboratory, Microbiology Department, Royal Victoria Hospital	Crick Worldwide Influenza Centre	
EPI1647032	HA	United Kingdom	2019-Nov-16	EPI_ISL_401703	A/Northern Ireland/30941/2019	Regional Virus Laboratory, Microbiology Department, Royal Victoria Hospital	Crick Worldwide Influenza Centre	
EPI1647030	HA	United Kingdom	2019-Nov-13	EPI_ISL_401702	A/Northern Ireland/30814/2019	Regional Virus Laboratory, Microbiology Department, Royal Victoria Hospital	Crick Worldwide Influenza Centre	
EPI1647028	HA	United Kingdom	2019-Nov-13	EPI_ISL_401701	A/Northern Ireland/30714/2019	Regional Virus Laboratory, Microbiology Department, Royal Victoria Hospital	Crick Worldwide Influenza Centre	
EPI1647026	HA	United Kingdom	2019-Nov-13	EPI_ISL_401700	A/Northern Ireland/30703/2019	Regional Virus Laboratory, Microbiology Department, Royal Victoria Hospital	Crick Worldwide Influenza Centre	
EPI1647024	HA	United Kingdom	2019-Nov-13	EPI_ISL_401699	A/Northern Ireland/30622/2019	Regional Virus Laboratory,	Crick Worldwide Influenza Centre	

Segment ID	Segment	Country	Collection date	Isolate-ID	Isolate name	Originating Lab	Submitting Lab	Authors
						Microbiology Department, Royal Victoria Hospital		
EPI1647022	HA	United Kingdom	2019-Nov-12	EPI_ISL_401698	A/Northern Ireland/30584/2019	Regional Virus Laboratory, Microbiology Department, Royal Victoria Hospital	Crick Worldwide Influenza Centre	
EPI1647020	HA	United Kingdom	2019-Nov-12	EPI_ISL_401697	A/Northern Ireland/30522/2019	Regional Virus Laboratory, Microbiology Department, Royal Victoria Hospital	Crick Worldwide Influenza Centre	
EPI1647018	HA	United Kingdom	2019-Nov-11	EPI_ISL_401696	A/Northern Ireland/30510/2019	Regional Virus Laboratory, Microbiology Department, Royal Victoria Hospital	Crick Worldwide Influenza Centre	
EPI1647016	HA	United Kingdom	2019-Nov-08	EPI_ISL_401695	A/Northern Ireland/30346/2019	Regional Virus Laboratory, Microbiology Department, Royal Victoria Hospital	Crick Worldwide Influenza Centre	
EPI1647014	HA	United Kingdom	2019-Nov-09	EPI_ISL_401694	A/Northern Ireland/30331/2019	Regional Virus Laboratory, Microbiology Department, Royal Victoria Hospital	Crick Worldwide Influenza Centre	
EPI1647012	HA	United Kingdom	2019-Nov-09	EPI_ISL_401693	A/Northern Ireland/30295/2019	Regional Virus Laboratory, Microbiology Department, Royal Victoria Hospital	Crick Worldwide Influenza Centre	
EPI1647010	HA	United Kingdom	2019-Nov-09	EPI_ISL_401692	A/Northern Ireland/30294/2019	Regional Virus Laboratory, Microbiology Department, Royal Victoria Hospital	Crick Worldwide Influenza Centre	

Segment ID	Segment	Country	Collection date	Isolate-ID	Isolate name	Originating Lab	Submitting Lab	Authors
EPI1647008	HA	United Kingdom	2019-Nov-07	EPI_ISL_401691	A/Northern Ireland/30098/2019	Regional Virus Laboratory, Microbiology Department, Royal Victoria Hospital	Crick Worldwide Influenza Centre	
EPI1647006	HA	United Kingdom	2019-Nov-05	EPI_ISL_401690	A/Northern Ireland/30012/2019	Regional Virus Laboratory, Microbiology Department, Royal Victoria Hospital	Crick Worldwide Influenza Centre	
EPI1647004	HA	United Kingdom	2019-Nov-06	EPI_ISL_401689	A/Northern Ireland/29990/2019	Regional Virus Laboratory, Microbiology Department, Royal Victoria Hospital	Crick Worldwide Influenza Centre	
EPI1647002	HA	United Kingdom	2019-Nov-05	EPI_ISL_401688	A/Northern Ireland/29901/2019	Regional Virus Laboratory, Microbiology Department, Royal Victoria Hospital	Crick Worldwide Influenza Centre	
EPI1647000	HA	United Kingdom	2019-Nov-03	EPI_ISL_401687	A/Northern Ireland/29790/2019	Regional Virus Laboratory, Microbiology Department, Royal Victoria Hospital	Crick Worldwide Influenza Centre	
EPI1646998	HA	United Kingdom	2019-Nov-04	EPI_ISL_401686	A/Northern Ireland/29789/2019	Regional Virus Laboratory, Microbiology Department, Royal Victoria Hospital	Crick Worldwide Influenza Centre	
EPI1646996	HA	United Kingdom	2019-Nov-04	EPI_ISL_401685	A/Northern Ireland/29702/2019	Regional Virus Laboratory, Microbiology Department, Royal Victoria Hospital	Crick Worldwide Influenza Centre	
EPI1646994	HA	United Kingdom	2019-Nov-03	EPI_ISL_401684	A/Northern Ireland/29496/2019	Regional Virus Laboratory, Microbiology	Crick Worldwide Influenza Centre	

Segment ID	Segment	Country	Collection date	Isolate-ID	Isolate name	Originating Lab	Submitting Lab	Authors
						Department, Royal Victoria Hospital		
EPI1646992	HA	United Kingdom	2019-Oct-23	EPI_ISL_401683	A/Northern Ireland/29475/2019	Regional Virus Laboratory, Microbiology Department, Royal Victoria Hospital	Crick Worldwide Influenza Centre	
EPI1646719	HA	United Kingdom	2019-Nov-03	EPI_ISL_401561	A/England/94580411/2019	UK Health Security Agency - Colindale	Crick Worldwide Influenza Centre	
EPI1646718	HA	United Kingdom	2019-Oct-25	EPI_ISL_401560	A/England/94540759/2019	UK Health Security Agency - Colindale	Crick Worldwide Influenza Centre	
EPI1646717	HA	United Kingdom	2019-Oct-25	EPI_ISL_401559	A/England/94540758/2019	UK Health Security Agency - Colindale	Crick Worldwide Influenza Centre	
EPI1646716	HA	United Kingdom	2019-Oct-24	EPI_ISL_401558	A/England/94500542/2019	UK Health Security Agency - Colindale	Crick Worldwide Influenza Centre	
EPI1646715	HA	United Kingdom	2019-Oct-25	EPI_ISL_401557	A/England/94460567/2019	UK Health Security Agency - Colindale	Crick Worldwide Influenza Centre	
EPI1646714	HA	United Kingdom	2019-Oct-10	EPI_ISL_401556	A/England/94240408/2019	UK Health Security Agency - Colindale	Crick Worldwide Influenza Centre	
EPI1646713	HA	United Kingdom	2019-Oct-11	EPI_ISL_401555	A/England/94220533/2019	UK Health Security Agency - Colindale	Crick Worldwide Influenza Centre	
EPI1646712	HA	United Kingdom	2019-Oct-01	EPI_ISL_401554	A/England/94120567/2019	UK Health Security Agency - Colindale	Crick Worldwide Influenza Centre	
EPI1646711	HA	United Kingdom	2019-Nov-11	EPI_ISL_401553	A/England/350/2019	UK Health Security Agency - Colindale	Crick Worldwide Influenza Centre	
EPI1646710	HA	United Kingdom	2019-Oct-30	EPI_ISL_401552	A/England/347/2019	UK Health Security Agency - Colindale	Crick Worldwide Influenza Centre	
EPI1646709	HA	United Kingdom	2019-Oct-21	EPI_ISL_401551	A/England/344/2019	UK Health Security Agency - Colindale	Crick Worldwide Influenza Centre	
EPI1646708	HA	United Kingdom	2019-Oct-28	EPI_ISL_401550	A/England/335/2019	UK Health Security Agency - Colindale	Crick Worldwide Influenza Centre	
EPI1646707	HA	United Kingdom	2019-Oct-25	EPI_ISL_401549	A/England/331/2019	UK Health Security Agency - Colindale	Crick Worldwide Influenza Centre	
EPI1646706	HA	United Kingdom	2019-Oct-24	EPI_ISL_401548	A/England/330/2019	UK Health Security Agency - Colindale	Crick Worldwide Influenza Centre	
EPI1646705	HA	United Kingdom	2019-Oct-16	EPI_ISL_401547	A/England/325/2019	UK Health Security Agency - Colindale	Crick Worldwide Influenza Centre	
EPI1646704	HA	United Kingdom	2019-Oct-14	EPI_ISL_401546	A/England/323/2019	UK Health Security Agency - Colindale	Crick Worldwide Influenza Centre	

Segment ID	Segment	Country	Collection date	Isolate-ID	Isolate name	Originating Lab	Submitting Lab	Authors
EPI1646703	HA	United Kingdom	2019-Oct-07	EPI_ISL_401545	A/England/320/2019	UK Health Security Agency - Colindale	Crick Worldwide Influenza Centre	
EPI1646702	HA	United Kingdom	2019-Oct-15	EPI_ISL_401544	A/England/309/2019	UK Health Security Agency - Colindale	Crick Worldwide Influenza Centre	
EPI1646701	HA	United Kingdom	2019-Oct-05	EPI_ISL_401543	A/England/307/2019	UK Health Security Agency - Colindale	Crick Worldwide Influenza Centre	
EPI1640352	HA	United Kingdom	2019-Dec-11	EPI_ISL_400617	A/Wrexham/5534/2019	Public Health Wales Microbiology Cardiff	Public Health Wales Microbiology Cardiff	
EPI1640344	HA	United Kingdom	2019-Dec-11	EPI_ISL_400616	A/Oswestry/1328/2019	Public Health Wales Microbiology Cardiff	Public Health Wales Microbiology Cardiff	
EPI1640336	HA	United Kingdom	2019-Dec-10	EPI_ISL_400615	A/Ruabon/7607/2019	Public Health Wales Microbiology Cardiff	Public Health Wales Microbiology Cardiff	
EPI1640328	HA	United Kingdom	2019-Dec-09	EPI_ISL_400614	A/Prestatyn/5947/2019	Public Health Wales Microbiology Cardiff	Public Health Wales Microbiology Cardiff	
EPI1640320	HA	United Kingdom	2019-Dec-09	EPI_ISL_400613	A/Ruabon/1386/2019	Public Health Wales Microbiology Cardiff	Public Health Wales Microbiology Cardiff	
EPI1640312	HA	United Kingdom	2019-Dec-09	EPI_ISL_400612	A/Colwyn Bay/3386/2019	Public Health Wales Microbiology Cardiff	Public Health Wales Microbiology Cardiff	
EPI1640304	HA	United Kingdom	2019-Dec-08	EPI_ISL_400611	A/Deeside/2062/2019	Public Health Wales Microbiology Cardiff	Public Health Wales Microbiology Cardiff	
EPI1640296	HA	United Kingdom	2019-Dec-08	EPI_ISL_400610	A/Aberdare/9950/2019	Public Health Wales Microbiology Cardiff	Public Health Wales Microbiology Cardiff	

Segment ID	Segment	Country	Collection date	Isolate-ID	Isolate name	Originating Lab	Submitting Lab	Authors
EPI1640280	HA	United Kingdom	2019-Dec-12	EPI_ISL_400609	A/Pencader/1133/2019	Public Health Wales Microbiology Cardiff	Public Health Wales Microbiology Cardiff	
EPI1640272	HA	United Kingdom	2019-Dec-09	EPI_ISL_400608	A/Cardiff/9474/2019	Public Health Wales Microbiology Cardiff	Public Health Wales Microbiology Cardiff	
EPI1640264	HA	United Kingdom	2019-Dec-09	EPI_ISL_400607	A/Cardiff/9451/2019	Public Health Wales Microbiology Cardiff	Public Health Wales Microbiology Cardiff	
EPI1640256	HA	United Kingdom	2019-Dec-09	EPI_ISL_400606	A/Cardiff/9449/2019	Public Health Wales Microbiology Cardiff	Public Health Wales Microbiology Cardiff	
EPI1640248	HA	United Kingdom	2019-Dec-05	EPI_ISL_400605	A/Newport/9420/2019	Public Health Wales Microbiology Cardiff	Public Health Wales Microbiology Cardiff	
EPI1640239	HA	United Kingdom	2019-Dec-04	EPI_ISL_400604	A/Caerphilly/9415/2019	Public Health Wales Microbiology Cardiff	Public Health Wales Microbiology Cardiff	
EPI1640231	HA	United Kingdom	2019-Dec-04	EPI_ISL_400603	A/Newport/9414/2019	Public Health Wales Microbiology Cardiff	Public Health Wales Microbiology Cardiff	
EPI1640223	HA	United Kingdom	2019-Dec-02	EPI_ISL_400602	A/Newport/9412/2019	Public Health Wales Microbiology Cardiff	Public Health Wales Microbiology Cardiff	
EPI1640207	HA	United Kingdom	2019-Dec-02	EPI_ISL_400600	A/Cwmbran/9410/2019	Public Health Wales Microbiology Cardiff	Public Health Wales Microbiology Cardiff	
EPI1640199	HA	United Kingdom	2019-Dec-08	EPI_ISL_400599	A/Newport/9043/2019	Public Health Wales Microbiology Cardiff	Public Health Wales	

Segment ID	Segment	Country	Collection date	Isolate-ID	Isolate name	Originating Lab	Submitting Lab	Authors
							Microbiology Cardiff	
EPI1640191	HA	United Kingdom	2019-Dec-08	EPI_ISL_400598	A/Caerleon/9037/2019	Public Health Wales Microbiology Cardiff	Public Health Wales Microbiology Cardiff	
EPI1640180	HA	United Kingdom	2019-Dec-06	EPI_ISL_400597	A/Swansea/2210/2019	Public Health Wales Microbiology Cardiff	Public Health Wales Microbiology Cardiff	
EPI1640170	HA	United Kingdom	2019-Dec-05	EPI_ISL_400596	A/Swansea/2113/2019	Public Health Wales Microbiology Cardiff	Public Health Wales Microbiology Cardiff	
EPI1640162	HA	United Kingdom	2019-Dec-05	EPI_ISL_400595	A/Swansea/2096/2019	Public Health Wales Microbiology Cardiff	Public Health Wales Microbiology Cardiff	
EPI1640152	HA	United Kingdom	2019-Dec-02	EPI_ISL_400593	A/Swansea/0494/2019	Public Health Wales Microbiology Cardiff	Public Health Wales Microbiology Cardiff	
EPI1640144	HA	United Kingdom	2019-Dec-12	EPI_ISL_400591	A/Pembroke/9162/2019	Public Health Wales Microbiology Cardiff	Public Health Wales Microbiology Cardiff	
EPI1640136	HA	United Kingdom	2019-Dec-12	EPI_ISL_400590	A/Milford Haven/9127/2019	Public Health Wales Microbiology Cardiff	Public Health Wales Microbiology Cardiff	
EPI1639768	HA	United Kingdom	2019-Dec-06	EPI_ISL_400544	A/Cardiff/0273/2019	Public Health Wales Microbiology Cardiff	Public Health Wales Microbiology Cardiff	
EPI1639760	HA	United Kingdom	2019-Dec-05	EPI_ISL_400543	A/Cardiff/1419/2019	Public Health Wales Microbiology Cardiff	Public Health Wales Microbiology Cardiff	

Segment ID	Segment	Country	Collection date	Isolate-ID	Isolate name	Originating Lab	Submitting Lab	Authors
EPI1639752	HA	United Kingdom	2019-Dec-05	EPI_ISL_400542	A/Carmarthen/6881/2019	Public Health Wales Microbiology Cardiff	Public Health Wales Microbiology Cardiff	
EPI1639738	HA	United Kingdom	2019-Dec-05	EPI_ISL_400540	A/Wrexham/8948/2019	Public Health Wales Microbiology Cardiff	Public Health Wales Microbiology Cardiff	
EPI1639730	HA	United Kingdom	2019-Dec-04	EPI_ISL_400539	A/Prestatyn/7098/2019	Public Health Wales Microbiology Cardiff	Public Health Wales Microbiology Cardiff	
EPI1639720	HA	United Kingdom	2019-Dec-04	EPI_ISL_400537	A/Holywell/6458/2019	Public Health Wales Microbiology Cardiff	Public Health Wales Microbiology Cardiff	
EPI1639712	HA	United Kingdom	2019-Dec-04	EPI_ISL_400536	A/Wrexham/1655/2019	Public Health Wales Microbiology Cardiff	Public Health Wales Microbiology Cardiff	
EPI1639704	HA	United Kingdom	2019-Dec-04	EPI_ISL_400535	A/Barry/1294/2019	Public Health Wales Microbiology Cardiff	Public Health Wales Microbiology Cardiff	
EPI1639696	HA	United Kingdom	2019-Dec-03	EPI_ISL_400534	A/Wrexham/9440/2019	Public Health Wales Microbiology Cardiff	Public Health Wales Microbiology Cardiff	
EPI1639688	HA	United Kingdom	2019-Dec-03	EPI_ISL_400533	A/Wrexham/2835/2019	Public Health Wales Microbiology Cardiff	Public Health Wales Microbiology Cardiff	
EPI1639680	HA	United Kingdom	2019-Dec-02	EPI_ISL_400532	A/Mold/1968/2019	Public Health Wales Microbiology Cardiff	Public Health Wales Microbiology Cardiff	
EPI1639672	HA	United Kingdom	2019-Dec-02	EPI_ISL_400531	A/Pwllheli/2143/2019	Public Health Wales Microbiology Cardiff	Public Health Wales	

Segment ID	Segment	Country	Collection date	Isolate-ID	Isolate name	Originating Lab	Submitting Lab	Authors
							Microbiology Cardiff	
EPI1639664	HA	United Kingdom	2019-Dec-01	EPI_ISL_400530	A/Cardiff/1286/2019	Public Health Wales Microbiology Cardiff	Public Health Wales Microbiology Cardiff	
EPI1639656	HA	United Kingdom	2019-Nov-30	EPI_ISL_400529	A/Llanfairfechan/0913/2019	Public Health Wales Microbiology Cardiff	Public Health Wales Microbiology Cardiff	
EPI1639648	HA	United Kingdom	2019-Nov-28	EPI_ISL_400528	A/Chirk/3206/2019	Public Health Wales Microbiology Cardiff	Public Health Wales Microbiology Cardiff	
EPI1639640	HA	United Kingdom	2019-Nov-26	EPI_ISL_400527	A/Brackla/3277/2019	Public Health Wales Microbiology Cardiff	Public Health Wales Microbiology Cardiff	
EPI1639632	HA	United Kingdom	2019-Dec-07	EPI_ISL_400526	A/Grangetown/9405/2019	Public Health Wales Microbiology Cardiff	Public Health Wales Microbiology Cardiff	
EPI1639623	HA	United Kingdom	2019-Dec-06	EPI_ISL_400525	A/Cardiff/9403/2019	Public Health Wales Microbiology Cardiff	Public Health Wales Microbiology Cardiff	
EPI1639615	HA	United Kingdom	2019-Dec-06	EPI_ISL_400524	A/Roath/9401/2019	Public Health Wales Microbiology Cardiff	Public Health Wales Microbiology Cardiff	
EPI1639607	HA	United Kingdom	2019-Dec-04	EPI_ISL_400523	A/Newport/9366/2019	Public Health Wales Microbiology Cardiff	Public Health Wales Microbiology Cardiff	
EPI1639599	HA	United Kingdom	2019-Dec-05	EPI_ISL_400522	A/Riverside/9365/2019	Public Health Wales Microbiology Cardiff	Public Health Wales Microbiology Cardiff	

Segment ID	Segment	Country	Collection date	Isolate-ID	Isolate name	Originating Lab	Submitting Lab	Authors
EPI1639583	HA	United Kingdom	2019-Dec-04	EPI_ISL_400520	A/Cardiff/9352/2019	Public Health Wales Microbiology Cardiff	Public Health Wales Microbiology Cardiff	
EPI1639575	HA	United Kingdom	2019-Dec-04	EPI_ISL_400519	A/Maesycwmmer/8953/2019	Public Health Wales Microbiology Cardiff	Public Health Wales Microbiology Cardiff	
EPI1639567	HA	United Kingdom	2019-Nov-27	EPI_ISL_400518	A/Gorseinon/1835/2019	Public Health Wales Microbiology Cardiff	Public Health Wales Microbiology Cardiff	
EPI1639559	HA	United Kingdom	2019-Dec-05	EPI_ISL_400517	A/Swansea/0922/2019	Public Health Wales Microbiology Cardiff	Public Health Wales Microbiology Cardiff	
EPI1639551	HA	United Kingdom	2019-Dec-04	EPI_ISL_400516	A/Swansea/0891/2019	Public Health Wales Microbiology Cardiff	Public Health Wales Microbiology Cardiff	
EPI1639543	HA	United Kingdom	2019-Dec-04	EPI_ISL_400515	A/Pontardawe/0726/2019	Public Health Wales Microbiology Cardiff	Public Health Wales Microbiology Cardiff	
EPI1639535	HA	United Kingdom	2019-Dec-04	EPI_ISL_400514	A/Swansea/0719/2019	Public Health Wales Microbiology Cardiff	Public Health Wales Microbiology Cardiff	
EPI1639527	HA	United Kingdom	2019-Dec-03	EPI_ISL_400513	A/Port Talbot/0536/2019	Public Health Wales Microbiology Cardiff	Public Health Wales Microbiology Cardiff	
EPI1639519	HA	United Kingdom	2019-Nov-04	EPI_ISL_400512	A/Swansea/7685/2019	Public Health Wales Microbiology Cardiff	Public Health Wales Microbiology Cardiff	
EPI1639505	HA	United Kingdom	2019-Nov-25	EPI_ISL_400510	A/Tywyn/0664/2019	Public Health Wales Microbiology Cardiff	Public Health Wales	

Segment ID	Segment	Country	Collection date	Isolate-ID	Isolate name	Originating Lab	Submitting Lab	Authors
							Microbiology Cardiff	
EPI1639497	HA	United Kingdom	2019-Dec-03	EPI_ISL_400509	A/Llangefni/9955/2019	Public Health Wales Microbiology Cardiff	Public Health Wales Microbiology Cardiff	
EPI1636154	HA	United Kingdom	2019-Dec-01	EPI_ISL_399971	A/Rhyl/4515/2019	Public Health Wales Microbiology Cardiff	Public Health Wales Microbiology Cardiff	
EPI1636146	HA	United Kingdom	2019-Nov-29	EPI_ISL_399970	A/Abergele/8675/2019	Public Health Wales Microbiology Cardiff	Public Health Wales Microbiology Cardiff	
EPI1636138	HA	United Kingdom	2019-Nov-28	EPI_ISL_399969	A/Wrexham/3835/2019	Public Health Wales Microbiology Cardiff	Public Health Wales Microbiology Cardiff	
EPI1636130	HA	United Kingdom	2019-Nov-29	EPI_ISL_399968	A/Cardiff/2436/2019	Public Health Wales Microbiology Cardiff	Public Health Wales Microbiology Cardiff	
EPI1636122	HA	United Kingdom	2019-Nov-30	EPI_ISL_399967	A/Cardiff/9250/2019	Public Health Wales Microbiology Cardiff	Public Health Wales Microbiology Cardiff	
EPI1636114	HA	United Kingdom	2019-Nov-30	EPI_ISL_399966	A/Cardiff/9249/2019	Public Health Wales Microbiology Cardiff	Public Health Wales Microbiology Cardiff	
EPI1636106	HA	United Kingdom	2019-Dec-01	EPI_ISL_399965	A/Cardiff/9248/2019	Public Health Wales Microbiology Cardiff	Public Health Wales Microbiology Cardiff	
EPI1636098	HA	United Kingdom	2019-Nov-29	EPI_ISL_399964	A/Cardiff/9234/2019	Public Health Wales Microbiology Cardiff	Public Health Wales Microbiology Cardiff	

Segment ID	Segment	Country	Collection date	Isolate-ID	Isolate name	Originating Lab	Submitting Lab	Authors
EPI1636090	HA	United Kingdom	2019-Nov-29	EPI_ISL_399963	A/Cardiff/9206/2019	Public Health Wales Microbiology Cardiff	Public Health Wales Microbiology Cardiff	
EPI1636082	HA	United Kingdom	2019-Nov-23	EPI_ISL_399962	A/Monmouth/9165/2019	Public Health Wales Microbiology Cardiff	Public Health Wales Microbiology Cardiff	
EPI1636074	HA	United Kingdom	2019-Nov-27	EPI_ISL_399961	A/Barry/9160/2019	Public Health Wales Microbiology Cardiff	Public Health Wales Microbiology Cardiff	
EPI1636050	HA	United Kingdom	2019-Nov-27	EPI_ISL_399958	A/Swansea/1853/2019	Public Health Wales Microbiology Cardiff	Public Health Wales Microbiology Cardiff	
EPI1636041	HA	United Kingdom	2019-Nov-27	EPI_ISL_399957	A/Swansea/1834/2019	Public Health Wales Microbiology Cardiff	Public Health Wales Microbiology Cardiff	
EPI1636026	HA	United Kingdom	2019-Nov-23	EPI_ISL_399955	A/Swansea/1326/2019	Public Health Wales Microbiology Cardiff	Public Health Wales Microbiology Cardiff	
EPI1636018	HA	United Kingdom	2019-Nov-22	EPI_ISL_399954	A/Britton Ferry/1325/2019	Public Health Wales Microbiology Cardiff	Public Health Wales Microbiology Cardiff	
EPI1636010	HA	United Kingdom	2019-Nov-23	EPI_ISL_399953	A/Maesteg/1131/2019	Public Health Wales Microbiology Cardiff	Public Health Wales Microbiology Cardiff	
EPI1636002	HA	United Kingdom	2019-Dec-01	EPI_ISL_399952	A/Swansea/0364/2019	Public Health Wales Microbiology Cardiff	Public Health Wales Microbiology Cardiff	
EPI1635994	HA	United Kingdom	2019-Nov-28	EPI_ISL_399951	A/Neath/0125/2019	Public Health Wales Microbiology Cardiff	Public Health Wales	

Segment ID	Segment	Country	Collection date	Isolate-ID	Isolate name	Originating Lab	Submitting Lab	Authors
							Microbiology Cardiff	
EPI1635986	HA	United Kingdom	2019-Nov-27	EPI_ISL_399950	A/Rhyl/9543/2019	Public Health Wales Microbiology Cardiff	Public Health Wales Microbiology Cardiff	
EPI1635725	HA	United Kingdom	2019-Oct-30	EPI_ISL_399844	A/England/347/2019	UK Health Security Agency - Colindale	UK Health Security Agency - Colindale	Thompson,C.
EPI1635717	HA	United Kingdom	2019-Nov-01	EPI_ISL_399843	A/England/194520480/2019	UK Health Security Agency - Colindale	UK Health Security Agency - Colindale	Thompson,C.
EPI1635709	HA	United Kingdom	2019-Oct-24	EPI_ISL_399842	A/England/194500542/2019	UK Health Security Agency - Colindale	UK Health Security Agency - Colindale	Thompson,C.
EPI1635701	HA	United Kingdom	2019-Oct-28	EPI_ISL_399841	A/England/335/2019	UK Health Security Agency - Colindale	UK Health Security Agency - Colindale	Thompson,C.
EPI1635693	HA	United Kingdom	2019-Oct-25	EPI_ISL_399840	A/England/194460567/2019	UK Health Security Agency - Colindale	UK Health Security Agency - Colindale	Thompson,C.
EPI1635685	HA	United Kingdom	2019-Oct-24	EPI_ISL_399839	A/England/194460566/2019	UK Health Security Agency - Colindale	UK Health Security Agency - Colindale	Thompson,C.
EPI1635677	HA	United Kingdom	2019-Oct-23	EPI_ISL_399838	A/England/194440722/2019	UK Health Security Agency - Colindale	UK Health Security Agency - Colindale	Thompson,C.
EPI1635669	HA	United Kingdom	2019-Oct-25	EPI_ISL_399837	A/England/331/2019	UK Health Security Agency - Colindale	UK Health Security Agency - Colindale	Thompson,C.
EPI1635661	HA	United Kingdom	2019-Oct-24	EPI_ISL_399836	A/England/330/2019	UK Health Security Agency - Colindale	UK Health Security Agency - Colindale	Thompson,C.
EPI1635653	HA	United Kingdom	2019-Oct-24	EPI_ISL_399835	A/England/346/2019	UK Health Security Agency - Colindale	UK Health Security Agency - Colindale	Thompson,C.
EPI1635645	HA	United Kingdom	2019-Oct-23	EPI_ISL_399834	A/England/356/2019	UK Health Security Agency - Colindale	UK Health Security Agency - Colindale	Thompson,C.

Segment ID	Segment	Country	Collection date	Isolate-ID	Isolate name	Originating Lab	Submitting Lab	Authors
EPI1635637	HA	United Kingdom	2019-Oct-20	EPI_ISL_399833	A/England/328/2019	UK Health Security Agency - Colindale	UK Health Security Agency - Colindale	Thompson,C.
EPI1635629	HA	United Kingdom	2019-Oct-18	EPI_ISL_399832	A/England/345/2019	UK Health Security Agency - Colindale	UK Health Security Agency - Colindale	Thompson,C.
EPI1635621	HA	United Kingdom	2019-Oct-21	EPI_ISL_399831	A/England/344/2019	UK Health Security Agency - Colindale	UK Health Security Agency - Colindale	Thompson,C.
EPI1635613	HA	United Kingdom	2019-Oct-21	EPI_ISL_399830	A/England/194380622/2019	UK Health Security Agency - Colindale	UK Health Security Agency - Colindale	Thompson,C.
EPI1635605	HA	United Kingdom	2019-Oct-21	EPI_ISL_399829	A/England/343/2019	UK Health Security Agency - Colindale	UK Health Security Agency - Colindale	Thompson,C.
EPI1635597	HA	United Kingdom	2019-Oct-22	EPI_ISL_399828	A/England/327/2019	UK Health Security Agency - Colindale	UK Health Security Agency - Colindale	Thompson,C.
EPI1635589	HA	United Kingdom	2019-Oct-17	EPI_ISL_399827	A/England/326/2019	UK Health Security Agency - Colindale	UK Health Security Agency - Colindale	Thompson,C.
EPI1635581	HA	United Kingdom	2019-Oct-16	EPI_ISL_399826	A/England/342/2019	UK Health Security Agency - Colindale	UK Health Security Agency - Colindale	Thompson,C.
EPI1635573	HA	United Kingdom	2019-Oct-16	EPI_ISL_399825	A/England/325/2019	UK Health Security Agency - Colindale	UK Health Security Agency - Colindale	Thompson,C.
EPI1635557	HA	United Kingdom	2019-Oct-16	EPI_ISL_399823	A/England/324/2019	UK Health Security Agency - Colindale	UK Health Security Agency - Colindale	Thompson,C.
EPI1635549	HA	United Kingdom	2019-Oct-14	EPI_ISL_399822	A/England/323/2019	UK Health Security Agency - Colindale	UK Health Security Agency - Colindale	Thompson,C.
EPI1635541	HA	United Kingdom	2019-Oct-15	EPI_ISL_399821	A/England/322/2019	UK Health Security Agency - Colindale	UK Health Security Agency - Colindale	Thompson,C.
EPI1635533	HA	United Kingdom	2019-Oct-16	EPI_ISL_399820	A/England/321/2019	UK Health Security Agency - Colindale	UK Health Security Agency - Colindale	Thompson,C.

Segment ID	Segment	Country	Collection date	Isolate-ID	Isolate name	Originating Lab	Submitting Lab	Authors
EPI1635517	HA	United Kingdom	2019-Oct-15	EPI_ISL_399818	A/England/309/2019	UK Health Security Agency - Colindale	UK Health Security Agency - Colindale	Thompson,C.
EPI1635509	HA	United Kingdom	2019-Oct-12	EPI_ISL_399817	A/England/194260576/2019	UK Health Security Agency - Colindale	UK Health Security Agency - Colindale	Thompson,C.
EPI1635493	HA	United Kingdom	2019-Oct-10	EPI_ISL_399815	A/England/194240408/2019	UK Health Security Agency - Colindale	UK Health Security Agency - Colindale	Thompson,C.
EPI1635485	HA	United Kingdom	2019-Oct-09	EPI_ISL_399814	A/England/308/2019	UK Health Security Agency - Colindale	UK Health Security Agency - Colindale	Thompson,C.
EPI1635477	HA	United Kingdom	2019-Oct-11	EPI_ISL_399813	A/England/194220533/2019	UK Health Security Agency - Colindale	UK Health Security Agency - Colindale	Thompson,C.
EPI1635469	HA	United Kingdom	2019-Oct-09	EPI_ISL_399812	A/England/194220531/2019	UK Health Security Agency - Colindale	UK Health Security Agency - Colindale	Thompson,C.
EPI1635461	HA	United Kingdom	2019-Oct-05	EPI_ISL_399811	A/England/307/2019	UK Health Security Agency - Colindale	UK Health Security Agency - Colindale	Thompson,C.
EPI1635453	HA	United Kingdom	2019-Oct-07	EPI_ISL_399810	A/England/320/2019	UK Health Security Agency - Colindale	UK Health Security Agency - Colindale	Thompson,C.
EPI1635445	HA	United Kingdom	2019-Oct-06	EPI_ISL_399809	A/England/306/2019	UK Health Security Agency - Colindale	UK Health Security Agency - Colindale	Thompson,C.
EPI1635429	HA	United Kingdom	2019-Oct-01	EPI_ISL_399807	A/England/194120567/2019	UK Health Security Agency - Colindale	UK Health Security Agency - Colindale	Thompson,C.
EPI1635405	HA	United Kingdom	2019-Sep-21	EPI_ISL_399804	A/England/318/2019	UK Health Security Agency - Colindale	UK Health Security Agency - Colindale	Thompson,C.
EPI1635285	HA	United Kingdom	2019-Jun-27	EPI_ISL_399789	A/England/92700431/2019	UK Health Security Agency - Colindale	UK Health Security Agency - Colindale	Thompson,C.
EPI1635277	HA	United Kingdom	2019-Jun-25	EPI_ISL_399788	A/England/92620632/2019	UK Health Security Agency - Colindale	UK Health Security Agency - Colindale	Thompson,C.

Segment ID	Segment	Country	Collection date	Isolate-ID	Isolate name	Originating Lab	Submitting Lab	Authors
EPI1635205	HA	United Kingdom	2019-Oct-24	EPI_ISL_399779	A/England/194500540/2019	UK Health Security Agency - Colindale	UK Health Security Agency - Colindale	Thompson,C.
EPI1635203	HA	United Kingdom	2019-Oct-25	EPI_ISL_399778	A/England/194440720/2019	UK Health Security Agency - Colindale	UK Health Security Agency - Colindale	Thompson,C.
EPI1635201	HA	United Kingdom	2019-Sep-27	EPI_ISL_399777	A/England/319/2019	UK Health Security Agency - Colindale	UK Health Security Agency - Colindale	Thompson,C.
EPI1635189	HA	United Kingdom	2019-Jul-20	EPI_ISL_399770	A/England/93040538/2019	UK Health Security Agency - Colindale	UK Health Security Agency - Colindale	Thompson,C.
EPI1629925	HA	United Kingdom	2019-Nov-19	EPI_ISL_398669	A/Swansea/8688/2019	Public Health Wales Microbiology Cardiff	Public Health Wales Microbiology Cardiff	
EPI1629917	HA	United Kingdom	2019-Nov-19	EPI_ISL_398668	A/Aberdare/6417/2019	Public Health Wales Microbiology Cardiff	Public Health Wales Microbiology Cardiff	
EPI1629909	HA	United Kingdom	2019-Nov-11	EPI_ISL_398667	A/Wrexham/5048/2019	Public Health Wales Microbiology Cardiff	Public Health Wales Microbiology Cardiff	
EPI1629901	HA	United Kingdom	2019-Nov-08	EPI_ISL_398666	A/Wrexham/0716/2019	Public Health Wales Microbiology Cardiff	Public Health Wales Microbiology Cardiff	
EPI1629893	HA	United Kingdom	2019-Nov-18	EPI_ISL_398665	A/Swansea/9601/2019	Public Health Wales Microbiology Cardiff	Public Health Wales Microbiology Cardiff	
EPI1629884	HA	United Kingdom	2019-Nov-15	EPI_ISL_398664	A/Swansea/9435/2019	Public Health Wales Microbiology Cardiff	Public Health Wales Microbiology Cardiff	
EPI1629875	HA	United Kingdom	2019-Nov-17	EPI_ISL_398663	A/Swansea/9423/2019	Public Health Wales Microbiology Cardiff	Public Health Wales	

Segment ID	Segment	Country	Collection date	Isolate-ID	Isolate name	Originating Lab	Submitting Lab	Authors
							Microbiology Cardiff	
EPI1626595	HA	United Kingdom	2019-Oct-30	EPI_ISL_398176	A/Monmouth/3635/2019	Public Health Wales Microbiology Cardiff	Public Health Wales Microbiology Cardiff	
EPI1626587	HA	United Kingdom	2019-Oct-27	EPI_ISL_398175	A/Cardiff/3570/2019	Public Health Wales Microbiology Cardiff	Public Health Wales Microbiology Cardiff	
EPI1626579	HA	United Kingdom	2019-Oct-18	EPI_ISL_398174	A/Cwmbran/4188/2019	Public Health Wales Microbiology Cardiff	Public Health Wales Microbiology Cardiff	
EPI1584154	HA	United Kingdom	2019-Aug-09	EPI_ISL_389331	A/Porthcawl/7839/2019	Public Health Wales Microbiology Cardiff	Public Health Wales Microbiology Cardiff	
EPI1584146	HA	United Kingdom	2019-Aug-06	EPI_ISL_389330	A/Neath/4294/2019	Public Health Wales Microbiology Cardiff	Public Health Wales Microbiology Cardiff	
EPI1584138	HA	United Kingdom	2019-Jul-25	EPI_ISL_389329	A/Wrexham/3245/2019	Public Health Wales Microbiology Cardiff	Public Health Wales Microbiology Cardiff	
EPI1584130	HA	United Kingdom	2019-Aug-14	EPI_ISL_389328	A/Cardiff/2575/2019	Public Health Wales Microbiology Cardiff	Public Health Wales Microbiology Cardiff	
EPI1584122	HA	United Kingdom	2019-Jul-30	EPI_ISL_389327	A/Port Talbot/2404/2019	Public Health Wales Microbiology Cardiff	Public Health Wales Microbiology Cardiff	
EPI1584114	HA	United Kingdom	2019-Jul-25	EPI_ISL_389326	A/Lima/2360/2019	Public Health Wales Microbiology Cardiff	Public Health Wales Microbiology Cardiff	

Segment ID	Segment	Country	Collection date	Isolate-ID	Isolate name	Originating Lab	Submitting Lab	Authors
EPI1584107	HA	United Kingdom	2019-Jun-12	EPI_ISL_389325	A/Cardiff/9746/2019	Public Health Wales Microbiology Cardiff	Public Health Wales Microbiology Cardiff	
EPI1584099	HA	United Kingdom	2019-Jun-02	EPI_ISL_389324	A/Cardiff/9558/2019	Public Health Wales Microbiology Cardiff	Public Health Wales Microbiology Cardiff	
EPI1584083	HA	United Kingdom	2019-Aug-04	EPI_ISL_389322	A/Port Talbot/4679/2019	Public Health Wales Microbiology Cardiff	Public Health Wales Microbiology Cardiff	
EPI1963694	HA	United Kingdom	2020-Mar-03	EPI_ISL_9029178	A/England/201141142/2020	UK Health Security Agency - Colindale	UK Health Security Agency - Colindale	UKHSA, Respiratory Virus Unit
EPI1963686	HA	United Kingdom	2020-Mar-02	EPI_ISL_9029177	A/England/201141141/2020	UK Health Security Agency - Colindale	UK Health Security Agency - Colindale	UKHSA, Respiratory Virus Unit
EPI1963678	HA	United Kingdom	2020-Mar-05	EPI_ISL_9029176	A/England/201141139/2020	UK Health Security Agency - Colindale	UK Health Security Agency - Colindale	UKHSA, Respiratory Virus Unit
EPI1963670	HA	United Kingdom	2020-Mar-01	EPI_ISL_9029173	A/England/201141138/2020	UK Health Security Agency - Colindale	UK Health Security Agency - Colindale	UKHSA, Respiratory Virus Unit
EPI1963662	HA	United Kingdom	2020-Feb-28	EPI_ISL_9029169	A/England/201141136/2020	UK Health Security Agency - Colindale	UK Health Security Agency - Colindale	UKHSA, Respiratory Virus Unit
EPI1963598	HA	United Kingdom	2020-Mar-09	EPI_ISL_9029144	A/England/201100892/2020	UK Health Security Agency - Colindale	UK Health Security Agency - Colindale	UKHSA, Respiratory Virus Unit
EPI1963502	HA	United Kingdom	2020-Mar-02	EPI_ISL_9029123	A/England/201041548/2020	UK Health Security Agency - Colindale	UK Health Security Agency - Colindale	UKHSA, Respiratory Virus Unit
EPI1755011	HA	United Kingdom	2020-Feb-19	EPI_ISL_487084	A/England/200840504/2020	UK Health Security Agency - Colindale	UK Health Security Agency - Colindale	
EPI1755003	HA	United Kingdom	2020-Feb-14	EPI_ISL_487083	A/England/200840503/2020	UK Health Security Agency - Colindale	UK Health Security Agency - Colindale	

Segment ID	Segment	Country	Collection date	Isolate-ID	Isolate name	Originating Lab	Submitting Lab	Authors
EPI1754995	HA	United Kingdom	2020-Feb-10	EPI_ISL_487082	A/England/200840502/2020	UK Health Security Agency - Colindale	UK Health Security Agency - Colindale	
EPI1754987	HA	United Kingdom	2020-Feb-13	EPI_ISL_487081	A/England/200840481/2020	UK Health Security Agency - Colindale	UK Health Security Agency - Colindale	
EPI1754979	HA	United Kingdom	2020-Feb-18	EPI_ISL_487080	A/England/200820423/2020	UK Health Security Agency - Colindale	UK Health Security Agency - Colindale	
EPI1754971	HA	United Kingdom	2020-Feb-18	EPI_ISL_487079	A/England/200820422/2020	UK Health Security Agency - Colindale	UK Health Security Agency - Colindale	
EPI1754963	HA	United Kingdom	2020-Feb-13	EPI_ISL_487078	A/England/200820421/2020	UK Health Security Agency - Colindale	UK Health Security Agency - Colindale	
EPI1754955	HA	United Kingdom	2020-Feb-18	EPI_ISL_487077	A/England/200820420/2020	UK Health Security Agency - Colindale	UK Health Security Agency - Colindale	
EPI1754947	HA	United Kingdom	2020-Feb-13	EPI_ISL_487076	A/England/200820419/2020	UK Health Security Agency - Colindale	UK Health Security Agency - Colindale	
EPI1754939	HA	United Kingdom	2020-Feb-13	EPI_ISL_487075	A/England/200820418/2020	UK Health Security Agency - Colindale	UK Health Security Agency - Colindale	
EPI1754931	HA	United Kingdom	2020-Feb-18	EPI_ISL_487074	A/England/200820417/2020	UK Health Security Agency - Colindale	UK Health Security Agency - Colindale	
EPI1754923	HA	United Kingdom	2020-Feb-13	EPI_ISL_487073	A/England/200820416/2020	UK Health Security Agency - Colindale	UK Health Security Agency - Colindale	
EPI1754891	HA	United Kingdom	2020-Jan-31	EPI_ISL_487069	A/England/102/2020	UK Health Security Agency - Colindale	UK Health Security Agency - Colindale	
EPI1754875	HA	United Kingdom	2020-Jan-31	EPI_ISL_487067	A/England/200660467/2020	UK Health Security Agency - Colindale	UK Health Security Agency - Colindale	
EPI1754859	HA	United Kingdom	2020-Feb-03	EPI_ISL_487065	A/England/200640430/2020	UK Health Security Agency - Colindale	UK Health Security Agency - Colindale	

Segment ID	Segment	Country	Collection date	Isolate-ID	Isolate name	Originating Lab	Submitting Lab	Authors
EPI1754851	HA	United Kingdom	2020-Feb-01	EPI_ISL_487064	A/England/101/2020	UK Health Security Agency - Colindale	UK Health Security Agency - Colindale	
EPI1754785	HA	United Kingdom	2020-Jan-30	EPI_ISL_487058	A/England/200580698/2020	UK Health Security Agency - Colindale	UK Health Security Agency - Colindale	
EPI1754777	HA	United Kingdom	2020-Jan-29	EPI_ISL_487057	A/England/99/2020	UK Health Security Agency - Colindale	UK Health Security Agency - Colindale	
EPI1754761	HA	United Kingdom	2019-Dec-27	EPI_ISL_487055	A/England/200181207/2019	UK Health Security Agency - Colindale	UK Health Security Agency - Colindale	
EPI1754753	HA	United Kingdom	2019-Dec-26	EPI_ISL_487054	A/England/200181205/2019	UK Health Security Agency - Colindale	UK Health Security Agency - Colindale	
EPI1754745	HA	United Kingdom	2019-Dec-18	EPI_ISL_487053	A/England/200181200/2019	UK Health Security Agency - Colindale	UK Health Security Agency - Colindale	
EPI1754737	HA	United Kingdom	2019-Dec-18	EPI_ISL_487052	A/England/200181199/2019	UK Health Security Agency - Colindale	UK Health Security Agency - Colindale	
EPI1754729	HA	United Kingdom	2019-Dec-18	EPI_ISL_487051	A/England/200181195/2019	UK Health Security Agency - Colindale	UK Health Security Agency - Colindale	
EPI1754721	HA	United Kingdom	2020-Jan-03	EPI_ISL_487050	A/England/200181194/2020	UK Health Security Agency - Colindale	UK Health Security Agency - Colindale	
EPI1754713	HA	United Kingdom	2020-Jan-03	EPI_ISL_487049	A/England/200181193/2020	UK Health Security Agency - Colindale	UK Health Security Agency - Colindale	
EPI1754705	HA	United Kingdom	2019-Dec-18	EPI_ISL_487048	A/England/200181192/2019	UK Health Security Agency - Colindale	UK Health Security Agency - Colindale	
EPI1754697	HA	United Kingdom	2019-Dec-19	EPI_ISL_487047	A/England/200181191/2019	UK Health Security Agency - Colindale	UK Health Security Agency - Colindale	
EPI1754689	HA	United Kingdom	2019-Dec-27	EPI_ISL_487046	A/England/200181189/2019	UK Health Security Agency - Colindale	UK Health Security Agency - Colindale	

Segment ID	Segment	Country	Collection date	Isolate-ID	Isolate name	Originating Lab	Submitting Lab	Authors
EPI1754681	HA	United Kingdom	2020-Jan-03	EPI_ISL_487045	A/England/48/2020	UK Health Security Agency - Colindale	UK Health Security Agency - Colindale	
EPI1754673	HA	United Kingdom	2019-Dec-27	EPI_ISL_487044	A/England/200181187/2019	UK Health Security Agency - Colindale	UK Health Security Agency - Colindale	
EPI1754665	HA	United Kingdom	2019-Dec-27	EPI_ISL_487043	A/England/200181186/2019	UK Health Security Agency - Colindale	UK Health Security Agency - Colindale	
EPI1754657	HA	United Kingdom	2019-Dec-27	EPI_ISL_487042	A/England/200181184/2019	UK Health Security Agency - Colindale	UK Health Security Agency - Colindale	
EPI1754649	HA	United Kingdom	2019-Dec-28	EPI_ISL_487041	A/England/200181183/2019	UK Health Security Agency - Colindale	UK Health Security Agency - Colindale	
EPI1754633	HA	United Kingdom	2019-Dec-30	EPI_ISL_487039	A/England/903/2019	UK Health Security Agency - Colindale	UK Health Security Agency - Colindale	
EPI1754625	HA	United Kingdom	2019-Dec-24	EPI_ISL_487038	A/England/200181149/2019	UK Health Security Agency - Colindale	UK Health Security Agency - Colindale	
EPI1754617	HA	United Kingdom	2019-Dec-23	EPI_ISL_487037	A/England/902/2019	UK Health Security Agency - Colindale	UK Health Security Agency - Colindale	
EPI1754609	HA	United Kingdom	2019-Dec-24	EPI_ISL_487036	A/England/200181140/2019	UK Health Security Agency - Colindale	UK Health Security Agency - Colindale	
EPI1754601	HA	United Kingdom	2019-Dec-24	EPI_ISL_487035	A/England/200181139/2019	UK Health Security Agency - Colindale	UK Health Security Agency - Colindale	
EPI1754593	HA	United Kingdom	2019-Dec-24	EPI_ISL_487034	A/England/200181137/2019	UK Health Security Agency - Colindale	UK Health Security Agency - Colindale	
EPI1754585	HA	United Kingdom	2019-Dec-23	EPI_ISL_487033	A/England/200181136/2019	UK Health Security Agency - Colindale	UK Health Security Agency - Colindale	
EPI1754577	HA	United Kingdom	2019-Dec-23	EPI_ISL_487032	A/England/200181135/2019	UK Health Security Agency - Colindale	UK Health Security Agency - Colindale	

Segment ID	Segment	Country	Collection date	Isolate-ID	Isolate name	Originating Lab	Submitting Lab	Authors
EPI1754569	HA	United Kingdom	2019-Dec-23	EPI_ISL_487031	A/England/200181133/2019	UK Health Security Agency - Colindale	UK Health Security Agency - Colindale	
EPI1754561	HA	United Kingdom	2019-Dec-23	EPI_ISL_487030	A/England/200181132/2019	UK Health Security Agency - Colindale	UK Health Security Agency - Colindale	
EPI1754553	HA	United Kingdom	2019-Dec-23	EPI_ISL_487029	A/England/200181131/2019	UK Health Security Agency - Colindale	UK Health Security Agency - Colindale	
EPI1754545	HA	United Kingdom	2019-Dec-24	EPI_ISL_487028	A/England/200181130/2019	UK Health Security Agency - Colindale	UK Health Security Agency - Colindale	
EPI1754537	HA	United Kingdom	2019-Dec-24	EPI_ISL_487027	A/England/926/2019	UK Health Security Agency - Colindale	UK Health Security Agency - Colindale	
EPI1754529	HA	United Kingdom	2019-Dec-25	EPI_ISL_487026	A/England/200120951/2019	UK Health Security Agency - Colindale	UK Health Security Agency - Colindale	
EPI1754521	HA	United Kingdom	2019-Dec-24	EPI_ISL_487025	A/England/200120950/2019	UK Health Security Agency - Colindale	UK Health Security Agency - Colindale	
EPI1754513	HA	United Kingdom	2019-Dec-25	EPI_ISL_487024	A/England/200120949/2019	UK Health Security Agency - Colindale	UK Health Security Agency - Colindale	
EPI1754497	HA	United Kingdom	2019-Dec-25	EPI_ISL_487022	A/England/200120929/2019	UK Health Security Agency - Colindale	UK Health Security Agency - Colindale	
EPI1754481	HA	United Kingdom	2019-Dec-15	EPI_ISL_487020	A/England/200120925/2019	UK Health Security Agency - Colindale	UK Health Security Agency - Colindale	
EPI1754473	HA	United Kingdom	2019-Dec-15	EPI_ISL_487019	A/England/200120924/2019	UK Health Security Agency - Colindale	UK Health Security Agency - Colindale	
EPI1754465	HA	United Kingdom	2019-Dec-17	EPI_ISL_487018	A/England/890/2019	UK Health Security Agency - Colindale	UK Health Security Agency - Colindale	
EPI1754457	HA	United Kingdom	2019-Dec-17	EPI_ISL_487017	A/England/889/2019	UK Health Security Agency - Colindale	UK Health Security Agency - Colindale	

Segment ID	Segment	Country	Collection date	Isolate-ID	Isolate name	Originating Lab	Submitting Lab	Authors
EPI1754449	HA	United Kingdom	2019-Dec-22	EPI_ISL_487016	A/England/878/2019	UK Health Security Agency - Colindale	UK Health Security Agency - Colindale	
EPI1754441	HA	United Kingdom	2019-Dec-21	EPI_ISL_487015	A/England/877/2019	UK Health Security Agency - Colindale	UK Health Security Agency - Colindale	
EPI1754433	HA	United Kingdom	2019-Dec-18	EPI_ISL_487014	A/England/863/2019	UK Health Security Agency - Colindale	UK Health Security Agency - Colindale	
EPI1754425	HA	United Kingdom	2019-Dec-18	EPI_ISL_487013	A/England/858/2019	UK Health Security Agency - Colindale	UK Health Security Agency - Colindale	
EPI1754417	HA	United Kingdom	2019-Dec-22	EPI_ISL_487012	A/England/857/2019	UK Health Security Agency - Colindale	UK Health Security Agency - Colindale	
EPI1754409	HA	United Kingdom	2019-Dec-12	EPI_ISL_487011	A/England/820/2019	UK Health Security Agency - Colindale	UK Health Security Agency - Colindale	
EPI1754382	HA	United Kingdom	2020-Jan-03	EPI_ISL_487002	A/England/200181204/2020	UK Health Security Agency - Colindale	UK Health Security Agency - Colindale	
EPI1754380	HA	United Kingdom	2019-Dec-27	EPI_ISL_487001	A/England/200181185/2019	UK Health Security Agency - Colindale	UK Health Security Agency - Colindale	
EPI1754370	HA	United Kingdom	2019-Dec-23	EPI_ISL_486996	A/England/200181128/2019	UK Health Security Agency - Colindale	UK Health Security Agency - Colindale	
EPI1747004	HA	United Kingdom	2020-Jan-04	EPI_ISL_471039	A/England/70/2020	UK Health Security Agency - Colindale	UK Health Security Agency - Colindale	
EPI1746988	HA	United Kingdom	2020-Jan-01	EPI_ISL_471031	A/England/69/2020	UK Health Security Agency - Colindale	UK Health Security Agency - Colindale	
EPI1746980	HA	United Kingdom	2019-Dec-30	EPI_ISL_471027	A/England/927/2019	UK Health Security Agency - Colindale	UK Health Security Agency - Colindale	
EPI1746972	HA	United Kingdom	2020-Jan-01	EPI_ISL_471026	A/England/200240790/2020	UK Health Security Agency - Colindale	UK Health Security Agency - Colindale	

Segment ID	Segment	Country	Collection date	Isolate-ID	Isolate name	Originating Lab	Submitting Lab	Authors
EPI1746956	HA	United Kingdom	2020-Jan-01	EPI_ISL_471024	A/England/200240787/2020	UK Health Security Agency - Colindale	UK Health Security Agency - Colindale	
EPI1746948	HA	United Kingdom	2020-Jan-01	EPI_ISL_471023	A/England/200240786/2020	UK Health Security Agency - Colindale	UK Health Security Agency - Colindale	
EPI1746940	HA	United Kingdom	2020-Jan-08	EPI_ISL_471022	A/England/200240784/2020	UK Health Security Agency - Colindale	UK Health Security Agency - Colindale	
EPI1746932	HA	United Kingdom	2019-Dec-31	EPI_ISL_471021	A/England/200240782/2019	UK Health Security Agency - Colindale	UK Health Security Agency - Colindale	
EPI1746908	HA	United Kingdom	2019-Dec-11	EPI_ISL_471018	A/England/200240763/2019	UK Health Security Agency - Colindale	UK Health Security Agency - Colindale	
EPI1746888	HA	United Kingdom	2019-Dec-15	EPI_ISL_471014	A/England/200240757/2019	UK Health Security Agency - Colindale	UK Health Security Agency - Colindale	
EPI1746878	HA	United Kingdom	2019-Dec-26	EPI_ISL_471012	A/England/200240755/2019	UK Health Security Agency - Colindale	UK Health Security Agency - Colindale	
EPI1746870	HA	United Kingdom	2019-Dec-19	EPI_ISL_471011	A/England/200240754/2019	UK Health Security Agency - Colindale	UK Health Security Agency - Colindale	
EPI1746862	HA	United Kingdom	2019-Dec-20	EPI_ISL_471010	A/England/200220539/2019	UK Health Security Agency - Colindale	UK Health Security Agency - Colindale	
EPI1746854	HA	United Kingdom	2020-Jan-07	EPI_ISL_471009	A/England/200220536/2020	UK Health Security Agency - Colindale	UK Health Security Agency - Colindale	
EPI1746846	HA	United Kingdom	2020-Jan-07	EPI_ISL_471008	A/England/200220535/2020	UK Health Security Agency - Colindale	UK Health Security Agency - Colindale	
EPI1746844	HA	United Kingdom	2020-Jan-07	EPI_ISL_471007	A/England/55/2020	UK Health Security Agency - Colindale	UK Health Security Agency - Colindale	
EPI1746836	HA	United Kingdom	2020-Jan-07	EPI_ISL_471006	A/England/200220529/2020	UK Health Security Agency - Colindale	UK Health Security Agency - Colindale	

Segment ID	Segment	Country	Collection date	Isolate-ID	Isolate name	Originating Lab	Submitting Lab	Authors
EPI1746828	HA	United Kingdom	2020-Jan-07	EPI_ISL_471005	A/England/200220528/2020	UK Health Security Agency - Colindale	UK Health Security Agency - Colindale	
EPI1746826	HA	United Kingdom	2020-Jan-07	EPI_ISL_471004	A/England/200220527/2020	UK Health Security Agency - Colindale	UK Health Security Agency - Colindale	
EPI1746810	HA	United Kingdom	2020-Jan-07	EPI_ISL_471002	A/England/200220525/2020	UK Health Security Agency - Colindale	UK Health Security Agency - Colindale	
EPI1746802	HA	United Kingdom	2019-Dec-31	EPI_ISL_471001	A/England/200220522/2019	UK Health Security Agency - Colindale	UK Health Security Agency - Colindale	
EPI1746794	HA	United Kingdom	2019-Dec-30	EPI_ISL_471000	A/England/200220521/2019	UK Health Security Agency - Colindale	UK Health Security Agency - Colindale	
EPI1746786	HA	United Kingdom	2020-Jan-07	EPI_ISL_470999	A/England/200220518/2020	UK Health Security Agency - Colindale	UK Health Security Agency - Colindale	
EPI1746762	HA	United Kingdom	2019-Dec-27	EPI_ISL_470996	A/England/908/2019	UK Health Security Agency - Colindale	UK Health Security Agency - Colindale	
EPI1746754	HA	United Kingdom	2019-Dec-31	EPI_ISL_470995	A/England/200200774/2019	UK Health Security Agency - Colindale	UK Health Security Agency - Colindale	
EPI1746746	HA	United Kingdom	2019-Dec-28	EPI_ISL_470994	A/England/200200773/2019	UK Health Security Agency - Colindale	UK Health Security Agency - Colindale	
EPI1746738	HA	United Kingdom	2019-Dec-31	EPI_ISL_470993	A/England/200200772/2019	UK Health Security Agency - Colindale	UK Health Security Agency - Colindale	
EPI1746726	HA	United Kingdom	2019-Dec-29	EPI_ISL_470990	A/England/905/2019	UK Health Security Agency - Colindale	UK Health Security Agency - Colindale	
EPI1746702	HA	United Kingdom	2019-Dec-30	EPI_ISL_470987	A/England/893/2019	UK Health Security Agency - Colindale	UK Health Security Agency - Colindale	
EPI1746694	HA	United Kingdom	2019-Dec-30	EPI_ISL_470986	A/England/892/2019	UK Health Security Agency - Colindale	UK Health Security Agency - Colindale	

Segment ID	Segment	Country	Collection date	Isolate-ID	Isolate name	Originating Lab	Submitting Lab	Authors
EPI1746686	HA	United Kingdom	2019-Dec-30	EPI_ISL_470980	A/England/891/2019	UK Health Security Agency - Colindale	UK Health Security Agency - Colindale	
EPI1746678	HA	United Kingdom	2019-Dec-23	EPI_ISL_470965	A/England/200120945/2019	UK Health Security Agency - Colindale	UK Health Security Agency - Colindale	
EPI1746670	HA	United Kingdom	2019-Dec-30	EPI_ISL_470951	A/England/200120646/2019	UK Health Security Agency - Colindale	UK Health Security Agency - Colindale	
EPI1746662	HA	United Kingdom	2019-Dec-30	EPI_ISL_470950	A/England/887/2019	UK Health Security Agency - Colindale	UK Health Security Agency - Colindale	
EPI1746654	HA	United Kingdom	2019-Dec-24	EPI_ISL_470949	A/England/886/2019	UK Health Security Agency - Colindale	UK Health Security Agency - Colindale	
EPI1746646	HA	United Kingdom	2019-Dec-30	EPI_ISL_470948	A/England/200120622/2019	UK Health Security Agency - Colindale	UK Health Security Agency - Colindale	
EPI1746638	HA	United Kingdom	2019-Dec-20	EPI_ISL_470947	A/England/885/2019	UK Health Security Agency - Colindale	UK Health Security Agency - Colindale	
EPI1746630	HA	United Kingdom	2019-Dec-23	EPI_ISL_470946	A/England/884/2019	UK Health Security Agency - Colindale	UK Health Security Agency - Colindale	
EPI1746622	HA	United Kingdom	2019-Dec-27	EPI_ISL_470945	A/England/883/2019	UK Health Security Agency - Colindale	UK Health Security Agency - Colindale	
EPI1746614	HA	United Kingdom	2019-Dec-26	EPI_ISL_470944	A/England/200100406/2019	UK Health Security Agency - Colindale	UK Health Security Agency - Colindale	
EPI1746606	HA	United Kingdom	2019-Dec-16	EPI_ISL_470943	A/England/925/2019	UK Health Security Agency - Colindale	UK Health Security Agency - Colindale	
EPI1746598	HA	United Kingdom	2019-Dec-19	EPI_ISL_470942	A/England/200100391/2019	UK Health Security Agency - Colindale	UK Health Security Agency - Colindale	
EPI1746590	HA	United Kingdom	2019-Dec-23	EPI_ISL_470941	A/England/921/2019	UK Health Security Agency - Colindale	UK Health Security Agency - Colindale	

Segment ID	Segment	Country	Collection date	Isolate-ID	Isolate name	Originating Lab	Submitting Lab	Authors
EPI1746582	HA	United Kingdom	2019-Dec-24	EPI_ISL_470940	A/England/920/2019	UK Health Security Agency - Colindale	UK Health Security Agency - Colindale	
EPI1746574	HA	United Kingdom	2019-Dec-24	EPI_ISL_470939	A/England/919/2019	UK Health Security Agency - Colindale	UK Health Security Agency - Colindale	
EPI1746566	HA	United Kingdom	2019-Dec-27	EPI_ISL_470938	A/England/200100180/2019	UK Health Security Agency - Colindale	UK Health Security Agency - Colindale	
EPI1746558	HA	United Kingdom	2019-Dec-24	EPI_ISL_470937	A/England/918/2019	UK Health Security Agency - Colindale	UK Health Security Agency - Colindale	
EPI1746550	HA	United Kingdom	2019-Dec-24	EPI_ISL_470936	A/England/876/2019	UK Health Security Agency - Colindale	UK Health Security Agency - Colindale	
EPI1746534	HA	United Kingdom	2019-Dec-23	EPI_ISL_470934	A/England/917/2019	UK Health Security Agency - Colindale	UK Health Security Agency - Colindale	
EPI1746526	HA	United Kingdom	2019-Dec-24	EPI_ISL_470933	A/England/916/2019	UK Health Security Agency - Colindale	UK Health Security Agency - Colindale	
EPI1746518	HA	United Kingdom	2019-Dec-23	EPI_ISL_470932	A/England/200100150/2019	UK Health Security Agency - Colindale	UK Health Security Agency - Colindale	
EPI1746510	HA	United Kingdom	2019-Dec-23	EPI_ISL_470931	A/England/200100148/2019	UK Health Security Agency - Colindale	UK Health Security Agency - Colindale	
EPI1746502	HA	United Kingdom	2019-Dec-23	EPI_ISL_470930	A/England/915/2019	UK Health Security Agency - Colindale	UK Health Security Agency - Colindale	
EPI1746494	HA	United Kingdom	2019-Dec-24	EPI_ISL_470929	A/England/914/2019	UK Health Security Agency - Colindale	UK Health Security Agency - Colindale	
EPI1746486	HA	United Kingdom	2019-Dec-20	EPI_ISL_470928	A/England/913/2019	UK Health Security Agency - Colindale	UK Health Security Agency - Colindale	
EPI1746478	HA	United Kingdom	2019-Dec-27	EPI_ISL_470927	A/England/875/2019	UK Health Security Agency - Colindale	UK Health Security Agency - Colindale	

Segment ID	Segment	Country	Collection date	Isolate-ID	Isolate name	Originating Lab	Submitting Lab	Authors
EPI1746470	HA	United Kingdom	2019-Dec-23	EPI_ISL_470926	A/England/873/2019	UK Health Security Agency - Colindale	UK Health Security Agency - Colindale	
EPI1746462	HA	United Kingdom	2019-Dec-18	EPI_ISL_470925	A/England/872/2019	UK Health Security Agency - Colindale	UK Health Security Agency - Colindale	
EPI1746438	HA	United Kingdom	2019-Dec-23	EPI_ISL_470922	A/England/871/2019	UK Health Security Agency - Colindale	UK Health Security Agency - Colindale	
EPI1746430	HA	United Kingdom	2019-Dec-23	EPI_ISL_470921	A/England/870/2019	UK Health Security Agency - Colindale	UK Health Security Agency - Colindale	
EPI1746422	HA	United Kingdom	2019-Dec-23	EPI_ISL_470920	A/England/868/2019	UK Health Security Agency - Colindale	UK Health Security Agency - Colindale	
EPI1746414	HA	United Kingdom	2019-Dec-19	EPI_ISL_470919	A/England/867/2019	UK Health Security Agency - Colindale	UK Health Security Agency - Colindale	
EPI1746406	HA	United Kingdom	2019-Dec-23	EPI_ISL_470918	A/England/866/2019	UK Health Security Agency - Colindale	UK Health Security Agency - Colindale	
EPI1746390	HA	United Kingdom	2019-Dec-23	EPI_ISL_470916	A/England/865/2019	UK Health Security Agency - Colindale	UK Health Security Agency - Colindale	
EPI1746382	HA	United Kingdom	2019-Dec-23	EPI_ISL_470915	A/England/195220316/2019	UK Health Security Agency - Colindale	UK Health Security Agency - Colindale	
EPI1746374	HA	United Kingdom	2019-Dec-18	EPI_ISL_470914	A/England/864/2019	UK Health Security Agency - Colindale	UK Health Security Agency - Colindale	
EPI1746366	HA	United Kingdom	2019-Dec-20	EPI_ISL_470913	A/England/856/2019	UK Health Security Agency - Colindale	UK Health Security Agency - Colindale	
EPI1746358	HA	United Kingdom	2019-Dec-19	EPI_ISL_470912	A/England/855/2019	UK Health Security Agency - Colindale	UK Health Security Agency - Colindale	
EPI1746350	HA	United Kingdom	2019-Dec-18	EPI_ISL_470911	A/England/195200851/2019	UK Health Security Agency - Colindale	UK Health Security Agency - Colindale	

Segment ID	Segment	Country	Collection date	Isolate-ID	Isolate name	Originating Lab	Submitting Lab	Authors
EPI1746342	HA	United Kingdom	2019-Dec-19	EPI_ISL_470910	A/England/195200845/2019	UK Health Security Agency - Colindale	UK Health Security Agency - Colindale	
EPI1746334	HA	United Kingdom	2019-Dec-18	EPI_ISL_470909	A/England/195200840/2019	UK Health Security Agency - Colindale	UK Health Security Agency - Colindale	
EPI1746290	HA	United Kingdom	2020-Jan-03	EPI_ISL_470893	A/England/200240795/2020	UK Health Security Agency - Colindale	UK Health Security Agency - Colindale	
EPI1746286	HA	United Kingdom	2019-Dec-09	EPI_ISL_470891	A/England/200240748/2019	UK Health Security Agency - Colindale	UK Health Security Agency - Colindale	
EPI1746282	HA	United Kingdom	2020-Jan-07	EPI_ISL_470889	A/England/200220538/2020	UK Health Security Agency - Colindale	UK Health Security Agency - Colindale	
EPI1746280	HA	United Kingdom	2020-Jan-07	EPI_ISL_470888	A/England/200220537/2020	UK Health Security Agency - Colindale	UK Health Security Agency - Colindale	
EPI1746278	HA	United Kingdom	2020-Jan-07	EPI_ISL_470887	A/England/200220532/2020	UK Health Security Agency - Colindale	UK Health Security Agency - Colindale	
EPI1746276	HA	United Kingdom	2020-Jan-07	EPI_ISL_470886	A/England/200220531/2020	UK Health Security Agency - Colindale	UK Health Security Agency - Colindale	
EPI1746274	HA	United Kingdom	2020-Jan-07	EPI_ISL_470885	A/England/200220530/2020	UK Health Security Agency - Colindale	UK Health Security Agency - Colindale	
EPI1746272	HA	United Kingdom	2019-Dec-30	EPI_ISL_470884	A/England/200220520/2019	UK Health Security Agency - Colindale	UK Health Security Agency - Colindale	
EPI1746270	HA	United Kingdom	2019-Dec-31	EPI_ISL_470883	A/England/200200354/2019	UK Health Security Agency - Colindale	UK Health Security Agency - Colindale	
EPI1666946	HA	United Kingdom	2019-Nov-25	EPI_ISL_405271	A/England/522/2019	UK Health Security Agency - Colindale	UK Health Security Agency - Colindale	Thompson,C.
EPI1666938	HA	United Kingdom	2019-Nov-22	EPI_ISL_405270	A/England/602/2019	UK Health Security Agency - Colindale	UK Health Security Agency - Colindale	Thompson,C.

Segment ID	Segment	Country	Collection date	Isolate-ID	Isolate name	Originating Lab	Submitting Lab	Authors
EPI1666930	HA	United Kingdom	2019-Nov-21	EPI_ISL_405269	A/England/194880738/2019	UK Health Security Agency - Colindale	UK Health Security Agency - Colindale	Thompson,C.
EPI1666922	HA	United Kingdom	2019-Nov-23	EPI_ISL_405268	A/England/518/2019	UK Health Security Agency - Colindale	UK Health Security Agency - Colindale	Thompson,C.
EPI1666914	HA	United Kingdom	2019-Nov-22	EPI_ISL_405267	A/England/517/2019	UK Health Security Agency - Colindale	UK Health Security Agency - Colindale	Thompson,C.
EPI1666906	HA	United Kingdom	2019-Nov-25	EPI_ISL_405266	A/England/194880442/2019	UK Health Security Agency - Colindale	UK Health Security Agency - Colindale	Thompson,C.
EPI1666898	HA	United Kingdom	2019-Nov-24	EPI_ISL_405265	A/England/492/2019	UK Health Security Agency - Colindale	UK Health Security Agency - Colindale	Thompson,C.
EPI1666890	HA	United Kingdom	2019-Nov-23	EPI_ISL_405264	A/England/194860494/2019	UK Health Security Agency - Colindale	UK Health Security Agency - Colindale	Thompson,C.
EPI1666882	HA	United Kingdom	2019-Nov-25	EPI_ISL_405263	A/England/194860493/2019	UK Health Security Agency - Colindale	UK Health Security Agency - Colindale	Thompson,C.
EPI1666874	HA	United Kingdom	2019-Nov-25	EPI_ISL_405262	A/England/490/2019	UK Health Security Agency - Colindale	UK Health Security Agency - Colindale	Thompson,C.
EPI1666866	HA	United Kingdom	2019-Nov-24	EPI_ISL_405261	A/England/489/2019	UK Health Security Agency - Colindale	UK Health Security Agency - Colindale	Thompson,C.
EPI1666858	HA	United Kingdom	2019-Nov-24	EPI_ISL_405260	A/England/194860484/2019	UK Health Security Agency - Colindale	UK Health Security Agency - Colindale	Thompson,C.
EPI1666850	HA	United Kingdom	2019-Nov-20	EPI_ISL_405259	A/England/488/2019	UK Health Security Agency - Colindale	UK Health Security Agency - Colindale	Thompson,C.
EPI1666842	HA	United Kingdom	2019-Nov-25	EPI_ISL_405258	A/England/487/2019	UK Health Security Agency - Colindale	UK Health Security Agency - Colindale	Thompson,C.
EPI1666834	HA	United Kingdom	2019-Nov-25	EPI_ISL_405257	A/England/194860477/2019	UK Health Security Agency - Colindale	UK Health Security Agency - Colindale	Thompson,C.

Segment ID	Segment	Country	Collection date	Isolate-ID	Isolate name	Originating Lab	Submitting Lab	Authors
EPI1666826	HA	United Kingdom	2019-Nov-18	EPI_ISL_405256	A/England/482/2019	UK Health Security Agency - Colindale	UK Health Security Agency - Colindale	Thompson,C.
EPI1666818	HA	United Kingdom	2019-Nov-19	EPI_ISL_405255	A/England/481/2019	UK Health Security Agency - Colindale	UK Health Security Agency - Colindale	Thompson,C.
EPI1666810	HA	United Kingdom	2019-Nov-15	EPI_ISL_405254	A/England/194840330/2019	UK Health Security Agency - Colindale	UK Health Security Agency - Colindale	Thompson,C.
EPI1666802	HA	United Kingdom	2019-Nov-20	EPI_ISL_405253	A/England/478/2019	UK Health Security Agency - Colindale	UK Health Security Agency - Colindale	Thompson,C.
EPI1666794	HA	United Kingdom	2019-Nov-20	EPI_ISL_405252	A/England/477/2019	UK Health Security Agency - Colindale	UK Health Security Agency - Colindale	Thompson,C.
EPI1666786	HA	United Kingdom	2019-Nov-20	EPI_ISL_405251	A/England/476/2019	UK Health Security Agency - Colindale	UK Health Security Agency - Colindale	Thompson,C.
EPI1666778	HA	United Kingdom	2019-Nov-19	EPI_ISL_405250	A/England/194840318/2019	UK Health Security Agency - Colindale	UK Health Security Agency - Colindale	Thompson,C.
EPI1666770	HA	United Kingdom	2019-Nov-23	EPI_ISL_405249	A/England/474/2019	UK Health Security Agency - Colindale	UK Health Security Agency - Colindale	Thompson,C.
EPI1666762	HA	United Kingdom	2019-Nov-22	EPI_ISL_405248	A/England/194840307/2019	UK Health Security Agency - Colindale	UK Health Security Agency - Colindale	Thompson,C.
EPI1666754	HA	United Kingdom	2019-Nov-22	EPI_ISL_405247	A/England/194840306/2019	UK Health Security Agency - Colindale	UK Health Security Agency - Colindale	Thompson,C.
EPI1666746	HA	United Kingdom	2019-Nov-18	EPI_ISL_405246	A/England/194840305/2019	UK Health Security Agency - Colindale	UK Health Security Agency - Colindale	Thompson,C.
EPI1666738	HA	United Kingdom	2019-Nov-21	EPI_ISL_405245	A/England/194840303/2019	UK Health Security Agency - Colindale	UK Health Security Agency - Colindale	Thompson,C.
EPI1666730	HA	United Kingdom	2019-Nov-24	EPI_ISL_405244	A/England/194840300/2019	UK Health Security Agency - Colindale	UK Health Security Agency - Colindale	Thompson,C.

Segment ID	Segment	Country	Collection date	Isolate-ID	Isolate name	Originating Lab	Submitting Lab	Authors
EPI1666722	HA	United Kingdom	2019-Nov-24	EPI_ISL_405243	A/England/194840298/2019	UK Health Security Agency - Colindale	UK Health Security Agency - Colindale	Thompson,C.
EPI1666714	HA	United Kingdom	2019-Nov-24	EPI_ISL_405242	A/England/194840297/2019	UK Health Security Agency - Colindale	UK Health Security Agency - Colindale	Thompson,C.
EPI1666706	HA	United Kingdom	2019-Nov-20	EPI_ISL_405241	A/England/194840296/2019	UK Health Security Agency - Colindale	UK Health Security Agency - Colindale	Thompson,C.
EPI1666698	HA	United Kingdom	2019-Nov-20	EPI_ISL_405240	A/England/502/2019	UK Health Security Agency - Colindale	UK Health Security Agency - Colindale	Thompson,C.
EPI1666690	HA	United Kingdom	2019-Nov-19	EPI_ISL_405239	A/England/194840294/2019	UK Health Security Agency - Colindale	UK Health Security Agency - Colindale	Thompson,C.
EPI1666682	HA	United Kingdom	2019-Oct-20	EPI_ISL_405238	A/England/501/2019	UK Health Security Agency - Colindale	UK Health Security Agency - Colindale	Thompson,C.
EPI1666674	HA	United Kingdom	2019-Nov-21	EPI_ISL_405237	A/England/472/2019	UK Health Security Agency - Colindale	UK Health Security Agency - Colindale	Thompson,C.
EPI1666666	HA	United Kingdom	2019-Nov-19	EPI_ISL_405236	A/England/194840289/2019	UK Health Security Agency - Colindale	UK Health Security Agency - Colindale	Thompson,C.
EPI1666658	HA	United Kingdom	2019-Nov-21	EPI_ISL_405235	A/England/471/2019	UK Health Security Agency - Colindale	UK Health Security Agency - Colindale	Thompson,C.
EPI1666650	HA	United Kingdom	2019-Nov-17	EPI_ISL_405234	A/England/194820760/2019	UK Health Security Agency - Colindale	UK Health Security Agency - Colindale	Thompson,C.
EPI1666642	HA	United Kingdom	2019-Nov-17	EPI_ISL_405233	A/England/194820759/2019	UK Health Security Agency - Colindale	UK Health Security Agency - Colindale	Thompson,C.
EPI1666634	HA	United Kingdom	2019-Nov-21	EPI_ISL_405232	A/England/194820756/2019	UK Health Security Agency - Colindale	UK Health Security Agency - Colindale	Thompson,C.
EPI1666626	HA	United Kingdom	2019-Nov-25	EPI_ISL_405231	A/England/465/2019	UK Health Security Agency - Colindale	UK Health Security Agency - Colindale	Thompson,C.

Segment ID	Segment	Country	Collection date	Isolate-ID	Isolate name	Originating Lab	Submitting Lab	Authors
EPI1666618	HA	United Kingdom	2019-Nov-22	EPI_ISL_405230	A/England/194820126/2019	UK Health Security Agency - Colindale	UK Health Security Agency - Colindale	Thompson,C.
EPI1666610	HA	United Kingdom	2019-Nov-19	EPI_ISL_405229	A/England/194800521/2019	UK Health Security Agency - Colindale	UK Health Security Agency - Colindale	Thompson,C.
EPI1666602	HA	United Kingdom	2019-Nov-19	EPI_ISL_405228	A/England/194800324/2019	UK Health Security Agency - Colindale	UK Health Security Agency - Colindale	Thompson,C.
EPI1666594	HA	United Kingdom	2019-Nov-19	EPI_ISL_405227	A/England/194800321/2019	UK Health Security Agency - Colindale	UK Health Security Agency - Colindale	Thompson,C.
EPI1666586	HA	United Kingdom	2019-Nov-20	EPI_ISL_405226	A/England/194800320/2019	UK Health Security Agency - Colindale	UK Health Security Agency - Colindale	Thompson,C.
EPI1666578	HA	United Kingdom	2019-Nov-18	EPI_ISL_405225	A/England/463/2019	UK Health Security Agency - Colindale	UK Health Security Agency - Colindale	Thompson,C.
EPI1666570	HA	United Kingdom	2019-Nov-19	EPI_ISL_405224	A/England/462/2019	UK Health Security Agency - Colindale	UK Health Security Agency - Colindale	Thompson,C.
EPI1666562	HA	United Kingdom	2019-Nov-15	EPI_ISL_405223	A/England/194800310/2019	UK Health Security Agency - Colindale	UK Health Security Agency - Colindale	Thompson,C.
EPI1666554	HA	United Kingdom	2019-Nov-15	EPI_ISL_405222	A/England/194800309/2019	UK Health Security Agency - Colindale	UK Health Security Agency - Colindale	Thompson,C.
EPI1666546	HA	United Kingdom	2019-Nov-18	EPI_ISL_405221	A/England/194800308/2019	UK Health Security Agency - Colindale	UK Health Security Agency - Colindale	Thompson,C.
EPI1666538	HA	United Kingdom	2019-Nov-18	EPI_ISL_405220	A/England/194800306/2019	UK Health Security Agency - Colindale	UK Health Security Agency - Colindale	Thompson,C.
EPI1666530	HA	United Kingdom	2019-Nov-18	EPI_ISL_405219	A/England/194800305/2019	UK Health Security Agency - Colindale	UK Health Security Agency - Colindale	Thompson,C.
EPI1666522	HA	United Kingdom	2019-Nov-18	EPI_ISL_405218	A/England/459/2019	UK Health Security Agency - Colindale	UK Health Security Agency - Colindale	Thompson,C.

Segment ID	Segment	Country	Collection date	Isolate-ID	Isolate name	Originating Lab	Submitting Lab	Authors
EPI1666514	HA	United Kingdom	2019-Nov-15	EPI_ISL_405217	A/England/194800303/2019	UK Health Security Agency - Colindale	UK Health Security Agency - Colindale	Thompson,C.
EPI1666506	HA	United Kingdom	2019-Nov-18	EPI_ISL_405216	A/England/499/2019	UK Health Security Agency - Colindale	UK Health Security Agency - Colindale	Thompson,C.
EPI1666498	HA	United Kingdom	2019-Nov-18	EPI_ISL_405215	A/England/194800301/2019	UK Health Security Agency - Colindale	UK Health Security Agency - Colindale	Thompson,C.
EPI1666490	HA	United Kingdom	2019-Nov-18	EPI_ISL_405214	A/England/458/2019	UK Health Security Agency - Colindale	UK Health Security Agency - Colindale	Thompson,C.
EPI1666482	HA	United Kingdom	2019-Nov-13	EPI_ISL_405213	A/England/194800296/2019	UK Health Security Agency - Colindale	UK Health Security Agency - Colindale	Thompson,C.
EPI1666474	HA	United Kingdom	2019-Nov-18	EPI_ISL_405212	A/England/194800294/2019	UK Health Security Agency - Colindale	UK Health Security Agency - Colindale	Thompson,C.
EPI1666466	HA	United Kingdom	2019-Nov-19	EPI_ISL_405211	A/England/194800293/2019	UK Health Security Agency - Colindale	UK Health Security Agency - Colindale	Thompson,C.
EPI1666458	HA	United Kingdom	2019-Nov-18	EPI_ISL_405210	A/England/456/2019	UK Health Security Agency - Colindale	UK Health Security Agency - Colindale	Thompson,C.
EPI1666450	HA	United Kingdom	2019-Nov-19	EPI_ISL_405209	A/England/194800291/2019	UK Health Security Agency - Colindale	UK Health Security Agency - Colindale	Thompson,C.
EPI1666442	HA	United Kingdom	2019-Nov-20	EPI_ISL_405208	A/England/194800289/2019	UK Health Security Agency - Colindale	UK Health Security Agency - Colindale	Thompson,C.
EPI1666434	HA	United Kingdom	2019-Nov-20	EPI_ISL_405207	A/England/455/2019	UK Health Security Agency - Colindale	UK Health Security Agency - Colindale	Thompson,C.
EPI1666426	HA	United Kingdom	2019-Nov-20	EPI_ISL_405206	A/England/194800287/2019	UK Health Security Agency - Colindale	UK Health Security Agency - Colindale	Thompson,C.
EPI1666418	HA	United Kingdom	2019-Nov-20	EPI_ISL_405205	A/England/194800286/2019	UK Health Security Agency - Colindale	UK Health Security Agency - Colindale	Thompson,C.

Segment ID	Segment	Country	Collection date	Isolate-ID	Isolate name	Originating Lab	Submitting Lab	Authors
EPI1666410	HA	United Kingdom	2019-Nov-20	EPI_ISL_405204	A/England/194800285/2019	UK Health Security Agency - Colindale	UK Health Security Agency - Colindale	Thompson,C.
EPI1666402	HA	United Kingdom	2019-Nov-18	EPI_ISL_405203	A/England/453/2019	UK Health Security Agency - Colindale	UK Health Security Agency - Colindale	Thompson,C.
EPI1666394	HA	United Kingdom	2019-Nov-18	EPI_ISL_405202	A/England/452/2019	UK Health Security Agency - Colindale	UK Health Security Agency - Colindale	Thompson,C.
EPI1666386	HA	United Kingdom	2019-Nov-15	EPI_ISL_405201	A/England/194800281/2019	UK Health Security Agency - Colindale	UK Health Security Agency - Colindale	Thompson,C.
EPI1666378	HA	United Kingdom	2019-Nov-22	EPI_ISL_405200	A/England/450/2019	UK Health Security Agency - Colindale	UK Health Security Agency - Colindale	Thompson,C.
EPI1666370	HA	United Kingdom	2019-Nov-21	EPI_ISL_405199	A/England/448/2019	UK Health Security Agency - Colindale	UK Health Security Agency - Colindale	Thompson,C.
EPI1666362	HA	United Kingdom	2019-Nov-20	EPI_ISL_405198	A/England/446/2019	UK Health Security Agency - Colindale	UK Health Security Agency - Colindale	Thompson,C.
EPI1666354	HA	United Kingdom	2019-Nov-20	EPI_ISL_405197	A/England/444/2019	UK Health Security Agency - Colindale	UK Health Security Agency - Colindale	Thompson,C.
EPI1666346	HA	United Kingdom	2019-Nov-20	EPI_ISL_405196	A/England/194780654/2019	UK Health Security Agency - Colindale	UK Health Security Agency - Colindale	Thompson,C.
EPI1666338	HA	United Kingdom	2019-Nov-21	EPI_ISL_405195	A/England/442/2019	UK Health Security Agency - Colindale	UK Health Security Agency - Colindale	Thompson,C.
EPI1666330	HA	United Kingdom	2019-Nov-21	EPI_ISL_405194	A/England/440/2019	UK Health Security Agency - Colindale	UK Health Security Agency - Colindale	Thompson,C.
EPI1666322	HA	United Kingdom	2019-Nov-17	EPI_ISL_405193	A/England/438/2019	UK Health Security Agency - Colindale	UK Health Security Agency - Colindale	Thompson,C.
EPI1666314	HA	United Kingdom	2019-Nov-17	EPI_ISL_405192	A/England/437/2019	UK Health Security Agency - Colindale	UK Health Security Agency - Colindale	Thompson,C.

Segment ID	Segment	Country	Collection date	Isolate-ID	Isolate name	Originating Lab	Submitting Lab	Authors
EPI1666306	HA	United Kingdom	2019-Nov-18	EPI_ISL_405191	A/England/498/2019	UK Health Security Agency - Colindale	UK Health Security Agency - Colindale	Thompson,C.
EPI1666298	HA	United Kingdom	2019-Nov-18	EPI_ISL_405190	A/England/427/2019	UK Health Security Agency - Colindale	UK Health Security Agency - Colindale	Thompson,C.
EPI1666290	HA	United Kingdom	2019-Nov-18	EPI_ISL_405189	A/England/194780297/2019	UK Health Security Agency - Colindale	UK Health Security Agency - Colindale	Thompson,C.
EPI1666282	HA	United Kingdom	2019-Nov-14	EPI_ISL_405188	A/England/425/2019	UK Health Security Agency - Colindale	UK Health Security Agency - Colindale	Thompson,C.
EPI1666274	HA	United Kingdom	2019-Nov-15	EPI_ISL_405187	A/England/424/2019	UK Health Security Agency - Colindale	UK Health Security Agency - Colindale	Thompson,C.
EPI1666266	HA	United Kingdom	2019-Nov-16	EPI_ISL_405186	A/England/423/2019	UK Health Security Agency - Colindale	UK Health Security Agency - Colindale	Thompson,C.
EPI1666258	HA	United Kingdom	2019-Nov-15	EPI_ISL_405185	A/England/422/2019	UK Health Security Agency - Colindale	UK Health Security Agency - Colindale	Thompson,C.
EPI1666250	HA	United Kingdom	2019-Nov-15	EPI_ISL_405184	A/England/421/2019	UK Health Security Agency - Colindale	UK Health Security Agency - Colindale	Thompson,C.
EPI1666242	HA	United Kingdom	2019-Nov-16	EPI_ISL_405183	A/England/194780289/2019	UK Health Security Agency - Colindale	UK Health Security Agency - Colindale	Thompson,C.
EPI1666234	HA	United Kingdom	2019-Nov-16	EPI_ISL_405182	A/England/194780288/2019	UK Health Security Agency - Colindale	UK Health Security Agency - Colindale	Thompson,C.
EPI1666226	HA	United Kingdom	2019-Nov-16	EPI_ISL_405181	A/England/194780287/2019	UK Health Security Agency - Colindale	UK Health Security Agency - Colindale	Thompson,C.
EPI1666218	HA	United Kingdom	2019-Nov-17	EPI_ISL_405180	A/England/420/2019	UK Health Security Agency - Colindale	UK Health Security Agency - Colindale	Thompson,C.
EPI1666210	HA	United Kingdom	2019-Nov-16	EPI_ISL_405179	A/England/419/2019	UK Health Security Agency - Colindale	UK Health Security Agency - Colindale	Thompson,C.

Segment ID	Segment	Country	Collection date	Isolate-ID	Isolate name	Originating Lab	Submitting Lab	Authors
EPI1666202	HA	United Kingdom	2019-Nov-18	EPI_ISL_405178	A/England/418/2019	UK Health Security Agency - Colindale	UK Health Security Agency - Colindale	Thompson,C.
EPI1666194	HA	United Kingdom	2019-Nov-22	EPI_ISL_405177	A/England/429/2019	UK Health Security Agency - Colindale	UK Health Security Agency - Colindale	Thompson,C.
EPI1666186	HA	United Kingdom	2019-Nov-15	EPI_ISL_405176	A/England/417/2019	UK Health Security Agency - Colindale	UK Health Security Agency - Colindale	Thompson,C.
EPI1666178	HA	United Kingdom	2019-Nov-18	EPI_ISL_405175	A/England/194780278/2019	UK Health Security Agency - Colindale	UK Health Security Agency - Colindale	Thompson,C.
EPI1666162	HA	United Kingdom	2019-Nov-18	EPI_ISL_405173	A/England/194780208/2019	UK Health Security Agency - Colindale	UK Health Security Agency - Colindale	Thompson,C.
EPI1666154	HA	United Kingdom	2019-Nov-16	EPI_ISL_405172	A/England/415/2019	UK Health Security Agency - Colindale	UK Health Security Agency - Colindale	Thompson,C.
EPI1666146	HA	United Kingdom	2019-Nov-15	EPI_ISL_405171	A/England/413/2019	UK Health Security Agency - Colindale	UK Health Security Agency - Colindale	Thompson,C.
EPI1666138	HA	United Kingdom	2019-Nov-19	EPI_ISL_405170	A/England/412/2019	UK Health Security Agency - Colindale	UK Health Security Agency - Colindale	Thompson,C.
EPI1666130	HA	United Kingdom	2019-Nov-19	EPI_ISL_405169	A/England/411/2019	UK Health Security Agency - Colindale	UK Health Security Agency - Colindale	Thompson,C.
EPI1666122	HA	United Kingdom	2019-Nov-15	EPI_ISL_405168	A/England/410/2019	UK Health Security Agency - Colindale	UK Health Security Agency - Colindale	Thompson,C.
EPI1666114	HA	United Kingdom	2019-Nov-17	EPI_ISL_405167	A/England/409/2019	UK Health Security Agency - Colindale	UK Health Security Agency - Colindale	Thompson,C.
EPI1666106	HA	United Kingdom	2019-Nov-15	EPI_ISL_405166	A/England/408/2019	UK Health Security Agency - Colindale	UK Health Security Agency - Colindale	Thompson,C.
EPI1666098	HA	United Kingdom	2019-Nov-19	EPI_ISL_405165	A/England/407/2019	UK Health Security Agency - Colindale	UK Health Security Agency - Colindale	Thompson,C.

Segment ID	Segment	Country	Collection date	Isolate-ID	Isolate name	Originating Lab	Submitting Lab	Authors
EPI1666090	HA	United Kingdom	2019-Nov-15	EPI_ISL_405164	A/England/406/2019	UK Health Security Agency - Colindale	UK Health Security Agency - Colindale	Thompson,C.
EPI1666082	HA	United Kingdom	2019-Nov-22	EPI_ISL_405163	A/England/405/2019	UK Health Security Agency - Colindale	UK Health Security Agency - Colindale	Thompson,C.
EPI1666074	HA	United Kingdom	2019-Nov-15	EPI_ISL_405162	A/England/194780186/2019	UK Health Security Agency - Colindale	UK Health Security Agency - Colindale	Thompson,C.
EPI1666066	HA	United Kingdom	2019-Nov-19	EPI_ISL_405161	A/England/497/2019	UK Health Security Agency - Colindale	UK Health Security Agency - Colindale	Thompson,C.
EPI1666058	HA	United Kingdom	2019-Nov-16	EPI_ISL_405160	A/England/404/2019	UK Health Security Agency - Colindale	UK Health Security Agency - Colindale	Thompson,C.
EPI1666050	HA	United Kingdom	2019-Nov-15	EPI_ISL_405159	A/England/194780183/2019	UK Health Security Agency - Colindale	UK Health Security Agency - Colindale	Thompson,C.
EPI1666042	HA	United Kingdom	2019-Nov-19	EPI_ISL_405158	A/England/496/2019	UK Health Security Agency - Colindale	UK Health Security Agency - Colindale	Thompson,C.
EPI1666034	HA	United Kingdom	2019-Nov-18	EPI_ISL_405157	A/England/194760535/2019	UK Health Security Agency - Colindale	UK Health Security Agency - Colindale	Thompson,C.
EPI1666026	HA	United Kingdom	2019-Nov-14	EPI_ISL_405156	A/England/402/2019	UK Health Security Agency - Colindale	UK Health Security Agency - Colindale	Thompson,C.
EPI1666018	HA	United Kingdom	2019-Nov-14	EPI_ISL_405155	A/England/401/2019	UK Health Security Agency - Colindale	UK Health Security Agency - Colindale	Thompson,C.
EPI1666010	HA	United Kingdom	2019-Nov-13	EPI_ISL_405154	A/England/495/2019	UK Health Security Agency - Colindale	UK Health Security Agency - Colindale	Thompson,C.
EPI1666002	HA	United Kingdom	2019-Nov-12	EPI_ISL_405153	A/England/436/2019	UK Health Security Agency - Colindale	UK Health Security Agency - Colindale	Thompson,C.
EPI1665994	HA	United Kingdom	2019-Nov-20	EPI_ISL_405152	A/England/457/2019	UK Health Security Agency - Colindale	UK Health Security Agency - Colindale	Thompson,C.

Segment ID	Segment	Country	Collection date	Isolate-ID	Isolate name	Originating Lab	Submitting Lab	Authors
EPI1665986	HA	United Kingdom	2019-Nov-13	EPI_ISL_405151	A/England/194740633/2019	UK Health Security Agency - Colindale	UK Health Security Agency - Colindale	Thompson,C.
EPI1665978	HA	United Kingdom	2019-Nov-13	EPI_ISL_405150	A/England/194740632/2019	UK Health Security Agency - Colindale	UK Health Security Agency - Colindale	Thompson,C.
EPI1665970	HA	United Kingdom	2019-Nov-14	EPI_ISL_405149	A/England/194740631/2019	UK Health Security Agency - Colindale	UK Health Security Agency - Colindale	Thompson,C.
EPI1665962	HA	United Kingdom	2019-Nov-13	EPI_ISL_405148	A/England/599/2019	UK Health Security Agency - Colindale	UK Health Security Agency - Colindale	Thompson,C.
EPI1665954	HA	United Kingdom	2019-Nov-14	EPI_ISL_405147	A/England/194740627/2019	UK Health Security Agency - Colindale	UK Health Security Agency - Colindale	Thompson,C.
EPI1665946	HA	United Kingdom	2019-Nov-04	EPI_ISL_405146	A/England/389/2019	UK Health Security Agency - Colindale	UK Health Security Agency - Colindale	Thompson,C.
EPI1665938	HA	United Kingdom	2019-Nov-13	EPI_ISL_405145	A/England/388/2019	UK Health Security Agency - Colindale	UK Health Security Agency - Colindale	Thompson,C.
EPI1665930	HA	United Kingdom	2019-Nov-13	EPI_ISL_405144	A/England/387/2019	UK Health Security Agency - Colindale	UK Health Security Agency - Colindale	Thompson,C.
EPI1665922	HA	United Kingdom	2019-Nov-11	EPI_ISL_405143	A/England/381/2019	UK Health Security Agency - Colindale	UK Health Security Agency - Colindale	Thompson,C.
EPI1665914	HA	United Kingdom	2019-Nov-12	EPI_ISL_405142	A/England/380/2019	UK Health Security Agency - Colindale	UK Health Security Agency - Colindale	Thompson,C.
EPI1665906	HA	United Kingdom	2019-Nov-11	EPI_ISL_405141	A/England/379/2019	UK Health Security Agency - Colindale	UK Health Security Agency - Colindale	Thompson,C.
EPI1665890	HA	United Kingdom	2019-Nov-15	EPI_ISL_405139	A/England/194680775/2019	UK Health Security Agency - Colindale	UK Health Security Agency - Colindale	Thompson,C.
EPI1665882	HA	United Kingdom	2019-Nov-08	EPI_ISL_405138	A/England/194660516/2019	UK Health Security Agency - Colindale	UK Health Security Agency - Colindale	Thompson,C.

Segment ID	Segment	Country	Collection date	Isolate-ID	Isolate name	Originating Lab	Submitting Lab	Authors
EPI1665874	HA	United Kingdom	2019-Nov-08	EPI_ISL_405137	A/England/194660515/2019	UK Health Security Agency - Colindale	UK Health Security Agency - Colindale	Thompson,C.
EPI1665866	HA	United Kingdom	2019-Nov-09	EPI_ISL_405136	A/England/434/2019	UK Health Security Agency - Colindale	UK Health Security Agency - Colindale	Thompson,C.
EPI1665858	HA	United Kingdom	2019-Nov-09	EPI_ISL_405135	A/England/366/2019	UK Health Security Agency - Colindale	UK Health Security Agency - Colindale	Thompson,C.
EPI1665842	HA	United Kingdom	2019-Nov-08	EPI_ISL_405133	A/England/194620578/2019	UK Health Security Agency - Colindale	UK Health Security Agency - Colindale	Thompson,C.
EPI1665834	HA	United Kingdom	2019-Nov-11	EPI_ISL_405132	A/England/374/2019	UK Health Security Agency - Colindale	UK Health Security Agency - Colindale	Thompson,C.
EPI1665826	HA	United Kingdom	2019-Nov-11	EPI_ISL_405131	A/England/373/2019	UK Health Security Agency - Colindale	UK Health Security Agency - Colindale	Thompson,C.
EPI1665818	HA	United Kingdom	2019-Nov-11	EPI_ISL_405130	A/England/350/2019	UK Health Security Agency - Colindale	UK Health Security Agency - Colindale	Thompson,C.
EPI1665810	HA	United Kingdom	2019-Nov-11	EPI_ISL_405129	A/England/349/2019	UK Health Security Agency - Colindale	UK Health Security Agency - Colindale	Thompson,C.
EPI1665802	HA	United Kingdom	2019-Nov-11	EPI_ISL_405128	A/England/348/2019	UK Health Security Agency - Colindale	UK Health Security Agency - Colindale	Thompson,C.
EPI1665794	HA	United Kingdom	2019-Nov-07	EPI_ISL_405127	A/England/364/2019	UK Health Security Agency - Colindale	UK Health Security Agency - Colindale	Thompson,C.
EPI1665786	HA	United Kingdom	2019-Nov-03	EPI_ISL_405126	A/England/194580411/2019	UK Health Security Agency - Colindale	UK Health Security Agency - Colindale	Thompson,C.
EPI1665778	HA	United Kingdom	2019-Nov-05	EPI_ISL_405125	A/England/363/2019	UK Health Security Agency - Colindale	UK Health Security Agency - Colindale	Thompson,C.
EPI1665770	HA	United Kingdom	2019-Nov-04	EPI_ISL_405124	A/England/194560464/2019	UK Health Security Agency - Colindale	UK Health Security Agency - Colindale	Thompson,C.

Segment ID	Segment	Country	Collection date	Isolate-ID	Isolate name	Originating Lab	Submitting Lab	Authors
EPI1665762	HA	United Kingdom	2019-Nov-04	EPI_ISL_405123	A/England/194560463/2019	UK Health Security Agency - Colindale	UK Health Security Agency - Colindale	Thompson,C.
EPI1665754	HA	United Kingdom	2019-Oct-31	EPI_ISL_405122	A/England/362/2019	UK Health Security Agency - Colindale	UK Health Security Agency - Colindale	Thompson,C.
EPI1665746	HA	United Kingdom	2019-Nov-04	EPI_ISL_405121	A/England/361/2019	UK Health Security Agency - Colindale	UK Health Security Agency - Colindale	Thompson,C.
EPI1665730	HA	United Kingdom	2019-Oct-25	EPI_ISL_405119	A/England/194540759/2019	UK Health Security Agency - Colindale	UK Health Security Agency - Colindale	Thompson,C.
EPI1665722	HA	United Kingdom	2019-Oct-25	EPI_ISL_405118	A/England/194540758/2019	UK Health Security Agency - Colindale	UK Health Security Agency - Colindale	Thompson,C.
EPI1665706	HA	United Kingdom	2019-Oct-19	EPI_ISL_405116	A/England/194540755/2019	UK Health Security Agency - Colindale	UK Health Security Agency - Colindale	Thompson,C.
EPI1665698	HA	United Kingdom	2019-Nov-02	EPI_ISL_405115	A/England/360/2019	UK Health Security Agency - Colindale	UK Health Security Agency - Colindale	Thompson,C.
EPI1665690	HA	United Kingdom	2019-Nov-04	EPI_ISL_405114	A/England/339/2019	UK Health Security Agency - Colindale	UK Health Security Agency - Colindale	Thompson,C.
EPI1665682	HA	United Kingdom	2019-Nov-04	EPI_ISL_405113	A/England/341/2019	UK Health Security Agency - Colindale	UK Health Security Agency - Colindale	Thompson,C.
EPI1665674	HA	United Kingdom	2019-Nov-05	EPI_ISL_405112	A/England/340/2019	UK Health Security Agency - Colindale	UK Health Security Agency - Colindale	Thompson,C.
EPI1665658	HA	United Kingdom	2019-Oct-29	EPI_ISL_405110	A/England/194520489/2019	UK Health Security Agency - Colindale	UK Health Security Agency - Colindale	Thompson,C.
EPI1665650	HA	United Kingdom	2019-Oct-26	EPI_ISL_405109	A/England/194520488/2019	UK Health Security Agency - Colindale	UK Health Security Agency - Colindale	Thompson,C.
EPI1665642	HA	United Kingdom	2019-Oct-26	EPI_ISL_405108	A/England/194520487/2019	UK Health Security Agency - Colindale	UK Health Security Agency - Colindale	Thompson,C.

Segment ID	Segment	Country	Collection date	Isolate-ID	Isolate name	Originating Lab	Submitting Lab	Authors
EPI1665634	HA	United Kingdom	2019-Nov-05	EPI_ISL_405107	A/England/372/2019	UK Health Security Agency - Colindale	UK Health Security Agency - Colindale	Thompson,C.
EPI1665626	HA	United Kingdom	2019-Oct-27	EPI_ISL_405106	A/England/337/2019	UK Health Security Agency - Colindale	UK Health Security Agency - Colindale	Thompson,C.
EPI1665618	HA	United Kingdom	2019-Nov-01	EPI_ISL_405105	A/England/336/2019	UK Health Security Agency - Colindale	UK Health Security Agency - Colindale	Thompson,C.
EPI1665610	HA	United Kingdom	2019-Oct-28	EPI_ISL_405104	A/England/359/2019	UK Health Security Agency - Colindale	UK Health Security Agency - Colindale	Thompson,C.
EPI1665602	HA	United Kingdom	2019-Oct-28	EPI_ISL_405103	A/England/357/2019	UK Health Security Agency - Colindale	UK Health Security Agency - Colindale	Thompson,C.
EPI1665594	HA	United Kingdom	2019-Oct-31	EPI_ISL_405102	A/England/334/2019	UK Health Security Agency - Colindale	UK Health Security Agency - Colindale	Thompson,C.
EPI1665586	HA	United Kingdom	2019-Oct-30	EPI_ISL_405101	A/England/194480340/2019	UK Health Security Agency - Colindale	UK Health Security Agency - Colindale	Thompson,C.
EPI1665578	HA	United Kingdom	2019-Oct-30	EPI_ISL_405100	A/England/333/2019	UK Health Security Agency - Colindale	UK Health Security Agency - Colindale	Thompson,C.
EPI1665570	HA	United Kingdom	2019-Oct-31	EPI_ISL_405099	A/England/332/2019	UK Health Security Agency - Colindale	UK Health Security Agency - Colindale	Thompson,C.
EPI1665562	HA	United Kingdom	2019-Oct-28	EPI_ISL_405098	A/England/194440328/2019	UK Health Security Agency - Colindale	UK Health Security Agency - Colindale	Thompson,C.
EPI1665554	HA	United Kingdom	2019-Oct-22	EPI_ISL_405097	A/England/329/2019	UK Health Security Agency - Colindale	UK Health Security Agency - Colindale	Thompson,C.
EPI1665546	HA	United Kingdom	2019-Nov-15	EPI_ISL_405096	A/England/194840326/2019	UK Health Security Agency - Colindale	UK Health Security Agency - Colindale	Thompson,C.
EPI1665544	HA	United Kingdom	2019-Nov-21	EPI_ISL_405095	A/England/475/2019	UK Health Security Agency - Colindale	UK Health Security Agency - Colindale	Thompson,C.

Segment ID	Segment	Country	Collection date	Isolate-ID	Isolate name	Originating Lab	Submitting Lab	Authors
EPI1665542	HA	United Kingdom	2019-Nov-20	EPI_ISL_405094	A/England/194840321/2019	UK Health Security Agency - Colindale	UK Health Security Agency - Colindale	Thompson,C.
EPI1665540	HA	United Kingdom	2019-Nov-19	EPI_ISL_405093	A/England/194840320/2019	UK Health Security Agency - Colindale	UK Health Security Agency - Colindale	Thompson,C.
EPI1665538	HA	United Kingdom	2019-Nov-18	EPI_ISL_405092	A/England/194820761/2019	UK Health Security Agency - Colindale	UK Health Security Agency - Colindale	Thompson,C.
EPI1665536	HA	United Kingdom	2019-Nov-22	EPI_ISL_405091	A/England/194820264/2019	UK Health Security Agency - Colindale	UK Health Security Agency - Colindale	Thompson,C.
EPI1665534	HA	United Kingdom	2019-Nov-19	EPI_ISL_405090	A/England/194800322/2019	UK Health Security Agency - Colindale	UK Health Security Agency - Colindale	Thompson,C.
EPI1665532	HA	United Kingdom	2019-Nov-19	EPI_ISL_405089	A/England/194800299/2019	UK Health Security Agency - Colindale	UK Health Security Agency - Colindale	Thompson,C.
EPI1665530	HA	United Kingdom	2019-Nov-15	EPI_ISL_405088	A/England/416/2019	UK Health Security Agency - Colindale	UK Health Security Agency - Colindale	Thompson,C.
EPI1665526	HA	United Kingdom	2019-Nov-12	EPI_ISL_405086	A/England/194700625/2019	UK Health Security Agency - Colindale	UK Health Security Agency - Colindale	Thompson,C.
EPI1665524	HA	United Kingdom	2019-Nov-10	EPI_ISL_405085	A/England/194660518/2019	UK Health Security Agency - Colindale	UK Health Security Agency - Colindale	Thompson,C.
EPI1665519	HA	United Kingdom	2019-Oct-28	EPI_ISL_405082	A/England/358/2019	UK Health Security Agency - Colindale	UK Health Security Agency - Colindale	Thompson,C.
EPI1704411	HA	United Kingdom	2019-Dec-11	EPI_ISL_413643	A/Aberystwyth/9599/2019	Public Health Wales Microbiology Cardiff	Public Health Wales Microbiology Cardiff	
EPI1704403	HA	United Kingdom	2019-Dec-09	EPI_ISL_413640	A/Mountain Ash/9548/2019	Public Health Wales Microbiology Cardiff	Public Health Wales Microbiology Cardiff	

Segment ID	Segment	Country	Collection date	Isolate-ID	Isolate name	Originating Lab	Submitting Lab	Authors
EPI1704398	HA	United Kingdom	2019-Nov-27	EPI_ISL_413639	A/Pontypridd/9299/2019	Public Health Wales Microbiology Cardiff	Public Health Wales Microbiology Cardiff	
EPI1704389	HA	United Kingdom	2020-Jan-27	EPI_ISL_413638	A/Monmouth/0982/2020	Public Health Wales Microbiology Cardiff	Public Health Wales Microbiology Cardiff	
EPI1704373	HA	United Kingdom	2019-Dec-31	EPI_ISL_413636	A/Pontyclun/0383/2019	Public Health Wales Microbiology Cardiff	Public Health Wales Microbiology Cardiff	
EPI1704362	HA	United Kingdom	2019-Dec-30	EPI_ISL_413634	A/Monmouth/0201/2019	Public Health Wales Microbiology Cardiff	Public Health Wales Microbiology Cardiff	
EPI1704354	HA	United Kingdom	2019-Dec-18	EPI_ISL_413633	A/Rhondda/0198/2019	Public Health Wales Microbiology Cardiff	Public Health Wales Microbiology Cardiff	
EPI1608131	HA	United Kingdom	2019-Oct-16	EPI_ISL_395175	A/Cardiff/3356/2019	Public Health Wales Microbiology Cardiff	Public Health Wales Microbiology Cardiff	
EPI1608116	HA	United Kingdom	2019-Oct-21	EPI_ISL_395173	A/Pontypool/9038/2019	Public Health Wales Microbiology Cardiff	Public Health Wales Microbiology Cardiff	
EPI1607288	HA	United Kingdom	2019-Oct-07	EPI_ISL_395058	A/Newport/7226/2019	Public Health Wales Microbiology Cardiff	Public Health Wales Microbiology Cardiff	
EPI1607280	HA	United Kingdom	2019-Oct-11	EPI_ISL_395057	A/Port Talbot/4478/2019	Public Health Wales Microbiology Cardiff	Public Health Wales Microbiology Cardiff	
EPI1729142	HA	United Kingdom	2020-Jan-22	EPI_ISL_427853	A/England/11499/2020	U.S. Air Force School of	U.S. Air Force School of	Gruner, W.E.; Fries, A.C.; Garrett, C.M.; Powell, M.L.; Hanson, J.F.; DeMarcus, L.S.;

Segment ID	Segment	Country	Collection date	Isolate-ID	Isolate name	Originating Lab	Submitting Lab	Authors
						Aerospace Medicine	Aerospace Medicine	Sjoberg, P.A.; Bogue, A.L.; Robbins, A.S.; Macias, E.A.; Lambert, A.W.; Dane, D.J.
EPI1655160	HA	United Kingdom	2019-Sep-26	EPI_ISL_403215	A/England/9865/2019	U.S. Air Force School of Aerospace Medicine	U.S. Air Force School of Aerospace Medicine	Gruner, W.E.; Fries, A.C.; Garrett, C.M.; Powell, M.L.; Hanson, J.F.; DeMarcus, L.S.; Sjoberg, P.A.; Bogue, A.L.; Robbins, A.S.; Macias, E.A.; Lambert, A.W.; Dane, D.J.

Supplementary Table 2: Amino acid differences between the strains in Table 2. Residues which match the A/New Caledonia/71/2014 reference sequence are left blank for clarity.

Strain	9	78	91	92	96	121	122	128	131	135	138	142	144	158	159	160	171	183	190	193	225	246	261	262	311	312	326	406	478	479	484
**A/New Caledonia/71/2014 (cell)**	**N**	**G**	**S**	**K**	**N**	**N**	**N**	**T**	**T**	**T**	**A**	**R**	**S**	**N**	**Y**	**T**	**N**	**H**	**D**	**F**	**D**	**N**	**R**	**S**	**H**	**S**	**K**	**I**	**I**	**G**	**G**
A/New Caledonia/71/2014 (egg)																K		L			G										
A/Kansas/14/2017 (cell)	S		N					A			S	G	K		S	K				S					Q		R		M		
A/Kansas/14/2017 (egg)	S		N					A			S	G	K		S	K			N	S		T			Q		R		M		
A/HongKong/4801/2014	S																														
A/HongKong/5738/2014	S													R												N					
A/Victoria/5060/2014	S																														
A/Singapore/INFIMH-16-0019/2016	S					K						G					K											V		E	E
A/South Australia/135/2016	S					K											K											V			E
A/Greece/4/2017	S	D				K	D			K							K											V		E	E
A/Singapore/GP2646/2016	S			R		K											K			S					Q			V			E
A/Brisbane/190/2017	S								K			K				K							Q								
A/Washington/16/2017	S								K			K											Q								
A/Switzerland/8060/2017	S				S				K			K											Q								
A/England/70180215/2016	S					K	D						K											N							
